# Heterogeneous Visible‐Light Photocatalysis in Continuous‐Flow: A Synergistic Strategy for Challenging Trifluoromethylation of *α, α*‐Diaryl Allylic Alcohols via 1,2‐aryl Migration

**DOI:** 10.1002/advs.75371

**Published:** 2026-04-22

**Authors:** Peiwen Liu, Weiping Zhu, Xuhong Qian, Fang Zhao

**Affiliations:** ^1^ School of Pharmacy East China Normal University Shanghai P. R. China; ^2^ State Key Laboratory of Bioreactor Engineering Shanghai Key Laboratory of Chemical Biology School of Pharmacy East China University of Science and Technology Shanghai P. R. China

**Keywords:** 1,2‐aryl migration, continuous‐flow liquid‐solid process, heterogeneous photocatalyst, trifluoromethylation, visible‐light photocatalysis

## Abstract

Trifluoromethyl ketones are valuable scaffolds in drug discovery and material science, but their synthesis is often hindered by the need for prefunctionalized substrates, harsh oxidative conditions, and expensive trifluoromethylating reagents. To address these challenges, a continuous‐flow heterogeneous photocatalytic trifluoromethylation strategy is developed to synthesize *β*‐trifluoromethyl‐*α*‐aryl ketones via visible‐light‐induced 1,2‐aryl migration. Mesoporous graphitic carbon nitride (*mpg*‐C_3_N_4_) is employed as a recyclable photocatalyst, and trifluoromethanesulfonyl chloride (TfCl) serves as an affordable CF_3_ source, enabling a mild and sustainable radical pathway. The reaction is conducted in a continuous oscillatory flow system integrated with a custom photoreactor, ensuring efficient light utilization and stable catalyst handling. For the model substrate, an 83% isolated yield is achieved within 30 min, corresponding to a space‐time yield of 6.95 g·L^−1^·h^−1^, significantly surpassing that obtained under batch conditions (0.36 g·L^−1^·h^−1^ with 43% isolated yield in 12 h). The method shows broad substrate compatibility, obtaining *β*‐trifluoromethyl‐*α*‐aryl ketones in 54%–88% yields from 23 allyl alcohols. The scalability is confirmed with a gram‐scale preparation, and the intermediate is converted into a bioactive molecule via an integrated multi‐step continuous‐flow process. This work provides a sustainable, efficient strategy for synthesizing fluorinated compounds of pharmaceutical relevance.

## Graphical abstract

A continuous‐flow photocatalytic strategy is presented for the efficient synthesis of *β*‐trifluoromethyl ketones under visible light. Using a recyclable solid photocatalyst and an inexpensive CF_3_ source, the method enables rapid reactions, easy scale‐up, and smooth integration into multistep flow synthesis of bioactive molecules.

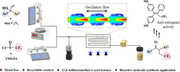

## 1 Introduction

**FIGURE 1:**
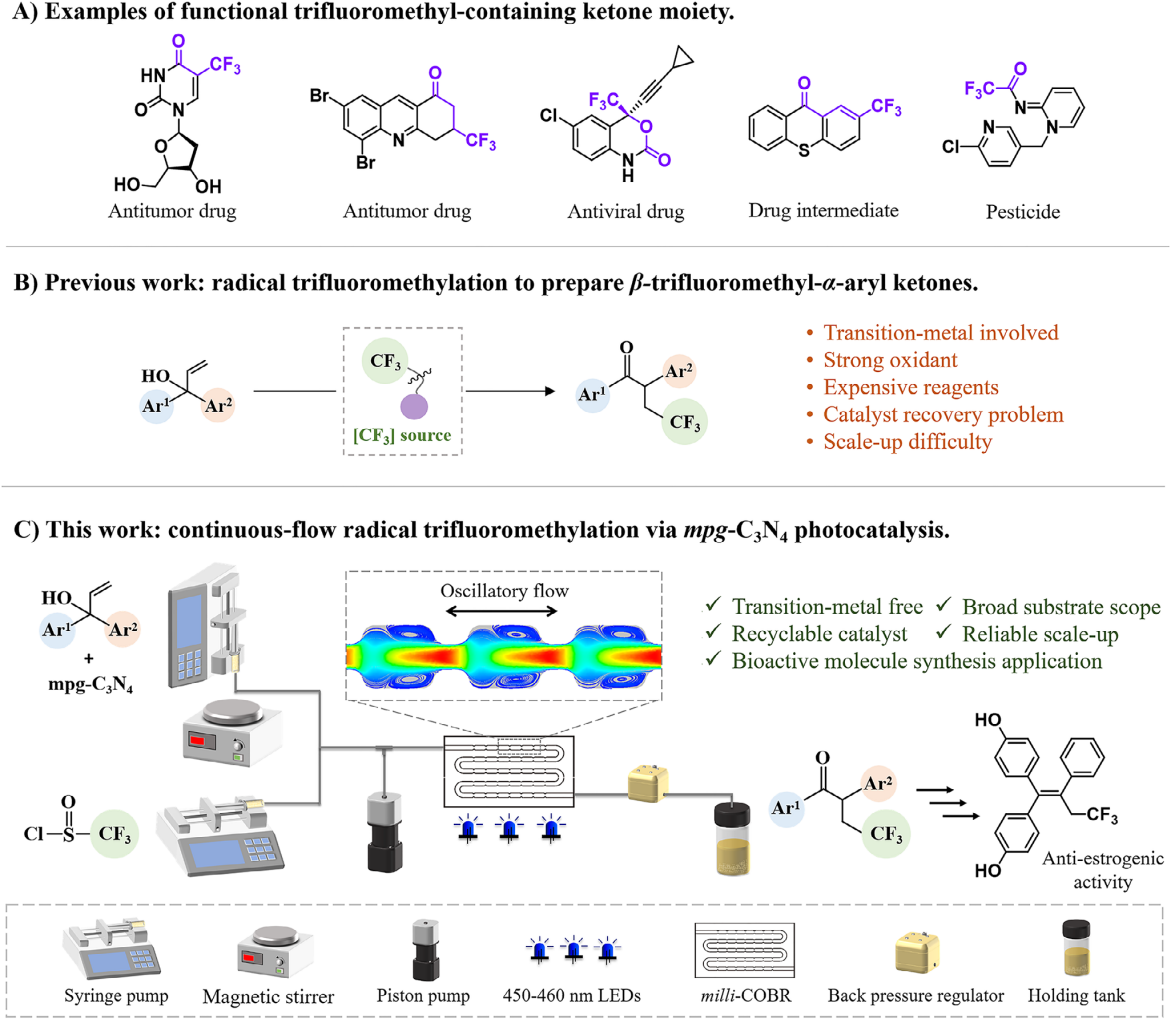
(A) Representative examples of CF_3_‐containing ketones. (B) Synthetic strategies toward *β*‐trifluoromethyl‐*α*‐aryl ketones. (C) This work represents a continuous‐flow liquid‐solid visible‐light‐induced trifluoromethylation of *α, α*‐diaryl allylic alcohols via 1,2‐aryl migration.

The trifluoromethyl group (CF_3_) is a highly valued substituent in organic and medicinal chemistry due to its unique physicochemical properties, including strong electronegativity, enhanced metabolic stability, and pronounced biological activity [1, 2, 3, 4, 5]. Incorporation of CF_3_ groups into drug molecules often improves lipophilicity, strengthens interactions with biological targets, and optimizes pharmacokinetic profiles [6, 7, 8]. Among CF_3_‐containing scaffolds, trifluoromethyl ketones (Figure 1A) hold a privileged position in drug discovery and functional material development [9, 10, 11, 12, 13, 14, 15, 16, 17]. Consequently, the development of efficient and broadly applicable methods for their synthesis has attracted sustained attention.

Conventional methods to access trifluoromethyl ketones generally rely on ionic carbonyl chemistry or multistep transformations from prefunctionalized substrates, which often compromise efficiency and scope [18, 19]. In 2013, Wu and co‐workers reported a copper‐catalyzed radical 1,2‐aryl migration trifluoromethylation to access *β*‐trifluoromethyl‐*α*‐aryl ketones [20], followed by Sodeoka's iron‐catalyzed variant [21]. Building upon these foundational studies, a range of radical trifluoromethylation strategies has been developed (Figure 1B). Yet, these methods remain dependent on transition‐metal catalysts, costly or specialized reagents, or stoichiometric oxidants, and they often pose challenges in catalyst recovery [22, 23, 24, 25]. More recently, visible‐light‐mediated photocatalytic protocols have emerged as a milder and greener alternative [26]. Nevertheless, this approach also faces challenges in catalyst recovery, and photochemical reactions are inherently difficult to reliable scale‐up.

Continuous‐flow technology has demonstrated significant advantages in fluorination reactions. By enhancing mass and heat transfer, continuous‐flow systems improve reaction safety and enable precise control over key parameters such as residence time and temperature, thereby improving reaction efficiency and scalability [27, 28, 29]. In particular, for visible‐light‐driven photochemical fluorination reactions, the inherent advantages of continuous‐flow reactors in terms of light utilization efficiency and reaction controllability make them an ideal platform for scale‐up [30, 31, 32]. Continuous‐flow fluorination methods have been widely applied in nucleophilic, electrophilic, and radical fluorination reactions. However, challenges associated with catalyst separation and recycling still persist in many of these systems, which may limit their practical application and sustainability.

To address these challenge, we employed mesoporous graphitic carbon nitride (*mpg*‐C_3_N_4_) as a recyclable heterogeneous photocatalyst [33, 34, 35, 36, 37], in combination with trifluoromethanesulfonyl chloride (TfCl), an inexpensive and readily available CF_3_ source, to enable visible‐light‐mediated radical 1,2‐aryl migration trifluoromethylation. To implement this chemistry efficiently and safely, we envisioned a continuous‐flow photochemical platform equipped with a custom‐designed photoreactor, *milli*‐COBR, developed based on our previously constructed microreactor (Photo‐*µ*COBR) [38]. Owing to its oscillatory mixing characteristics, the *milli*‐COBR effectively handles solid‐containing systems, ensuring stable operation without clogging [39, 40, 41, 42, 43], while providing precise control over irradiation, residence time, and reagent stoichiometry. By integrating a heterogeneous photocatalyst with this tailored continuous‐flow setup, the system enhances reaction efficiency, yield, and scalability, while simultaneously mitigating challenges associated with catalyst handling and recovery (Figure 1C).

Using this integrated platform, we explored the synthetic utility of the developed transformation. A diverse set of symmetric and unsymmetric *α, α*‐diaryl allylic alcohols underwent smooth trifluoromethylation, delivering the corresponding *β*‐trifluoromethyl‐*α*‐aryl ketones across a broad substrate range. The consistently high reaction efficiency observed under flow conditions, in stark contrast to the prolonged reaction times required in batch, underscores the benefits of combining heterogeneous photocatalysis with a controlled photochemical flow environment. The practicality of the system was further demonstrated through the gram‐scale preparation of a key *β*‐trifluoromethyl ketone intermediate, which was subsequently transformed into a bioactive antiestrogen candidate via an integrated flow process comprising both telescoped and discrete continuous‐flow steps. These results underscore the dependable performance and synthetic versatility of the developed protocol, providing a reliable approach for accessing structurally complex fluorinated molecules.

## 2 Results and Discussion

### 2.1 Characterization of Mpg‐C_3_N_4_

*Mpg*‐C_3_N_4_ was synthesized using cyanamide as the precursor and colloidal silica as a hard template, following a reported procedure [44]. The obtained material was systematically characterized to elucidate its compositional, textural, and structural features relevant to photocatalytic applications. Elemental analysis gave a C/N ratio of 0.60, slightly lower than the theoretical value of 0.65, which can be attributed to the presence of structural defects generated during thermal polymerization. The low hydrogen content further indicated a high degree of condensation of the cyanamide precursor (Table S1). High‐resolution transmission electron microscopy images revealed a well‐developed mesoporous architecture with pore diameters of approximately 10 nm, in good agreement with the average pore size of 11.7 nm derived from Brunauer–Emmett–Teller (BET) analysis (Figure S1 and Table S2). The material exhibited a high specific surface area of 111.2 m^2^·g^−^^1^, characteristic of mesostructured graphitic carbon nitride. X‐ray diffraction patterns showed two distinct reflections at 2θ = 13° and 28°, corresponding to the in‐plane structural packing of tri‐s‐triazine units and the interlayer stacking of conjugated aromatic layers, respectively (Figure S2).

These results confirm the successful synthesis of *mpg*‐C_3_N_4_ with a well‐defined mesoporous structure and preserved graphitic framework, providing a suitable solid photocatalyst for subsequent continuous‐flow photochemical studies.

### 2.2 Reaction Evaluation and Optimization in Flow

With *mpg*‐C_3_N_4_ in hand, its catalytic performance was first evaluated in the visible‐light‐mediated trifluoromethylation of 1,1‐diarylallyl alcohols, using benzophenone‐derived allylic alcohol **2a** as a model substrate and TfCl as an inexpensive CF_3_ source. To facilitate rapid optimization and ensure scalability, the reaction was conducted in a custom‐designed *milli*‐COBR integrated into a continuous‐flow platform (Figures S4 and S5).

Preliminary control experiments confirmed that both blue‐light irradiation (450‐460 nm) and *mpg*‐C_3_N_4_ were indispensable for productive transformation (Table 1, entries 1–3). In the absence of either component, no desired product was detected. The presence of a base proved critical for efficient conversion (Table 1, entries 1 and 4), with TMEDA emerging as the optimal choice among the bases examined when acetonitrile was used as the solvent (Table 1, entries 1 and 5–9). Evaluation of different light sources further showed that blue LEDs significantly outperformed incandescent lamps of comparable power (Table 1, entries 10 and 11), consistent with the broader and less effective emission profile of the latter (Figure S8). The effect of photocatalyst loading was subsequently investigated. An optimal loading of 20 wt.% *mpg*‐C_3_N_4_ (relative to the substrate) afforded the highest HPLC yield of 86%. Both lower and higher catalyst loadings resulted in diminished performance (Table 1, entries 1, 12–16), with excessive amounts likely causing increased light scattering and reduced effective photon flux within the reactor. In addition, a commercially available bulk graphitic carbon nitride (*g*‐C_3_N_4_) was also evaluated under the optimized conditions, which afforded the desired product, albeit in a lower yield (Table 1, entries 1 and 17). This reduced performance is likely attributed to its lower specific area and limited exposure of catalytic active sites, arising from the absence of mesoporosity, compared to *mpg*‐C_3_N_4_. Residence time was also found to be a key parameter: complete conversion required a residence time of 30 min, whereas shorter residence times led to incomplete reactions (Table 1, entries 1, 18–21).

**TABLE 1:** Optimization of reaction condition in flow.^a^

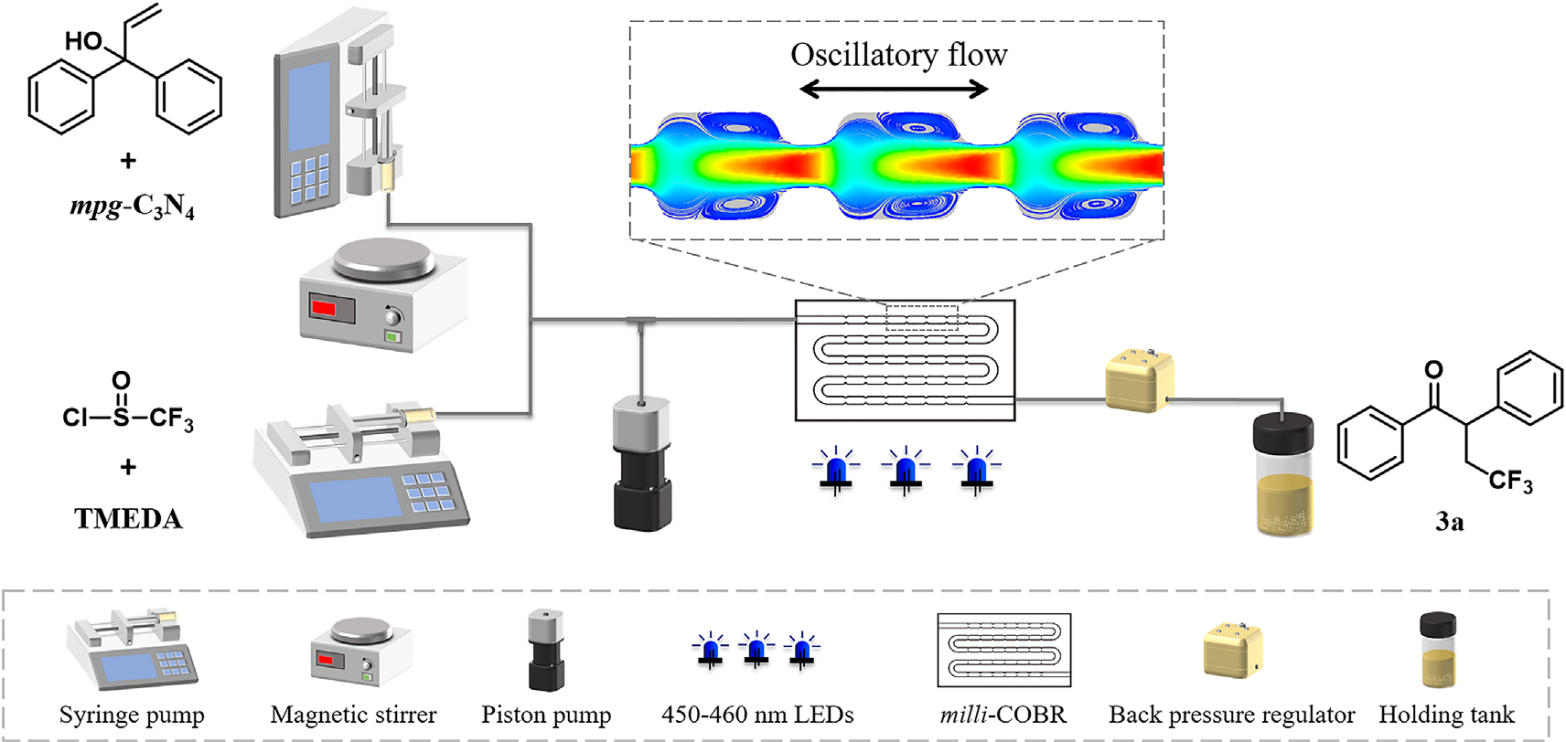					
Entry	Deviation	Yield of 3a^b^	Entry	Deviation	Yield of 3a^b)^
1	None	86%	12	5 wt.% *mpg*‐C_3_N_4_	14%
2	No light	n.d.	13	10 wt.% *mpg*‐C_3_N_4_	54%
3	No catalyst	<5%	14	15 wt.% *mpg*‐C_3_N_4_	82%
4	No base	31%	15	25 wt.% *mpg*‐C_3_N_4_	84%
5	DBU as base	46%	16	50 wt.% *mpg*‐C_3_N_4_	75%
6	Et_3_N as base	39%	17	20 wt.% *g*‐C_3_N_4_	59%
7	DCE as solvent	54%	18	τ = 10 min	38%
8	DMF as solvent	19%	19	τ = 20 min	75%
9	MeOH as solvent	22%	20	τ = 25 min	81%
10	Incandescent lamp (100W)	56%	21	τ = 45 min	83%
11	450‐460 nm LEDs (100 W)	82%	22^c^	Batch	43%

^a^ Standard condition: 450–460 nm blue LEDs irradiation (40 W), 0.02 mol·L^−1^ solution of **2a** in MeCN, *mpg*‐C_3_N_4_ (20 wt.% relative to **2a**), CF_3_SO_2_Cl (1.5 eq.), TMEDA (3.0 eq.), V*_milli_*_‐COBR_ = 1.8 mL, residence time (τ) = 30 min, nitrogen atmosphere, room temperature, 25 psi. Oscillatory flow condition: *f* = 2 Hz, *x*_o_ = 10 mm;

^b^ Yield of **3a** determined by HPLC analysis based on an external calibration curve of the product. n.d. = not detected;

^c^ Batch condition: 450–460 nm blue LEDs irradiation (40 W), 0.48 mmol **2a**, 5 mL MeCN, *mpg*‐C_3_N_4_ (50 wt.% relative to **2a**), CF_3_SO_2_Cl (1.5 eq.), TMEDA (3.0 eq.), 12 h, 0 °C. Yield of **3a** in batch refers to isolated yield.

Notably, the continuous‐flow process exhibited a pronounced advantage over the corresponding batch protocol. Under batch conditions, the reaction was conducted in a 10 mL round‐bottom flask and delivered only 43% isolated yield after 12 h of irradiation, whereas the flow system, featuring channel internal diameters ranging from 1.0 to 2.5 mm, furnished the product in 83% isolated yield within 30 min (entries 1 and 22), which is beneficial for light penetration in photochemical processes. The inferior batch performance was attributed to increased side‐product formation, primarily benzophenone, under prolonged irradiation, as well as less effective exclusion of oxygen. These results highlight the efficiency and robustness of the continuous‐flow platform for visible‐light‐driven trifluoromethylation reactions.

### 2.3 Recyclability and Regeneration of Mpg‐C_3_N_4_

**FIGURE 2:**
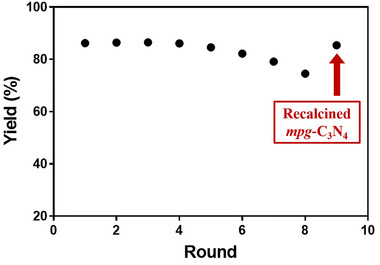
Experimental results of the recovery and reuse of *mpg*‐C_3_N_4_. Yield is determined by HPLC analysis based on an external calibration curve of the product.

Given its heterogeneous nature, the recyclability of *mpg*‐C_3_N_4_ was systematically evaluated under continuous‐flow conditions. The photocatalyst maintained high catalytic activity over five consecutive cycles, after which a gradual decline in performance was observed (Figure 2; Table S3). FT‐IR analysis revealed no discernible changes in the chemical structure of *mpg*‐C_3_N_4_ after repeated use, indicating that catalyst deactivation did not arise from framework degradation. In contrast, BET measurements showed a pronounced decrease in specific surface area, suggesting partial pore blockage by strongly adsorbed organic byproducts during operation (Figure S9 and Table S4). Notably, a simple calcination treatment effectively restored both the surface area and catalytic activity of the material. These results demonstrated the chemical robustness of *mpg*‐C_3_N_4_ and highlighted its suitability for long‐term operation in continuous‐flow photochemical processes, where catalyst regeneration was critical for process sustainability.

### 2.4 Mechanistic Insights

To gain insight into the reaction mechanism, two control experiments were conducted. The addition of the radical scavenger 2,2,6,6‐tetramethylpiperidin‐1‐oxyl (TEMPO) completely suppressed product formation, indicating the involvement of radical intermediates (Figure S10A). In addition, *α, α*‐dicyclohexylallyl alcohol failed to undergo rearrangement under the standard conditions, suggesting that aryl migration is essential for the transformation (Figure S10B).

**FIGURE 3:**
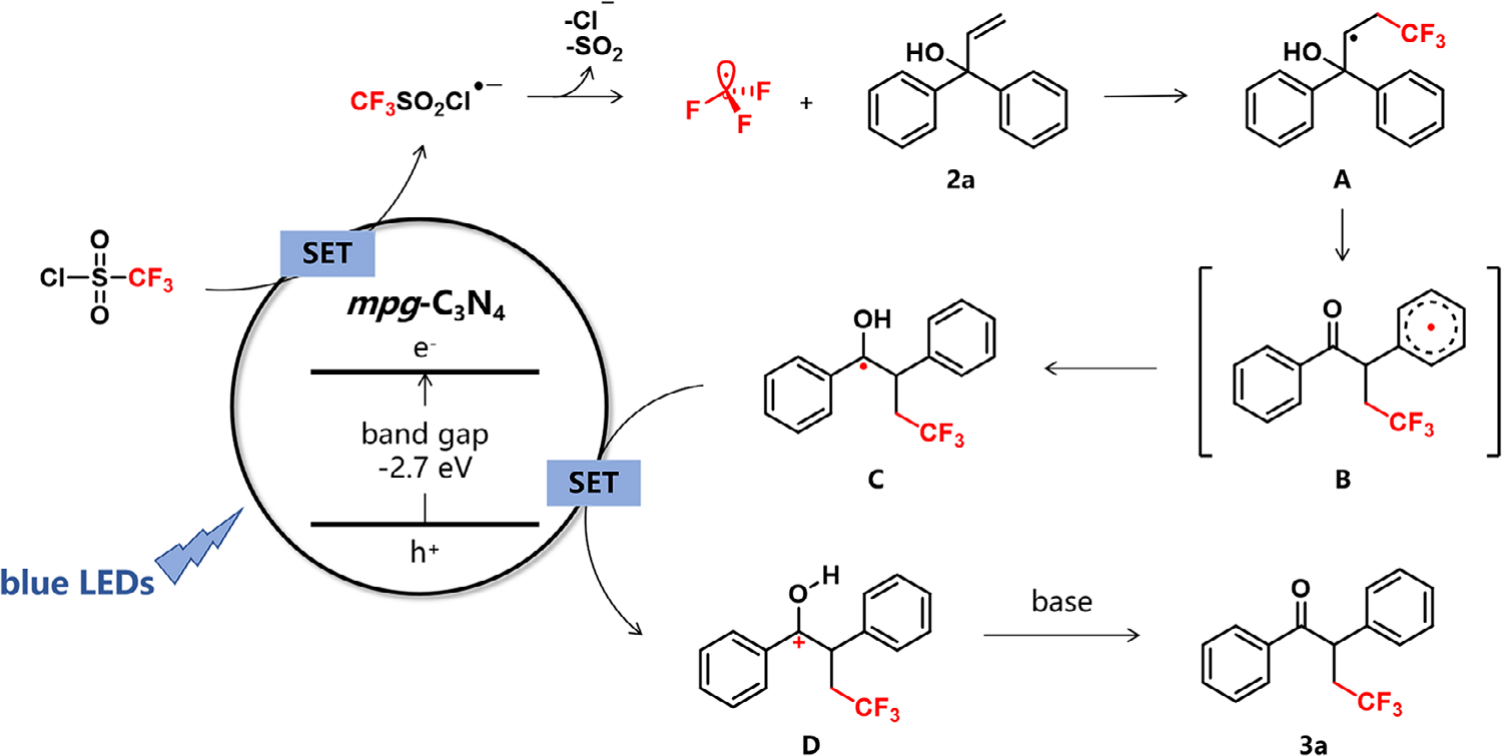
Speculation of reaction mechanism.

On the basis of these results, a plausible reaction mechanism was proposed in Figure 3. Upon visible‐light excitation, *mpg*‐C_3_N_4_ was promoted to its excited state and engaged in single‐electron transfer (SET) with trifluoromethanesulfonyl chloride, generating a CF_3_ radical. The CF_3_ radical added to allylic alcohol **2a** to form carbon‐centered radical intermediate **A**. Subsequent 1,2‐aryl migration proceeded via a spiro[2, 5]‐octadienyl radical intermediate **B**, affording radical **C**. Final oxidation followed by base‐assisted deprotonation furnished the *β*‐trifluoromethyl‐*α*‐aryl ketone product **3a**, completing the catalytic cycle.

### 2.5 Substrate Scope and Regioselectivity

Under the optimized conditions, the substrate scope of the radical trifluoromethylation was systematically examined (Table 2). A broad range of symmetric allyl alcohols (**2b**‐**h**) were smoothly converted into the corresponding *β*‐trifluoromethyl*‐α‐*aryl ketones in moderate to good isolated yields (69%–86%), largely independent of the electronic properties or substitution patterns of the aryl groups.

**TABLE 2:** List of constructed *β*‐trifluoromethyl‐*α*‐aryl ketone compounds in flow.^a^

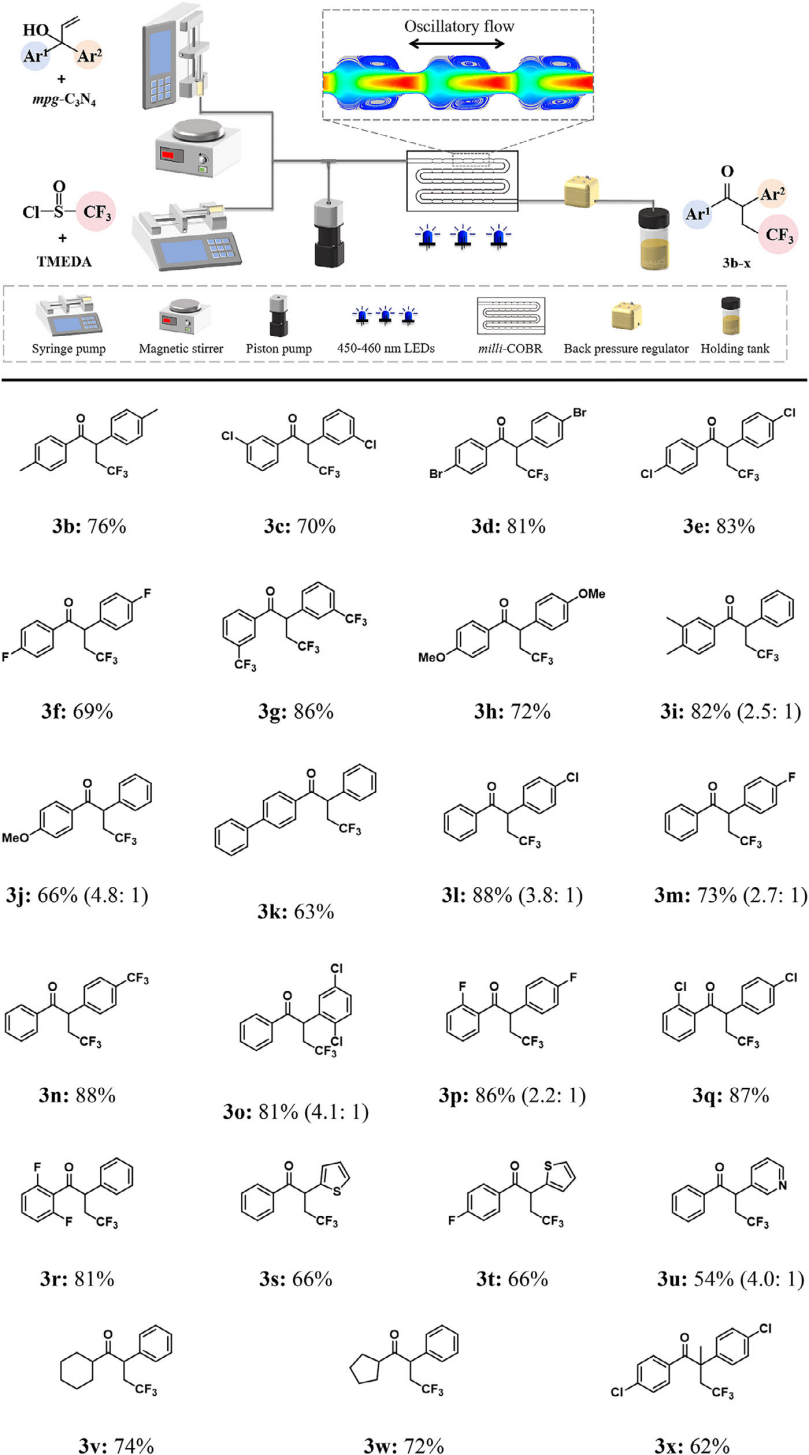

^a^ Standard condition: 450–460 nm LEDs irradiation (40 W), 0.02 mol·L^−1^ solution of substrate in MeCN, *mpg*‐C_3_N_4_ (20 wt.% relative to substrate), CF_3_SO_2_Cl (1.5 eq.), TMEDA (3.0 eq.), V*_milli_*_‐COBR_ = 1.8 mL, τ = 30 min, nitrogen atmosphere, room temperature, 25 psi. Oscillatory flow condition: *f* = 2 Hz, *x*_o_ = 10 mm. Yields refer to isolated yields. For compounds **3i**, **3j**, **3l**, **3o**, and **3u**, regioisomer ratios were calculated based on the isolated yields of each isomer when separable. For compounds **3m** and **3p**, which were obtained as inseparable mixtures, the regioisomer ratios were determined by NMR analysis.

Encouraged by these results, unsymmetric allyl alcohols were subsequently investigated. These substrates also delivered the desired products in moderate to good yields (63%–88%), while exhibiting pronounced regioselectivity. For substrates bearing *para‐* or *meta‐*substituents, migration preferentially occurred from the more electron‐deficient aryl group, affording regioselective *β*‐trifluoromethyl‐*α*‐aryl ketones (**3i**‐**n**). In contrast, aryl groups bearing *ortho*‐substituents migrated more slowly, irrespective of their electronic nature, resulting in diminished selectivity (**3o**–**r**).

Notably, substrates **2m** and **2p**, each containing a *para*‐fluoro substituent, produced inseparable mixtures of constitutional isomers with ratios of 2.7: 1 and 2.2: 1, respectively. In contrast, several substrates (**2k**, **2n**, **2s**, **2t**, **2v**, and **2w**) afforded single rearrangement products exclusively. The methodology was further extended to *α*‐heteroaryl‐ and *α*‐alkyl‐substituted allyl alcohols (**2s**‐**w**), and a *β*‐trifluoromethyl ketone bearing a quaternary carbon center (**3x**) was also successfully obtained.

These results demonstrate the broad substrate compatibility, predictable regioselectivity, and synthetic versatility of the developed heterogeneous photocatalytic trifluoromethylation strategy.

### 2.6 Continuous‐Flow Synthesis of Bioactive Molecule 6j via Photocatalytic Trifluoromethylation

**FIGURE 4:**
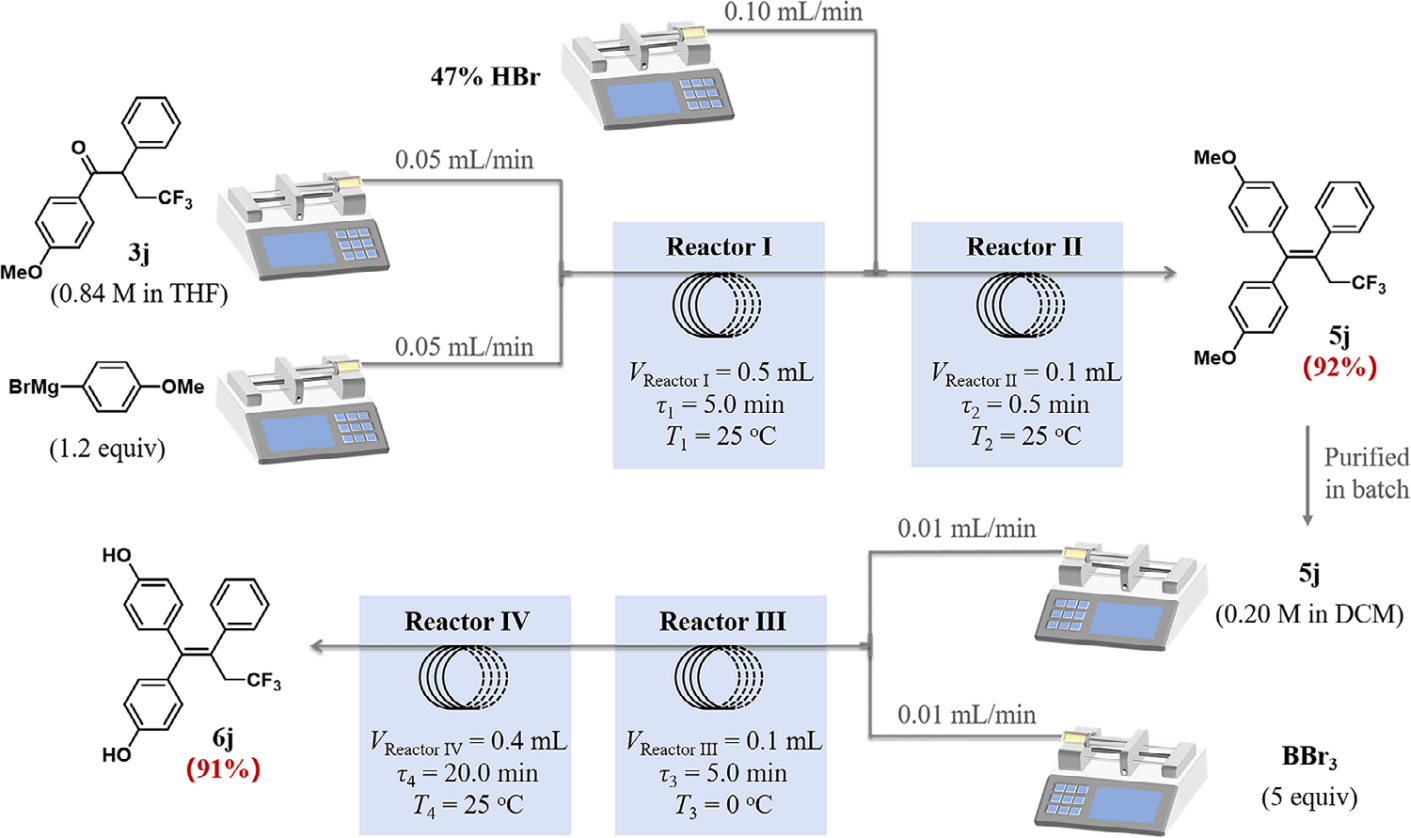
Continuous‐flow synthesis of the bioactive molecule **6j** with potent antiestrogenic activity from **3j**.

To further showcase the scalability of the method and the advantages of continuous‐flow technology, the gram‐scale synthesis of compound **3j** was achieved and subsequently applied to the preparation of bioactive molecule **6j** [45, 46], a potent antiestrogenic agent (Figure 4). The synthesis involved three key transformations, each optimized in flow.

The first step was a Grignard addition to intermediate **3j**, which requires strictly anhydrous conditions. Reaction parameters including temperature, residence time, and reagent stoichiometry, were systematically examined, with conversion as the optimization metric. Under mild conditions (25°C), complete conversion was achieved within 10 min using only 1.2 eq. of Grignard reagent. Increasing substrate concentration further accelerated the reaction, enabling full conversion within 5 min.

The subsequent HBr‐mediated elimination, typically challenging under batch conditions due to the volatility and corrosiveness of HBr, proceeded smoothly and safely in flow. Moreover, because the solvent was identical to that used in the preceding Grignard step, the two reactions could be telescoped directly without intermediate purification. Optimization revealed that the HBr‐mediated step alone reached high yields of **5j** (97.4%) at 25 °C with a residence time of 30 s. Under these telescoped conditions, gram‐scale synthesis of **5j** was accomplished in just 5.5 min total residence time, affording a 92% isolated yield, significantly outperforming the batch process (14 h, 86% yield).

The final BBr_3_‐mediated deprotection step, highly demanding in batch due to the reactivity and volatility of BBr_3_, proceeded efficiently under flow without the need for cryogenic conditions. Elevated temperatures significantly accelerated the reaction, and kinetic monitoring revealed a two‐step process involving rapid formation of mono‐deprotected intermediates followed by a slower, rate‐determining deprotection to the final product (Figure S14). Under optimized flow conditions (0°C, 25 min, 5 eq. BBr_3_), nearly quantitative conversion (99.9%) and excellent HPLC yield (96.2%) were achieved, enabling an isolated yield of 91% from **5j** to **6j**. Compared with the corresponding batch protocol for this step (48 h, 85% isolated yield), the flow process offered greatly enhanced safety, efficiency, and scalability. Notably, the overall transformation from **3j** to **6j** was accomplished in 83.7% isolated yield within a total reaction time of 30.5 min under flow conditions, compared to 73.1% over 62 h for the batch process.

## 3 Conclusion

In summary, this work demonstrated a practical strategy for integrating heterogeneous photocatalysis with continuous‐flow technology to address long‐standing challenges in radical trifluoromethylation. By leveraging a robust, recyclable photocatalyst and a tailored flow platform, we provided a blueprint for performing complex transformations in a more efficient, safe, and scalable manner. Beyond the synthesis of *β‐*trifluoromethyl‐*α*‐aryl ketones, this approach highlighted the potential of combining solid photocatalysts with flow chemistry to enable sustainable and versatile methodologies. We envisioned that such strategies can be further extended to other challenging radical processes and the scalable preparation of structurally diverse fluorinated and bioactive molecules.

## Conflicts of Interest

The authors declare no conflicts of interest.

## Supporting information




**Supporting File**: advs75371‐sup‐0001‐SuppMat.docx.

## Data Availability

The data that support the findings of this study are available from the corresponding author upon reasonable request.
