# Supplementary material for: Heterogeneous Visible‐Light Photocatalysis in Continuous‐Flow: A Synergistic Strategy for Challenging Trifluoromethylation of α, α‐Diaryl Allylic Alcohols via 1,2‐aryl Migration
Source: Adv Sci (Weinh). 2026 Apr 22;13(40):e75371. doi: 10.1002/advs.75371 (PMC13335433; doi:10.1002/advs.75371)
Supplement: Supplementary file 1 — Supporting File: advs75371‐sup‐0001‐SuppMat.docx. [file ADVS-13-e75371-s001.docx]

**SUPPORTING INFORMATION**

**Heterogeneous Visible-light Photocatalysis in Continuous-flow: A Synergistic Strategy for Challenging Trifluoromethylation of *α*, *α*-Diaryl Allylic Alcohols via 1,2-aryl Migration**

Peiwen Liu^1^, Weiping Zhu^2^, ^*^, Fang Zhao^1^, ^*^, Xuhong Qian^1, 2 *^

^1^ School of Pharmacy, East China Normal University, Shanghai 200241, China

^2^ State Key Laboratory of Bioreactor Engineering, Shanghai Key Laboratory of Chemical Biology, School of Pharmacy, East China University of Science and Technology, Shanghai 200237, China

* Corresponding author. *E-mail address*: [wpzhu@ecust.edu.cn](mailto:wpzhu@ecust.edu.cn); [fzhao@pharm.ecnu.edu.cn;](mailto:fzhao@pharm.ecnu.edu.cn;) [xhqian@ecust.edu.cn](mailto:xhqian@mail.ecust.edu.cn)

**Table of Contents**

**1. Preparation and Characterization of *mpg*-C_3_N_4_1**

1.1 Synthesis of *mpg*-C_3_N_4_S1

1.2 Physicochemical CharacterizationS1

**2. Continuous-flow Synthesis of Compounds 3a–xS5**

2.1 Batch Synthesis of Substrates 2a–xS5

2.2 Reactor Configuration and Cold-model ExperimentS12

2.3 Continuous-Flow Synthesis of Compounds 3a–xS15

2.4 Catalyst Recovery and Recycling ExperimentsS27

2.5 Mechanistic StudiesS28

**3. Synthesis of Bioactive Molecule 6jS29**

3.1 Synthesis of Bioactive Molecule 6j in BatchS29

3.2 Step 1: Grignard Addition Reaction in FlowS31

3.3 Step 2: HBr-Mediated Elimination Reaction in FlowS33

3.4 Step 3: BBr₃-Mediated Deprotection Reaction in FlowS35

**4. Spectroscopic Data S38**

1. **Preparation and Characterization of *mpg*-C_3_N_4_**

**1.1 Synthesis of *mpg*-C_3_N_4_**

Mesoporous graphitic carbon nitride (*mpg*-C₃N₄) was synthesized via a hard-templating method using cyanamide (99%, Shanghai Chuangsai Technology Co., Ltd., China) as the precursor and colloidal silica (40 wt% dispersion in water, Sigma-Aldrich) as the template. Typically, cyanamide (3.0 g, 71.4 mmol) and colloidal silica (7.5 g) were placed in a 50 mL round-bottom flask equipped with a magnetic stir bar (B15-3, Shanghai Sile Instrument Co., Ltd.) and sealed with a balloon. The mixture was stirred at 70 °C for 16 h to afford a white solid.

The resulting solid was transferred into an alumina crucible (100 mL, Shanghai Huake Industrial Co., Ltd.) and calcined in a muffle furnace (SGM·M6/10, Sigma (Shanghai) High-Temperature Furnace Co., Ltd.) at 550 °C for 8 h with a heating rate of 2.2 ^o^C·min⁻¹.

Separately, an aqueous ammonium fluoride solution (4.2 M) was prepared by dissolving ammonium fluoride (AR, Shanghai Titan Scientific Co., Ltd.) (12.0 g, 210.4 mmol) in deionized water (50 mL). After cooling to room temperature, the calcined yellow solid was immersed in the ammonium fluoride solution and stirred at ambient temperature for 24 h to remove the silica template. The resulting product was collected by centrifugation, washed thoroughly with deionized water and ethanol, and dried under vacuum to afford *mpg*-C_3_N_4_ as a yellow powder (2.1 g).

**1.2 Physicochemical Characterization**

Elemental composition of *mpg*-C_3_N_4_ was determined using an elemental analyzer (Vario EL Cube, Elementar Analysensysteme GmbH). Fourier-transform infrared (FT-IR) spectra were recorded on an infrared spectrometer (INVENIO S, Bruker Scientific Instruments Co., Ltd.). The morphology of the material was examined by high-resolution transmission electron microscopy (Themis Z, Thermo Fisher Scientific (China)). The specific surface area and pore size distribution were analyzed using a surface area and porosity analyzer (3Flex, Micromeritics Instrument Corporation). The phase purity and crystallinity were characterized by powder X-ray diffraction (Smart Lab, Rigaku Corporation).

Table S1. Elemental analysis results of *mpg*-C_3_N_4_.

| **Entry** | **Sample weight（mg）** | **N content**  **（%）** | **C content**  **（%）** | **H content**  **（%）** | **C/N ratio** |
| --- | --- | --- | --- | --- | --- |
| 1 | 2.3810 | 55.05 | 32.98 | 3.33 | 0.60 |
| 2 | 2.4400 | 55.08 | 33.07 | 3.21 | 0.60 |


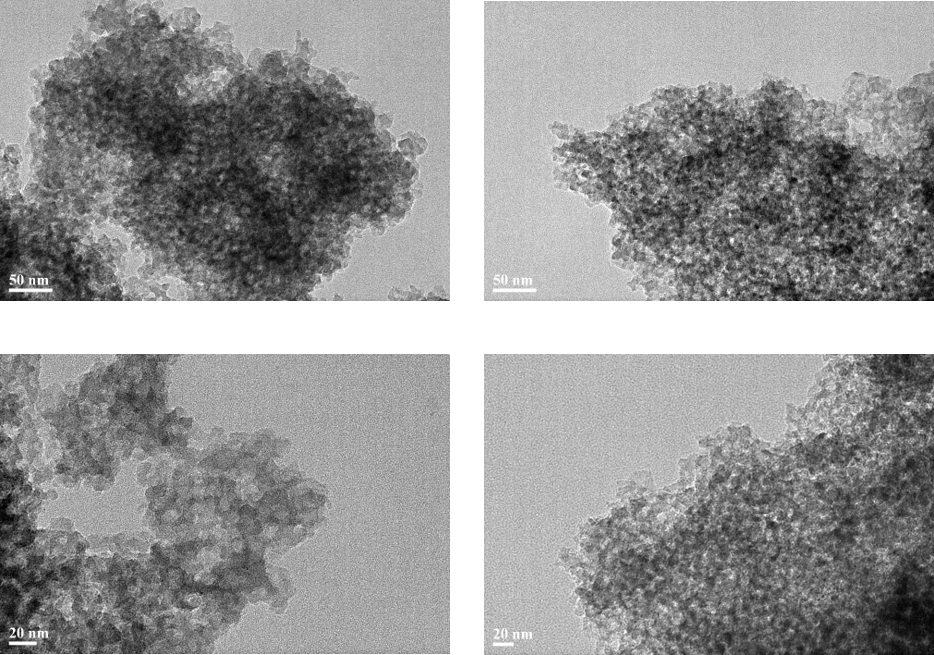


Figure S1. High-resolution TEM images of *mpg*-C_3_N_4_.

Table S2. BET surface area and porosity parameters of mpg-C₃N₄.

| **Specific surface area（m^2^·g^-1^）** | **Total pore volume**  **（cm^3^·g^-1^）** | **Pore diameter**  **（nm）** |
| --- | --- | --- |
| 111.18 | 0.38 | 11.70 |


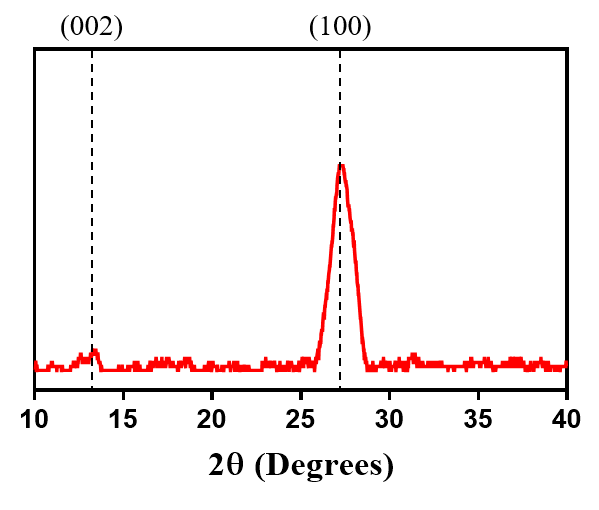


Figure S2. X-ray diffraction (XRD) pattern of *mpg*-C_3_N_4_.


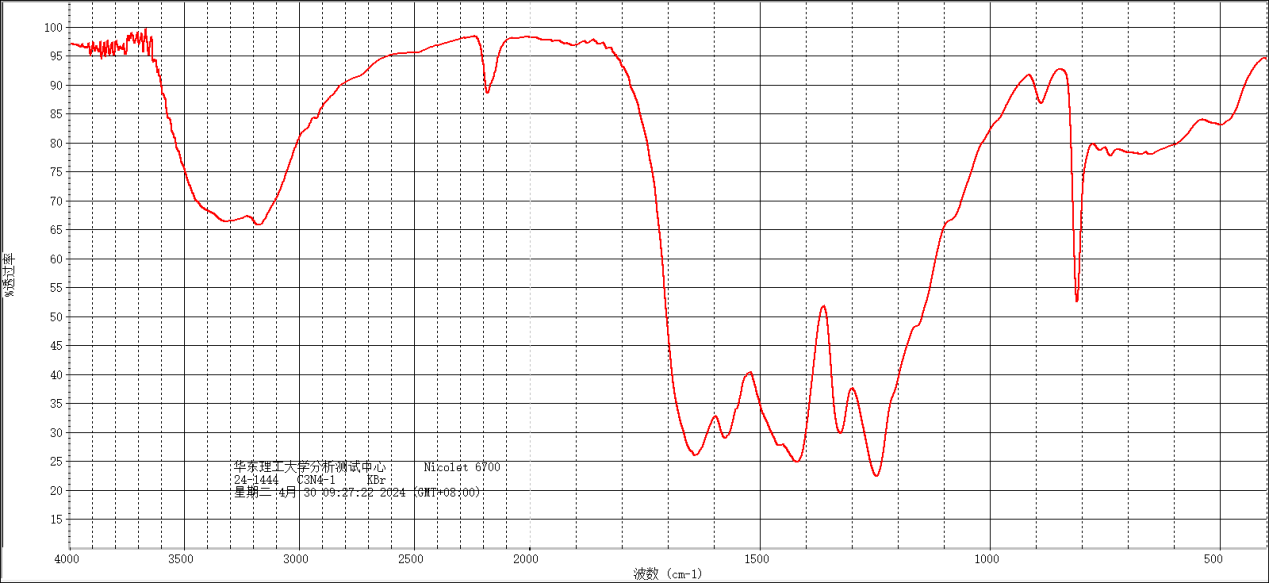


Figure S3. Fourier-transform infrared (FT-IR) spectrum of *mpg*-C_3_N_4_.

1. **Continuous-flow Synthesis of Compounds 3a–x**

**2.1 Batch Synthesis of Substrates 2a–x**

The synthesis of *α, α*-diarylallyl alcohols **2a–x** is illustrated in Scheme S1. A representative procedure is described as follows:

Benzophenone (1 g, 5.5 mmol) and anhydrous zinc chloride (0.075 g, 0.55 mmol) were weighed into a 25 mL two-neck round-bottom flask equipped with a magnetic stir bar (B15-3, Shanghai Sile Instrument Co., Ltd.). The flask was cooled in an ice bath under nitrogen atmosphere, and a solution of vinylmagnesium bromide in tetrahydrofuran (1.0 M, 7.5 mL) was added dropwise. The reaction mixture was stirred for 2 h at 0 ^o^C, and the reaction progress was monitored by thin-layer chromatography (TLC). After completion, the reaction was quenched with a saturated aqueous ammonium chloride solution, and the product was extracted with ethyl acetate several times. The combined organic layers were dried over anhydrous sodium sulfate, and the solvent was removed under reduced pressure (Rotary evaporator, RV-3, IKA). The crude product was purified by column chromatography on silica gel (300–400 mesh) to afford the target *α, α*-diarylallyl alcohol **2a** as a colorless oil (0.91 g, 91% yield). All other substrates **2b–x** were synthesized following the same general procedure using the corresponding ketone starting materials.

Scheme S1. Synthetic route for substrates **2a-x**.

Reagents and Materials: All chemical reagents were purchased from Shanghai Titan Scientific Co., Ltd., and no further purification was performed. Glassware was obtained from Beijing Xinweier Glass Instrument Co., Ltd.

Characterization: ^1^H, ^13^C, and ^19^F NMR spectra were recorded on a Bruker AVANCE NEO spectrometer. High-resolution mass spectra (HRMS) were acquired using either an EI-GCT Premier or ESI-Xevo G2 TOF mass spectrometer (Waters Technology (Shanghai) Co., Ltd.). Detailed structures and NMR data for compounds **2a–x** are provided below:

**1,1-diphenylprop-2-en-1-ol (2a)**

Colorless oil, yield =91%. ^1^H NMR (400 MHz, DMSO-*d*_6_) δ 7.38-7.36 (m, 4H), 7.33-7.31 (m, 4H), 7.24-7.20 (m, *J* =8 Hz, 2H), 6.62-6.55 (dd, *J* =20, 8 Hz, 1H), 5.98 (s, 1H), 5.24-5.18 (m, 2H). ^13^C NMR (100 MHz, DMSO-*d*_6_) δ 147.44, 144.93, 128.20, 127.22, 127.01, 113.48, 78.40.

**1,1-di-p-tolylprop-2-en-1-ol (2b)**

Colorless oil, yield = 72%. ^1^H NMR (400 MHz, DMSO-*d*_6_) δ 7.20 (d, *J* =8 Hz, 4H), 7.09 (d, *J* =8 Hz, 4H), 6.53-6.46 (m, 1H), 5.80 (s, 1H), 5.18-5.13 (m, 2H), 2.26 (s, 6H). ^13^C NMR (100 MHz, DMSO-*d*_6_) δ 144.91, 144.35, 135.61, 128.39, 126.85, 112.74, 77.83, 20.77.

**1,1-bis(3-chlorophenyl) prop-2-en-1-ol (2c)**

Colorless oil, yield = 83%. ^1^H NMR (400 MHz, DMSO-*d*_6_) δ 7.40-7.26 (m, 8H), 6.62-6.55 (m, 1H), 6.32 (s, 1H), 5.29-5.21 (m, 2H). ^13^C NMR (100 MHz, DMSO-*d*_6_) δ 149.27, 143.46, 133.26, 130.34, 127.31, 126.76, 125.88, 114.55, 77.72, 67.49, 25.60.

**1,1-bis(4-bromophenyl) prop-2-en-1-ol (2d)**

Colorless oil, yield = 88%. ^1^H NMR (400 MHz, DMSO-*d*_6_) δ 7.51-7.49 (d, *J* =8 Hz, 4H), 7.28-7.26 (d, *J* =8 Hz, 4H), 6.54-6.47 (m, 1H), 6.20 (s, 1H), 5.26-5.16 (m, 2H). ^13^C NMR (100 MHz, DMSO-*d*_6_) δ 145.83, 143.32, 130.75, 128.93, 120.06, 113.85, 77.32.

**1,1-bis(4-chlorophenyl) prop-2-en-1-ol (2e)**

Colorless oil, yield = 62%.^1^H NMR (400 MHz, DMSO-*d*_6_) δ 7.38-7.32 (m, 8H), 6.56-6.49 (m, 1H), 6.20 (s, 1H), 5.26-5.16 (m, 2H). ^13^C NMR (100 MHz, DMSO-*d*_6_) δ 145.44, 143.45, 131.45, 128.55, 127.80, 113.76, 77.21.

**1,1-bis(4-fluorophenyl) prop-2-en-1-ol (2f)**

Colorless oil, yield = 90%. ^1^H NMR (400 MHz, DMSO-*d*_6_) δ 7.37-7.33 (m, 4H), 7.14-7.10 (m, 4H), 6.57-6.50 (m, 1H), 6.11 (s, 1H), 5.23-5.14 (m, 2H). ^13^C NMR (100 MHz, DMSO-*d*_6_) δ 162.18, 159.76, 144.09, 142.92 (d, *J* = 3.1 Hz), 128.68 (d, *J* =8.1 Hz), 114.46 (d, *J* = 21.2 Hz), 113.31, 77.24. ^19^F NMR (376 MHz, DMSO-*d*_6_) δ -116.40 (t, *J* =11 Hz, 2F).

**1,1-bis(3-(trifluoromethyl) phenyl) prop-2-en-1-ol (2g)**

Colorless oil, yield = 48%. ^1^H NMR (400 MHz, DMSO-*d*_6_) δ 7.75 (s, 2H), 7.64-7.54 (m, 6H), 6.71-6.64 (m, 1H), 6.55 (s, 1H), 5.34-5.24 (m, 2H). ^13^C NMR (100 MHz, DMSO-*d*_6_) δ 147.99, 143.39, 131.45 (q, *J* = 32.3 Hz), 129.41, 126.06 (q, *J* = 3.1 Hz), 124.26 (q, *J* =272.5 Hz), 123.27 (q, *J* = 4.1 Hz), 114.96, 77.91. ^19^F NMR (376 MHz, DMSO-*d*_6_) δ -61.23 (s, 6F).

**1,1-bis(4-methoxyphenyl) prop-2-en-1-ol (2h)**

Colorless oil, yield = 75%. ^1^H NMR (400 MHz, DMSO-*d*_6_) δ 7.22-7.20 (d, *J* =8 Hz, 4H), 6.86-6.84 (d, *J* =8 Hz, 4H), 6.50-6.44 (m, 1H), 5.74 (s, 1H), 5.16-5.10 (m, 2H), 3.72 (s, 6H). ^13^C NMR (100 MHz, DMSO-*d*_6_) δ 157.79, 144.94, 139.21, 127.89, 112.95, 112.31, 77.30, 54.99.

**1-(3,4-dimethylphenyl)-1-phenylprop-2-en-1-ol (2i)**

Colorless oil, yield = 86%. ^1^H NMR (400 MHz, DMSO-*d*_6_) δ 7.37-7.35 (t, *J* =4 Hz, 2H), 7.32- 7.29 (t, *J* =8 Hz, 1H), 7.24-7.20 (t, *J* =4 Hz, 1H), 7.15 (s, 1H), 7.09-7.04 (t, *J* =8 Hz, 2H), 6.59-6.52 (m, 1H), 5.85 (s, 1H), 5.22-5.17 (M, 2H), 2.20 (s, 6H). ^13^C NMR (100 MHz, DMSO-*d*_6_) δ 147.76, 145.24, 145.02, 135.81, 134.83, 129.37, 128.46, 128.23, 127.28, 126.99, 124.77, 113.23, 78.30, 39.94, 39.73, 39.52, 20.25, 19.56.

**1-(4-methoxyphenyl)-1-phenylprop-2-en-1-ol (2j)**

Colorless oil, yield = 65%. ^1^H NMR (400 MHz, DMSO-*d*_6_) δ 7.32-7.18 (m, 7H), 6.84-6.82 (d, *J* =8 Hz, 4H), 6.53-6.46 (m, 1H), 5.83 (s, 1H), 5.17-5.11 (m, 2H), 3.69 (s, 3H). ^13^C NMR (100 MHz, DMSO-*d*_6_) δ 158.07, 147.34, 144.88, 139.23, 128.16, 127.84, 126.84, 126.60, 113.22, 112.84, 77.79, 55.21.

**1-([1,1'-biphenyl]-4-yl)-1-phenylprop-2-en-1-ol (2k)**

Colorless oil, yield = 78%. ^1^H NMR (400 MHz, DMSO-*d*_6_) δ 7.65, 7.64-7.59 (m, 4H), 7.47-7.39 (m, 6H), 7.36-7.30 (m, 3H), 7.25-7.21 (m, 1H), 6.64-6.57 (m, 1H), 6.03 (s, 1H), 5.26-5.22 (d, *J* =16 Hz, 2H). ^13^C NMR (100 MHz, DMSO-*d*_6_) δ 146.83, 146.16, 144.28, 139.89, 138.38, 128.87, 127.75, 127.27, 126.66, 126.58, 126.05, 113.02, 77.72.

**1-(4-chlorophenyl)-1-phenylprop-2-en-1-ol (2l)**

Colorless oil, yield = 77%. ^1^H NMR (400 MHz, DMSO-*d*_6_) δ 7.35-7.20 (m, 7H), 6.58-6.51 (m, 1H), 6.09 (s, 1H), 5.24-5.16 (m, 2H). ^13^C NMR (100 MHz, DMSO-*d*_6_) δ 146.48, 145.92, 143.94, 131.26, 128.61, 127.84, 127.69, 126.73, 126.64, 113.39, 77.55.

**1-(4-fluorophenyl)-1-phenylprop-2-en-1-ol (2m)**

Colorless oil, yield = 68%. ^1^H NMR (400 MHz, DMSO-*d*_6_) δ 7.37-7.29 (m, 6H), 7.24-7.22 (m, 1H), 7.14-7.10 (m, 2H), 6.59-6.52 (m,1H), 6.04 (s, 1H), 5.23-5.15 (m, 2H). ^13^C NMR (100 MHz, DMSO-*d*_6_) δ 162.10, 159.69, 146.71, 144.25, 143.12 (d, *J* =3.1 Hz), 128.70 (d, *J* =8.1 Hz), 127.79, 126.63, 114.37 (d, *J* =21.2 Hz), 113.13, 77.54. ^19^F NMR (376 MHz, DMSO-*d*_6_) δ -116.52 (m, 1F).

**1-phenyl-1-(4-(trifluoromethyl )phenyl) prop-2-en-1-ol (2n)**

Colorless oil, yield = 67%. ^1^H NMR (400 MHz, DMSO-*d*_6_) δ 7.69-7.67 (d, *J* =8 Hz, 4H), 7.58-7.56 (d, *J* =8 Hz, 4H), 7.37-7.30 (m, 4H), 7.26-7.24 (d, *J* =8 Hz, 4H), 6.63-6.56 (m, 1H), 6.23 (s,1H), 5.28-5.20 (m, 2H). ^13^C NMR (100 MHz, DMSO-*d*_6_) δ 151.51, 146.23, 143.61, 127.93 (q, *J* =32.3 Hz), 127.40, 127.10, 126.87, 126.68, 124.72 (q, *J* =3.2 Hz), 124.68 (q, *J* =272.0 Hz), 113.72, 77.72. ^19^F NMR (376 MHz, DMSO-*d*_6_) δ -60.95 (s, 1F).

**1-(2,5-dichlorophenyl)-1-phenylprop-2-en-1-ol (2o)**

Colorless oil, yield = 54%. ^1^H NMR (400 MHz, DMSO-*d*_6_) δ 7.94 (s, 1H), 7.39-7.35 (m, 2H), 7.31-7.24 (m, 3H), 7.22-7.18 (m, 2H), 6.80-6.73 (m,1H), 6.21 (s, 1H), 5.49-5.44 (d, *J* =16 Hz, 1H), 5.33-5.31 (d, *J* =8 Hz, 1H). ^13^C NMR (100 MHz, DMSO-*d*_6_) δ 145.62, 144.61, 140.26, 132.56, 131.69, 130.19, 128.58, 128.12, 127.91, 126.99, 126.70, 114.75, 77.39.

**1-(2-fluorophenyl)-1-(4-fluorophenyl) prop-2-en-1-ol (2p)**

Colorless oil, yield = 79%. ^1^H NMR (400 MHz, DMSO-*d*_6_) δ 7.75-7.71 (t, *J* =8 Hz, 1H), 7.36-7.22 (m, 4H), 7.13-7.04 (m, 3H), 6.61-6.54 (m, 1H), 6.14 (s, 1H), 5.40-5.36 (d, *J* = 16 Hz, 1H), 5.27-5.25 (d, *J* =8 Hz, 1H). ^13^C NMR (100 MHz, DMSO-*d*_6_) δ 162.27, 160.05 (d, *J* = 39.4 Hz), 157.79, 142.24 (d, *J* = 3.1 Hz), 141.80 (d, *J* = 3.1 Hz), 133.33 (d, *J* = 12.1 Hz), 129.35 (d, *J* = 8.1 Hz), 128.37 (d, *J* = 8.1 Hz), 127.67 (d, *J* = 4.1 Hz), 124.00 (d, *J* = 3.1 Hz), 115.83 (d, *J* = 22.2 Hz), 114.47 (d, *J* = 21.2 Hz), 113.55, 75.72 (d, *J* = 2.0 Hz). ^19^F NMR (376 MHz, DMSO-*d*_6_) δ -109.54 (m, 1F), -116.13 (m, 1F).

**1-(2-chlorophenyl)-1-(4-chlorophenyl) prop-2-en-1-ol (2q)**

Colorless oil, yield = 76%. ^1^H NMR (400 MHz, DMSO-*d*_6_) δ 7.91-7.89 (d, *J* =8 Hz, 1H), 7.42-7.31 (m, 5H), 7.20-7.18 (d, *J* =8 Hz, 2H), 6.76-6.69 (m, 1H), 6.13 (s, 1H), 5.43-5.39 (d, *J* =16 Hz, 1H), 5.30-5.28 (d, *J* =8 Hz, 1H). ^13^C NMR (100 MHz, DMSO-*d*_6_) δ 144.50, 142.92, 141.09, 131.64, 131.32, 130.96, 129.02, 128.54, 127.77, 126.82, 114.26, 77.22.

**1-(2,6-difluorophenyl)-1-phenylprop-2-en-1-ol (2r)**

Colorless oil, yield = 82%. ^1^H NMR (400 MHz, DMSO-*d*_6_) δ 7.41-7.29 (m, 5H), 7.25-7.21 (m, 1H), 7.03-6.99 (m, 2H), 6.21 (s, 1H), 5.23-5.19 (d, *J* =16 Hz, 1H), 5.08-5.06 d, *J* =8 Hz, 1H. ^13^C NMR (100 MHz, DMSO-*d*_6_) δ 161.03 (dd, *J* = 251.5, 8.1 Hz), 146.76, 144.45 (t, *J* = 2.7 Hz), 130.27 (t, *J* = 11.1 Hz), 128.24, 126.87, 125.60, 123.07 (t, *J* = 13.4 Hz), 113.09, 112.39 (dd, *J* = 22.0, 6.4 Hz), 76.83. ^19^F NMR (376 MHz, DMSO-*d*_6_) δ -105.27 (m, 2F).

**1-phenyl-1-(thiophen-2-yl) prop-2-en-1-ol (2s)**

Light yellow oil, yield = 64%. ^1^H NMR (400 MHz, DMSO-*d*_6_) δ 7.45-7.40 (m, 3H), 7.34-7.30 (m, 2H), 7.26-7.22 (m, 1H), 6.95-6.94 (t, *J* =4 Hz, 1H), 6.86-6.85 (d, *J* =4 Hz, 1H), 6.58-6.51 (m, 1H), 6.36 (s, 1H), 5.26-5.20 (m, 2H). ^13^C NMR (100 MHz, DMSO-*d*_6_) δ 152.36, 146.29, 143.92, 127.74, 126.83, 126.35, 126.06, 125.05, 124.59, 112.84, 76.27.

**1-(4-fluorophenyl)-1-(thiophen-2-yl) prop-2-en-1-ol (2t)**

Light yellow oil, yield = 68%. ^1^H NMR (400 MHz, DMSO-*d*_6_) δ 7.46-7.40 (m, 3H), 7.16-7.11 (m, 2H), 6.97-6.95 (t, *J* =4 Hz, 1H), 6.86-6.85 (d, *J* =4 Hz, 1H), 6.57-6.50 (m, 1H), 6.43 (s, 1H), 5.24-5.20 (m, 2H). ^13^C NMR (100 MHz, DMSO-*d*_6_) δ 162.81, 160.39, 152.63, 144.24, 143.00 (d, *J* = 3.1 Hz), 128.66 (d, *J* = 8.1 Hz), 126.92, 125.69, 125.19, 114.91 (d, *J* = 21.2 Hz), 113.55, 76.47. ^19^F NMR (376 MHz, DMSO-*d*_6_) δ -116.18 (m, 1F).

**1-phenyl-1-(pyridin-3-yl) prop-2-en-1-ol (2u)**

Colorless oil, yield = 61%. ^1^H NMR (400 MHz, DMSO-*d*_6_) δ 8.56 (s, 1H), 8.44-8.43 (d, *J* =4 Hz, 1H), 7.72-7.69 (d, *J* =12 Hz, 1H), 7.39-7.35 (m, 5H), 7.34-7.31 (m, 1H), 6.65-6.58 (m, 1H), 6.26 (s, 1H), 5.29-5.21 (m, 1H). ^13^C NMR (100 MHz, DMSO-*d*_6_) δ 148.59, 148.19, 146.58, 144.07, 142.67, 134.72, 128.45, 127.35, 127.06, 123.42, 114.17, 77.25.

**1-cyclohexyl-1-phenylprop-2-en-1-ol (2v)**

Colorless oil, yield = 58%. ^1^H NMR (400 MHz, DMSO-*d*_6_) δ 7.46-7.44 (d, *J* =8 Hz, 2H), 7.33-7.29 (t, *J* =8 Hz, 2H), 7.20-7.17 (t, *J* =8 Hz, 2H), 6.33-6.26 (m, 1H), 5.33-5.27(d, *J* =24 Hz, 1H), 5.10-5.07 (d, *J* =12 Hz, 1H), 4.85 (s, 1H), 1.71-1.57 (m, 5H), 1.34-1.31 (d, *J* =12 Hz, 1H), 1.19-0.99 (m, 5H). ^13^C NMR (100 MHz, DMSO-*d*_6_) δ 146.68, 143.97, 127.49, 125.75, 125.60, 111.78, 78.04, 47.34, 26.71, 26.30, 26.24, 26.16.

**1-cyclopentyl-1-phenylprop-2-en-1-ol (2w)**

Colorless oil, yield = 59%. ^1^H NMR (400 MHz, DMSO-*d*_6_) δ 7.50-7.48 (d, *J* =8 Hz, 2H), 7.33-7.29 (m, 2H), 7.20-7.17 (m, 1H), 6.27-6.20 (m, 1H), 5.29-4.93 (m, 1H), 2.52-2.45 (m, 1H), 1.55-1.46 (m, 5H), 1.45-1.34 (m, 2H), 1.32-1.17 (m,1H). ^13^C NMR (100 MHz, DMSO-*d*_6_) δ 147.75, 145.25, 128.04, 126.28, 126.02, 111.81, 77.62, 49.20, 27.15, 27.08, 26.35, 26.18.

**1,1-bis(4-chlorophenyl)-2-methylprop-2-en-1-ol (2x)**

Colorless oil, yield = 47%.。^1^H NMR (400 MHz, DMSO-*d*_6_) δ 7.36-7.34 (d, *J* =12 Hz, 4H), 7.28-7.26 (d, *J* =12 Hz, 4H), 6.19 (s, 1H), 5.05 (s, 1H), 4.45 (s, 1H), 1.68 (s, 3H). ^13^C NMR (100 MHz, DMSO-*d*_6_) δ 149.91, 144.83, 132.02, 129.72, 128.08, 115.56, 81.29, 20.25.

**2.2 Reactor Configuration and Cold-model Experiment**

To overcome the clogging issue observed in high solid-content systems, we scaled up our previously developed micro-scale photoreactor, Photo-*μ*COBR, which had demonstrated excellent performance in enhancing gas–liquid mixing and mass transfer, as well as in handling gas-liquid-solid three-phase systems containing small amounts of solid particles. However, as the solid concentration increased, the risk of channel blockage became significant.

To address this limitation, the channel dimensions were moderately enlarged, and the constriction geometry was optimized. The original sharp contraction–expansion structure was replaced by a smooth, streamlined transition featuring a gradual contraction and expansion profile. The wall curvature was fitted using a quadratic function to ensure continuous curvature and minimize local shear stress gradients. This design modification effectively reduces particle stagnation at the constriction regions under high solid-loading conditions.

The resulting *milli*-COBR has a channel diameter (D) of 2.5 mm, a constriction spacing (L) of 3.75 mm, and a contraction ratio (*α*) of 16%. The channel model was constructed using SolidWorks 2018, and a schematic representation of the reactor structure is shown in Figure S4.


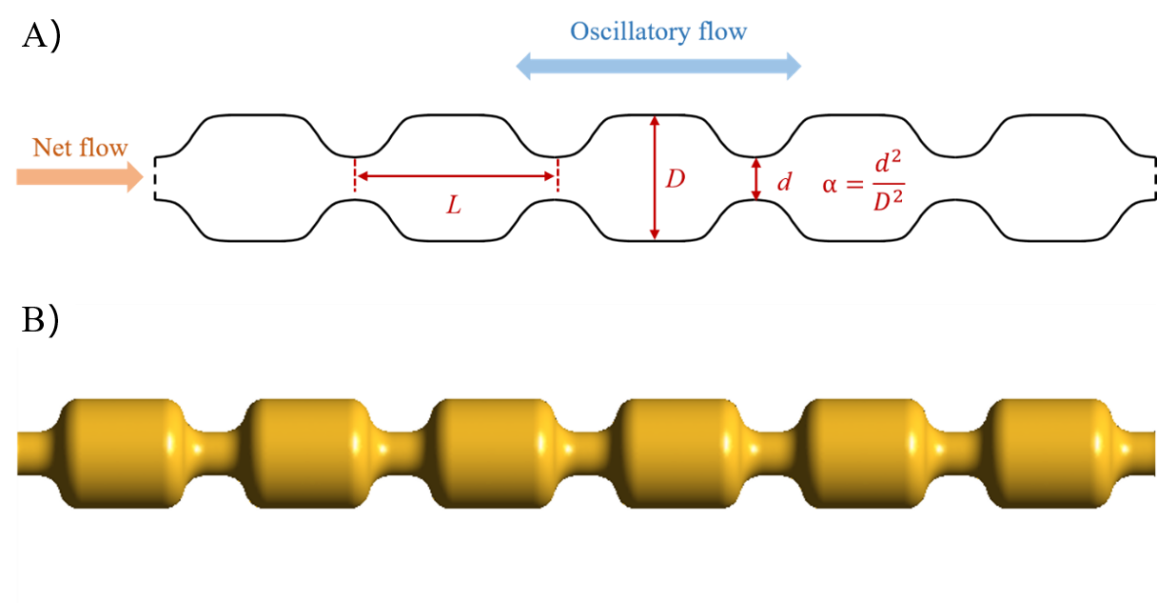


Figure S4. (A) Schematic representation of the *milli*-COBR channel geometry; (B) 3D model of the *milli*-COBR channel.

A glass-based photoreactor *milli*-COBR (1.8 mL) was fabricated via femtosecond laser internal engraving, and the actual reactor photograph is displayed in Figure S5.


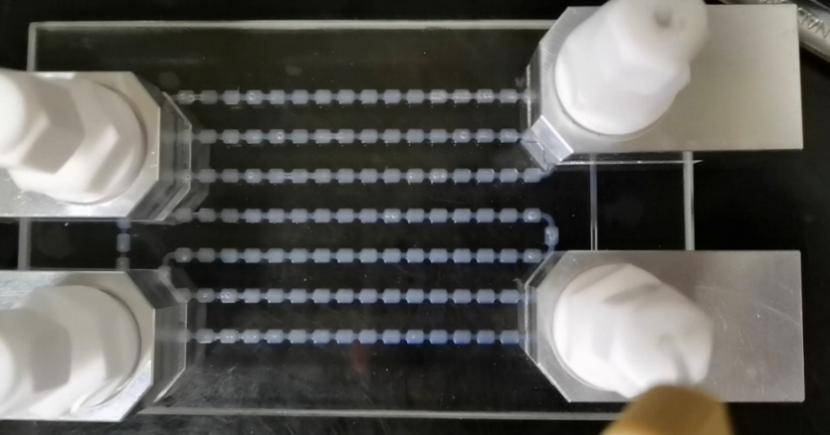


Figure S5. Photograph of the glass *milli*-COBR reactor fabricated by femtosecond laser engraving.

To evaluate the capability of the *milli*-COBR for continuous processing of solid-containing systems, a cold-model experiment was carried out. In a typical procedure, anhydrous lithium chloride powder (5 g, 118 mmol) was ground in a mortar and dispersed in diethyl ether (95 g, 1281 mmol) to form a 5 wt% suspension. The suspension was transferred to a 25 mL glass syringe equipped with a magnetic stir bar (85-1, Shanghai Sile Instrument Co., Ltd.) and pumped vertically at a flow rate of 0.5 mL·min⁻¹ using a syringe pump (TYD02-01, Baoding Lead Fluid Technology Co., Ltd.). Continuous stirring was maintained to prevent solid sedimentation. Oscillatory flow was introduced at the reactor inlet through a T-shaped connection, with the oscillation frequency and amplitude controlled at 2 Hz and 10 mm, respectively. PTFE capillaries (I.D. 1.6 mm, O.D. 3.2 mm) were used throughout the setup. After steady operation, the suspension state inside the reactor and the solid flow at the outlet were visually inspected.

To further verify the reactor’s anti-clogging performance, suspensions of AlCl_3_ in diethyl ether (solid particle size < 0.5 mm) with varying solid loadings (1–5 wt%) were processed under identical oscillatory conditions. As shown in Figure S6, the solid particles remained well suspended throughout the reactor, and no clogging or sedimentation was observed even after 40 min of continuous operation at 5 wt% solid loading. The outlet stream exhibited negligible loss of solid content, confirming the excellent potential of the *milli*-COBR for continuous handling of heterogeneous, solid-containing reaction systems.


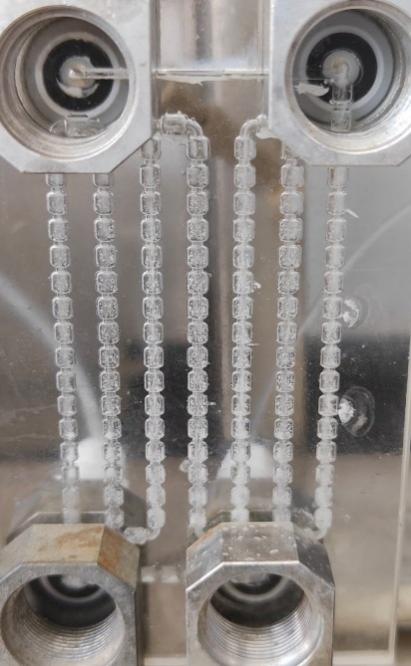


Figure S6. Photograph showing the suspension behavior during the cold-model experiment in the *milli*-COBR.

Reagents and Materials: All chemical reagents were purchased from Shanghai Titan Scientific Co., Ltd., and no further purification was performed. Glassware was obtained from Beijing Xinweier Glass Instrument Co., Ltd. And all accessories related to the continuous-flow experiments were purchased from Nanjing Runze Fluid Control Equipment Co., Ltd.

**2.3 Continuous-Flow Synthesis of Compounds 3a–x**

The continuous-flow photochemical setup for the synthesis of compounds **3a–x** is shown in Figures S7. A typical procedure is described as follows:

Compound **2a** (0.21 g, 1.0 mmol) and *mpg*-C_3_N_4_ (0.042 g) were weighed into a 50 mL two-necked round-bottom flask (Flask A). Under anhydrous and nitrogen atmosphere, anhydrous acetonitrile (25 mL) was added, and the resulting suspension was transferred into a 25 mL glass syringe (Syringe A) equipped with a magnetic stir bar. In a separate 50 mL two-necked flask (Flask B), trifluoromethanesulfonyl chloride (TfCl) (0.141 mL, 1.5 mmol), *N, N, N′, N′*-tetramethylethylenediamine (TMEDA) (0.35 mL, 3.0 mmol), and anhydrous acetonitrile (50 mL) were mixed thoroughly and transferred to another glass syringe (Syringe B). Both solutions were delivered into the *milli*-COBR reactor using syringe pumps (TYD02-01, Baoding Lead Fluid Technology Co., Ltd.) at a flow rate of 0.03 mL·min⁻¹ each. Pump A for the substrate suspension was positioned vertically above a magnetic stirrer (85-1, Shanghai Siluo Instrument Co., Ltd.) to maintain uniform suspension of *mpg*-C_3_N_4_. The reactor was irradiated with a 40 W blue LED panel (450–460 nm, Shenzhen Facai Optoelectronics Technology Co., Ltd.) as the light source, with a cooling fan employed to dissipate heat. An oscillatory flow field was introduced at the reactor inlet through a T-shaped connector, driven by a piston pump (MP2500-2L (PEEK), Baoding Dirui Electronic Technology Co., Ltd.). The oscillation frequency and amplitude were set to 2 Hz and 10 mm, respectively, and controlled via computer interface. A back-pressure regulator (BPR10, Zaiput Flow Technologies) was installed at the reactor outlet and set to 25 psi to maintain a strong oscillatory intensity. Unless otherwise stated, PTFE capillaries (I.D. 1.0 mm, O.D. 1.6 mm) were used throughout the continuous-flow setup.

After steady-state operation, the reactor effluent was continuously collected for 350 min (22 mL). Solvent removal under reduced pressure afforded the crude product, which was purified by silica gel column chromatography (300–400 mesh) to give **3a** as a colorless oil (0.073 g, 83% yield). Using the same procedure, a series of target compounds (**3b–x**) were synthesized.

For the optimization of continuous-flow conditions, reaction yields were determined by HPLC analysis to enable rapid and reliable evaluation. The HPLC yields were quantified based on an external calibration curve. Specifically, a series of standard solutions of the target product with known concentrations (0.002-0.01 M) were prepared and analyzed by HPLC to construct the calibration plot. The yields of the reaction samples were then calculated according to this calibration curve. The corresponding calibration plot and representative data are provided in Figure S7.


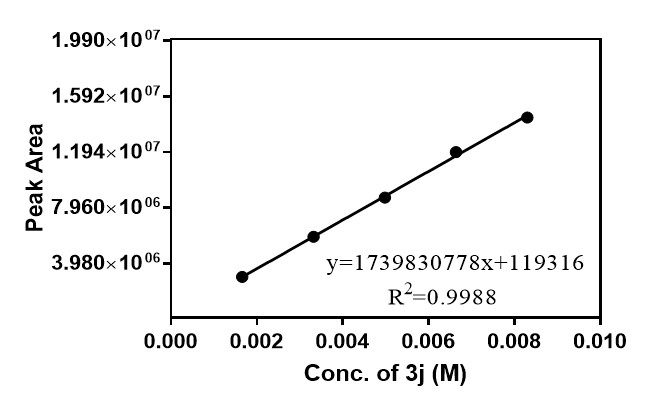


Figure S7. HPLC calibration curve of compound **3j**.

During the substrate scope investigation, the formation of regioisomeric products was observed for certain substrates (**2i**, **2j**, **2l**, **2m**, **2o**, **2p**, and **2u**), generally with moderate to good yields and pronounced regioselectivity. In most cases, the resulting isomers were successfully separated by column chromatography, and their individual yields were determined. The corresponding isomer ratios were calculated based on the isolated yields of each isomer. Notably, for substrates **2m** and **2p** bearing para-fluoro substituents, inseparable mixtures of constitutional isomers were obtained, with ratios of 2.7:1 and 2.2:1, respectively. In these cases, the isomer ratios were determined based on NMR analysis (Figures S128 and S143).

**
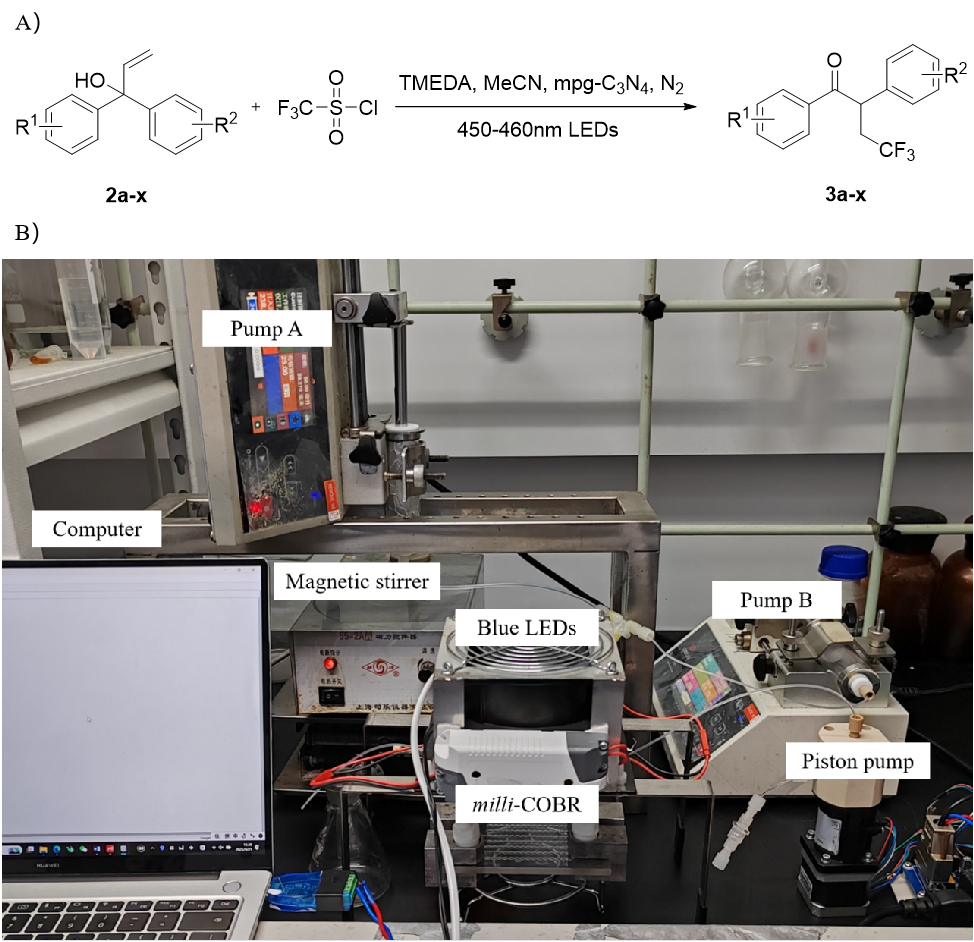
**

Figure S8. (A) Synthetic route for compounds **3a–x**. (B) The continuous-flow photochemical setup for the synthesis of compounds 3a–x.


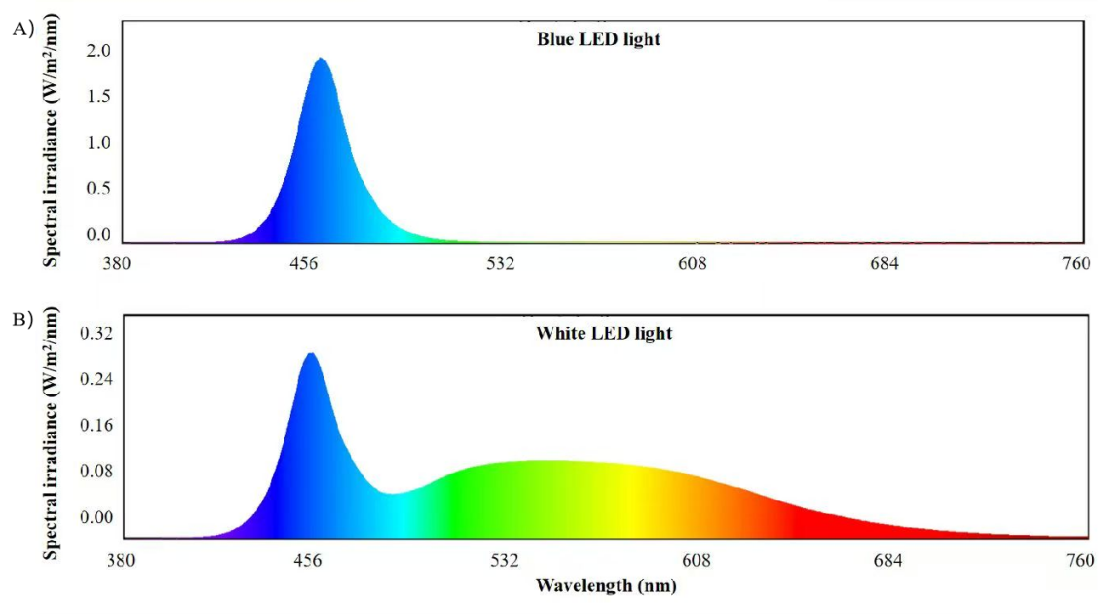


Figure S9. Emission spectrum of (A) blue LED light source and (B) incandescent lamp.

Reagents and Materials: All chemical reagents, except for substrates **2a–x**, were purchased from Shanghai Titan Scientific Co., Ltd., and no further purification was performed. Glassware was obtained from Beijing Xinweier Glass Instrument Co., Ltd. And all accessories related to the continuous-flow experiments were purchased from Nanjing Runze Fluid Control Equipment Co., Ltd.

Characterization: ^1^H, ^13^C, and ^19^F NMR spectra were recorded on a Bruker AVANCE NEO spectrometer. High-resolution mass spectra (HRMS) were acquired using either an EI-GCT Premier or ESI-Xevo G2 TOF mass spectrometer (Waters Technology (Shanghai) Co., Ltd.). Detailed structures and NMR data for compounds **3a–x** are provided below:

**4,4,4-trifluoro-1,2-diphenylbutan-1-one (3a)**

Colorless oil, yield = 83%. ^1^H NMR (400 MHz, CDCl_3_) δ 7.88-7.86 (m, 2H), 7.42-7.37 (m, 1H), 7.31-7.27 (m, 2H), 7.23-7.18 (m, 4H), 7.15-7.12 (m, 1H), 4.84-4.81 (m, 1H), 3.29-3.15 (m, 1H), 2.52-2.39 (m, 1H). ^13^C NMR (100 MHz, CDCl_3_) 194.89, 135.54, 133.80, 131.54, 127.49, 126.97, 126.83, 126.34, 126.19, 125.70 (q, *J* = 277.5 Hz), 45.30 (q, *J* = 2.7 Hz), 35.48 (q, *J* = 28.2 Hz). ^19^F NMR (376 MHz, DMSO-*d*_6_) δ -64.55 (t, *J* = 11 Hz, 3F). HRMS (EI) m/z calculated for C_16_H_13_F_3_O [M]: 278.0918, found: 278.0920.

**4,4,4-trifluoro-1,2-di-p-tolylbutan-1-one (3b)**

Colorless oil, yield = 76%. ^1^H NMR (400 MHz, CDCl_3_) δ 7.82-7.80 (d, *J* = 8 Hz, 2H), 7.15-7.12 (d, *J* = 12 Hz, 4H), 7.06-7.04 (d, *J* = 8 Hz, 2H), 4.82-4.78 (t, *J* = 8Hz, 1H), 3.27-3.18 (m, 1H), 2.50-2.49 (m, 1H), 2.30 (s, 2H), 2.22 (s, 2H). ^13^C NMR (100 MHz, CDCl_3_) δ 195.39, 143.18, 136.47, 133.62, 132.14, 128.94, 128.31, 127.93, 126.84 (q, *J* = 277.5 Hz), 45.60 (q, *J* = 2.1 Hz), 36.30 (q, *J* = 28.3 Hz), 20.56, 19.97. ^19^F NMR (376 MHz, CDCl_3_) δ -64.55 (t, *J* = 11 Hz, 3F). HRMS (EI) m/z calculated for C_18_H_17_F_3_O [M]: 306.1231, found: 306.1230.

**1,2-bis(3-chlorophenyl)-4,4,4-trifluorobutan-1-one (3c)**

Colorless oil, yield = 70%. ^1^H NMR (400 MHz, CDCl_3_) δ 7.92 (s, 1H), 7.81-7.79 (d, *J* = 8 Hz, 1H), 7.51-7.49 (d, *J* = 8 Hz, 1H), 7.38-7.34 (t, *J* = 8 Hz, 1H), 7.29-7.23 (m, 3H), 7.23-7.17 (m, 1H), 4.83-4.80 (t, *J* = 8 Hz, 1H), 3.33-3.20 (m, 1H), 2.60-2.47 (m, 1H). ^13^C NMR (100 MHz, CDCl_3_) δ 195.04, 138.70, 136.94, 135.31, 135.26, 133.61, 130.74, 130.11, 128.88,128.44, 128.06, 127.47, 126.81, 126.28 (q, *J* = 277.5 Hz), 46.90 (q, *J* = 2.1 Hz), 37.28 (q, *J* = 28.3 Hz). ^19^F NMR (376 MHz, CDCl_3_) δ -64.57 (t, *J* = 11 Hz, 3F). HRMS (EI) m/z calculated for C_16_H_11_Cl_2_F_3_O [M]: 346.0319, found: 346.0315.

**1,2-bis(4-bromophenyl)-4,4,4-trifluorobutan-1-one (3d)**

Colorless oil, yield = 81%. ^1^H NMR (400 MHz, CDCl_3_) δ 7.79-7.77 (d, *J* = 8 Hz, 2H), 7.56-7.53 (d, *J* = 12 Hz, 2H), 7.45-7.43 (d, *J* = 8 Hz, 2H), 7.16-7.14 (d, *J* = 8 Hz, 2H), 4.81-4.78 (t, *J* = 12 Hz, 1H), 3.28-3.19 (m, 1H), 2.56-2.48 (m, 1H). ^13^C NMR (100 MHz, CDCl_3_) δ 195.37, 136.00, 134.06, 131.20 (q, *J* = 277.5 Hz), 128.97, 122.26, 46.66 (q, *J* = 2.1 Hz), 37.14 (q, *J* = 28.3 Hz). ^19^F NMR (376 MHz, CDCl_3_) δ -64.41 (t, *J* = 11 Hz, 3F). HRMS (EI) m/z calculated for C_16_H_11_Br_2_F_3_O [M]: 433.9129, found: 433.9124.

**1,2-bis(4-chlorophenyl)-4,4,4-trifluorobutan-1-one (3e)**

Colorless oil, yield = 83%. ^1^H NMR (400 MHz, CDCl_3_) δ 7.91-7.89 (d, *J* = 8 Hz, 2H), 7.42-7.40 (d, *J* = 8 Hz, 2H), 7.34-7.32 (d, *J* = 8 Hz, 2H), 7.26-7.25 (d, *J* = 4 Hz, 2H), 4.88-4.84 (t, *J* = 8 Hz, 1H), 3.34-3.21 (m, 1H), 2.63-2.50 (m, 1H). ^13^C NMR (100 MHz, CDCl_3_) δ 195.25, 140.19, 135.53, 134.15, 133.70, 130.36, 130.18, 129.69, 129.36, 126.23 (q, *J* = 277.5 Hz), 46.61 (q, *J* = 2.1 Hz), 37.23 (q, *J* = 28.3 Hz). ^19^F NMR (376MHz, CDCl_3_) δ -64.41 (t, *J* = 11 Hz, 3F). HRMS (EI) m/z calculated for C_16_H_11_Cl_2_F_3_O [M]: 346.0139, found: 346.0141.

**4,4,4-trifluoro-1,2-bis(4-fluorophenyl) butan-1-one (3f)**

Colorless oil, yield = 69%. ^1^H NMR (400 MHz, CDCl_3_) δ 8.02-7.98 (m, 2H), 7.31-7.28 (m, 2H), 7.14-7.09 (m, 2H), 7.06-7.02 (m, 2H), 4.89-4.86 (t, *J* = 8 Hz, 1H), 3.34-3.21 (m, 1H), 2.63-2.50 (m, 1H). ^13^C NMR (100 MHz, CDCl_3_) δ 195.11, 165.90 (d, *J* = 257.6 Hz), 162.35 (d, *J* = 248.5 Hz), 132.99 (d, *J* = 3.1 Hz), 131.88 (d, *J* = 3.1 Hz), 131.51 (d, *J* = 11.1 Hz), 129.66 (d, *J* = 8.1 Hz), 126.28 (q, *J* = 277.5 Hz), 116.45 (d, *J* = 21.2 Hz), 115.97 (d, *J* = 22.3 Hz), 46.40 (q, *J* = 2.1 Hz), 37.42 (q, *J* = 28.3 Hz). ^19^F NMR (376 MHz, CDCl_3_) δ -64.51 (m, 3F), -104.03 (m, 1F), -113.83 (m, 1F). HRMS (EI) m/z calculated for C_16_H_11_F_5_O [M]: 314.0730, found: 314.0727.

**4,4,4-trifluoro-1,2-bis(3-(trifluoromethyl) phenyl) butan-1-one (3g)**

Colorless oil, yield = 86%. ^1^H NMR (400 MHz, CDCl_3_) δ 8.21 (s, 1H), 8.12-8.10 (d, *J* = 8 Hz, 1H), 7.80-7.79 (d, *J* = 4 Hz, 1H), 7.60-7.45 (m, 5H), 4.98-4.95 (t, *J* = 8 Hz, 1H), 3.38-3.25 (m, 1H), 2.67-2.54 (m, 1H). ^13^C NMR (100 MHz, CDCl_3_) δ 194.93, 137.64, 135.75, 132.18 (q, *J* = 32.8 Hz), 131.85, 131.62 (q, *J* = 32.8 Hz), 131.38, 130.20 (q, *J* = 3.1 Hz), 130.12, 129.59, 126.56 (q, *J* = 277.5 Hz), 125.63 (q, *J* = 3.1 Hz), 125.21 (q, *J* = 3.1 Hz), 124.79 (q, *J* = 2.1 Hz), 122.25 (q, *J* = 272.5 Hz), 122.07 (q, *J* = 272.5 Hz), 47.10 (q, *J* = 2.1 Hz), 37.32 (q, *J* = 28.7 Hz). ^19^F NMR (376 MHz, CDCl_3_) δ -62.84 (s, 3F), -63.07 (s, 3F), -64.53 (t, *J* = 11 Hz, 3F). HRMS (EI) m/z calculated for C_18_H_11_F_9_O [M-F+H]: 396.0716, found: 396.0717.

**4,4,4-trifluoro-1,2-bis(4-methoxyphenyl) butan-1-one (3h)**

Colorless oil, yield = 72%. ^1^H NMR (400 MHz, CDCl_3_) δ 7.96-7.94 (d, *J* = 8 Hz, 2H), 7.22-7.20 (d, *J* = 8 Hz, 2H), 6.89-6.82 (m, 4H), 4.83-4.79 (t, *J* = 8 Hz, 1H), 3.82 (s, 3H), 3.75 (s, 3H), 3.28-3.20 (m, 1H), 2.58-2.44 (m, 1H). ^13^C NMR (100 MHz, CDCl_3_) δ 194.36, 162.58, 158.01, 130.13, 128.79, 128.02, 127.56 (q, *J* = 277.5 Hz), 113.62, 112.82, 54.30 (q, *J* = 25.1 Hz), 44.93, 44.90, 36.36 (q, *J* = 27.9 Hz). ^19^F NMR (376 MHz, CDCl_3_) δ -64.49 (t, *J* = 11 Hz, 3F). HRMS (EI) m/z calculated for C_18_H_17_F_3_O_3_ [M]: 338.1130, found: 338.1128.

**1-(3,4-dimethylphenyl)-4,4,4-trifluoro-2-phenylbutan-1-one (3i)**

**2-(3,4-dimethylphenyl)-4,4,4-trifluoro-1-phenylbutan-1-one (3i’)**

Colorless oil, yield = 82% (2.5: 1). **3i-1:** ^1^H NMR (400 MHz, CDCl_3_) δ 7.79 (s, 1H), 7.75-7.73 (d, *J* = 8 Hz, 1H), 7.35-7.34 (d, *J* = 4 Hz, 4H), 7.30-7.26 (m, 1H), 7.20-7.18 (d, *J* = 8 Hz, 1H), 4.94-4.92 (t, *J* = 4 Hz, 1H), 3.39-3.27 (m, 1H), 2.64-2.59 (m, 1H), 2.31 (s, 6H). ^13^C NMR (100 MHz, CDCl_3_) δ 196.57, 143.12, 137.79, 137.12, 133.53, 129.86, 127.85, 127.71, 126.59, 125.09 (q, *J* = 277.5 Hz), 46.90, 37.37 (q, *J* = 27.9 Hz), 20.00, 19.80. ^19^F NMR (376 MHz, CDCl_3_) δ -64.60 (t, *J* = 11 Hz, 3F). HRMS (EI) m/z calculated for C_18_H_17_F_3_O [M]: 306.1231, found: 306.1229. **3i-2:** ^1^H NMR (400 MHz, CDCl_3_) δ 7.98-7.96 (d, *J* = 8 Hz, 2H), 7.52-7.48 (t, *J* = 8 Hz, 1H), 7.42-7.38 (t, *J* = 8 Hz, 2H), 7.08-7.02 (m, 3H), 4.85-4.83 (t, *J* = 4 Hz, 1H), 3.37-3.24 (m, 1H), 2.57-2.44 (m, 1H), 2.21 (s, 3H), 2.19 (s, 3H). ^13^C NMR (100 MHz, CDCl_3_) δ 196.58, 143.11, 137.83, 137.13, 133.59, 130.00, 129.87, 129.27, 128.03, 127.87, 127.71, 126.59, 125.12 (q, *J* = 277.5 Hz), 46.96, 37.40 (q, *J* = 27.9 Hz), 19.98, 19.78. ^19^F NMR (376 MHz, CDCl_3_) δ -64.67 (t, *J* = 11 Hz, 3F). HRMS (EI) m/z calculated for C_18_H_17_F_3_O [M]: 306.1231, found: 306.1229.

**4,4,4-trifluoro-1-(4-methoxyphenyl)-2-phenylbutan-1-one (3j)**

**4,4,4-trifluoro-2-(4-methoxyphenyl)-1-phenylbutan-1-one (3j’)**

Colorless oil, yield = 66% (4.8: 1). **3j:** ^1^H NMR (400 MHz, CDCl_3_) δ 7.89-7.86 (m, 2H), 7.24-7.22 (m, 4H), 7.22-7.14 (m, 1H), 6.81-6.77 (m, 2H), 4.80-4.76 (t, *J* = 8 Hz, 1H), 3.73 (s, 3H), 3.25-3.16 (m, 1H), 2.49-2.41 (m, 1H). ^13^C NMR (100 MHz, CDCl_3_) δ 194.16, 170.15, 162.66, 136.89, 130.16, 128.24, 127.54, 126.78 (q, *J* = 277.5 Hz), 112.85, 54.42, 45.77 (q, *J* = 2.1 Hz), 36.34 (q, *J* = 27.9 Hz). ^19^F NMR (376 MHz, CDCl_3_) δ -64.57 (t, *J* = 11 Hz, 3F). HRMS (EI) m/z calculated for C_17_H_15_F_3_O_2_ [M]: 308.1024, found: 308.1022. **3j’:** ^1^H NMR (400 MHz, CDCl_3_) δ 7.96-7.94 (m, 2H), 7.52-7.48 (m, 1H), 7.42-7.38 (m, 2H), 7.23-7.19 (m, 2H), 6.85-6.82 (m, 2H), 4.88-4.84 (t, *J* = 8 Hz, 1H), 3.74 (s, 3H), 3.30-3.19 (m, 1H), 2.62-2.45 (m, 1H). ^13^C NMR (100 MHz, CDCl_3_) δ 196.93, 171.21, 159.13, 135.71, 133.30, 131.22, 129.27, 128.66, 128.11, 126.46 (q, *J* = 277.5 Hz), 114.72, 55.22, 46.33 (q, *J* = 2.1 Hz), 37.37 (q, *J* = 27.9 Hz). ^19^F NMR (376 MHz, CDCl_3_) δ -64.52 (t, *J* = 11 Hz, 3F). HRMS (EI) m/z calculated for C_17_H_15_F_3_O_2_ [M]: 308.1024, found: 308.1022.

**1-([1,1'-biphenyl]-4-yl)-4,4,4-trifluoro-2-phenylbutan-1-one (3k)**

Colorless oil, yield = 63%. ^1^H NMR (400 MHz, CDCl_3_) δ 7.89-7.87 (d, *J* = 8 Hz, 2H), 7.43-7.38 (m, 5H), 7.32-7.25 (m, 6H), 7.23-7.21 (d, *J* = 8 Hz, 1H), 4.87-4.83 (t, *J* = 8 Hz, 1H), 3.31-3.17 (m, 1H), 2.54-2.41 (m, 1H). ^13^C NMR (100 MHz, CDCl_3_) δ 196.73, 140.81, 140.26, 136.38, 135.77, 133.45, 129.47, 129.40, 128.98, 128.89, 128.83, 128.75, 128.49, 128.35, 128.04, 127.90, 127.83, 127.55, 127.35, 127.27, 127.05 (q, *J* = 277.5 Hz), 46.85 (q, *J* = 2.1 Hz), 37.43 (q, *J* = 27.9 Hz). ^19^F NMR (376 MHz, CDCl_3_) δ -64.52 (t, *J* = 11 Hz, 3F). HRMS (EI) m/z calculated for C_22_H_17_F_3_O [M]: 354.1231, found: 354.1230.

**2-(4-chlorophenyl)-4,4,4-trifluoro-1-phenylbutan-1-one (3l)**

**1-(4-chlorophenyl)-4,4,4-trifluoro-2-phenylbutan-1-one (3l’)**

Colorless oil, yield = 88% (3.8: 1). **3l-1:** ^1^H NMR (400 MHz, CDCl_3_) δ 7.94-7.92 (d, *J* = 8 Hz, 2H), 7.54-7.50 (t, *J* = 8 Hz, 1H), 7.43-.39 (t, *J* = 8 Hz, 2H), 7.29-7.23 (m, 4H), 4.91-4.87 (t, *J* = 8 Hz, 1H), 3.29-3.21 (m, 1H), 2.58-2.50 (m, 1H). ^13^C NMR (100 MHz, CDCl_3_) δ 196.46, 135.83, 135.41, 133.93, 133.61, 129.56, 129.42, 128.79, 126.29 (q, *J* = 27.9 Hz), 46.49 (q, *J* = 2.1 Hz), 37.28 (q, *J* = 27.9 Hz). ^19^F NMR (376 MHz, CDCl_3_) δ -64.47 (t, *J* = 11 Hz, 3F). HRMS (EI) m/z calculated for C_16_H_12_ClF_3_O [M]: 312.0529, found: 312.0526. **3l-2:** ^1^H NMR (400 MHz, CDCl_3_) δ 7.93 (s, 1H), 7.91 (s, 1H), 7.42-7.40 (d, *J* = 8 Hz, 2H), 7.37-7.27 (m, 5H), 4.88-4.85 (t, *J* = 8 Hz, 1H), 3.40-3.26 (m, 1H), 2.63-2.50 (m, 1H). ^13^C NMR (100 MHz, CDCl_3_) δ 195.53, 139.91, 137.10, 133.96, 130.23, 129.47, 129.03, 128.03, 126.46 (q, *J* = 277.5 Hz), 47.32 (q, *J* = 2.1 Hz), 37.43 (q, *J* = 27.9 Hz). ^19^F NMR (376 MHz, CDCl_3_) δ -64.61 (t, *J* = 11 Hz, 3F). HRMS (EI) m/z calculated for C_16_H_12_ClF_3_O [M]: 312.0529, found: 312.0526.

**4,4,4-trifluoro-2-(4-fluorophenyl)-1-phenylbutan-1-one (3m)**

**4,4,4-trifluoro-1-(4-fluorophenyl)-2-phenylbutan-1-one (3m’)**

Colorless oi, yield = 73% (2.7: 1). ^1^H NMR (400 MHz, CDCl_3_) δ 8.01-7.92 (m, 2H), 7.54-7.39 (m, 1H), 7.34-7.22 (m, 4H), 7.09-6.98 (m, 2H), 4.92-4.83 (m, 1H), 3.37-3.21 (m, 1H), 2.61-2.47 (m, 1H). ^13^C NMR (100 MHz, CDCl_3_) δ 196.70, 195.17, 163.51, 137.28, 135.49, 133.53, 133.09, 132.04, 131.57, 131.48, 129.76, 129.68, 129.44, 128.80, 128.76, 127.98, 127.73, 124.98, 116.44, 116.23, 115.97, 115.75, 47.26, 46.34, 37.77, 37.55, 37.49, 37.21, 36.93. ^19^F NMR (376 MHz, CDCl_3_) δ -64.48 (t, *J* = 11 Hz, 1F), -64.60 (t, *J* = 11 Hz, 3F). HRMS (EI) m/z calculated for C_16_H_12_F_4_O [M]: 296.0824, found: 296.0821.

**4,4,4-trifluoro-1-phenyl-2-(4-(trifluoromethyl) phenyl) butan-1-one (3n)**

Colorless oil, yield = 88%. ^1^H NMR (400 MHz, CDCl_3_) δ 7.96-7.94 (d, *J* = 8 Hz, 2H), 7.59-7.52 (m, 3H), 7.46-7.41 (m, 4H), 5.01-4.98 (t, *J* = 8 Hz, 1H), 3.36-3.23 (m, 1H), 2.65-2.52 (m, 1H). ^13^C NMR (100 MHz, CDCl_3_) δ 196.18, 141.30, 135.30, 133.79, 130.39, 130.20 (d, *J* = 31.5 Hz), 129.11, 128.87, 128.80, 128.51, 126.98 (d, *J* = 4.3 Hz), 126.27 (q, *J* = 277.5 Hz), 123.66 (q, *J* = 277.5 Hz), 46.86 (q, *J* = 2.1 Hz), 37.28 (q, *J* = 27.9 Hz). ^19^F NMR (376 MHz, CDCl_3_) δ -62.77 (s, 1F), -64.69 (t, *J* = 11 Hz, 3F). HRMS (EI) m/z calculated for C_17_H_12_F_6_O [M]: 346.0792, found: 346.0797.

**2-(2,5-dichlorophenyl)-4,4,4-trifluoro-1-phenylbutan-1-one (3o)**

**1-(2,5-dichlorophenyl)-4,4,4-trifluoro-2-phenylbutan-1-one (3o’)**

Colorless oil, yield = 81% (4.1: 1). **3o-1:** ^1^H NMR (400 MHz, CDCl_3_) δ 7.32-7.24 (m, 5H), 7.17-7.15 (d, *J* =8 Hz, 2H), 7.08 (s, 1H), 4.77-4.74 (t, *J* =8 Hz, 1H), 3.34-3.26 (m, 1H), 2.65-2.52 (m, 1H). ^13^C NMR (100 MHz, CDCl_3_) δ 198.11, 139.35, 135.18, 132.87, 131.71, 131.62, 129.36, 129.12, 128.90, 128.47, 128.39, 126.28 (q, *J* = 277.5 Hz), 51.43 (q, *J* = 2.1 Hz), 35.94 (q, *J* = 27.9 Hz). ^19^F NMR (376 MHz, CDCl_3_) δ -69.06 (t, *J* = 11 Hz, 3F). HRMS (EI) m/z calculated for C_16_H_11_Cl_2_F_3_O [M]: 346.0139, found: 346.0143. **3o-2:** ^1^H NMR (400 MHz, CDCl_3_) δ 7.97-7.96 (d, *J* =4 Hz, 2H), 7.57-7.53 (t, *J* =8 Hz, 1H), 7.45-7.42 (t, *J* =8 Hz, 2H), 7.38-7.36 (d, *J* = 4 Hz, 1H), 7.21-7.16 (m, 2H), 5.42-5.39 (t, *J* =4 Hz, 1H), 3.32-3.18 (m, 1H), 2.52-2.39 (m, 1H). ^13^C NMR (100 MHz, CDCl_3_) δ 196.01, 136.81, 135.12, 133.93, 133.67, 131.64, 131.46, 129.47, 128.89, 128.72, 125.98 (q, *J* = 277.5 Hz), 42.93 (q, *J* = 2.1 Hz), 36.57 (q, *J* = 27.9 Hz). ^19^F NMR (376 MHz, CDCl_3_) δ -69.06 (t, *J* = 11 Hz, 3F). HRMS (EI) m/z calculated for C_16_H_11_Cl_2_F_3_O [M]: 346.0139, found: 346.0143.

**4,4,4-trifluoro-1-(2-fluorophenyl)-2-(4-fluorophenyl) butan-1-one (3p)**

**4,4,4-trifluoro-2-(2-fluorophenyl)-1-(4-fluorophenyl) butan-1-one (3p’)**

Colorless oil, yield = 88% (2.2: 1). ^1^H NMR (400 MHz, CDCl_3_) δ 8.02-7.44 (m, 2H), 7.25-6.94 (m, 6H), 5.25-4.85 (m, 1H), 3.34-3.18 (m, 1H), 2.62-2.43 (m, 1H). ^13^C NMR (100 MHz, CDCl_3_) δ 195.60, 195.56, 194.67, 167.26, 164.72, 163.59, 162.32, 161.13, 160.85, 159.79, 158.40, 135.06, 134.97, 132.20, 132.17, 131.59, 131.56, 131.40, 131.36, 131.33, 131.31, 130.13, 130.05, 129.91, 129.83, 128.67, 128.64, 127.66, 125.09, 125.05, 124.91, 124.88, 124.68, 124.65, 124.55, 124.46, 124.31, 116.83, 116.60, 116.25, 116.15, 116.07, 116.03, 115.94, 115.86, 50.29, 50.26, 50.21, 50.19, 38.84, 38.81, 38.78, 37.50, 37.21, 36.93, 36.65, 36.59, 36.31. ^19^F NMR (376 MHz, CDCl_3_) δ -64.48 (t, *J* = 8 Hz, 2F), -64.92 (t, *J* = 11 Hz, 3F). HRMS (EI) m/z calculated for C_16_H_11_F_5_O [M]: 314.0730, found: 314.0732.

**1-(2-chlorophenyl)-2-(4-chlorophenyl)-4,4,4-trifluorobutan-1-one (3q)**

Colorless oil, yield = 87%. ^1^H NMR (400 MHz, CDCl_3_) δ 7.40-7.30 (m, 2H), 7.30-7.23 (m, 2H), 7.23-7.20 (m, 2H), 7.19-7.14 (m, 2H), 4.85-4.81 (t, *J* = 8 Hz, 1H), 3.35-3.22 (m, 1H), 2.68-2.55 (m, 1H). ^13^C NMR (100 MHz, CDCl_3_) δ 199.18, 171.16, 137.88, 134.18, 131.99, 130.86, 130.56, 129.79, 129.39, 129.24, 127.61, 125.82 (q, *J* = 277.5 Hz), 50.70 (q, *J* = 2.1 Hz), 36.00 (q, *J* = 27.9 Hz). ^19^F NMR (376 MHz, CDCl_3_) δ -64.19 (t, *J* = 11 Hz, 3F). HRMS (EI) m/z calculated for C_16_H_11_Cl_2_F_3_O [M]: 346.0139, found: 346.0135.

**1-(2,6-difluorophenyl)-4,4,4-trifluoro-2-phenylbutan-1-one (3r)**

Colorless oil, yield = 81%. ^1^H NMR (400 MHz, CDCl_3_) δ 7.31-7.21 (m, 4H), 7.21-7.16 (m, 1H), 6.84-6.80 (t, *J* = 8 Hz, 2H), 4.59-4.56 (t, *J* = 8 Hz, 1H), 3.39-3.26 (m, 1H), 2.59-2.46 (m, 1H). ^13^C NMR (100 MHz, *d*-DMSO) δ 13C NMR (100 MHz, CDCl_3_) δ 193.69, 160.89, 160.82, 158.37, 158.30, 135.42, 132.86, 132.76, 132.65, 130.43, 129.18, 128.39, 128.23, 126.31 (q, *J* = 277.5 Hz), 116.9 (t, *J* = 19.1 Hz)2, 111.95 (dd, *J* = 21.6, 4.5 Hz), 53.29 (q, *J* = 2.1 Hz), 35.67 (q, *J* = 27.9 Hz). ^19^F NMR (376 MHz, CDCl_3_) δ -64.52 (m, 3F), -112.05 (s, 1F), -112.07 (s, 1F). HRMS (EI) m/z calculated for C_16_H_11_F_5_O [M]: 314.0730, found: 314.0727.

**4,4,4-trifluoro-1-phenyl-2-(thiophen-2-yl) butan-1-one (3s)**

Light yellow oil, yield = 66%. ^1^H NMR (400 MHz, CDCl_3_) δ 7.93-7.91 (m, 2H), 7.49-7.45 (t, *J* = 8 Hz, 1H), 7.38-7.34 (t, *J* = 8 Hz, 2H), 7.18-7.11 (m, 1H), 6.87-6.82 (m, 2H), 5.15-5.12 (t, *J* = 8 Hz, 1H), 3.40-3.19 (m, 1H), 2.62-2.49 (m, 1H). ^13^C NMR (100 MHz, CDCl_3_) δ 194.54, 138.14, 134.25, 132.62, 127.99, 127.82, 127.75, 126.35 (q, *J* = 277.5 Hz), 53.81, 40.59 (q, *J* = 2.1 Hz), 36.98 (q, *J* = 27.9 Hz), 28.68. ^19^F NMR (376 MHz, CDCl_3_) δ -64.79 (t, *J* = 11 Hz, 3F). HRMS (EI) m/z calculated for C_14_H_11_F_3_OS [M]: 284.0483, found: 284.0480.

**4,4,4-trifluoro-1-(4-fluorophenyl)-2-(thiophen-2-yl) butan-1-one (3t)**

Light yellow oil, yield = 66%. ^1^H NMR (400 MHz, CDCl_3_) δ 8.06-8.01 (m, 2H), 7.27-7.26 (d, *J* = 4 Hz, 1H), 7.22-7.08 (m, 2H), 6.94-6.91 (m, 2H), 5.19-5.14 (t, *J* = 8 Hz, 1H), 3.40-3.26 (m, 1H), 2.69-2.56 (m, 1H). ^13^C NMR (100 MHz, CDCl_3_) δ 194.00, 167.28, 164.73, 138.97, 131.63, 131.55 (d, *J* = 3.1 Hz), 127.45, 127.36, 126.14 (q, *J* = 277.5 Hz), 116.15 (dd, *J* = 30.3, 22.2 Hz), 41.66 (q, *J* = 2.1 Hz), 37.98 (q, *J* = 27.9 Hz). ^19^F NMR (376 MHz, CDCl_3_) δ -55.56 (s, 1F), -64.80 (t, *J* = 11 Hz, 3F). HRMS (EI) m/z calculated for C_14_H_10_F_4_OS [M]: 302.0388, found: 302.0391.

**4,4,4-trifluoro-1-phenyl-2-(pyridin-3-yl) butan-1-one (3u)**

**4,4,4-trifluoro-2-phenyl-1-(pyridin-3-yl) butan-1-one (3u’)**

Colorless oil, yield = 54% (4.0: 1). **3u-1:** ^1^H NMR (400 MHz, CDCl_3_) δ 8.79 (s, 1H), 7.99-7.97 (d, *J* = 4 Hz, 2H), 7.88-7.85 (d, *J* = 12 Hz, 1H), 7.69-7.67 (d, *J* = 8 Hz, 1H), 7.64-7.60 (m, 1H), 7.52-7.48 (m, 2H), 7.41-7.39 (m, 1H), 5.11-5.08 (t, *J* = 8 Hz, 1H), 3.36-3.23 (m, 1H), 2.73-2.60 (m, 1H). ^13^C NMR (100 MHz, CDCl_3_) δ 195.55, 171.22, 149.98, 136.65, 136.28, 134.75, 134.29, 129.13, 128.80, 120.91 (q, *J* = 277.5 Hz), 44.07 (q, *J* = 2.1 Hz), 37.20 (q, *J* = 27.9 Hz), 29.72, 21.07. ^19^F NMR (376 MHz, CDCl_3_) δ -64.26 (t, *J* = 11 Hz, 3F). HRMS (EI) m/z calculated for C_15_H_12_F_3_NO [M]: 279.0871, found: 279.0869. **3u-2:** ^1^H NMR (400 MHz, CDCl_3_) δ 9.19 (s, 1H), 8.73-8.72 (d, *J* = 4 Hz, 1H), 8.24-8.22 (d, *J* = 8 Hz, 1H), 7.39-7.26 (m, 6H), 4.89-4.86 (t, *J* = 8 Hz, 1H), 3.39-3.30 (m, 1H), 2.63-2.54 (m, 1H). ^13^C NMR (100 MHz, CDCl_3_) δ 195.61, 171.11, 153.58, 150.11, 136.51, 136.12, 131.08, 129.59, 128.22, 128.07, 126.24 (q, *J* = 277.5 Hz), 123.65, 47.83 (q, *J* = 2.1 Hz), 37.09 (q, *J* = 27.9 Hz), 21.00. ^19^F NMR (376 MHz, CDCl_3_) δ -64.58 (t, *J* = 11 Hz, 3F). HRMS (EI) m/z calculated for C_15_H_12_F_3_NO [M]: 279.0871, found: 279.0869.

**1-cyclohexyl-4,4,4-trifluoro-2-phenylbutan-1-one (3v)**

Colorless oil, yield = 74%. ^1^H NMR (400 MHz, CDCl_3_) δ 7.35-7.26 (m, 3H), 7.21-7.19 (d, *J* = 8 Hz, 2H), 4.14-4.10 (t, *J* = 8 Hz, 1H), 3.21-3.08 (m, 1H), 2.42-2.26 (m, 1H), 1.94-1.90 (m, 1H), 1.78-1.77 (d, *J* = 4 Hz, 1H), 1.65-1.60 (m, 2H), 1.40-1.34 (m, 2H), 1.29-1.17 (m, 2H), 1.17-1.04 (m, 2H). ^13^C NMR (100 MHz, CDCl_3_) δ 209.95, 136.93, 129.22, 128.20, 127.91, 126.36 (q, *J* = 277.5 Hz), 50.24 (q, *J* = 2.1 Hz), 36.40 (q, *J* = 27.9 Hz), 29.21, 28.09, 25.85, 25.64, 25.10, 25.06. ^19^F NMR (376 MHz, CDCl_3_) δ -64.87 (t, *J* = 11 Hz, 3F). HRMS (EI) m/z calculated for C_16_H_19_F_3_O [M]: 284.1388, found: 284.1390.

**1-cyclopentyl-4,4,4-trifluoro-2-phenylbutan-1-one (3w)**

Colorless oil, yield = 72%. ^1^H NMR (400 MHz, CDCl_3_) δ 7.40-7.30 (m, 3H), 7.30-7.23 (m, 2H), 4.13-4.09 (t, *J* = 8 Hz, 1H), 3.27-3.13 (m, 1H), 2.95-2.87 (m, 1H), 2.46-2.33 (m, 1H), 1.99-1.93 (m, 1H), 1.75-1.63 (m, 4H), 1.61-1.50 (m, 2H), 1.50- 1.39 (m, 1H). ^13^C NMR (100 MHz, CDCl_3_) δ 209.64, 137.12, 129.22, 128.23, 127.90, 126.45 (q, *J* = 277.5 Hz), 51.60 (q, *J* = 2.1 Hz), 36.24 (q, *J* = 27.9 Hz), 30.35, 28.66, 25.95, 25.92. ^19^F NMR (376 MHz, CDCl_3_) δ -64.78 (t, *J* = 11 Hz, 3F. HRMS (EI) m/z calculated for C_15_H_17_F_3_O [M]: 270.1231, found: 270.1233.

**1,2-bis(4-chlorophenyl)-4,4,4-trifluoro-2-methylbutan-1-one (3x)**

Colorless oil, yield = 62%. ^1^H NMR (400 MHz, CDCl_3_) δ 7.24-7.22 (d, *J =* 8 Hz, 2H), 7.19-7.16 (d, *J =* 12 Hz, 2H), 7.13-7.07 (m, 4H), 2.90-2.78 (m, 1H), 2.72-2.63 (m, 1H), 1.66 (s, 3H). ^13^C NMR (100 MHz, CDCl_3_) δ 199.67, 171.29, 139.26, 138.73, 134.20, 133.78, 131.01, 129.72, 128.69, 127.79 (q, *J* = 277.5 Hz), 124.97, 60.52, 51.67 (q, *J* = 2.1 Hz), 43.49 (q, *J* = 27.9 Hz), 22.15, 22.13, 21.15. ^19^F NMR (376 MHz, CDCl_3_) δ -58.56 (t, *J* = 11 Hz, 3F). HRMS (EI) m/z calculated for C_17_H_13_Cl_2_F_3_O [M-F+H]: 360.0296, found: 360.0299.

**2.4 Investigation of Inorganic Bases**

To evaluate the applicability of inorganic bases in the present continuous-flow system, a series of commonly used inorganic bases, including K_2_CO_3_, Cs_2_CO_3_, CsHCO_3_, and Na_2_HPO_4_, were examined under the standard flow conditions.

The results showed that all tested inorganic bases resulted in very low conversions, with HPLC yields of the desired product below 5% (Table S3). In contrast, amine-based organic bases (e.g., TMEDA) provided significantly higher reactivity under identical conditions.

The poor performance of inorganic bases is likely attributed to the limited solubility of inorganic bases in the organic solvent MeCN, resulting in insufficient basicity in the reaction medium. In addition, due to solubility limitations, inorganic bases were fed as solids along with the raw solution into the reaction system, and their dissolution during the reaction process reduced the overall reaction rate and led to poorer overall performance.

**Table S3.** Screening of inorganic bases in the continuous-flow system.^a^

| Entry | Base | Yield of 3a^b^ | |
| --- | --- | --- | --- |
| 1 | K_2_CO_3_ | 1.2% | |
| 2 | Cs_2_CO_3_ | 1.6% | |
| 3 | CsHCO_3_ | 4.3% | |
| 4 | Na_2_HPO_4_ | 2.8% | |
| ^a^ Standard condition: 450-460 nm blue LEDs irradiation (40 W), 0.02 mol·L^-1^ solution of **2a** in MeCN, *mpg*-C_3_N_4_ (20 wt% relative to **2a**), CF_3_SO_2_Cl (1.5 eq.), base (3.0 eq.), V*_milli_*_-COBR_ = 1.8 mL, residence time (τ) = 30 min, nitrogen atmosphere, room temperature, 25 psi. Oscillatory flow condition: *f* = 2 Hz, *x*_o_ = 10 mm. ^b^ Yield of **3a** determined by HPLC analysis based on an external calibration curve of the product. n.d. = not detected. | | |  |

**2.5 Catalyst Recovery and Recycling Experiments**

Catalyst recycling experiments were conducted following the same continuous-flow procedure as described for the synthesis of compound 3a. After steady-state operation conditions, the reactor effluent was collected and analyzed by HPLC to determine the product yield. The suspended *mpg*-C_3_N_4_ catalyst was then recovered by centrifugation, followed by sequential washing with ethanol and deionized water (three times each) to remove residual organic and ionic species. The washed catalyst was dried in a vacuum oven and reused directly in subsequent continuous-flow reactions under identical conditions.

The reaction, collection, and analysis procedures were repeated for several consecutive cycles to evaluate the recyclability and catalytic stability of *mpg*-C_3_N_4_. The results of catalyst recovery and recycling tests are summarized in Table S3.

Table S4. Catalyst recovery and recycling performance of *mpg*-C_3_N_4_ in the continuous-flow synthesis of compound **3a**.

| Run | Yield of **3a**^a^ |
| --- | --- |
| 1 | 86.2% |
| 2 | 86.4% |
| 3 | 86.5% |
| 4 | 86.1% |
| 5 | 84.6% |
| 6 | 82.2% |
| 7 | 79.1% |
| 8 | 74.5% |
| 9^b^ | 85.0% |

^a^ Yield of **3a** determined by HPLC analysis. ^b^ The ninth run was conducted using the recalcined *mpg*-C_3_N_4_.


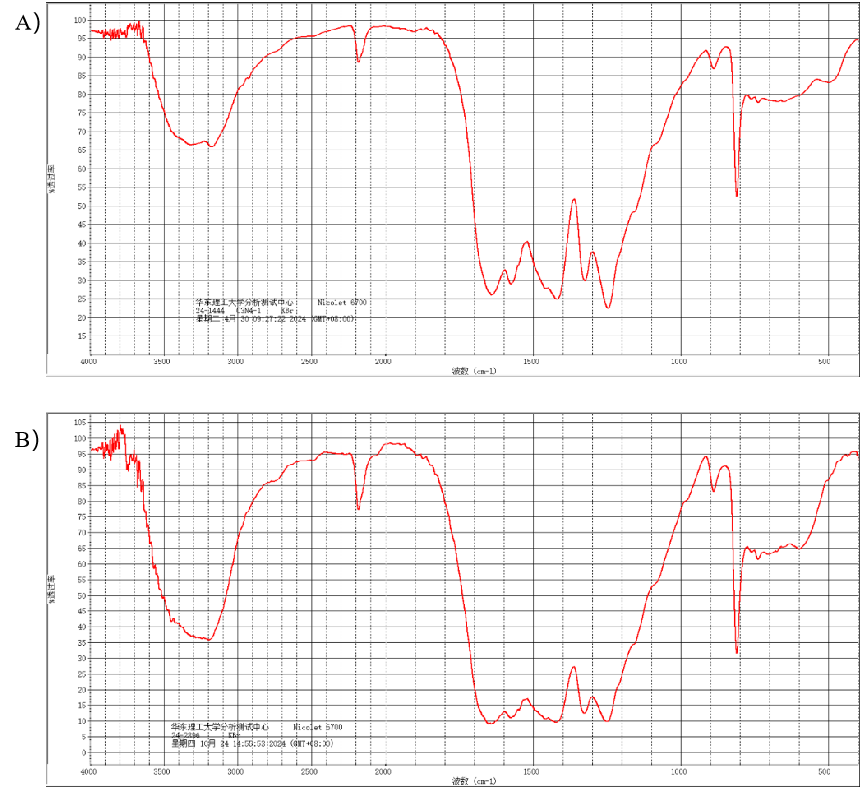


Figure S10. FT-IR spectra of *mpg*-C_3_N_4_ before and after catalytic deactivation

Table S5. Specific surface area change of *mpg*-C_3_N_4_

|  | Unused Catalyst | Catalyst After Eight Uses | Catalyst After Calcination |
| --- | --- | --- | --- |
| Specific Surface Area（m^2^·g^-1^） | 111.17 | 97.12 | 109.53 |

**2.6 Mechanistic Studies**

To gain further insight into the reaction mechanism, two control experiments were conducted. First, when 2,2,6,6-tetramethylpiperidin-1-oxyl (TEMPO) was added as a radical scavenger under the standard reaction conditions, the reaction was completely inhibited, indicating that the transformation proceeds via a radical pathway (Figure S10A).In addition, when α,α-dicyclohexenylmethanol was used as the substrate under the same reaction conditions, no rearranged product was observed (Figure S10B), further supporting the proposed radical-mediated mechanism.


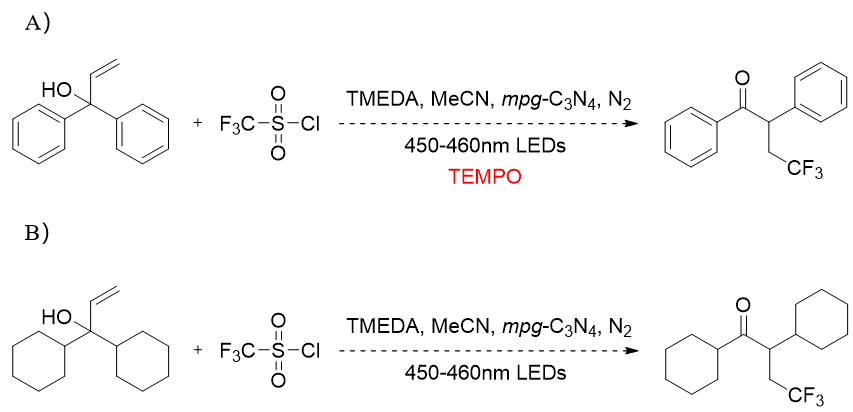


Figure S11. Control experiments for mechanistic verification: (A) Reaction performed in the presence of the radical scavenger 2,2,6,6-tetramethylpiperidin-1-oxyl (TEMPO); (B) Reaction using α,α-dicyclohexenylmethanol as the substrate.

**2.7 Gram Synthesis of Molecule 3j**

The gram-scale synthesis of **3j** was achieved by continuous-flow operation with material accumulation over multiple days.

A typical single run was conducted as follows. Compound **2j** (0.8 mmol, 0.168 g) and *mpg*-C_3_N_4_ (0.034 g) were added to a 100 mL round-bottom flask. Trifluoromethanesulfonyl chloride (TfCl, 0.113 mL, 1.2 mmol), *N*, *N*, *N′*, *N′*-tetramethylethylenediamine (TMEDA, 0.28 mL, 2.4 mmol), and anhydrous acetonitrile (40 mL) were added under a nitrogen atmosphere to form a suspension. The mixture was transferred into a 50 mL glass syringe equipped with a magnetic stir bar. The reaction mixture was delivered into the *milli*-COBR reactor using a syringe pump (TYD02-01, Baoding Lead Fluid Technology Co., Ltd.) at a flow rate of 0.06 mL·min^-1^. The syringe was placed vertically on a magnetic stirrer (85-1, Shanghai Siluo Instrument Co., Ltd.) to maintain a uniform suspension of *mpg*-C_3_N_4_. The reactor was irradiated with a 40 W blue LED panel (450-460 nm, Shenzhen Facai Optoelectronics Technology Co., Ltd.), and a cooling fan was used for heat dissipation. An oscillatory flow field was introduced at the reactor inlet through a T-shaped connector, driven by a piston pump (MP2500-2L (PEEK), Baoding Dirui Electronic Technology Co., Ltd.). The oscillation frequency and amplitude were set to 2 Hz and 10 mm, respectively, and controlled via computer interface. A back-pressure regulator (BPR10, Zaiput Flow Technologies) was installed at the reactor outlet and set to 25 psi to maintain a strong oscillatory intensity. Unless otherwise stated, PTFE capillaries (I.D. 1.0 mm, O.D. 1.6 mm) were used throughout the continuous-flow setup.

After reaching steady-state conditions, the reactor effluent was collected continuously, and each run was operated for approximately 550 min (33 mL).

For gram-scale preparation, the reaction was repeated daily following the same procedure for 14 runs. After each run, the collected solution was concentrated under reduced pressure to remove the solvent. The residues from each run were combined and purified by silica gel column chromatography (300-400 mesh) to afford compound **3j-1** as a colorless oil (1.50 g, 52.7% overall isolated yield).

1. **Synthesis of Bioactive Molecule 6j**

**3.1 Synthesis of Bioactive Molecule 6j in Batch**

To demonstrate the practical applicability of the developed visible-light-mediated strategy, a three-step continuous-flow synthesis of the bioactive molecule **6j**, which exhibits notable antiestrogenic activity, was carried out starting from intermediate **3j**. As shown in Scheme S2, the overall synthetic route involves a Grignard addition reaction to introduce the aryl moiety, followed by an elimination reaction to generate the corresponding olefin intermediate, and finally a reduction reaction to afford the target molecule **6j**. This batch protocol provided both the necessary synthetic reference and analytical standards for subsequent flow chemistry optimization.

Scheme S2. Overall synthetic route from intermediate **3j** to the bioactive molecule **6j**, highlighting the Grignard addition, elimination, and reduction steps.

A typical experimental procedure is described as follows:

Compound **3j** (0.50 g, 1.62 mmol) was placed in a 25 mL three-necked round-bottom flask (Flask A) equipped with a magnetic stir bar (B15-3, Shanghai Sile Instrument Co., Ltd.). Under anhydrous and nitrogen atmosphere, 4-methoxyphenylmagnesium bromide in THF (0.5 M, 4.9 mL) was added, and the mixture was refluxed at 66 ^o^C for 12 h. Reaction progress was monitored by TLC. After completion, the mixture was quenched with ice water and 6 N aqueous acetic acid, and THF was removed under reduced pressure. The aqueous layer was extracted several times with dichloromethane, and the combined organic extracts were dried over anhydrous Na_2_SO_4_ and concentrated to yield intermediate **4j**.

The obtained **4j** was dissolved in anhydrous THF (4.9 mL) in another 25 mL three-necked flask (Flask B), cooled in an ice bath, and maintained under nitrogen. Aqueous HBr solution (48 wt%, 5 mL) was added dropwise, and the reaction was stirred for 2 h. Upon completion (TLC monitoring), the mixture was quenched with ice water and concentrated under reduced pressure (Rotary evaporator, RV-3, IKA). The aqueous phase was extracted with dichloromethane, dried, and concentrated. The crude residue was purified by silica gel column chromatography (300–400 mesh) to give **5j** as a white solid (0.415 g, 83% yield). mp 114.0-116.0 ^o^C. ^1^H NMR (400 MHz, CDCl_3_) δ 7.26-7.22 (t, *J =* 8 Hz, 4H), 7.22-7.18 (t, *J =* 8 Hz, 3H), 6.97-6.95 (d, *J =* 8 Hz, 2H), 6.85-6.83 (d, *J =* 8 Hz, 2H), 6.62-6.60 (d, *J =* 8 Hz, 2H), 3.88 (s, 3H), 3.73 (s, 3H), 3.42-3.35 (m, 2H). ^13^C NMR (100 MHz, CDCl_3_) δ 158.85, 158.01, 144.97, 140.93, 134.73, 134.67, 131.77, 130.66, 129.58, 128.09, 127.45, 126.64, 124.68, 113.39 (q, *J* = 91.9 Hz), 55.17, 40.37 (q, *J* = 27.9 Hz). ^19^F NMR (376 MHz, CDCl_3_) δ -61.60 (t, *J* = 11 Hz, 3F). HRMS (EI) m/z calculated for C_24_H_21_F_3_O_2_ [M]: 398.1494, found: 398.1488.

Subsequently, compound **5j** (0.50 g, 1.26 mmol) was dissolved in anhydrous dichloromethane (5 mL) under nitrogen. The reaction flask was cooled to –78 ^o^C, and boron tribromide solution in dichloromethane (1.0 M, 7.6 mL) was added dropwise. The reaction mixture was then stirred at room temperature for 48 h. After TLC confirmed completion, the mixture was quenched with methanol, and the solvent was evaporated under reduced pressure (Rotary evaporator, RV-3, IKA). The aqueous phase was extracted with dichloromethane, and the combined organic layers were dried and concentrated. The crude product was purified by silica gel column chromatography (300–400 mesh) to afford **6j** as a white solid (0.44 g, 88% yield). mp 158.5-160.0 ^o^C. ^1^H NMR（400 MHz，DMSO-*d*_6_）δ 9.55 (s, 1H), 9.27 (s, 1H), 7.18 (s, 4H), 7.17 (s, 1H), 7.02-7.00 (d, *J =* 8 Hz, 2H), 6.81-6.79 (d, *J =* 8 Hz, 2H), 6.64-6.62 (d, *J =* 8 Hz, 2H), 6.44-6.42 (d, *J =* 8 Hz, 2H), 2.51 (s, 2H). ^13^C NMR (100 MHz, DMSO-*d*_6_) δ 157.07, 156.19, 145.51, 141.17, 133.24, 133.22, 131.69, 130.54, 129.90, 128.33, 128.15, 127.46, 126.88, 125.38, 115.29 (q, *J* = 91.9 Hz), 56.51, 39.98 (q, *J* = 27.9 Hz). ^19^F NMR (376 MHz, DMSO-*d*_6_) δ -60.39 (t, *J* = 11 Hz, 3F). HRMS (EI) m/z calculated for C_22_H_17_F_3_O_2_ [M]: 370.1181, found: 370.1179.

Reagents and Materials: All chemical reagents, unless otherwise specified, were purchased from Shanghai Titan Scientific Co., Ltd., and no further purification was performed. Glassware was obtained from Beijing Xinweier Glass Instrument Co., Ltd.

Characterization: ^1^H, ^13^C, and ^19^F NMR spectra were recorded on a Bruker AVANCE NEO spectrometer. High-resolution mass spectra (HRMS) were acquired using either an EI-GCT Premier or ESI-Xevo G2 TOF mass spectrometer (Waters Technology (Shanghai) Co., Ltd.).

This batch synthesis provided a benchmark for comparison with the continuous-flow process. In the following section, the corresponding continuous-flow synthesis and process optimization are described in detail.

**3.2 Step 1: Grignard Addition Reaction in Flow**

The continuous-flow setup for the synthesis of compound **4j** is shown in Figure S11. A typical procedure is described as follows:

Compound **3j** (1.93 g, 6.25 mmol) was placed in a 50 mL two-necked round-bottom flask under anhydrous and nitrogen atmosphere. Anhydrous tetrahydrofuran (THF, 25 mL) was added to dissolve the substrate, and the resulting solution was transferred into a 10 mL glass syringe (Syringe A). The solution was pumped at a flow rate of 0.025 mL·min⁻¹ using a syringe pump (TYD02-01, Baoding Lead Fluid Technology Co., Ltd.). A 0.5 M solution of 4-methoxyphenylmagnesium bromide in THF (10 mL) was loaded into another 10 mL glass syringe (Syringe B) and delivered at the same flow rate (0.025 mL·min⁻¹) using a second syringe pump (TYD02-01, Baoding Lead Fluid Technology Co., Ltd.). The two streams were combined through a T-shaped micromixer and then passed into a PTFE capillary microreactor (O.D. 1.6 mm, I.D. 0.8 mm, total volume 0.5 mL), where the Grignard addition reaction occurred. The microreactor was immersed in a water bath maintained at 25 °C for temperature control.

At the outlet of the microreactor, the effluent stream was merged via another T-shaped junction with a 6 N aqueous acetic acid quenching solution, delivered by syringe pump C (TYD02-01, Baoding Lead Fluid Technology Co., Ltd.) at a flow rate of 0.05 mL·min⁻¹. After steady-state operation conditions, three parallel samples of the reactor effluent were collected. Each sample was diluted tenfold with methanol, filtered, and analyzed by HPLC to determine the product yield.


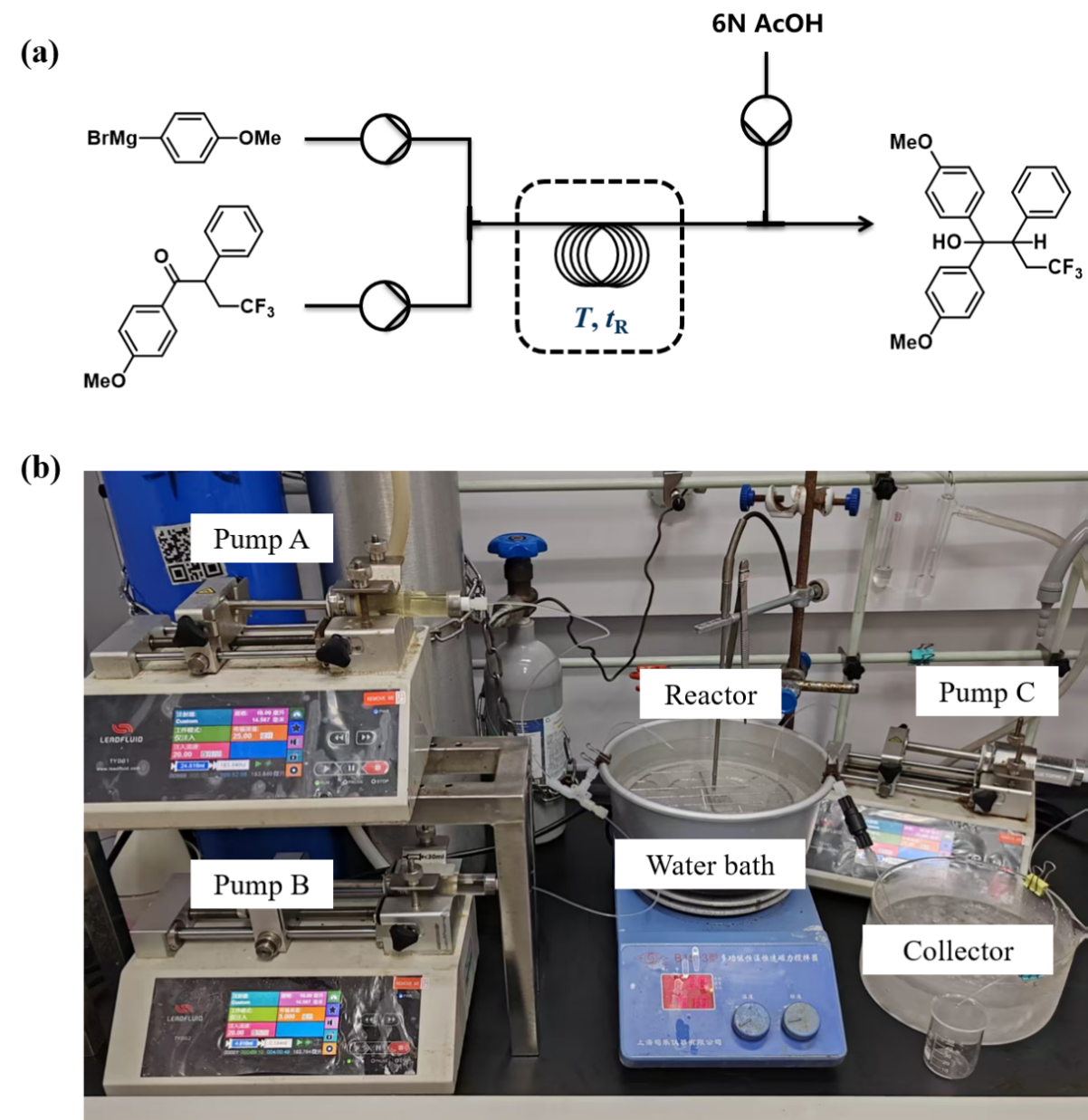


Figure S12. Photograph of continuous-flow setup for the synthesis of compound **4j**.

Reagents and Materials: All chemical reagents, except for substrates **3j**, were purchased from Shanghai Titan Scientific Co., Ltd., and no further purification was performed. Glassware was obtained from Beijing Xinweier Glass Instrument Co., Ltd. And all accessories related to the continuous-flow experiments were purchased from Nanjing Runze Fluid Control Equipment Co., Ltd.

To further improve the reaction performance, key operating parameters, including substrate concentration, reaction temperature, residence time, and equivalents of Grignard reagent, were systematically optimized. The detailed results of process parameter screening are summarized in Table S5.

Table S6. Optimization of Grignard reaction conditions in flow.

| Entry | Concentration of **3j**  （mol·L^-1^） | Temperature（^o^C） | Residence time（min） | Equivalent of Grignard reagent  （eq.） | Conversion of **3j**（%）^a^ |
| --- | --- | --- | --- | --- | --- |
| 1 | 0.25 | 25 | 5 | 2.0 | 84.8 |
| 2 | 0.25 | 25 | 10 | 2.0 | 99.8 |
| 3 | 0.25 | 25 | 15 | 2.0 | 99.8 |
| 4 | 0.25 | 50 | 1 | 2.0 | 85.4 |
| 5 | 0.25 | 50 | 5 | 2.0 | 97.9 |
| 6 | 0.25 | 50 | 10 | 2.0 | 99.8 |
| 7 | 0.25 | 50 | 15 | 2.0 | 99.7 |
| 8 | 0.25 | 66 | 1 | 2.0 | 87.3 |
| 9 | 0.25 | 66 | 5 | 2.0 | 99.4 |
| 10 | 0.25 | 66 | 10 | 2.0 | 99.6 |
| 11 | 0.25 | 66 | 15 | 2.0 | 99.8 |
| 12 | 0.25 | 25 | 10 | 1.0 | 94.6 |
| 13 | 0.25 | 25 | 10 | 1.1 | 98.0 |
| 14 | 0.25 | 25 | 10 | 1.2 | 99.7 |
| 15 | 0.25 | 25 | 10 | 1.5 | 99.8 |
| 16 | 0.25 | 25 | 10 | 1.8 | 99.6 |
| 17 | 0.42 | 25 | 1 | 1.2 | 90.4 |
| 18 | 0.42 | 25 | 2 | 1.2 | 95.4 |
| 19 | 0.42 | 25 | 5 | 1.2 | 99.9 |

^a^ Conversion determined by HPLC.

**3.3 Step 2: HBr-Mediated Elimination Reaction in Flow**

The continuous-flow setup for the synthesis of compound **5j** is shown in Figure S12. A typical procedure is described as follows:

Compound **3j** (3.2 g, 10.42 mmol) was weighed into a 50 mL two-necked round-bottom flask. Under anhydrous and nitrogen atmosphere, anhydrous tetrahydrofuran (THF, 25 mL) was added to dissolve the substrate, and the solution was transferred into a 10 mL glass syringe (Syringe A). Syringe A was mounted on a syringe pump (TYD02-02, Baoding Lead Fluid Technology Co., Ltd.) and delivered at a flow rate of 0.05 mL·min⁻¹. Separately, a 10 mL glass syringe was loaded with a THF solution of p-methoxyphenylmagnesium bromide (0.5 M, 10 mL) and pumped at an identical flow rate of 0.05 mL·min⁻¹. The two streams were combined through a T-shaped mixer and introduced into a capillary microreactor 1 (O.D. 1.6 mm, I.D. 0.8 mm, total volume 0.5 mL) to undergo the Grignard addition reaction. The outlet of microreactor 1 was connected to another T-shaped mixer, where the reaction effluent was merged with a 48 wt% aqueous solution of hydrobromic acid (HBr), which was pumped at a flow rate of 0.10 mL·min⁻¹. The mixture was then passed through capillary microreactor 2 (O.D. 1.6 mm, I.D. 0.8 mm, total volume 0.1 mL) to perform the elimination reaction.

Both microreactors were immersed in a thermostatic water bath maintained at 25 °C. At the outlet of microreactor 2, the effluent was quenched with water introduced through another syringe pump (TYD02-01, Baoding Lead Fluid Technology Co., Ltd.) at a flow rate of 0.20 mL·min⁻¹. After steady-state operation conditions, three parallel samples of the outflow were collected, diluted tenfold with methanol, filtered, and analyzed by HPLC to determine the reaction yield.


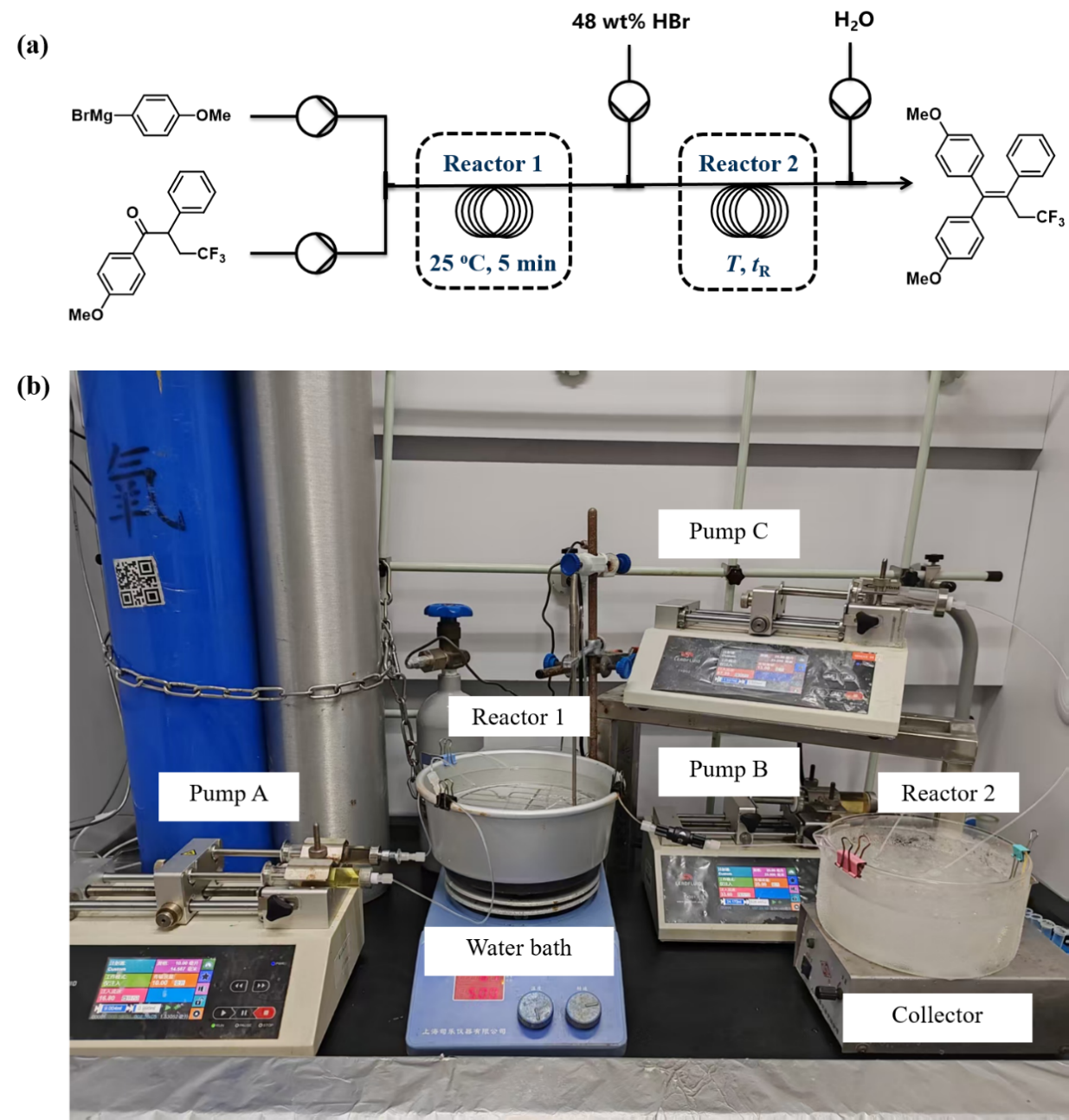


Figure S13. Photograph of the continuous-flow setup for the synthesis of compound **5j**.

Reagents and Materials: All chemical reagents, except for substrates **3j**, were purchased from Shanghai Titan Scientific Co., Ltd., and no further purification was performed. Glassware was obtained from Beijing Xinweier Glass Instrument Co., Ltd. And all accessories related to the continuous-flow experiments were purchased from Nanjing Runze Fluid Control Equipment Co., Ltd.

The optimization of continuous-flow reaction parameters for the HBr-mediated elimination step is summarized in Table S6.

Table S7. Optimization of elimination reaction conditions in flow.

| Entry | Temperature（^o^C） | Residence time（s） | Yield of **5j**（%）^a^ |
| --- | --- | --- | --- |
| 1 | 0 | 10 | 37.1 |
| 2 | 0 | 20 | 56.0 |
| 3 | 0 | 30 | 75.2 |
| 4 | 0 | 60 | 89.9 |
| 5 | 0 | 300 | 97.5 |
| 6 | 25 | 10 | 75.9 |
| 7 | 25 | 20 | 87.4 |
| 8 | 25 | 30 | 97.4 |
| 9 | 25 | 60 | 97.3 |
| 10 | 25 | 300 | 97.0 |

^a^ Yield of **5j** determined by HPLC.

**3.4 Step 3: BBr₃-Mediated Deprotection Reaction in Flow**

The continuous-flow setup for the synthesis of compound **6j** is shown in Figure S13. A typical experimental procedure is as follows:

Compound **5j** (0.66 g, 1.67 mmol) was weighed into a 25 mL two-necked round-bottom flask. Under anhydrous and nitrogen atmosphere, anhydrous dichloromethane (DCM, 10 mL) was added to dissolve the substrate, and the resulting solution was transferred into a 10 mL glass syringe (Syringe A). Syringe A was mounted on a syringe pump (TYD02-02, Baoding Lead Fluid Technology Co., Ltd.) and delivered at a flow rate of 0.01 mL·min⁻¹. Separately, a 10 mL glass syringe (Syringe B) was filled with a DCM solution of boron tribromide (BBr_3_, 1.0 M, 7.6 mL) and pumped at an identical flow rate of 0.01 mL·min⁻¹. The two liquid streams were combined via a T-shaped mixer and introduced sequentially into capillary microreactor 1 (O.D. 1.6 mm, I.D. 0.8 mm, total volume 0.1 mL) and capillary microreactor 2 (O.D. 1.6 mm, I.D. 0.8 mm, total volume 0.4 mL) for the deprotection reaction.

Microreactor 1 was maintained in an ice bath, while microreactor 2 was temperature-controlled in a water bath at ambient temperature. At the outlet of microreactor 2, the reaction effluent was merged with methanol through another T-shaped mixer to quench the reaction. Methanol was delivered by a single-channel syringe pump (TYD02-01, Baoding Lead Fluid Technology Co., Ltd.) at a flow rate of 0.20 mL·min⁻¹. After the system reached steady-state operation conditions, three parallel samples of the reaction outflow were collected, filtered, and analyzed by HPLC to determine the product yield.


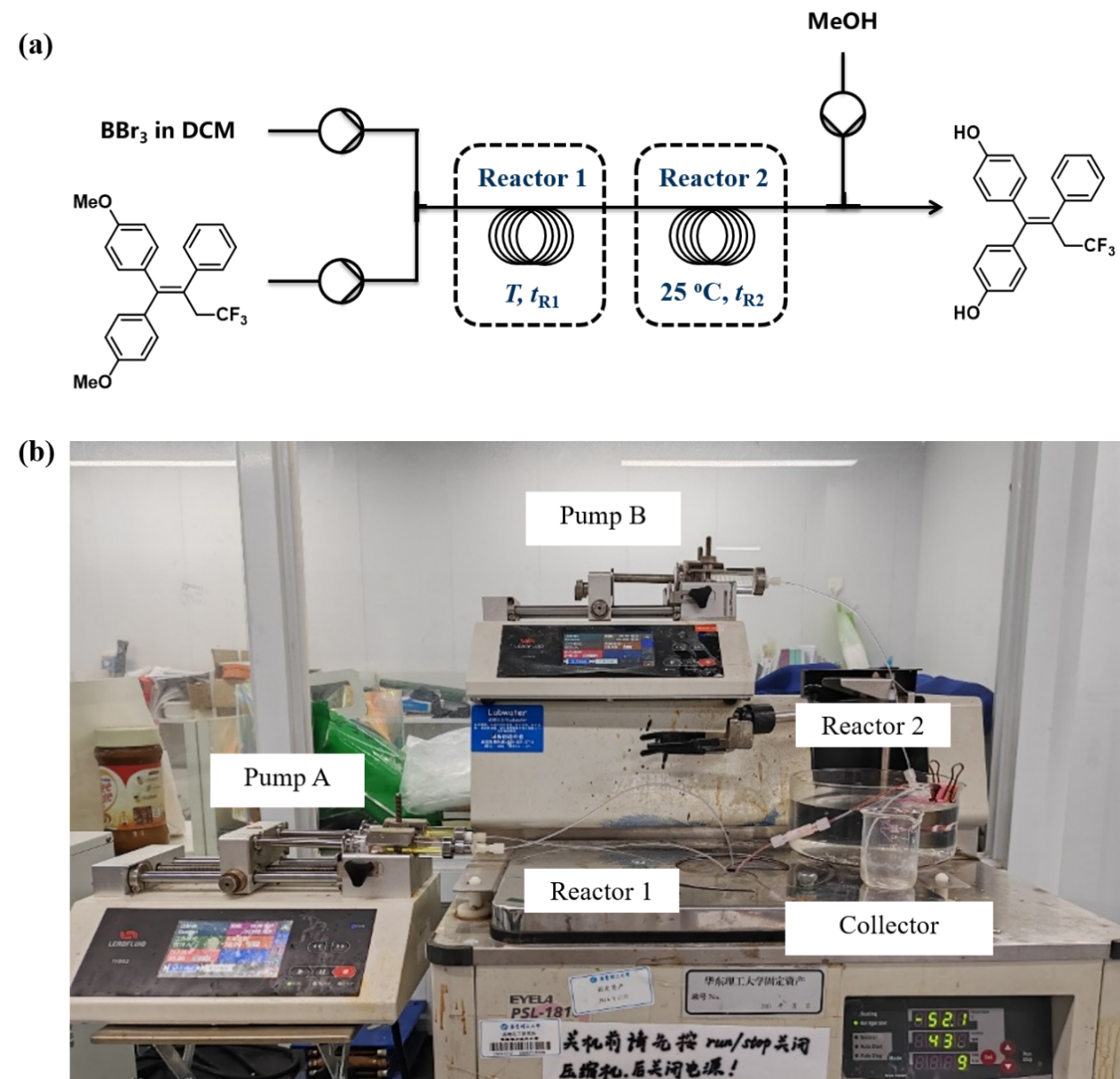


Figure S14. Photograph of the continuous-flow setup for the synthesis of compound **6j**.

Reagents and Materials: All chemical reagents, except for substrates **5j**, were purchased from Shanghai Titan Scientific Co., Ltd., and no further purification was performed. Glassware was obtained from Beijing Xinweier Glass Instrument Co., Ltd. And all accessories related to the continuous-flow experiments were purchased from Nanjing Runze Fluid Control Equipment Co., Ltd.

The optimization of continuous-flow parameters for the BBr_3_-mediated deprotection reaction is summarized in Table S7.

Table S8. Optimization of BBr_3_-mediated deprotection reaction conditions in flow.^a^

| Entry | Temperature（^o^C） | Residence time（min） | Equivalent of BBr_3_  （eq.） | Conversion of **5j**  （%）^b^ | Yield of **6j**  （%）^b^ |
| --- | --- | --- | --- | --- | --- |
| 1 | -52 | 10 | 6 | 81.0 | 40.2 |
| 2 | -52 | 15 | 6 | 93.1 | 63.8 |
| 3 | -52 | 20 | 6 | 98.4 | 83.7 |
| 4 | -52 | 25 | 6 | 99.3 | 90.1 |
| 5 | -52 | 30 | 6 | 99.6 | 96.6 |
| 6 | -20 | 10 | 6 | 85.6 | 48.0 |
| 7 | -20 | 15 | 6 | 97.2 | 70.7 |
| 8 | -20 | 20 | 6 | 99.2 | 89.3 |
| 9 | -20 | 25 | 6 | 99.4 | 92.7 |
| 10 | -20 | 30 | 6 | 99.8 | 96.2 |
| 11 | 0 | 10 | 6 | 95.5 | 66.3 |
| 12 | 0 | 15 | 6 | 97.9 | 78.4 |
| 13 | 0 | 20 | 6 | 99.7 | 92.2 |
| 14 | 0 | 25 | 6 | 99.8 | 96.4 |
| 15 | 0 | 30 | 6 | 99.8 | 95.9 |
| 16 | 0 | 25 | 1 | 95.2 | 59.4 |
| 17 | 0 | 25 | 2 | 99.0 | 66.4 |
| 18 | 0 | 25 | 3 | 99.7 | 76.3 |
| 19 | 0 | 25 | 4 | 99.8 | 94.5 |
| 20 | 0 | 25 | 5 | 99.9 | 96.2 |

^a^ Using 1.0 M BBr_3_ solution in DCM. ^b^ Determined by HPLC.


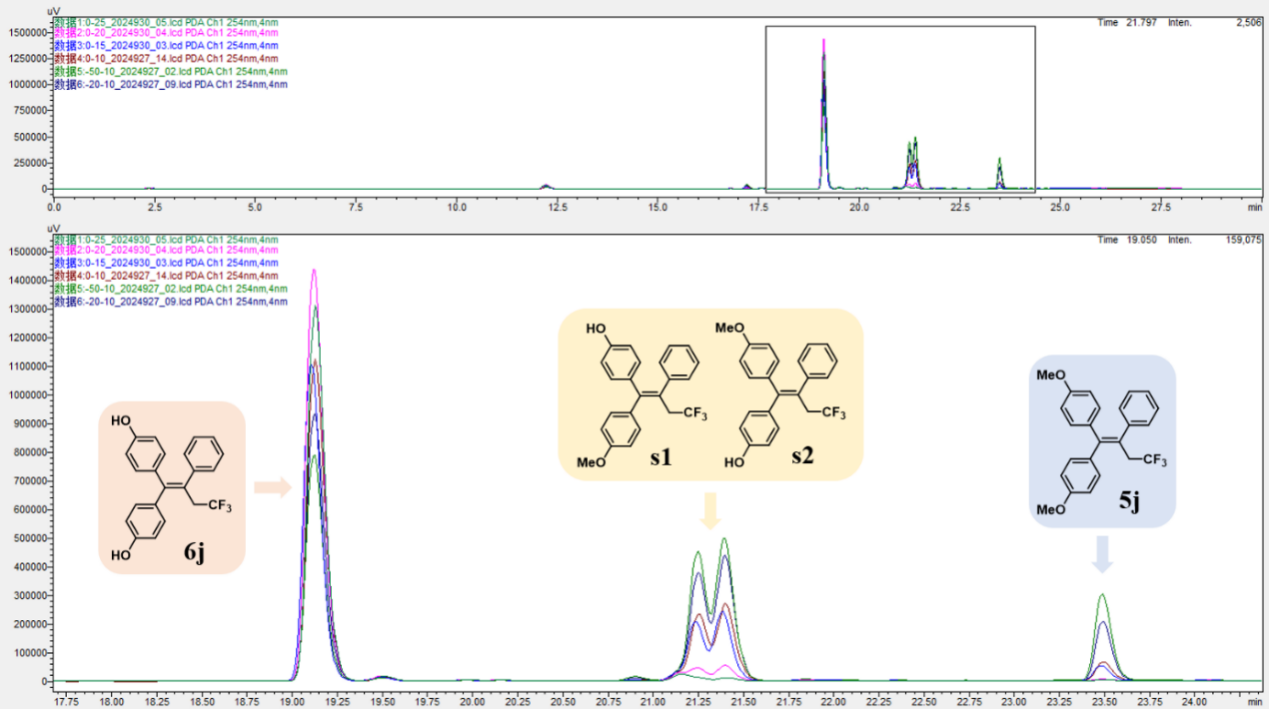


Figure S15. HPLC chromatograms of deprotection reaction samples under varying conditions.

1. **Spectroscopic Data**


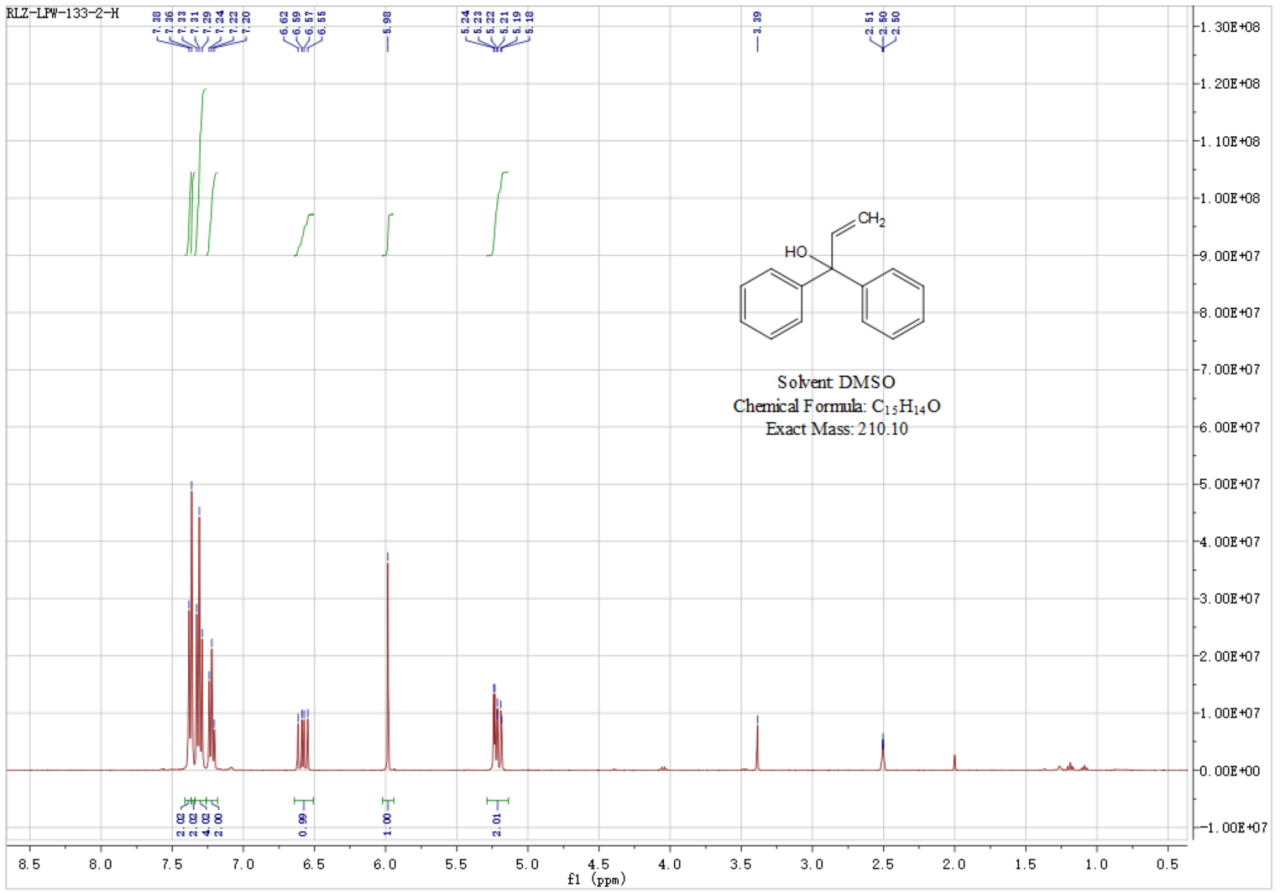


Figure S16. The ^1^H NMR of compound **2a.**


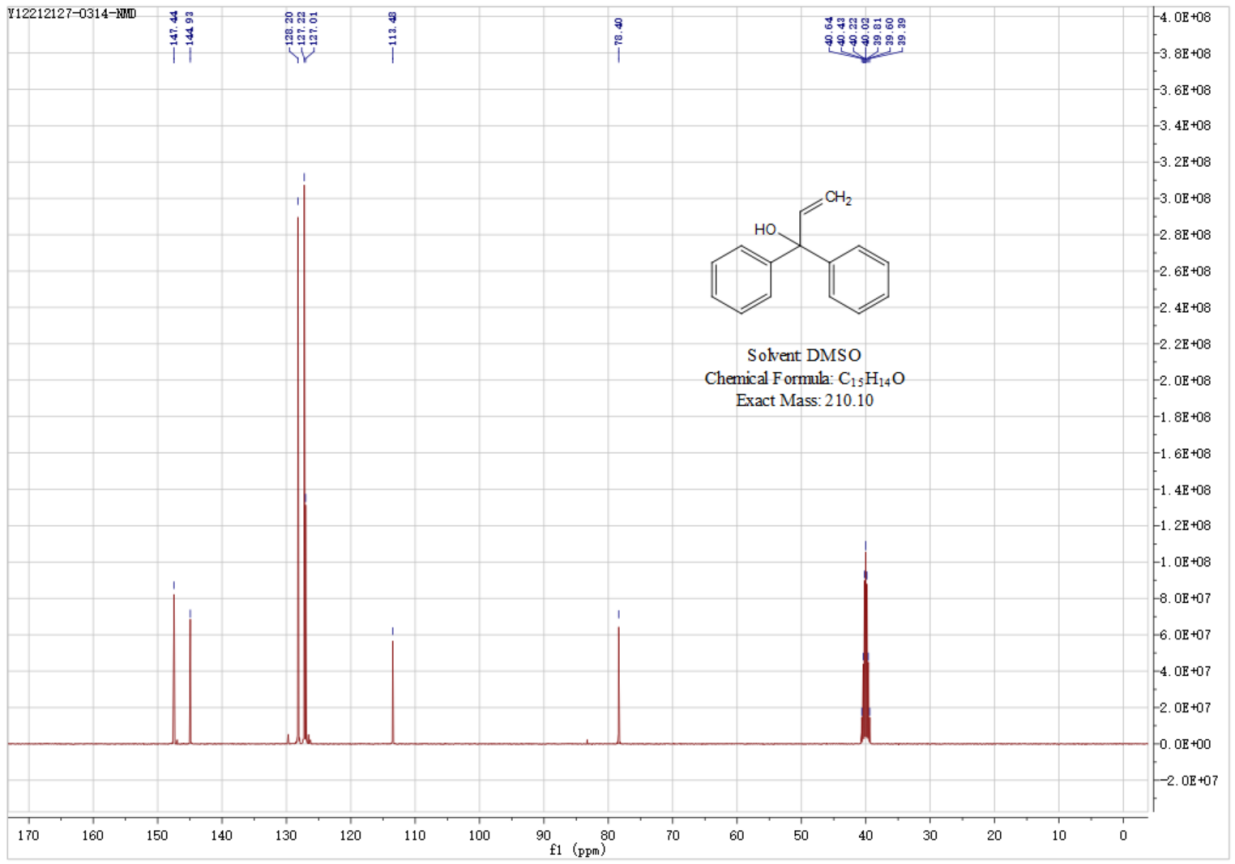


Figure S17. The ^13^C NMR of compound **2a.**


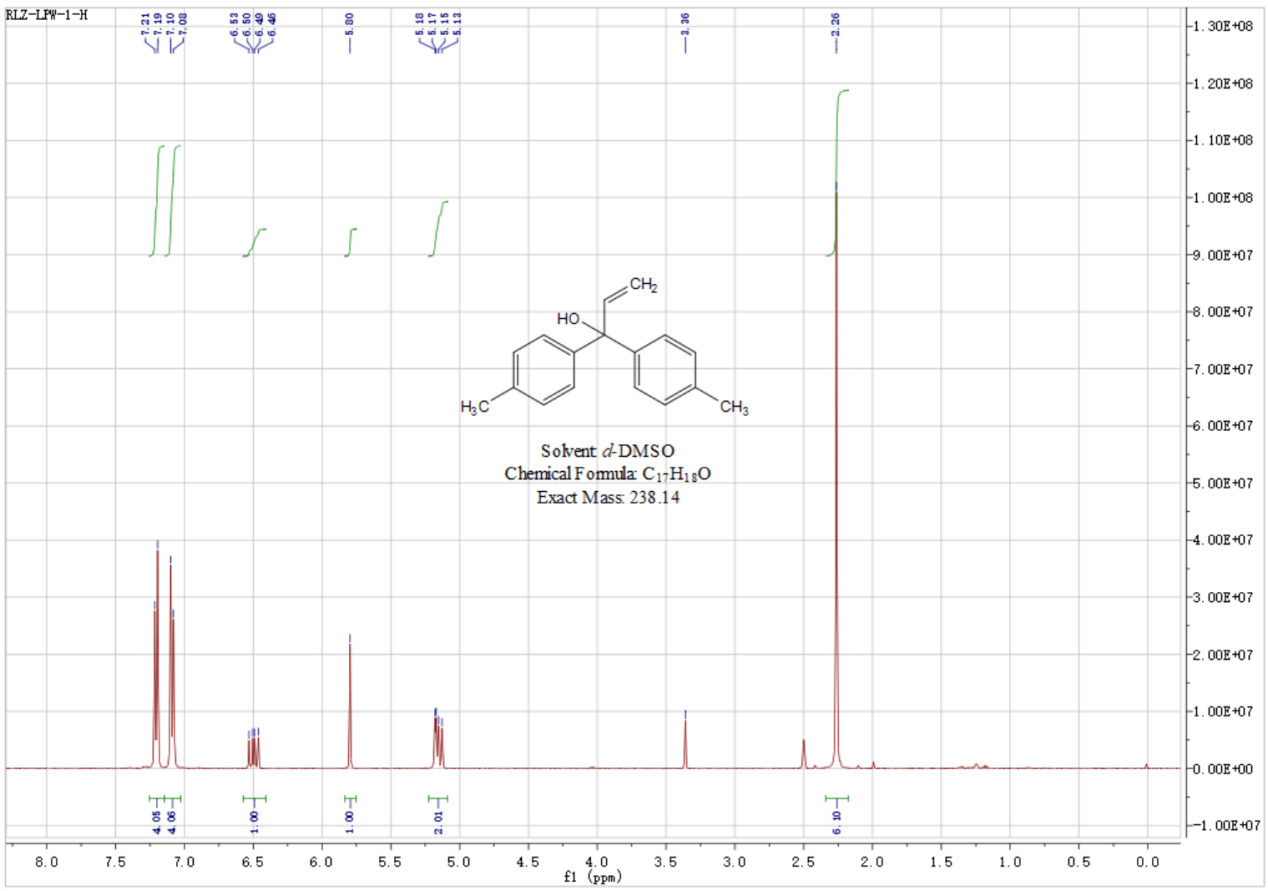


Figure S18. The ^1^H NMR of compound **2b.**


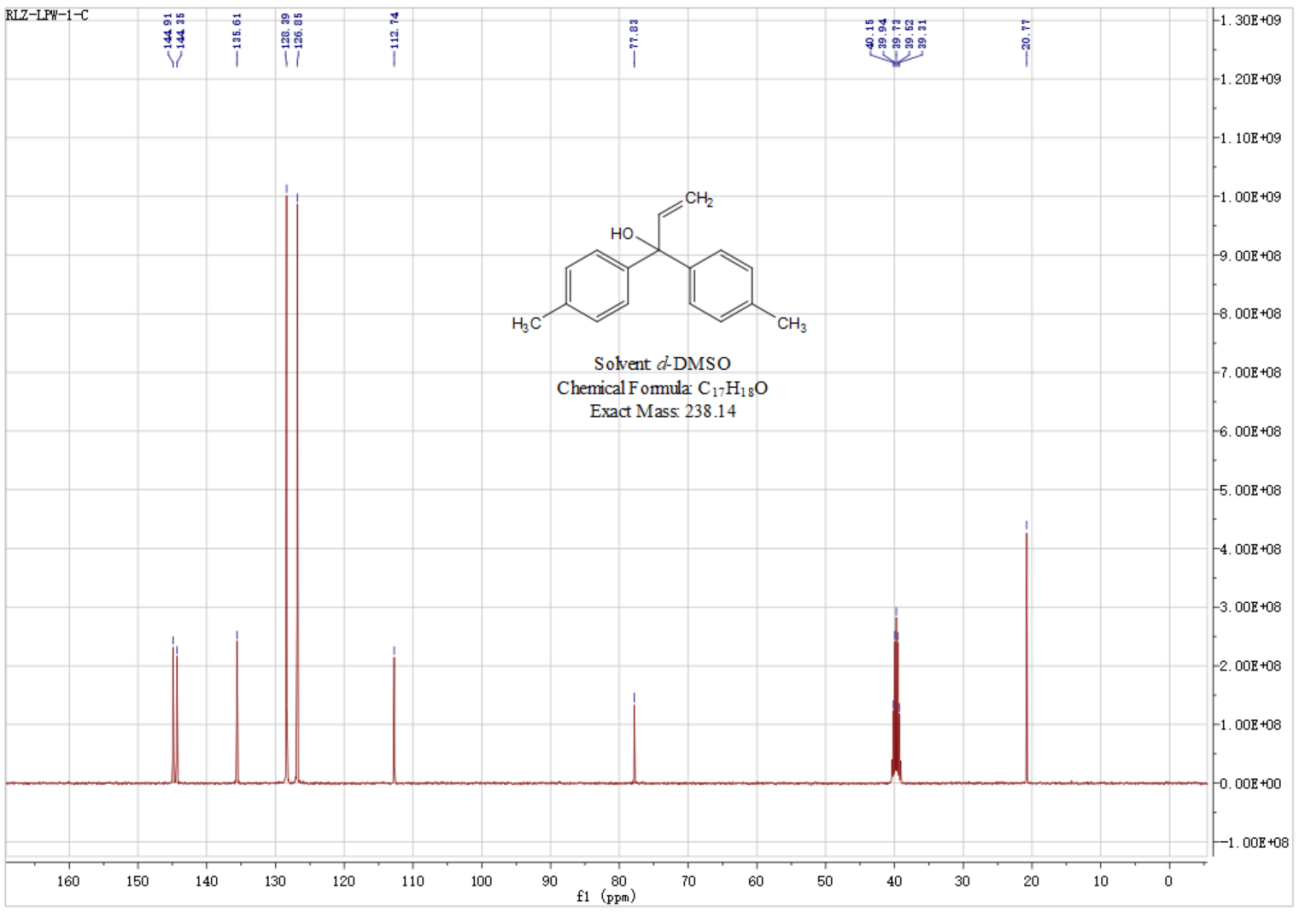


Figure S19. The ^13^C NMR of compound **2b.**


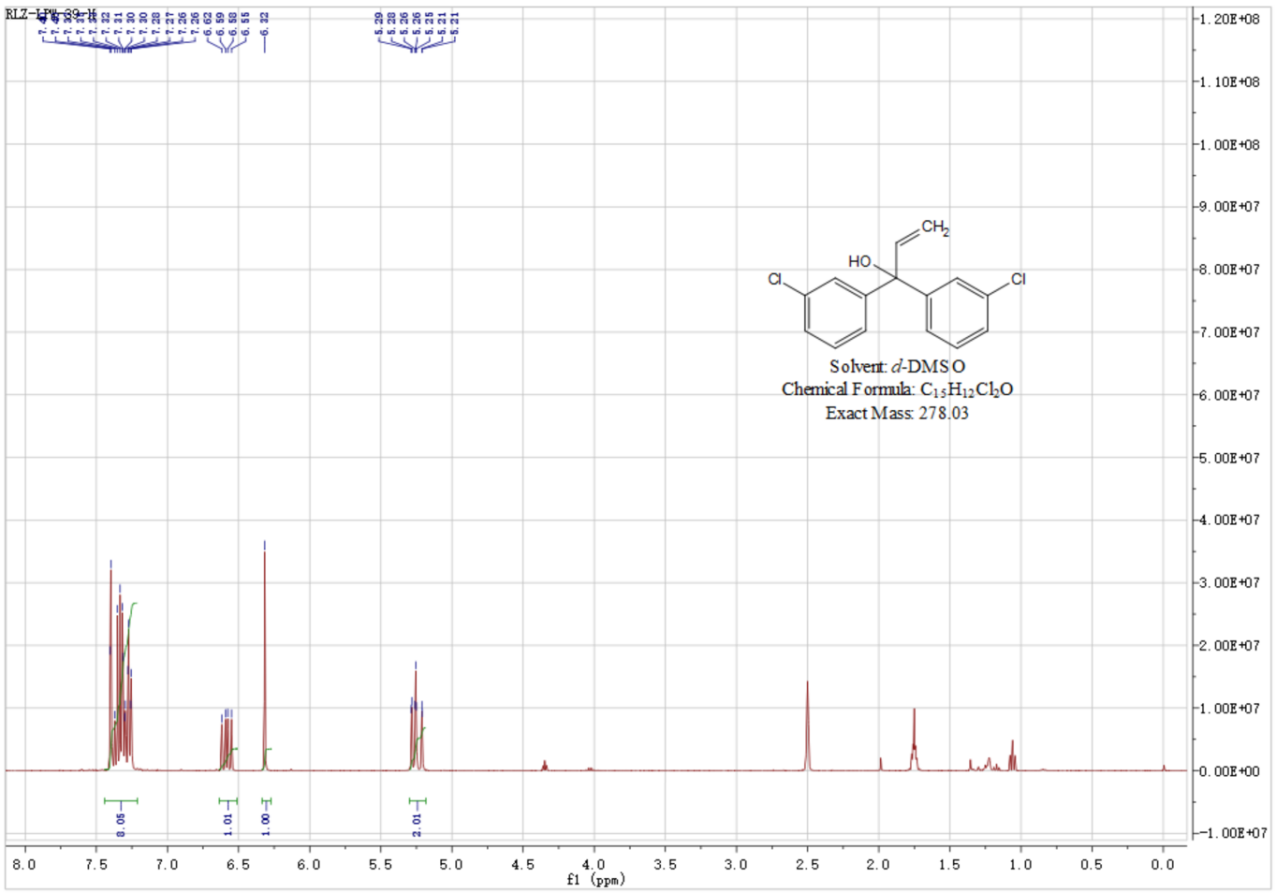


Figure S20. The ^1^H NMR of compound **2c.**


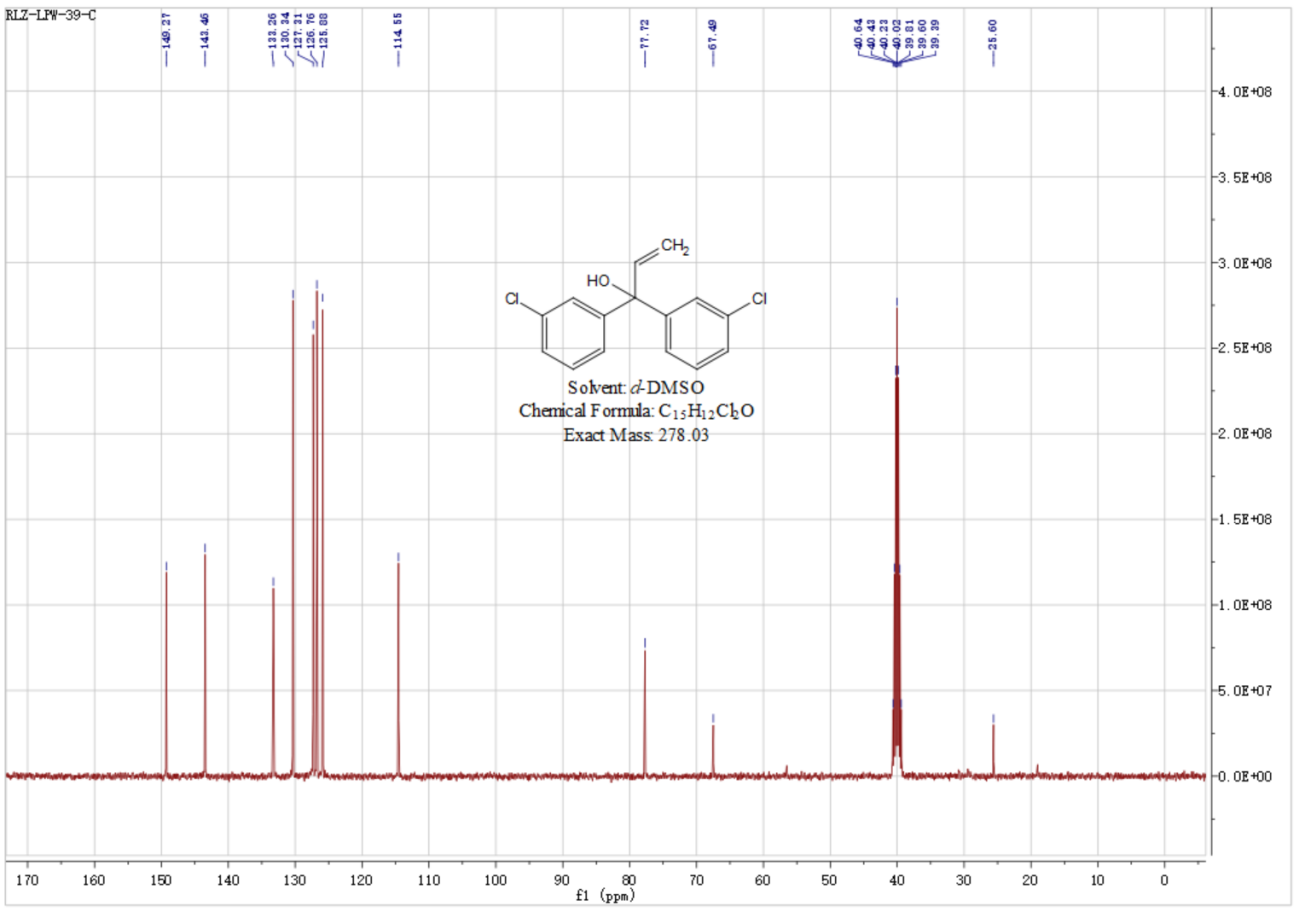


Figure S21. The ^13^C NMR of compound **2c.**


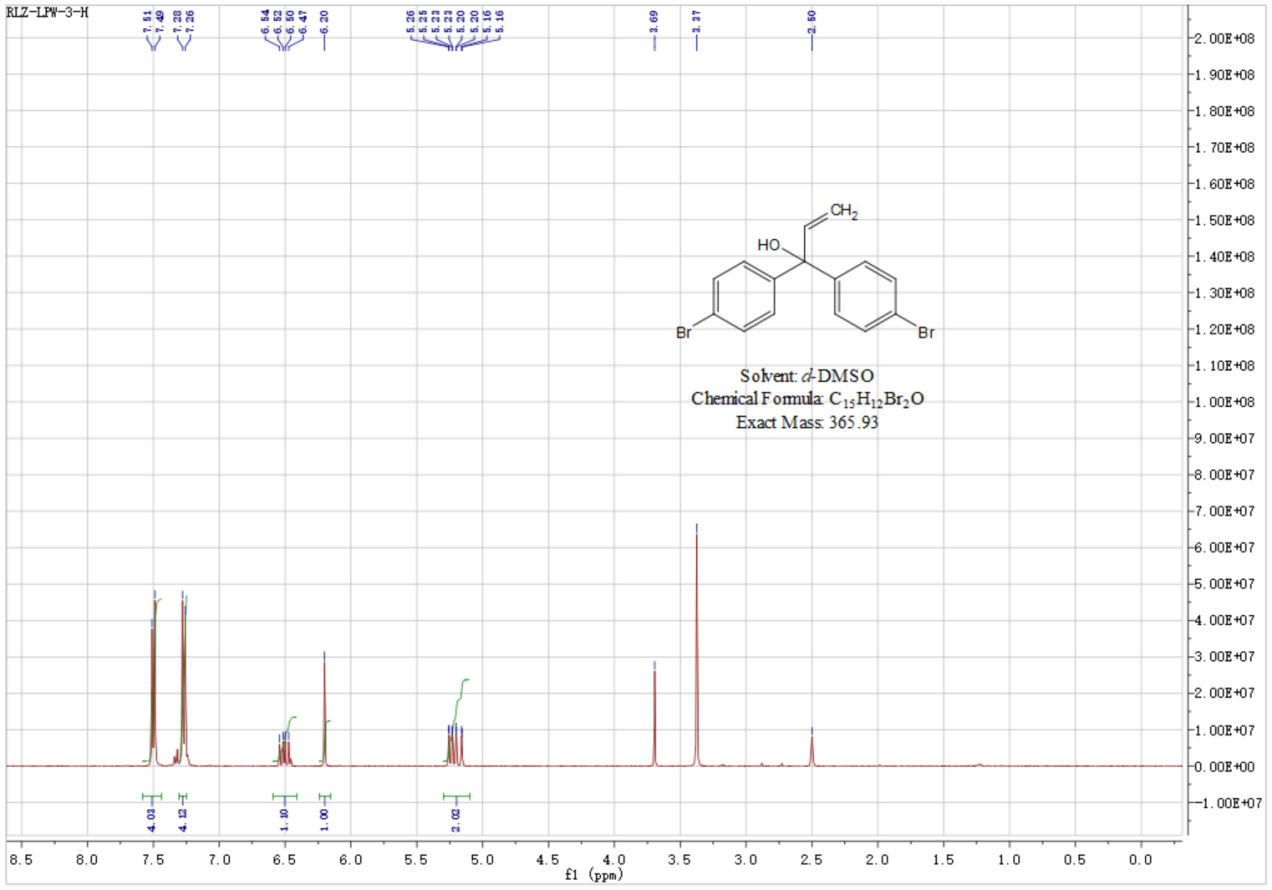


Figure S22. The ^1^H NMR of compound **2d.**


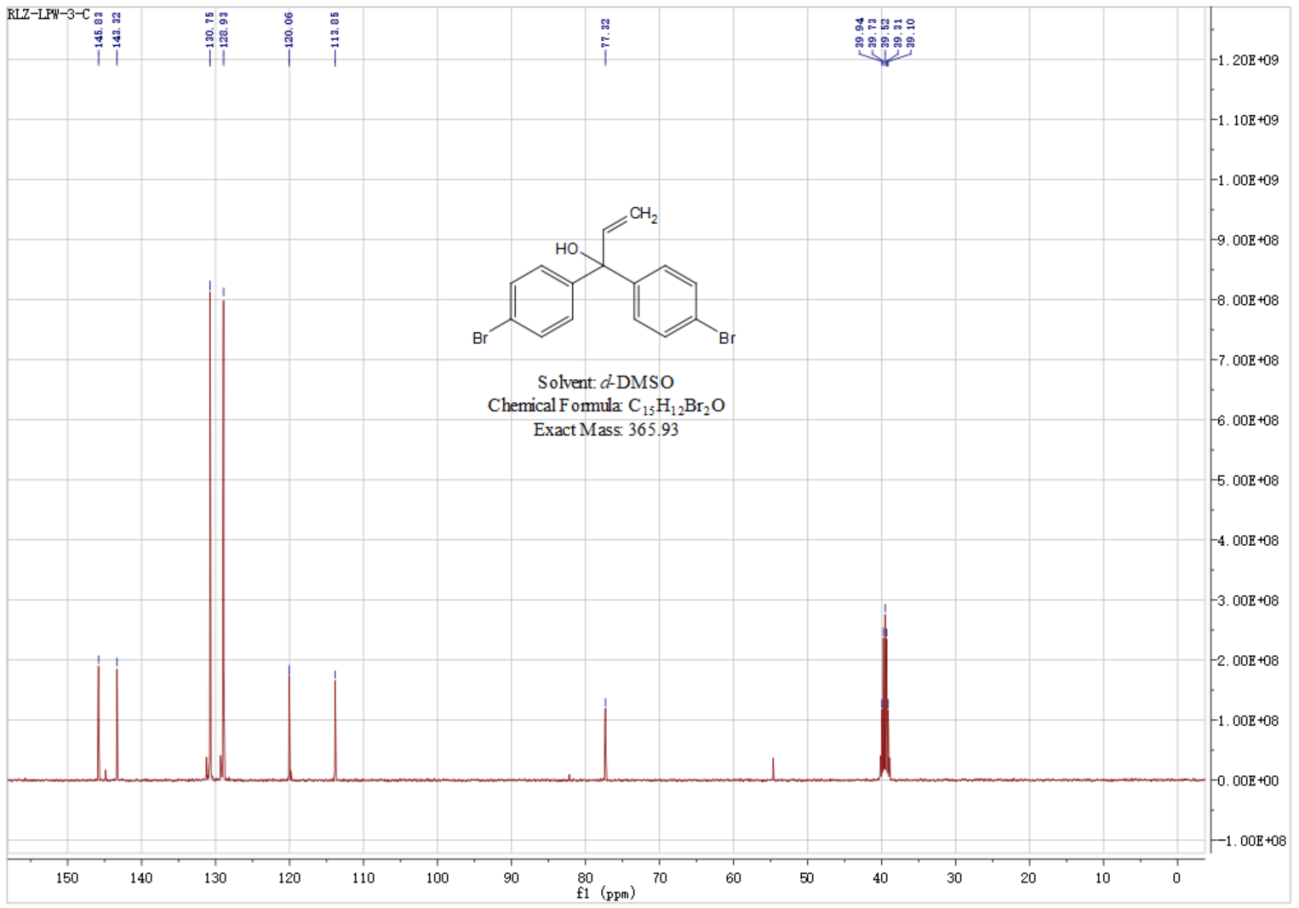


Figure S23. The ^13^C NMR of compound **2d.**


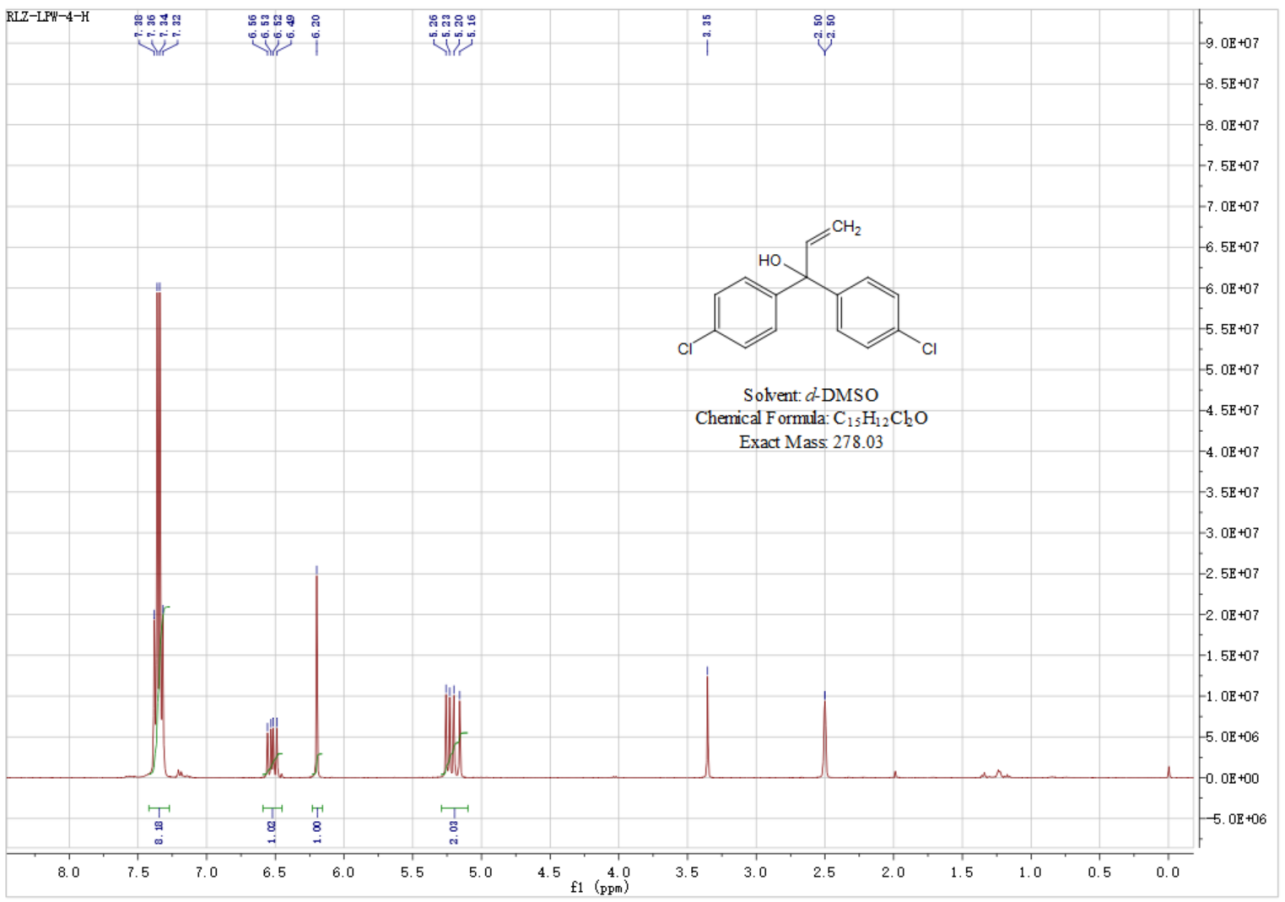


Figure S24. The ^1^H NMR of compound **2e.**


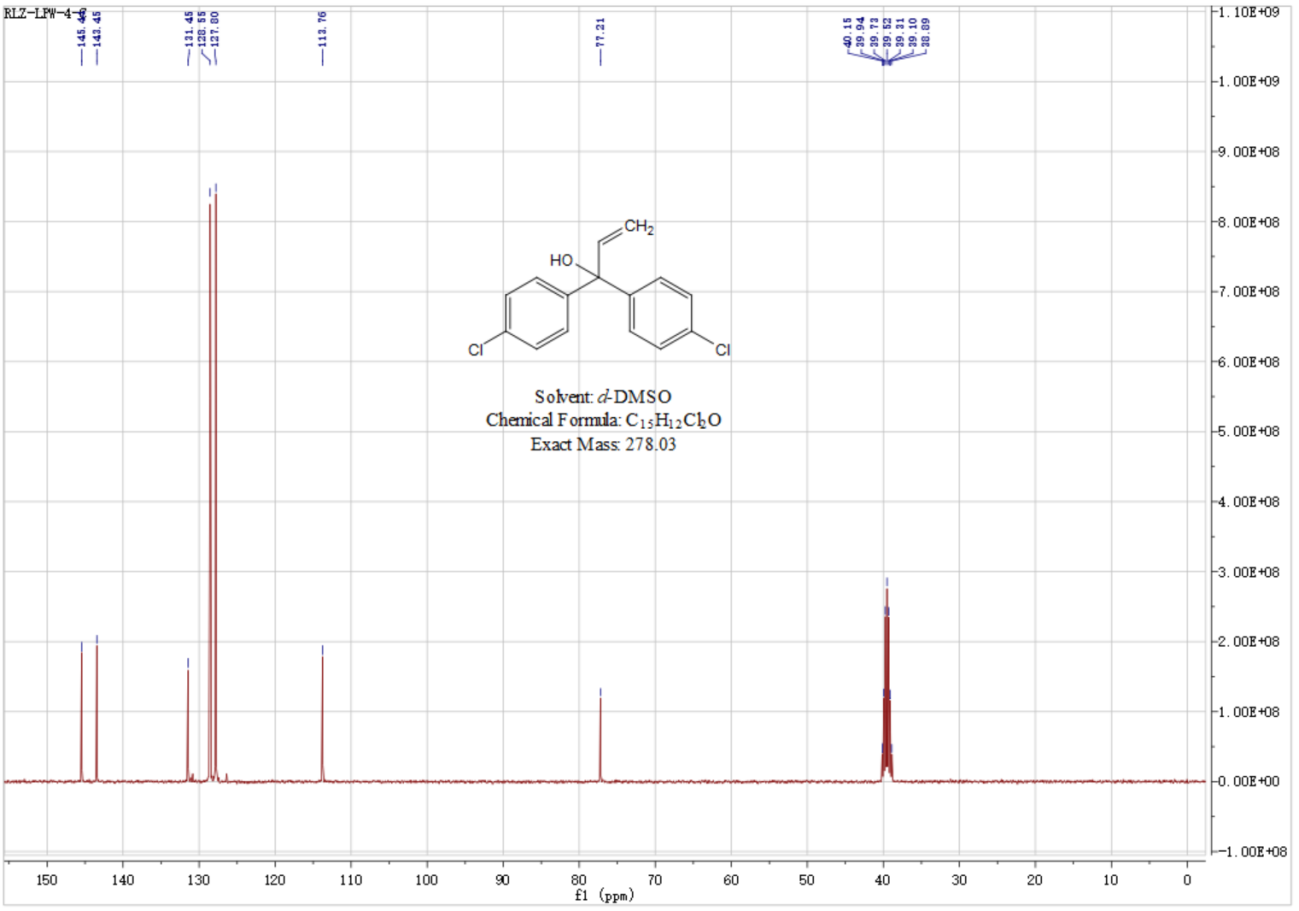


Figure S25. The ^13^C NMR of compound **2e.**


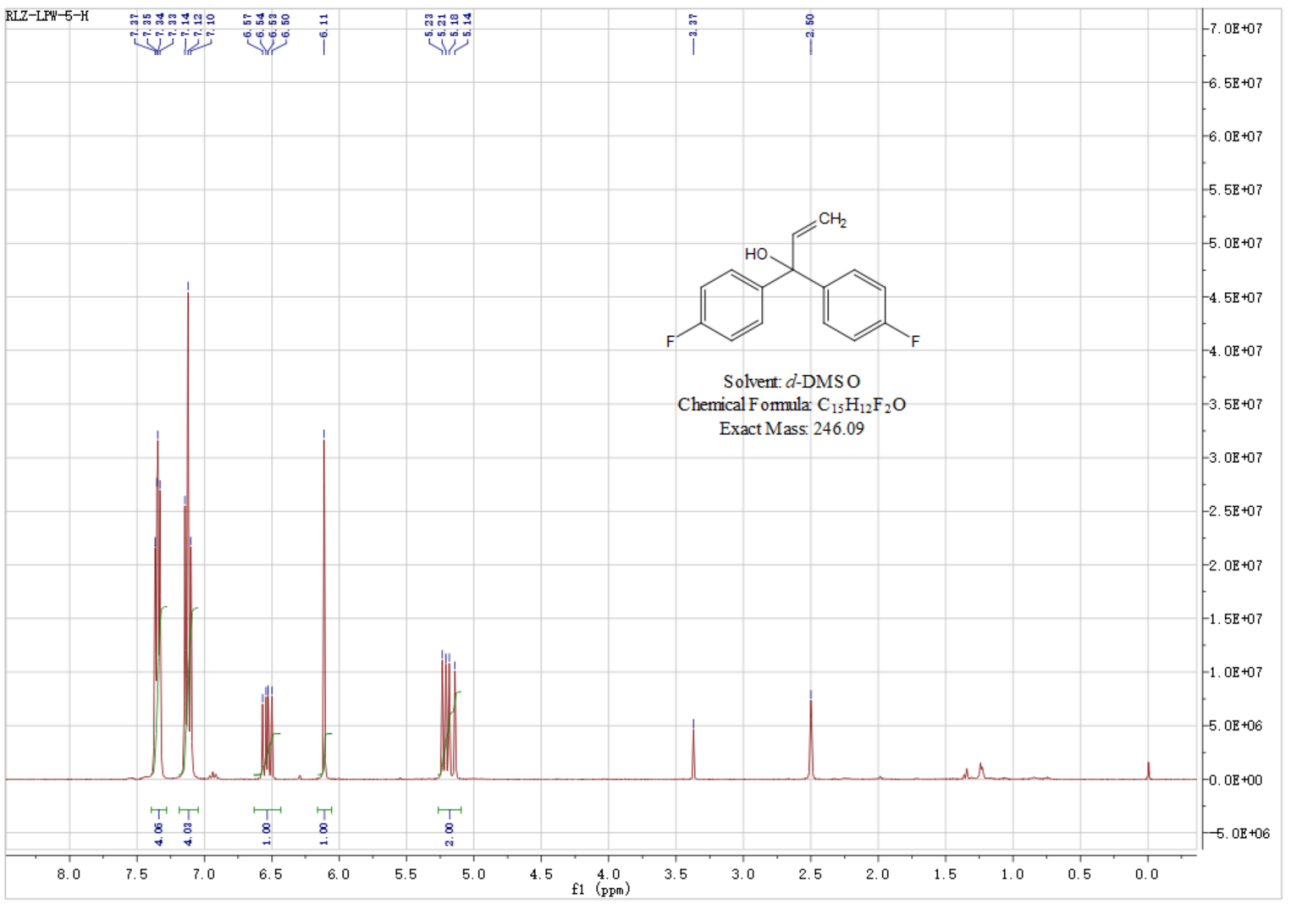


Figure S26. The ^1^H NMR of compound **2f.**


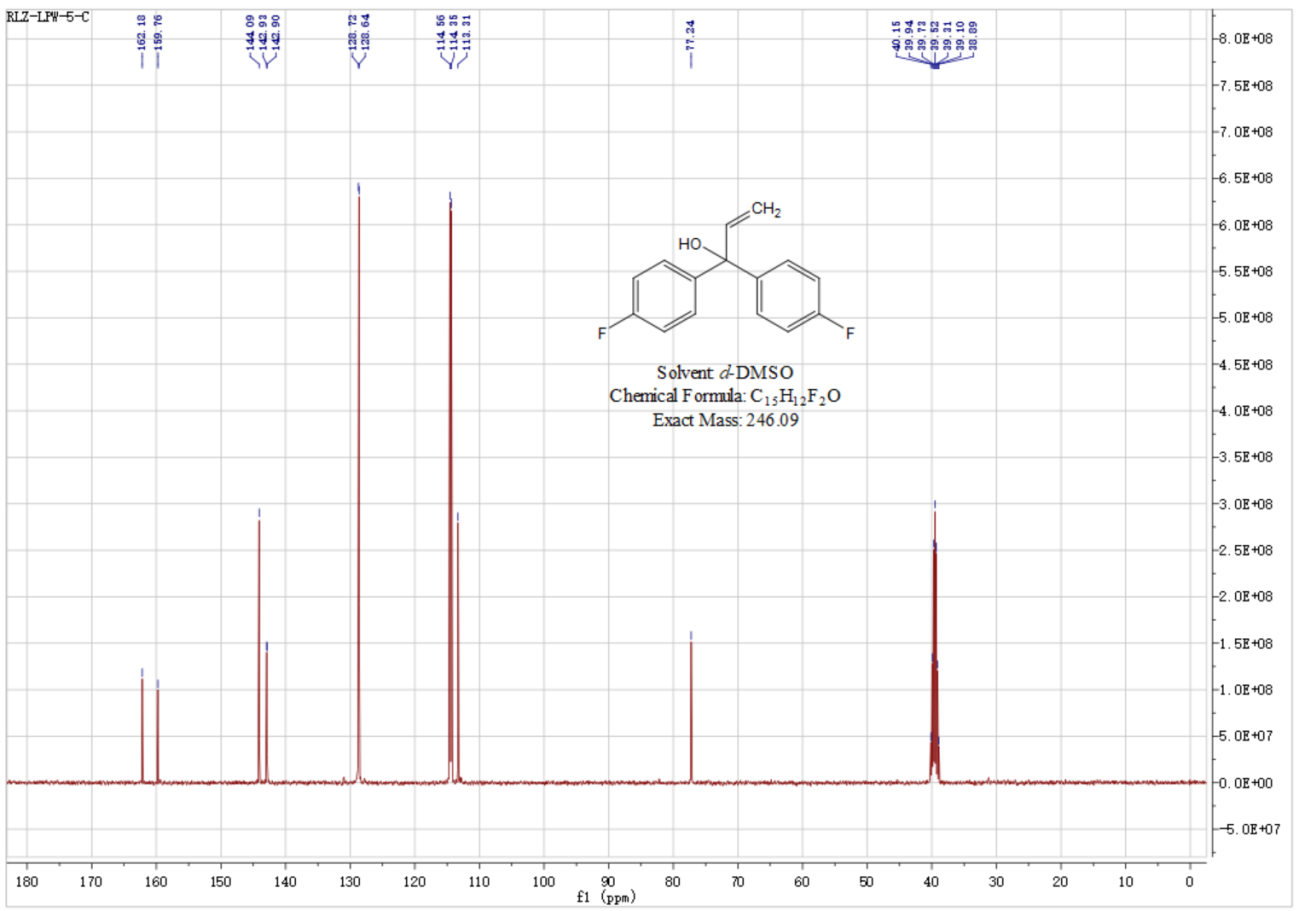


Figure S27. The ^13^C NMR of compound **2f.**


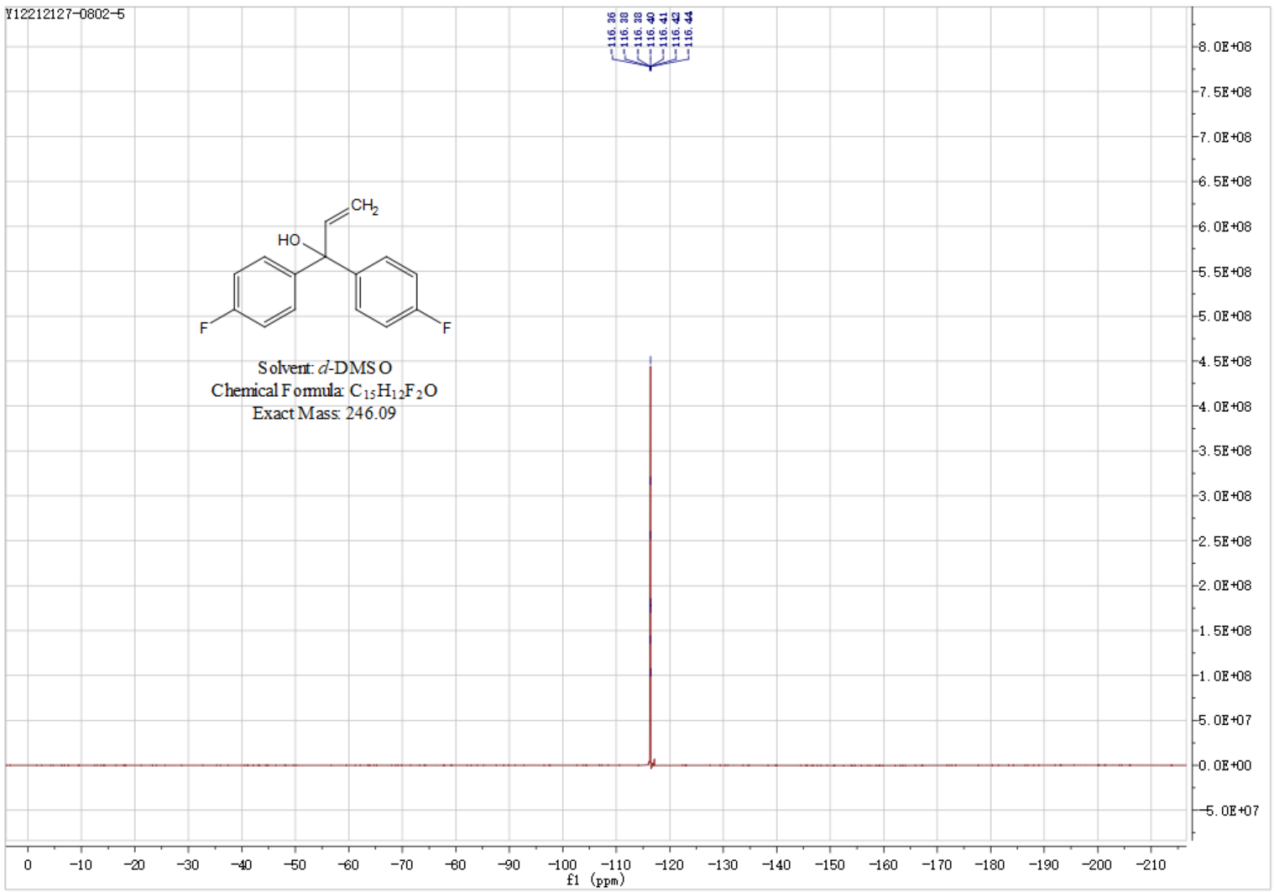


Figure S28. The ^19^F NMR of compound **2f.**


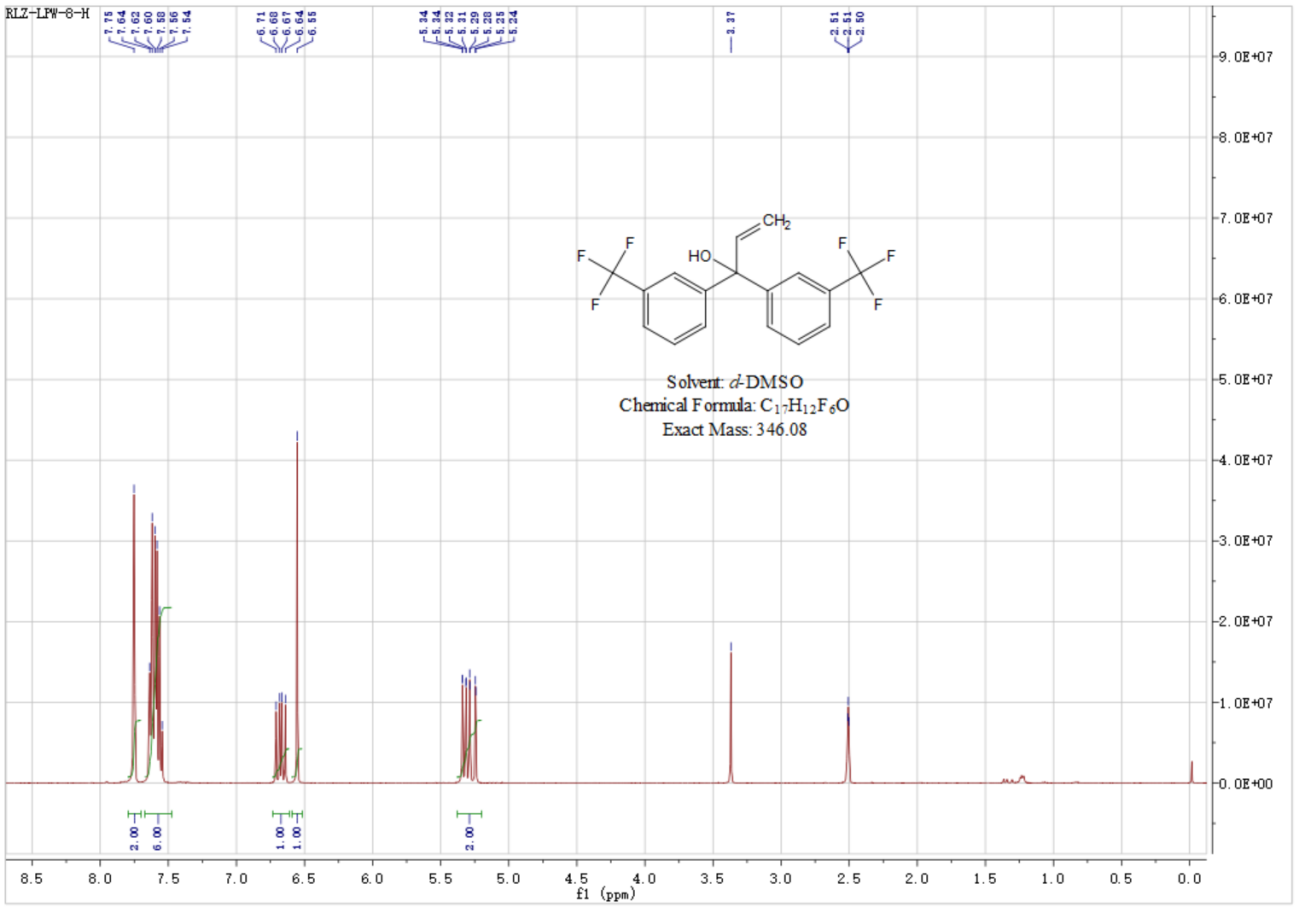


Figure S29. The ^1^H NMR of compound **2g.**


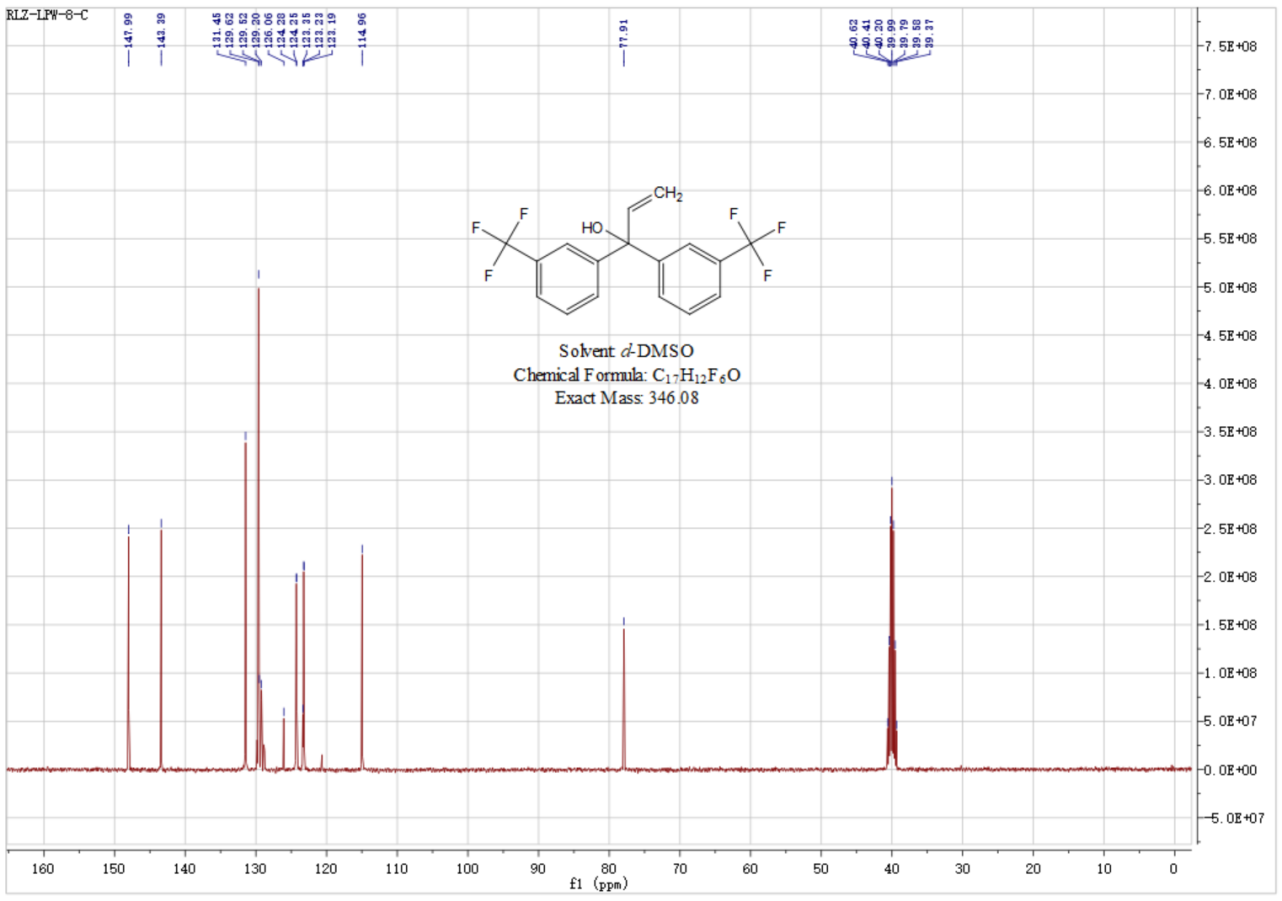


Figure S30. The ^13^C NMR of compound **2g.**


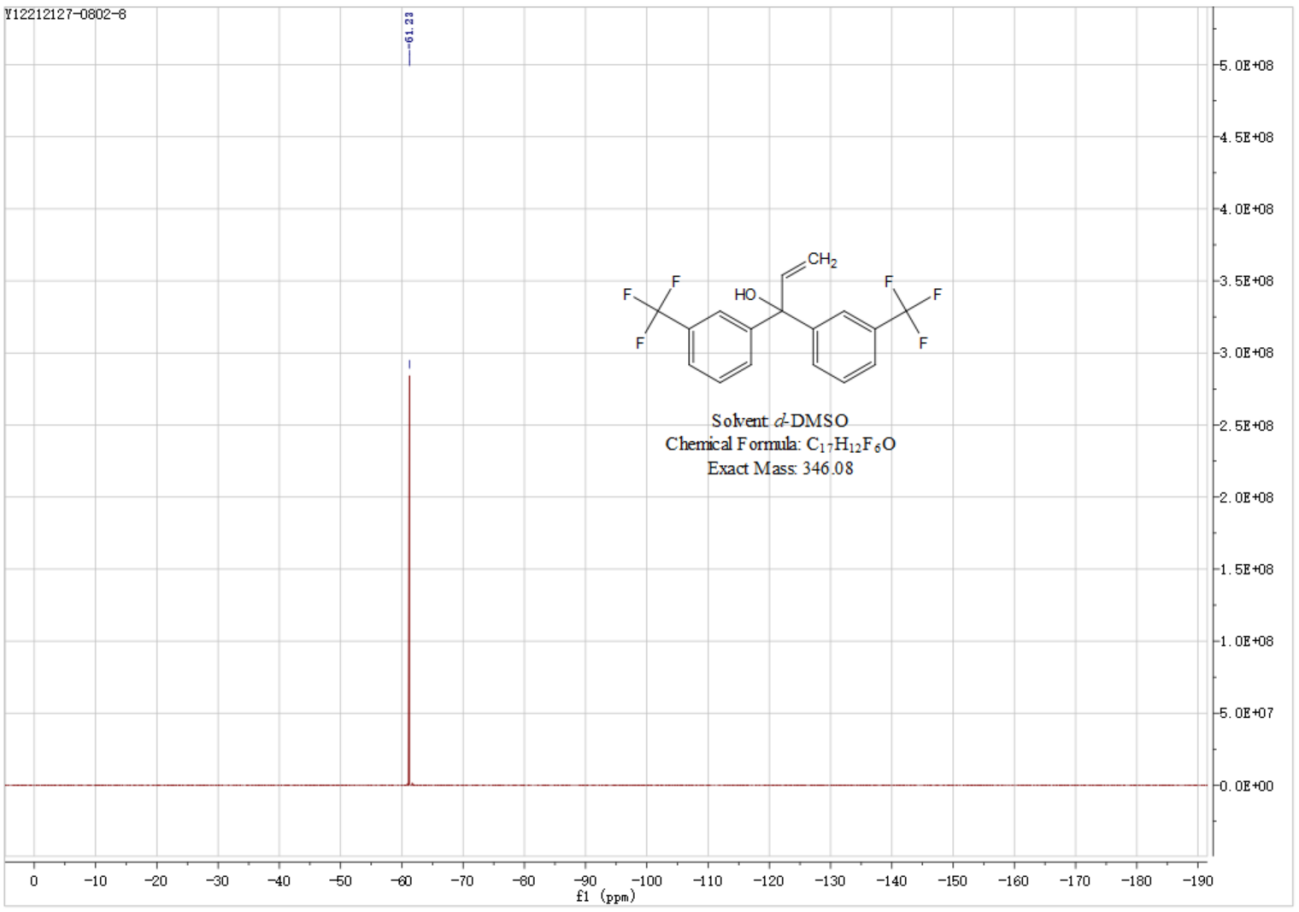


Figure S31. The ^19^F NMR of compound **2g.**


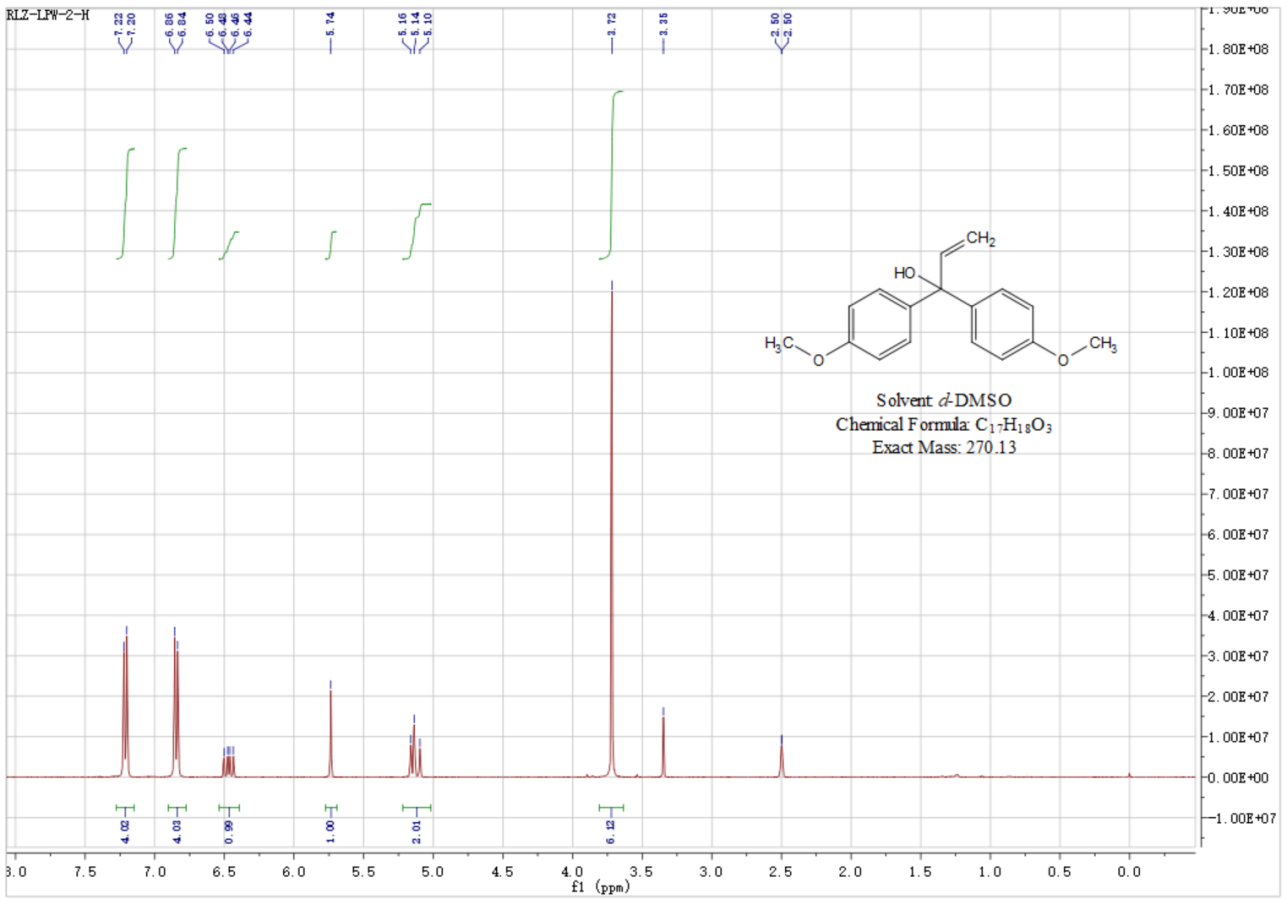


Figure S32. The ^1^H NMR of compound **2h.**


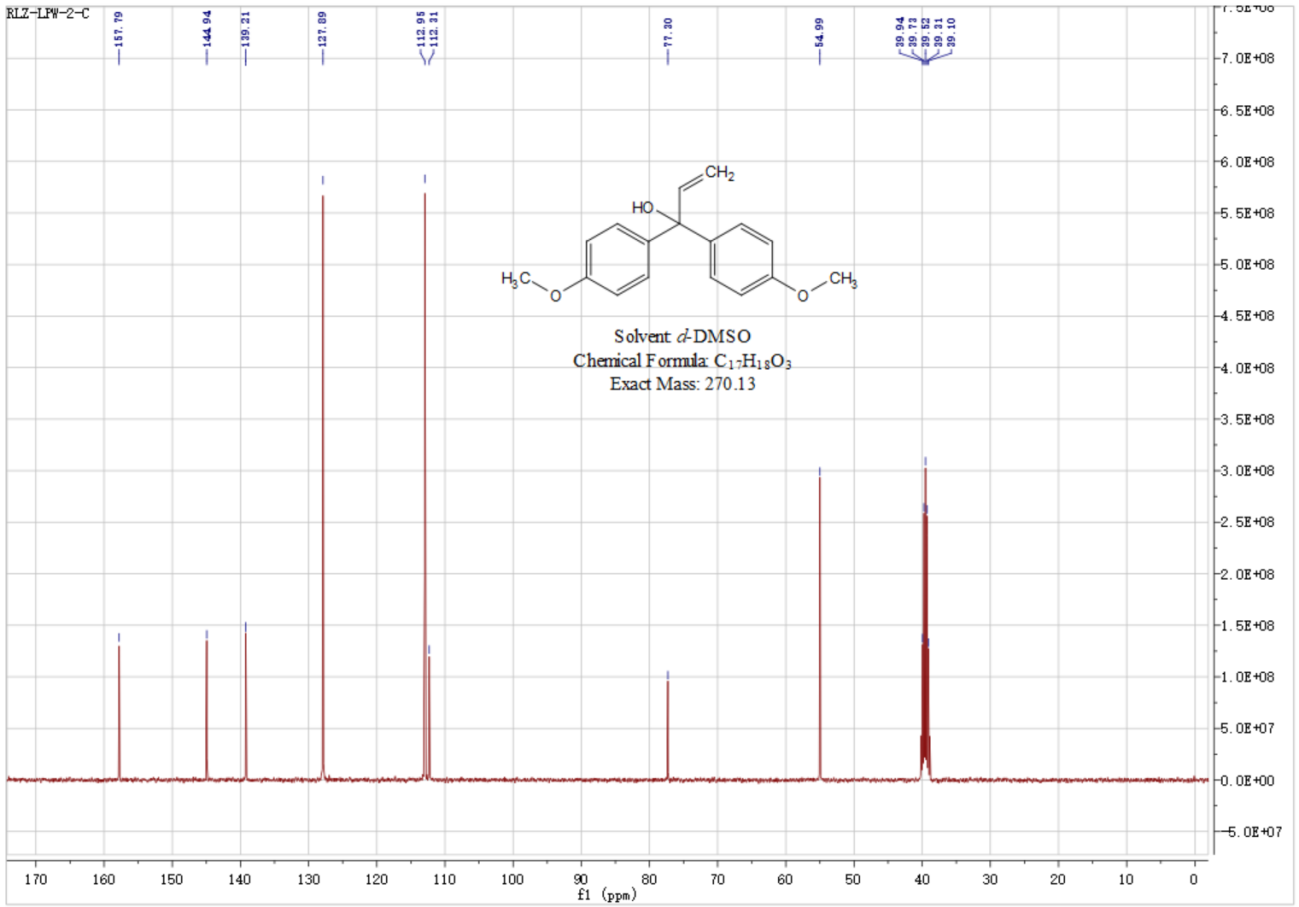


Figure S33. The ^13^C NMR of compound **2h.**


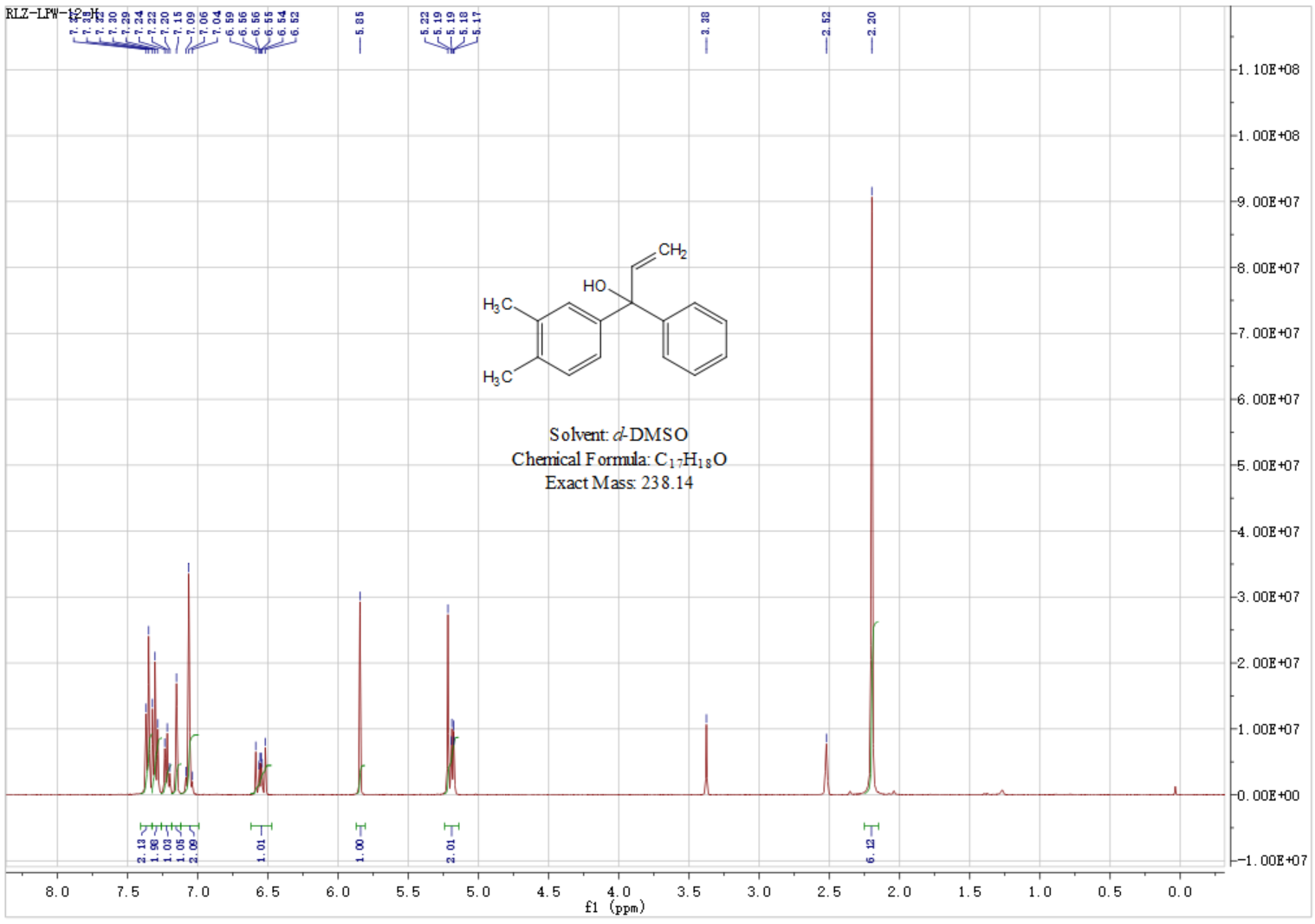


Figure S34. The ^1^H NMR of compound **2i.**


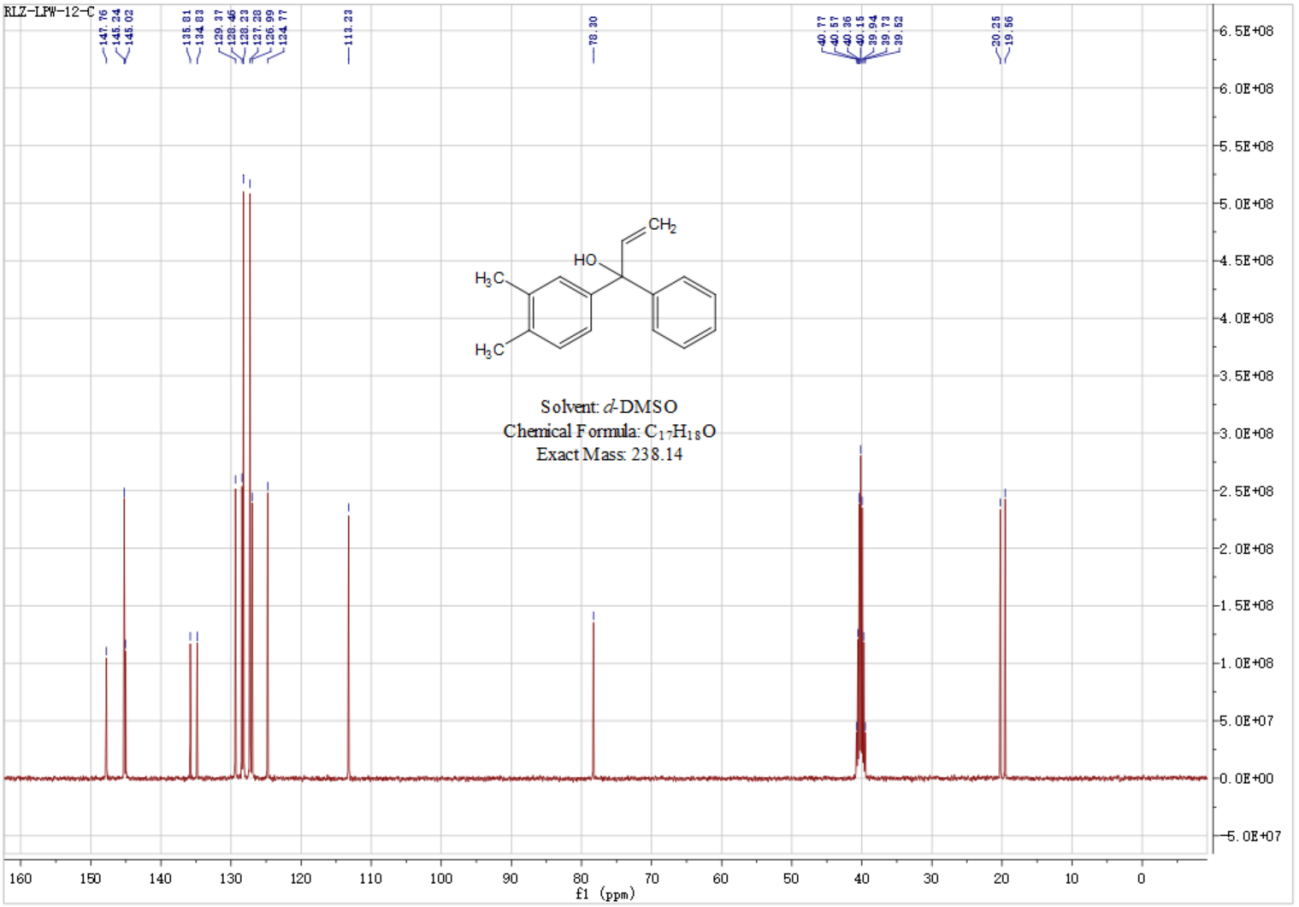


Figure S35. The ^13^C NMR of compound **2i.**


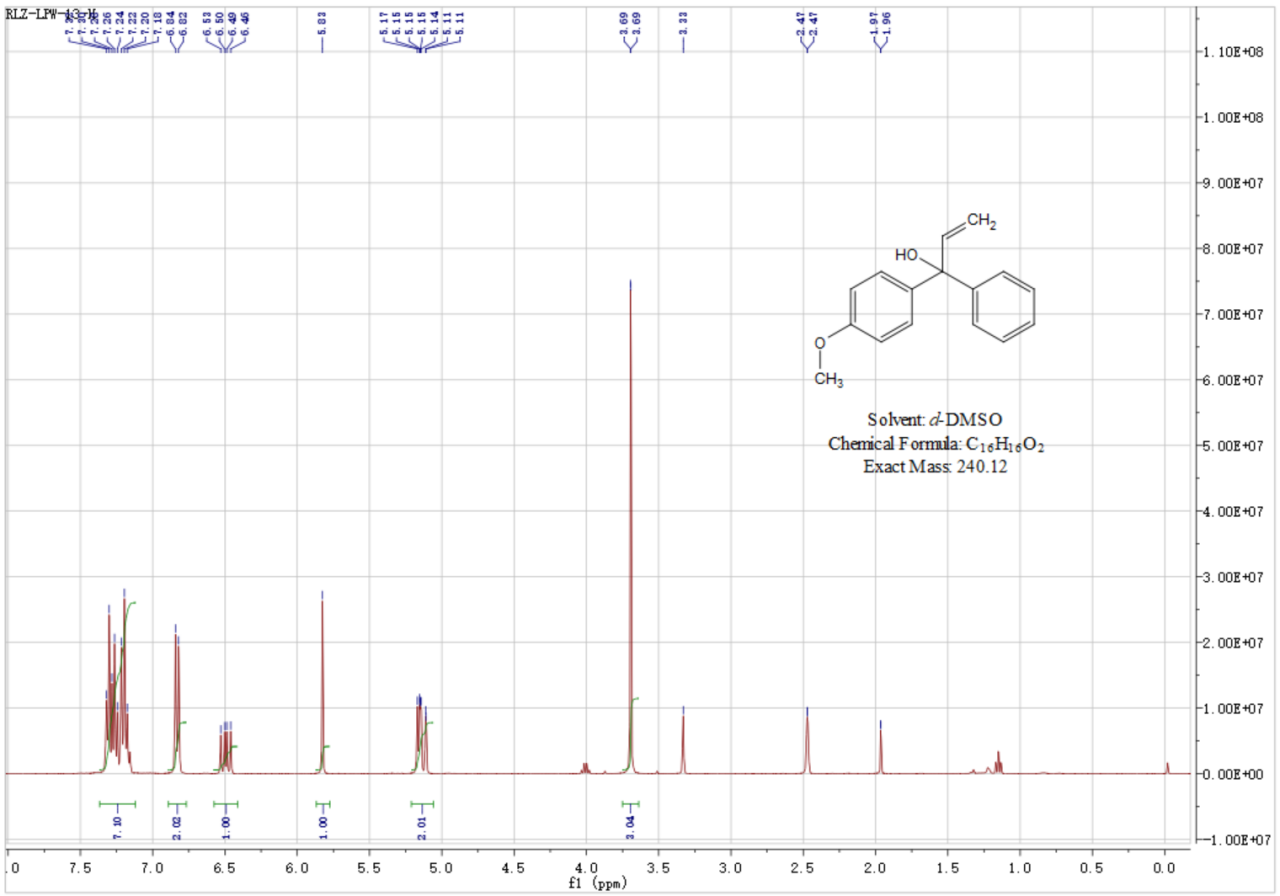


Figure S36. The ^1^H NMR of compound **2j.**


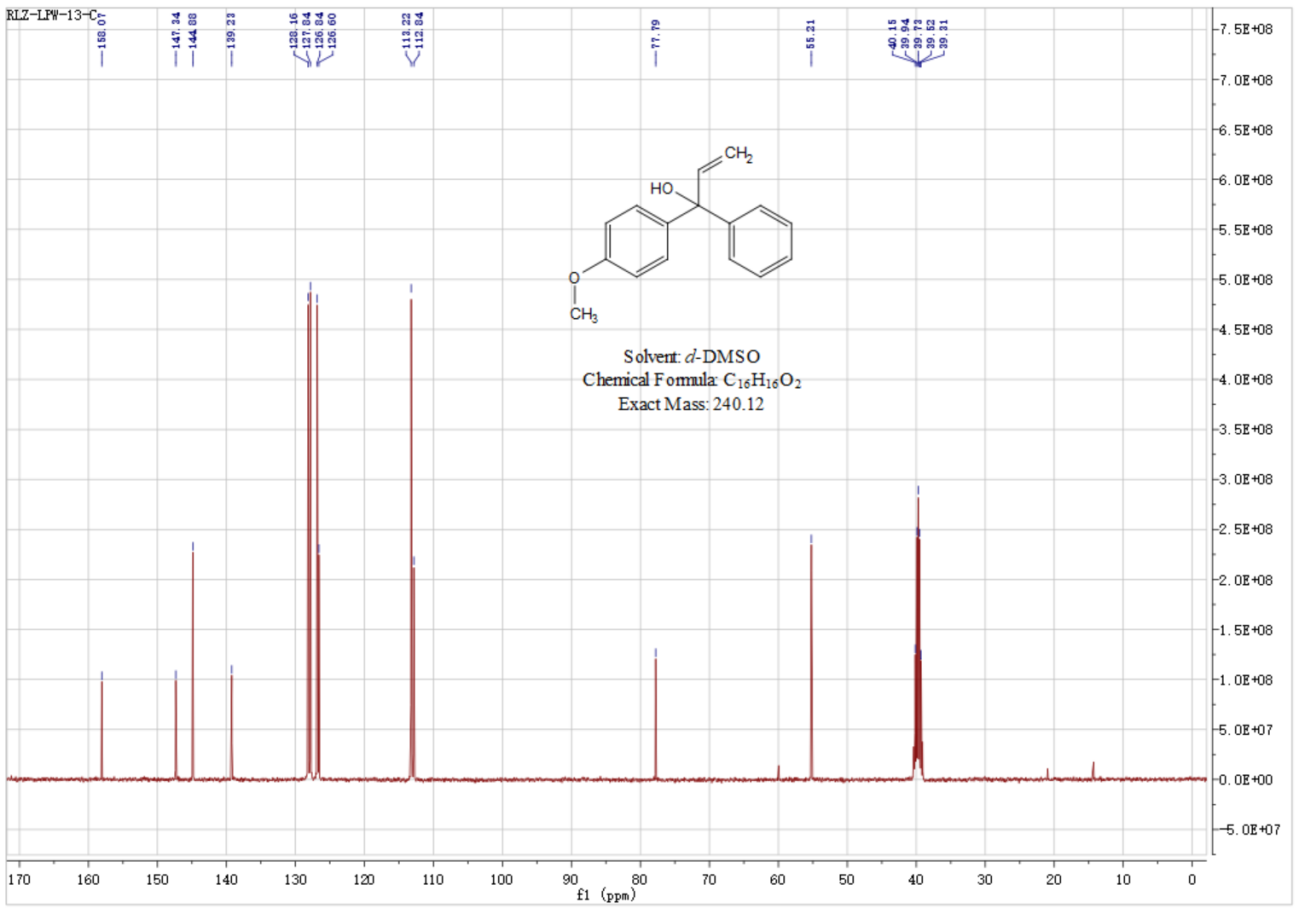


Figure S37. The ^13^C NMR of compound **2j.**


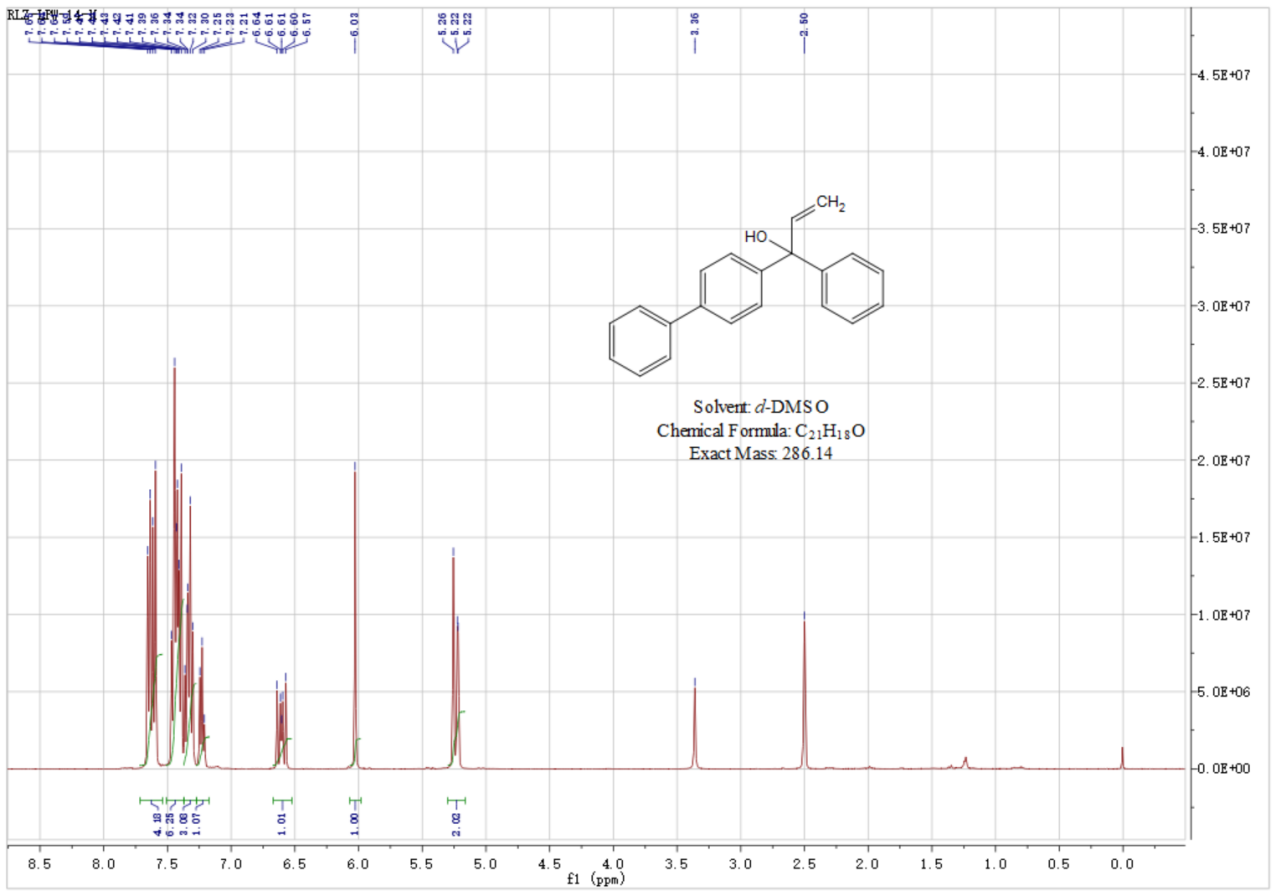


Figure S38. The ^1^H NMR of compound **2k.**


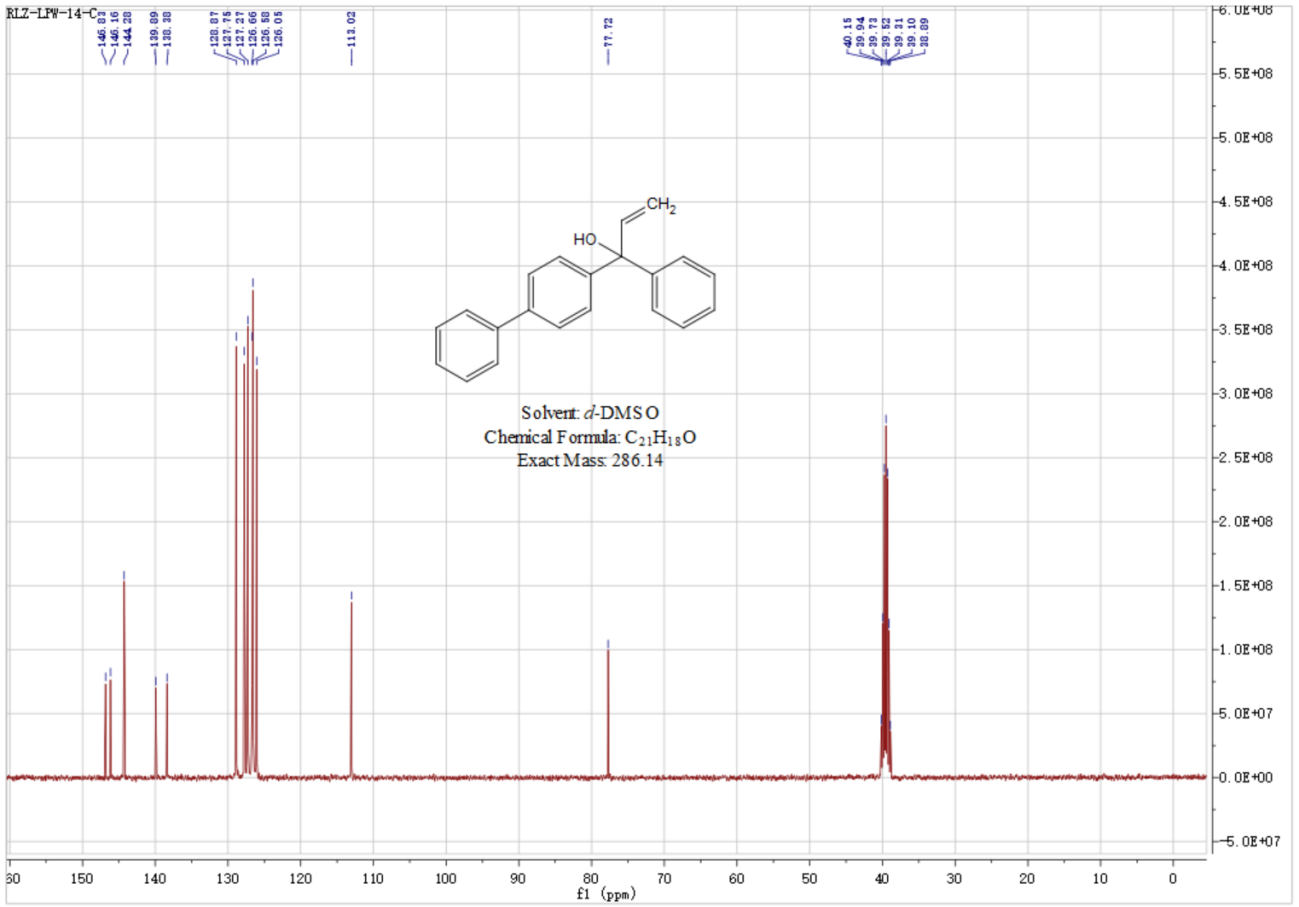


Figure S39. The ^13^C NMR of compound **2k.**


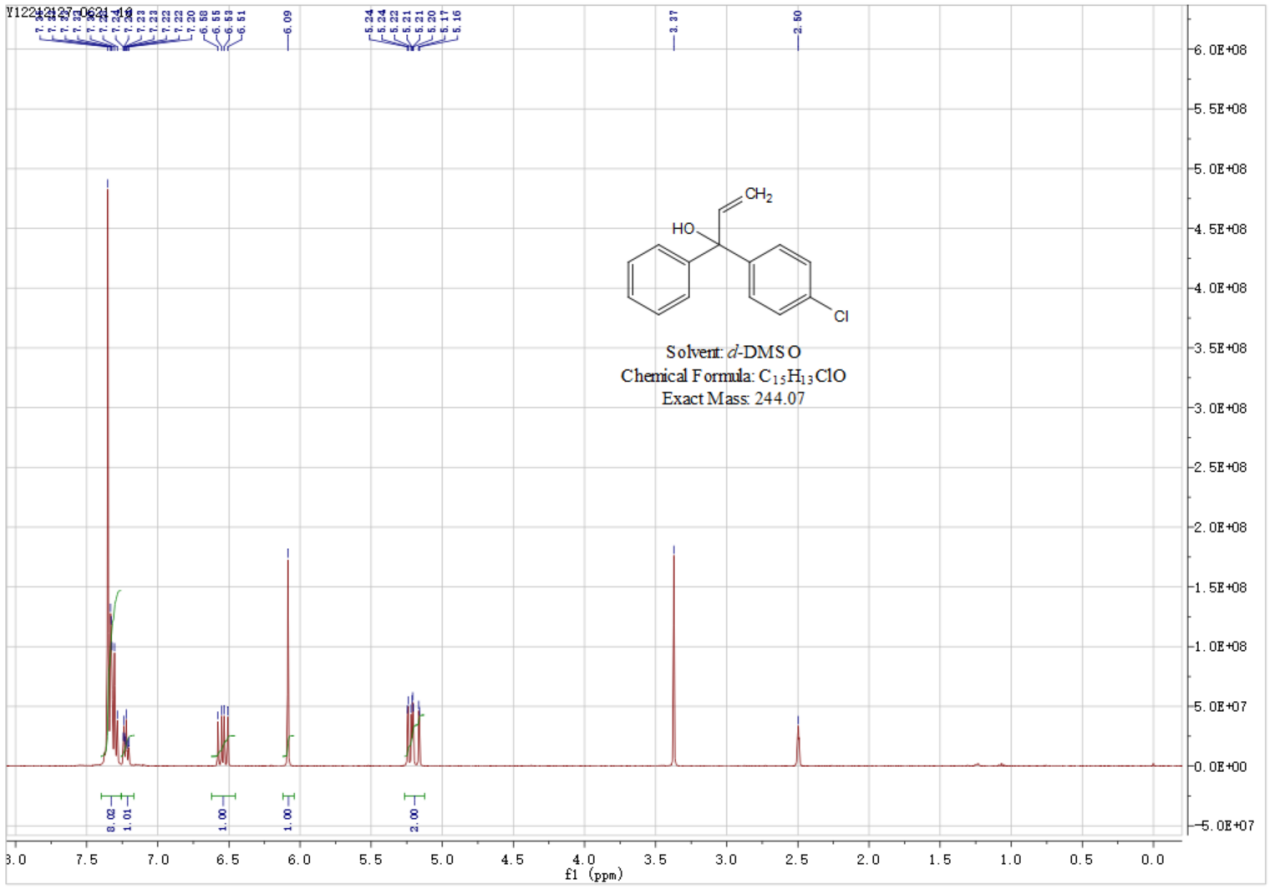


Figure S40. The ^1^H NMR of compound **2l.**


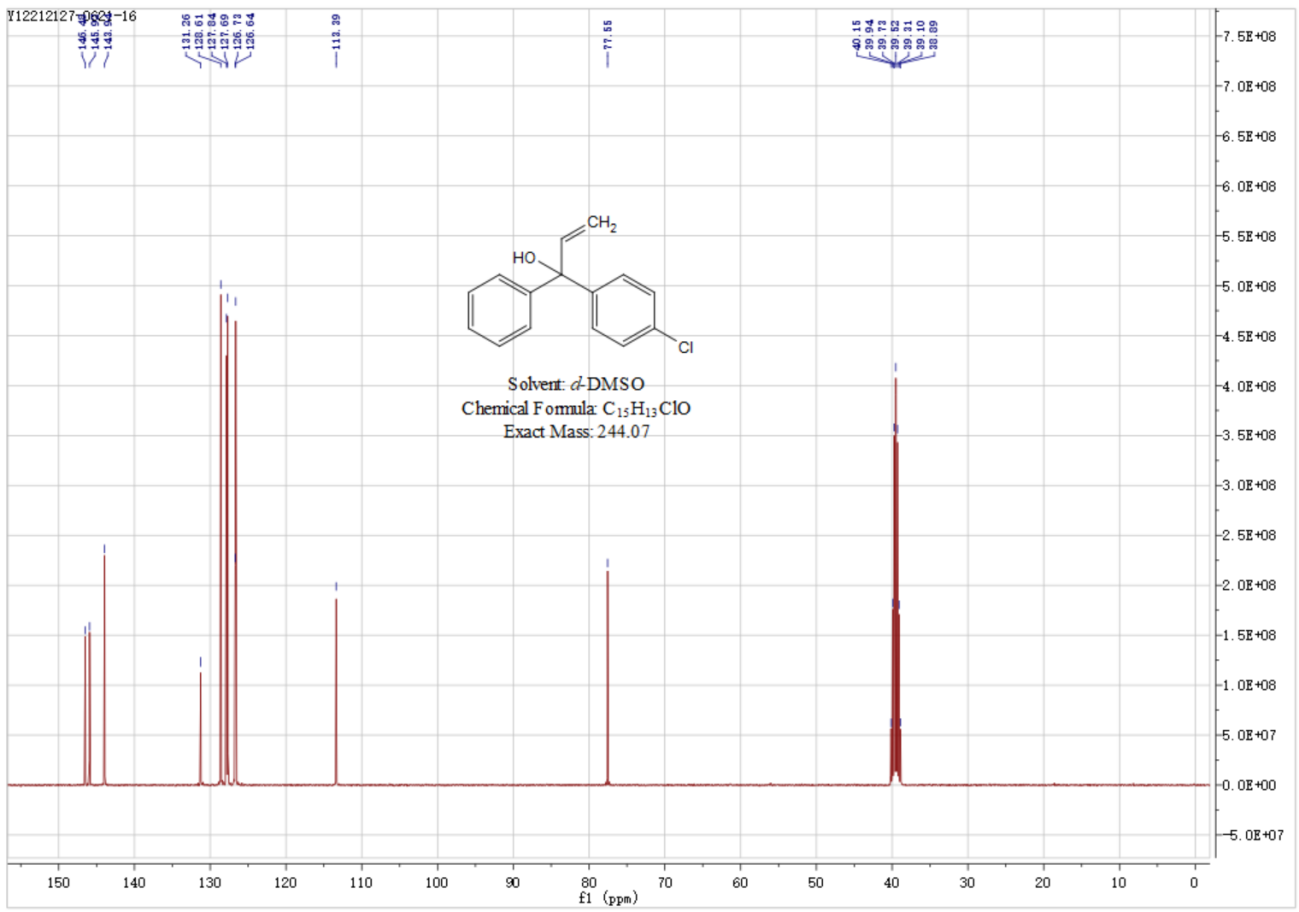


Figure S41. The ^13^C NMR of compound **2l.**


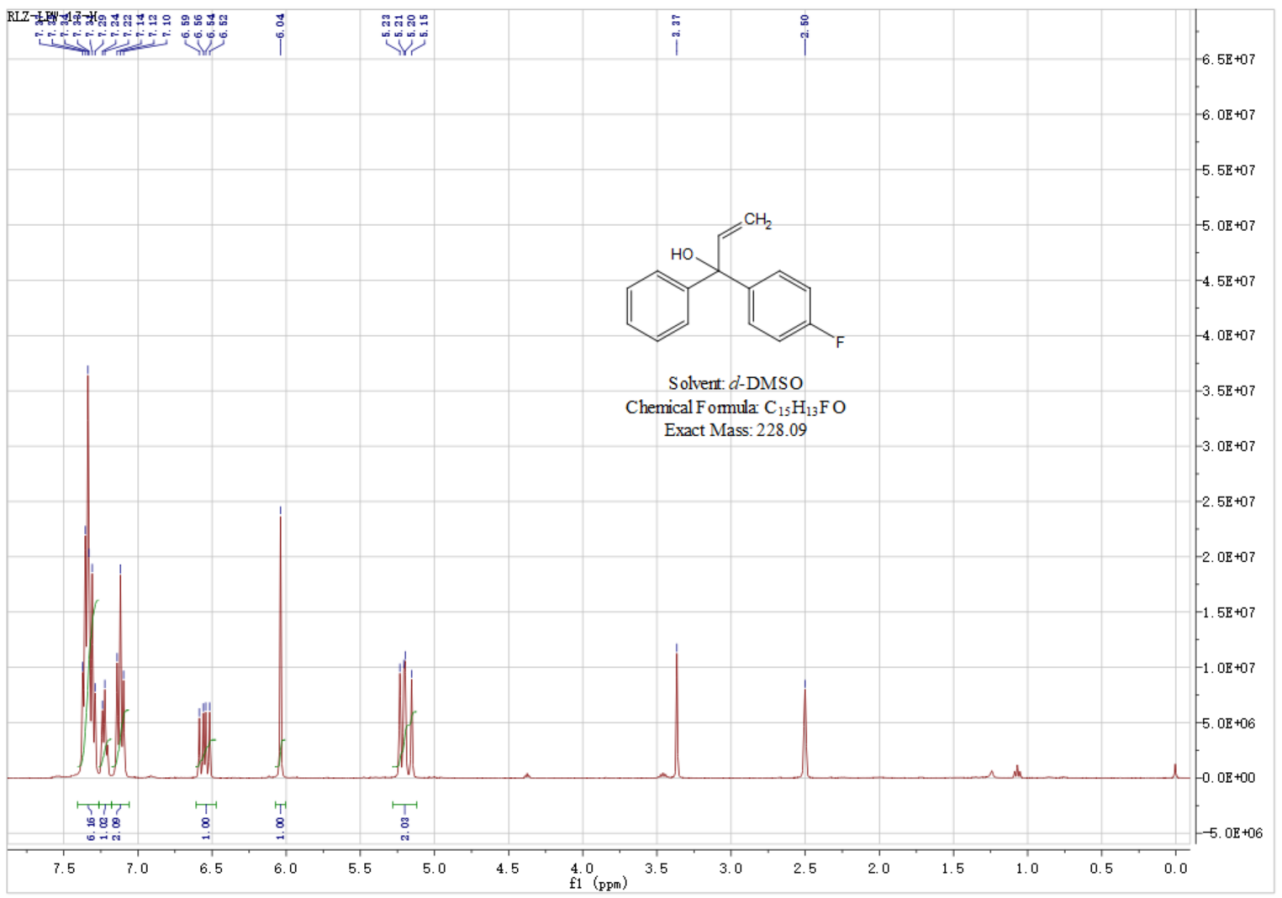


Figure S42. The ^1^H NMR of compound **2m.**


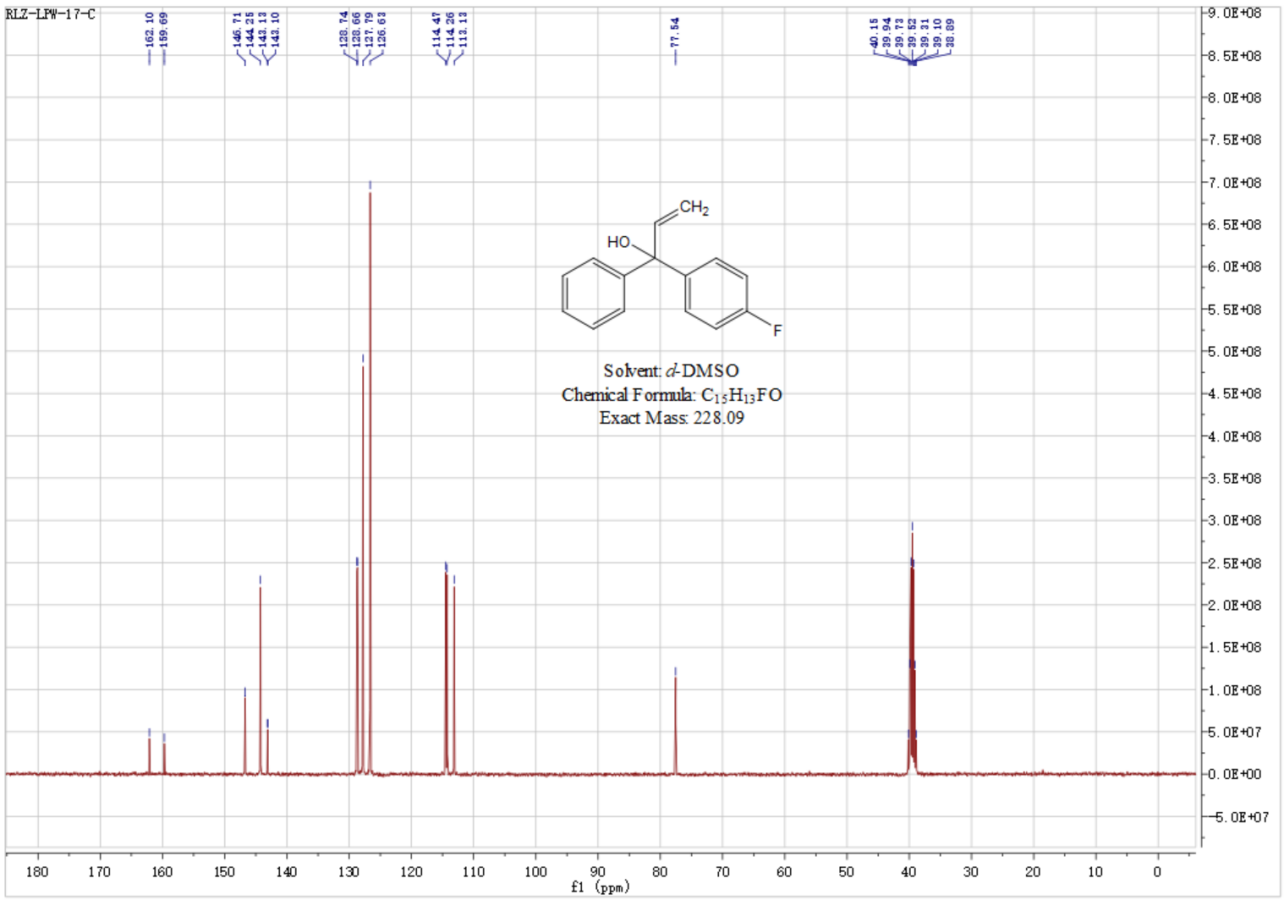


Figure S43. The ^13^C NMR of compound **2m.**


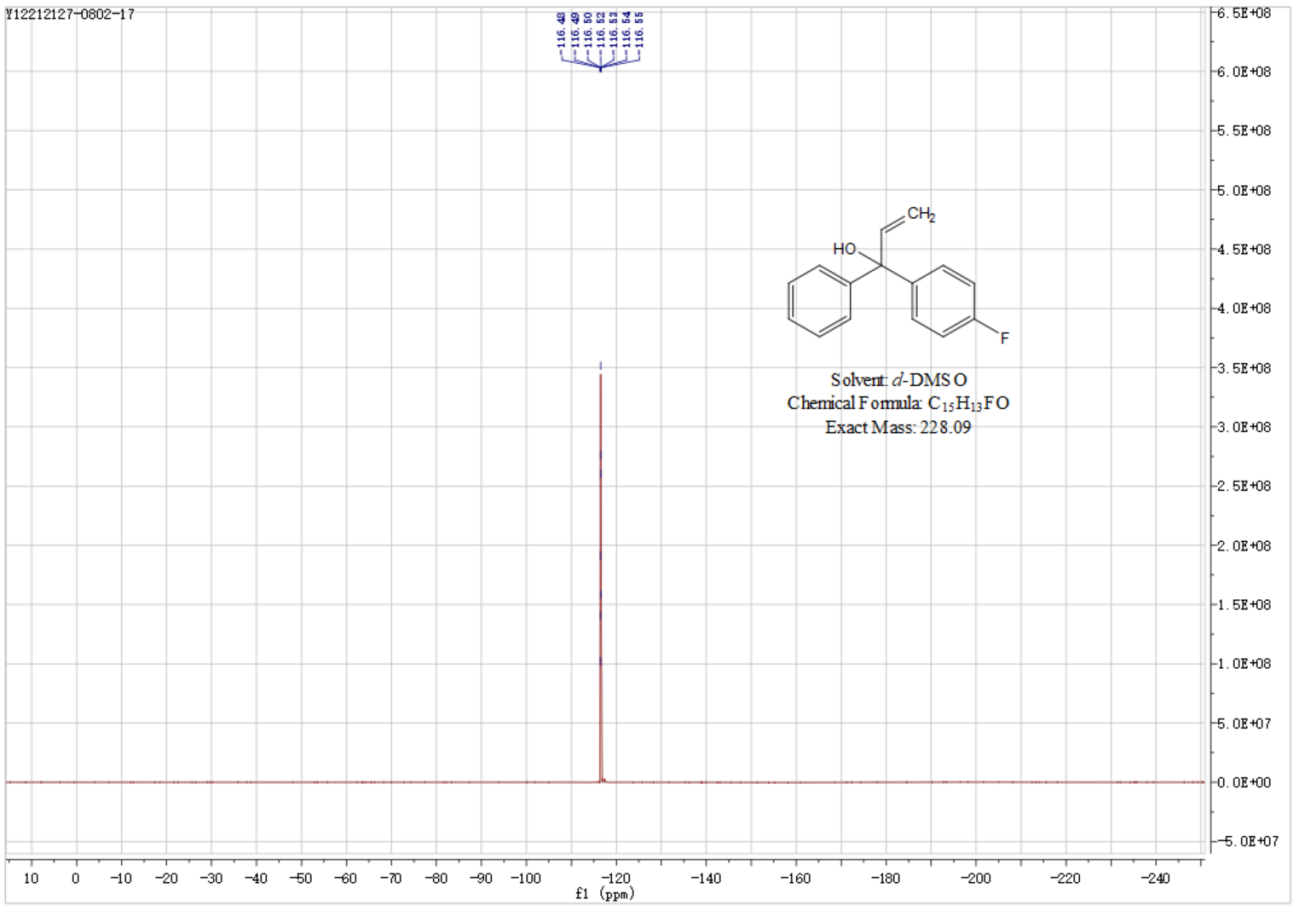


Figure S44. The ^19^F NMR of compound **2m.**


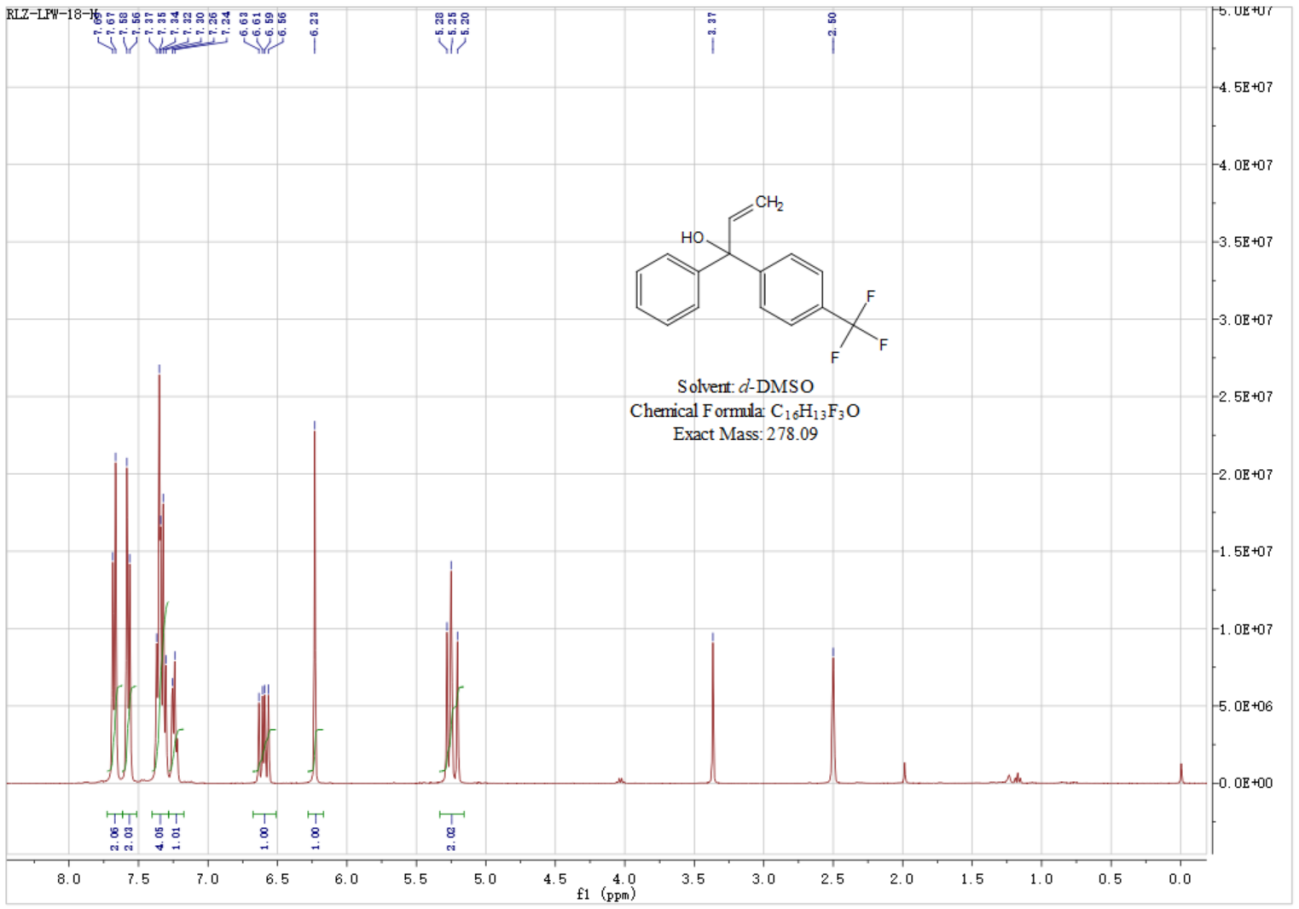


Figure S45. The ^1^H NMR of compound **2n.**


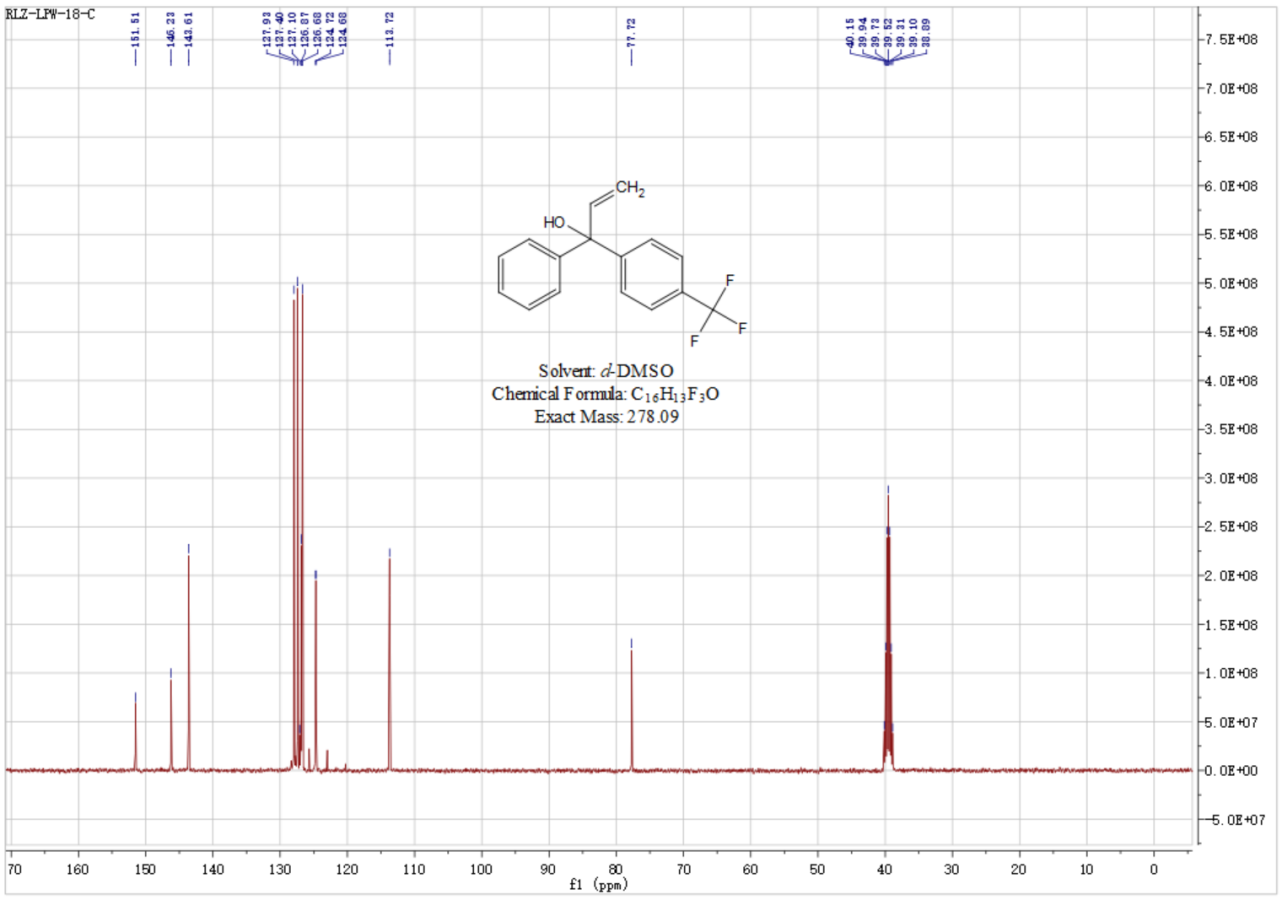


Figure S46. The ^13^C NMR of compound **2n.**


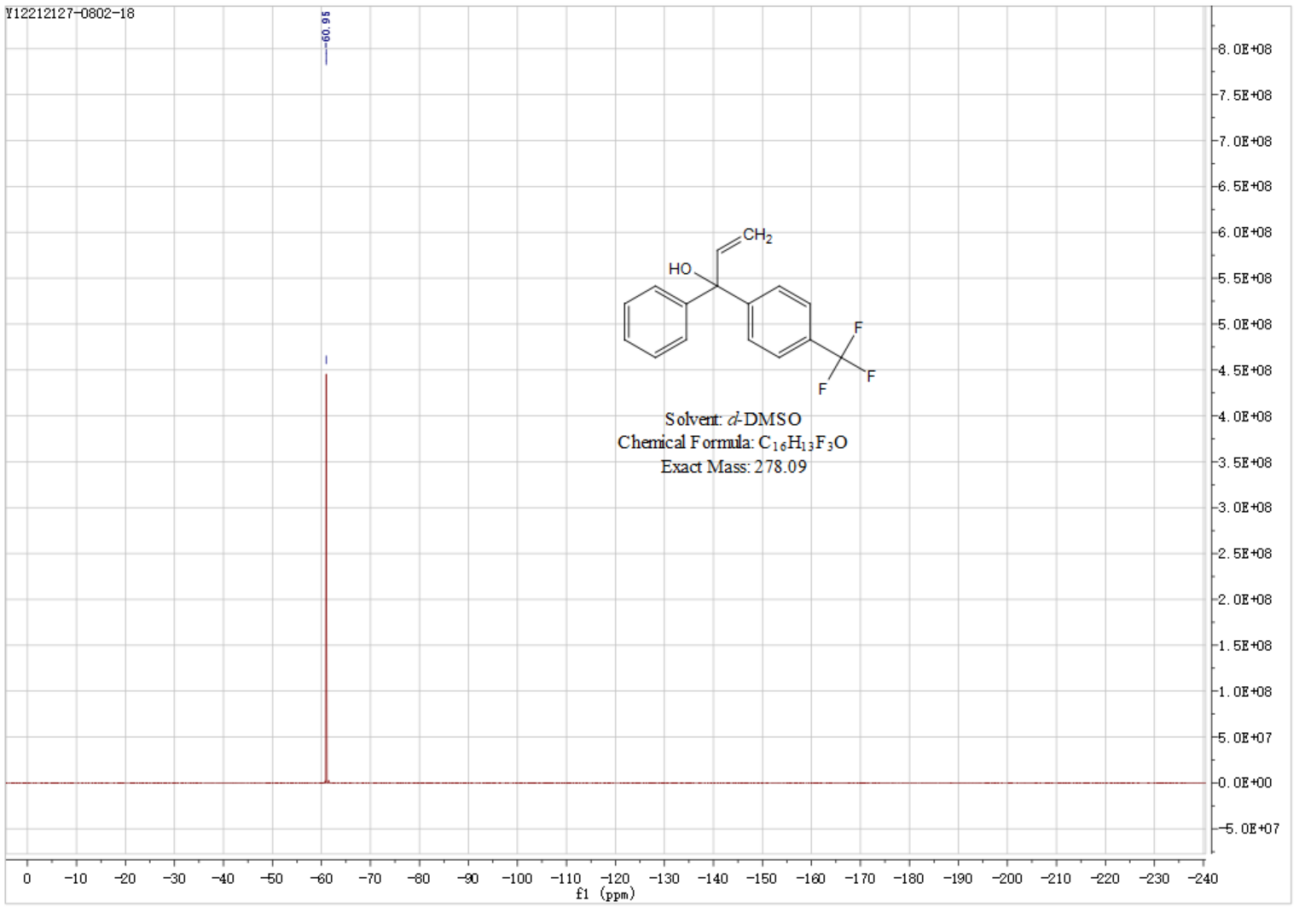


Figure S47. The ^19^F NMR of compound **2n.**


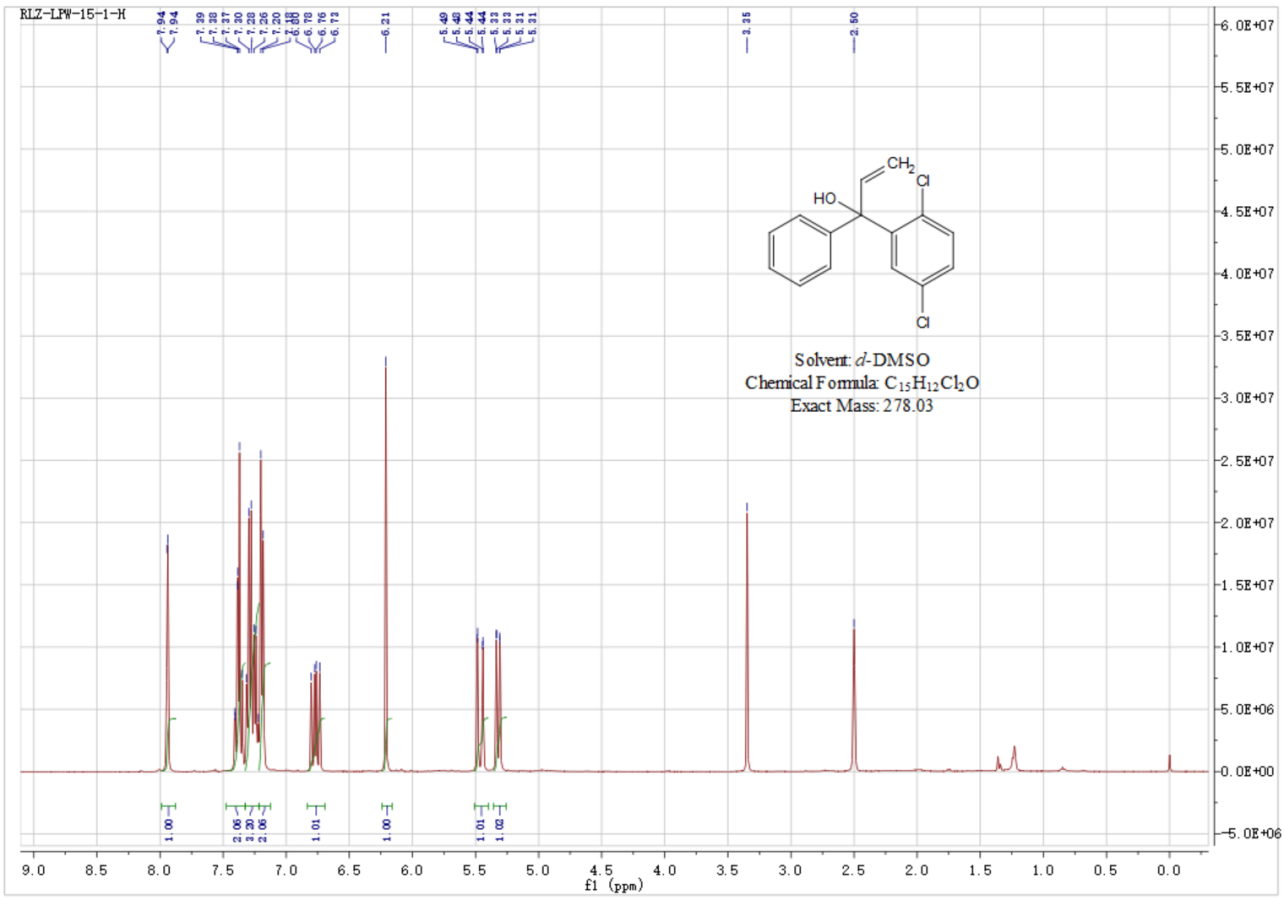


S48. The ^1^H NMR of compound **2o.**


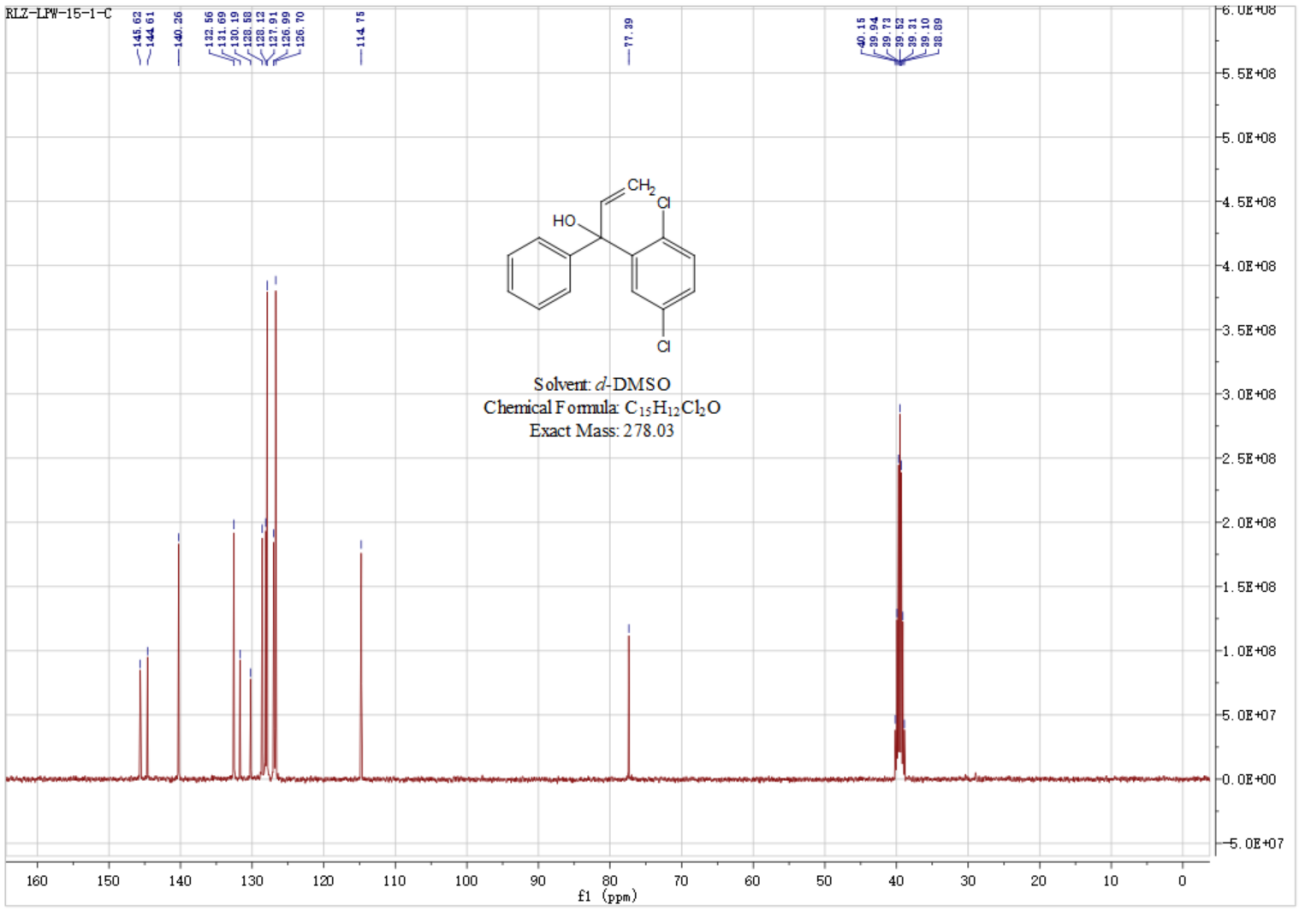


Figure S49. The ^13^C NMR of compound **2o.**


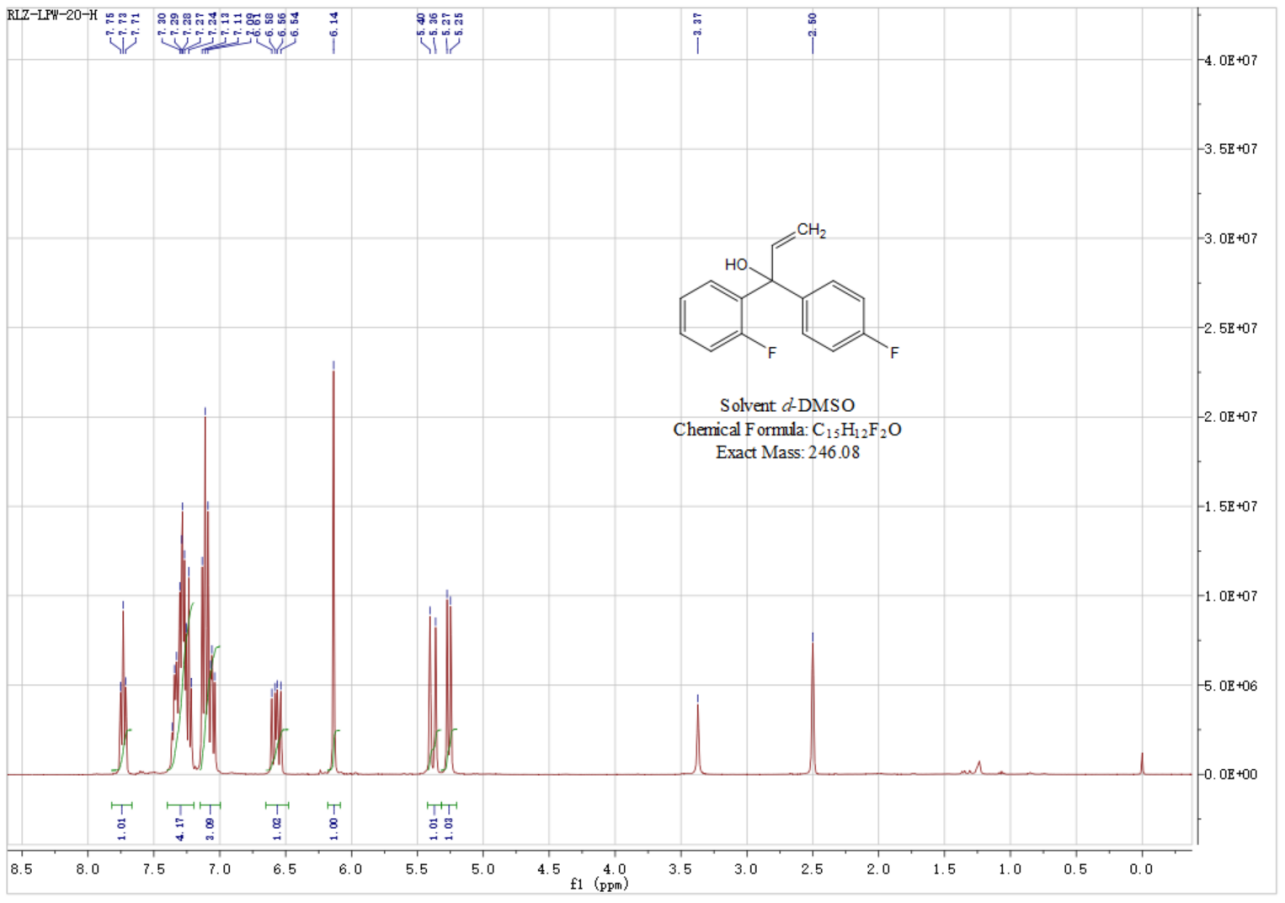


Figure S50. The ^1^H NMR of compound **2p.**


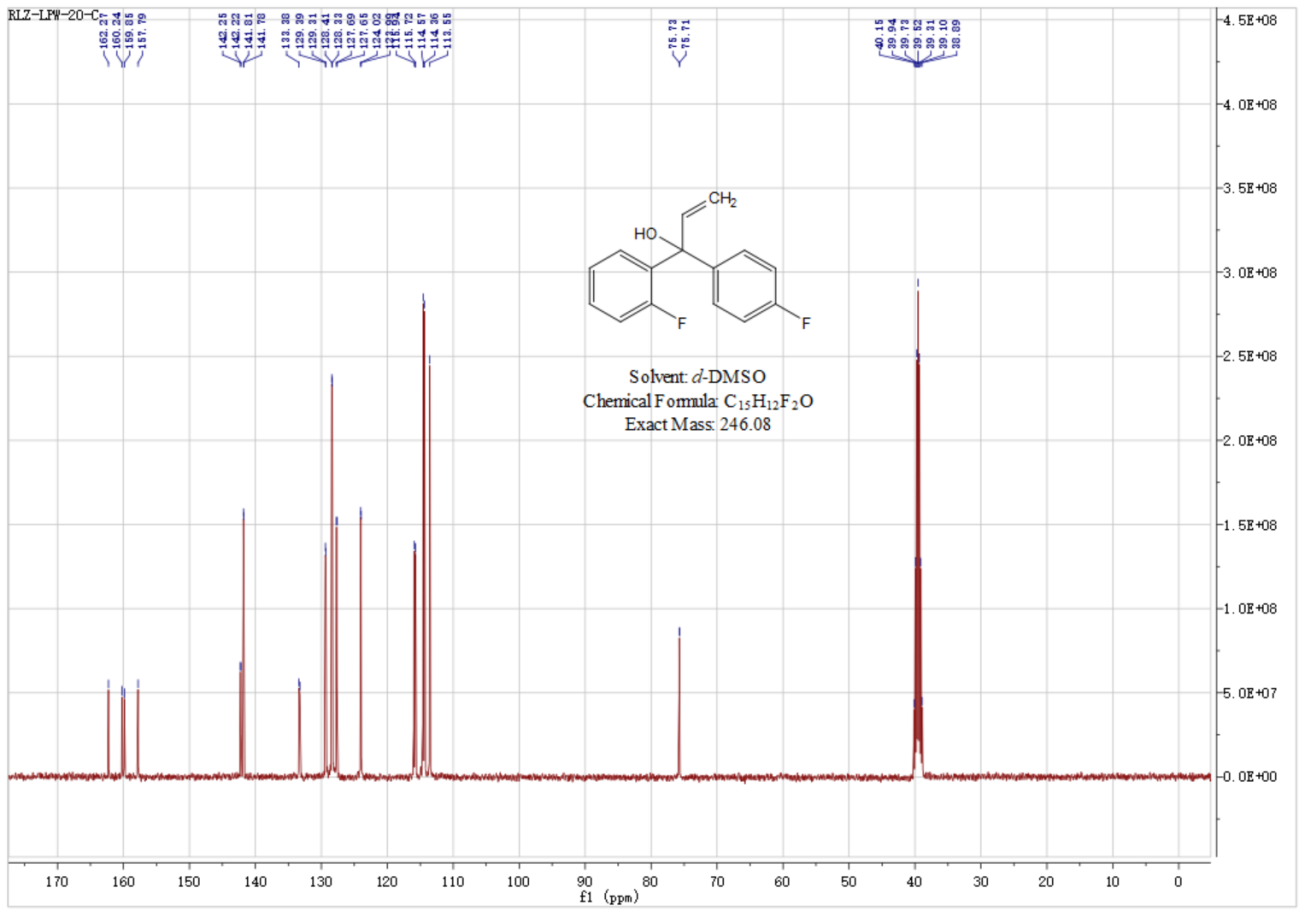


Figure S51. The ^13^C NMR of compound **2p.**


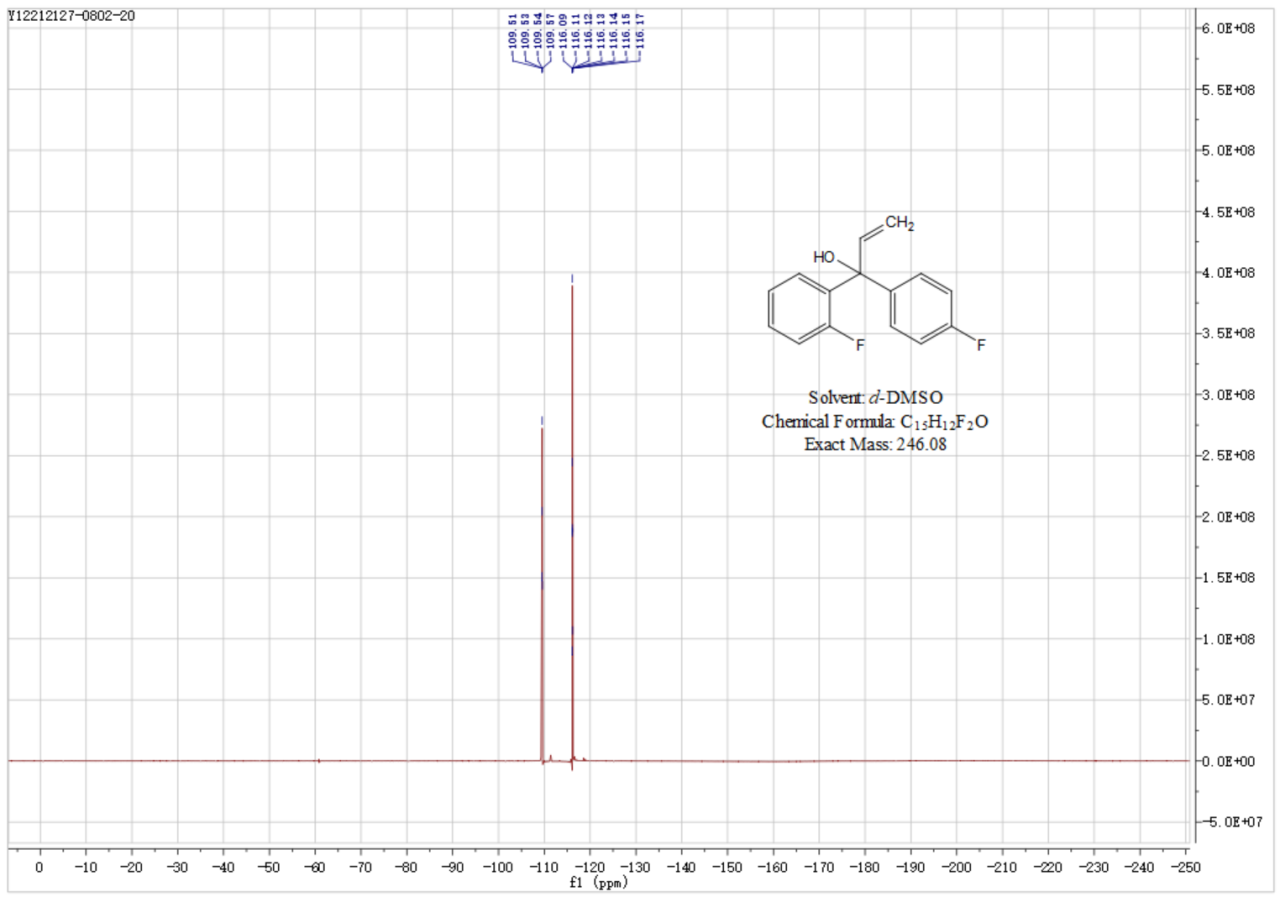


Figure S52. The ^19^F NMR of compound **2p.**


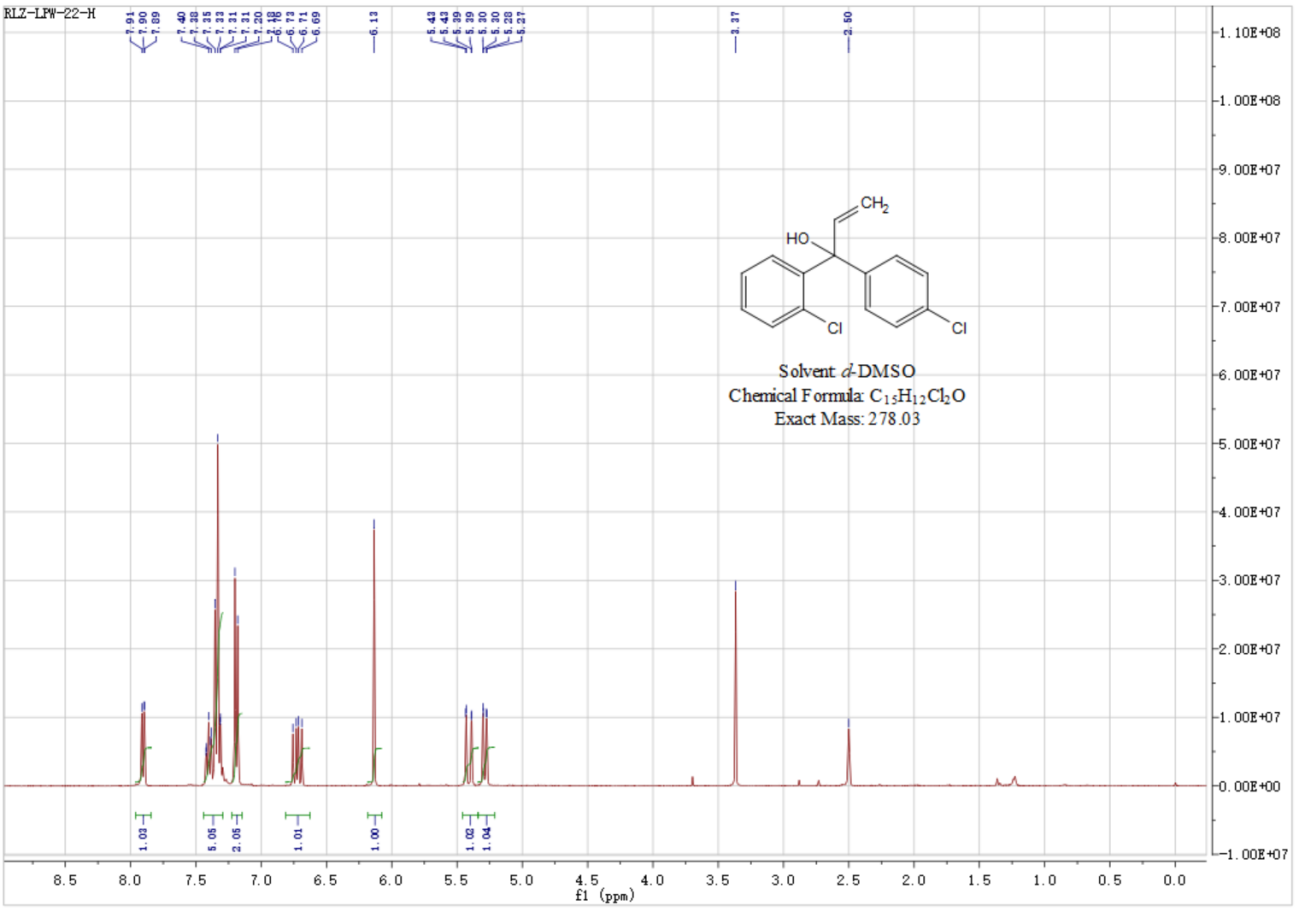


Figure S53. The ^1^H NMR of compound **2q.**


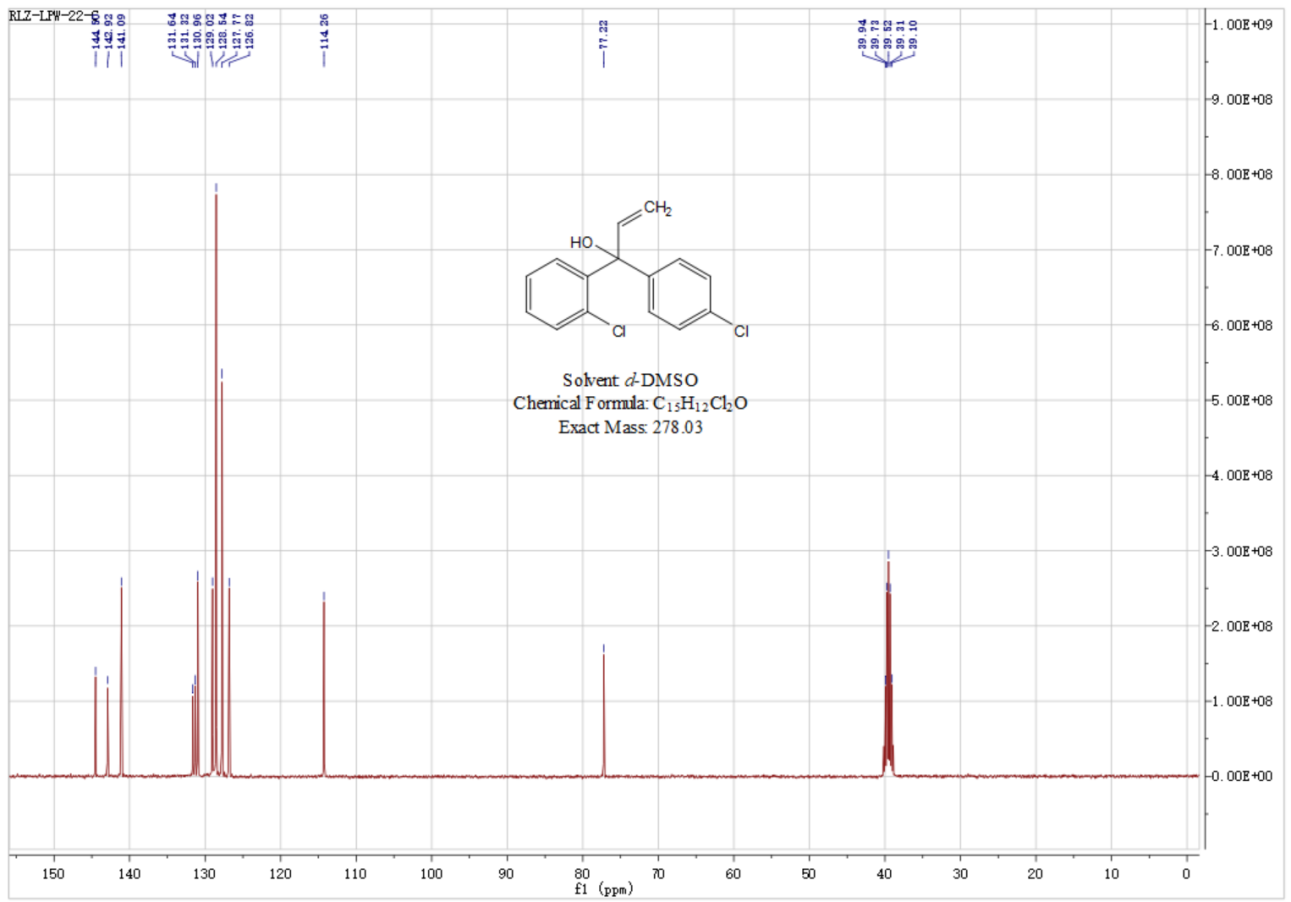


Figure S54. The ^13^C NMR of compound **2q.**


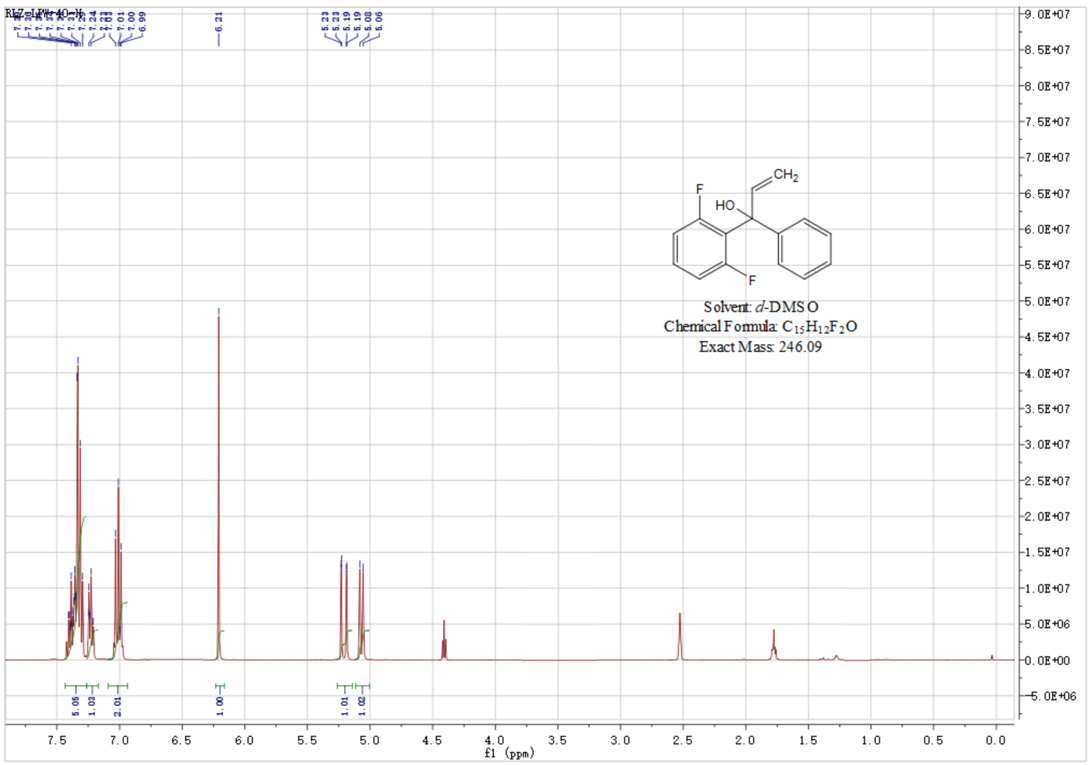


Figure S55. The ^1^H NMR of compound **2r.**


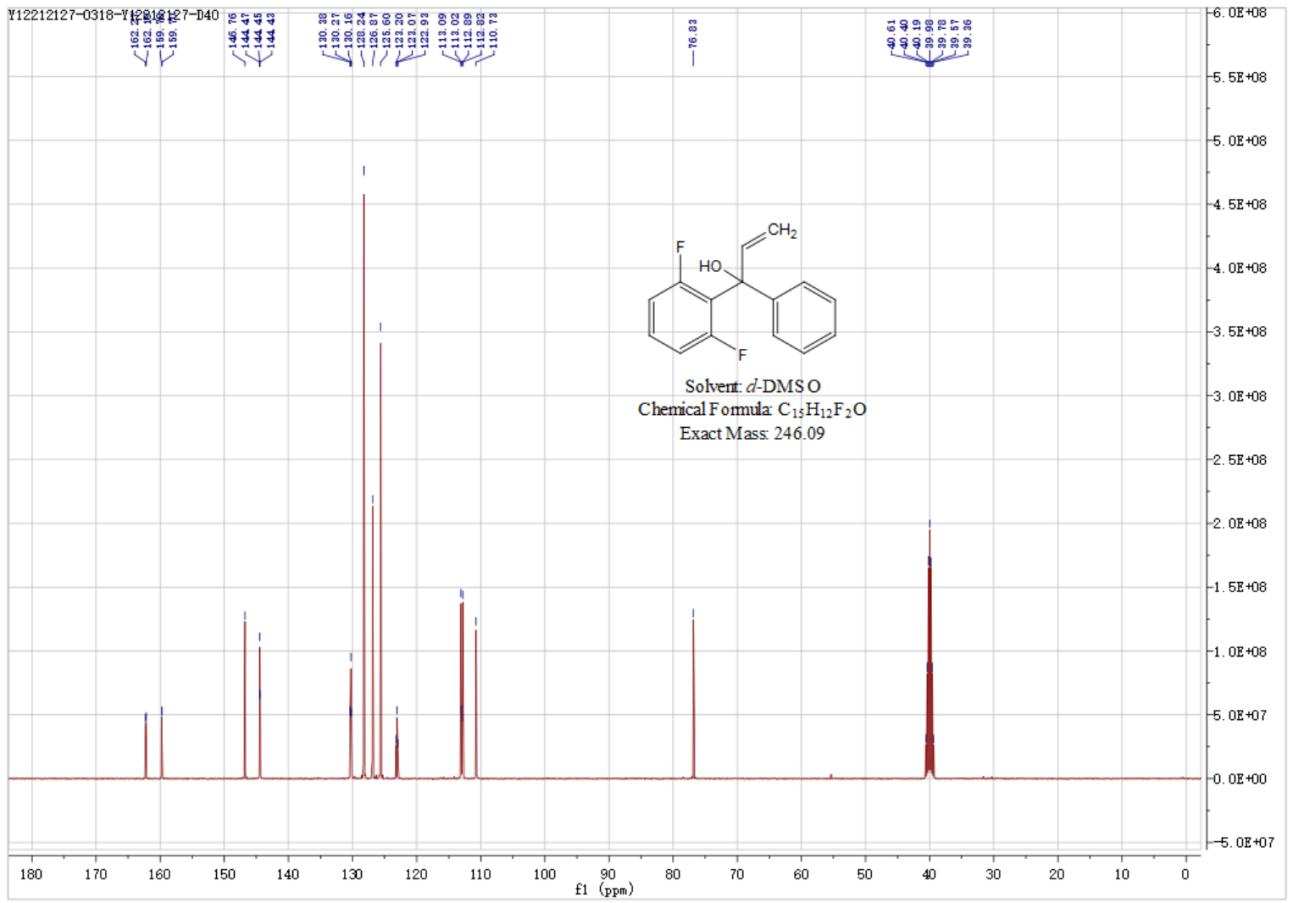


Figure S56. The ^13^C NMR of compound **2r.**


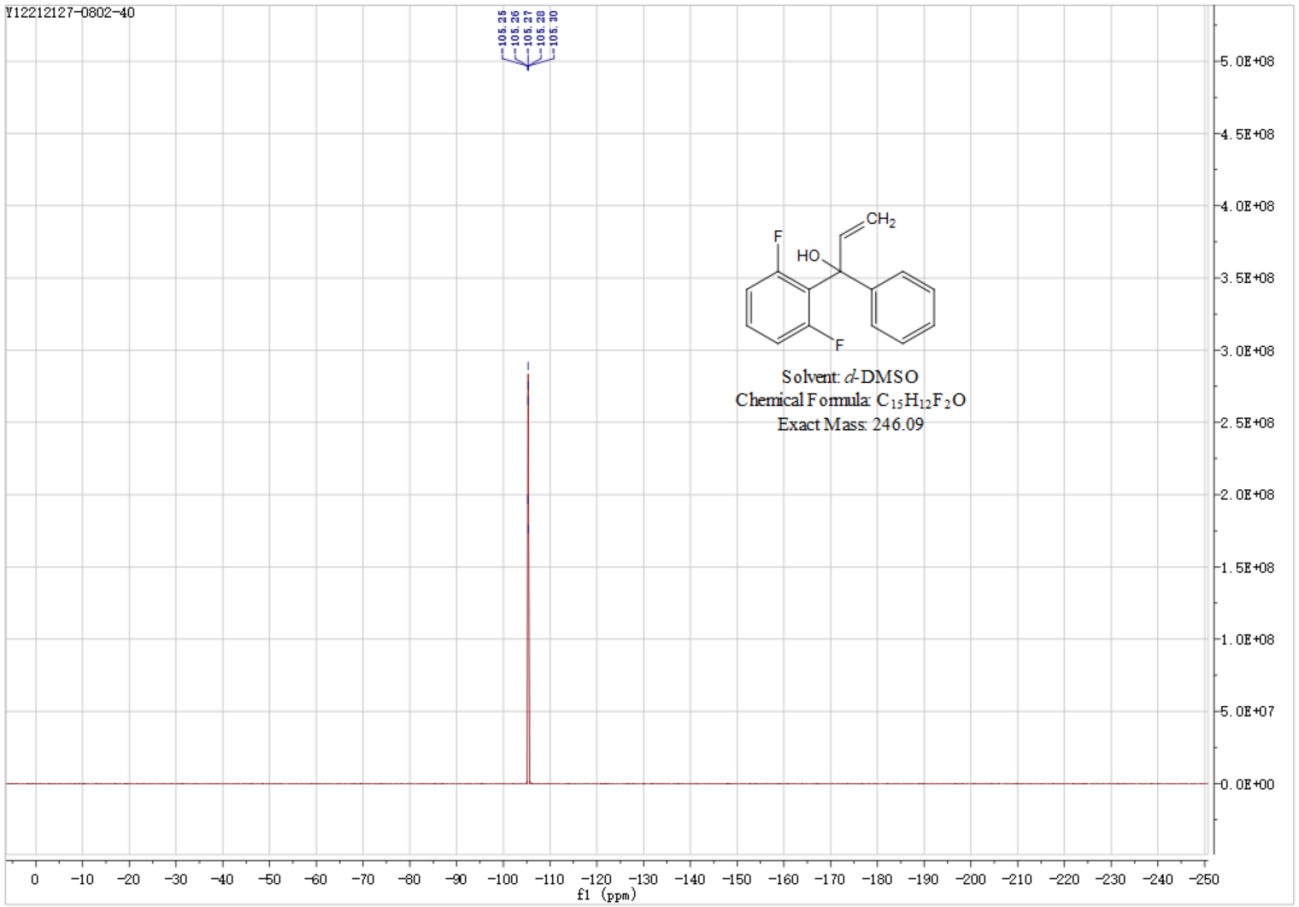


Figure S57. The ^19^F NMR of compound **2r.**


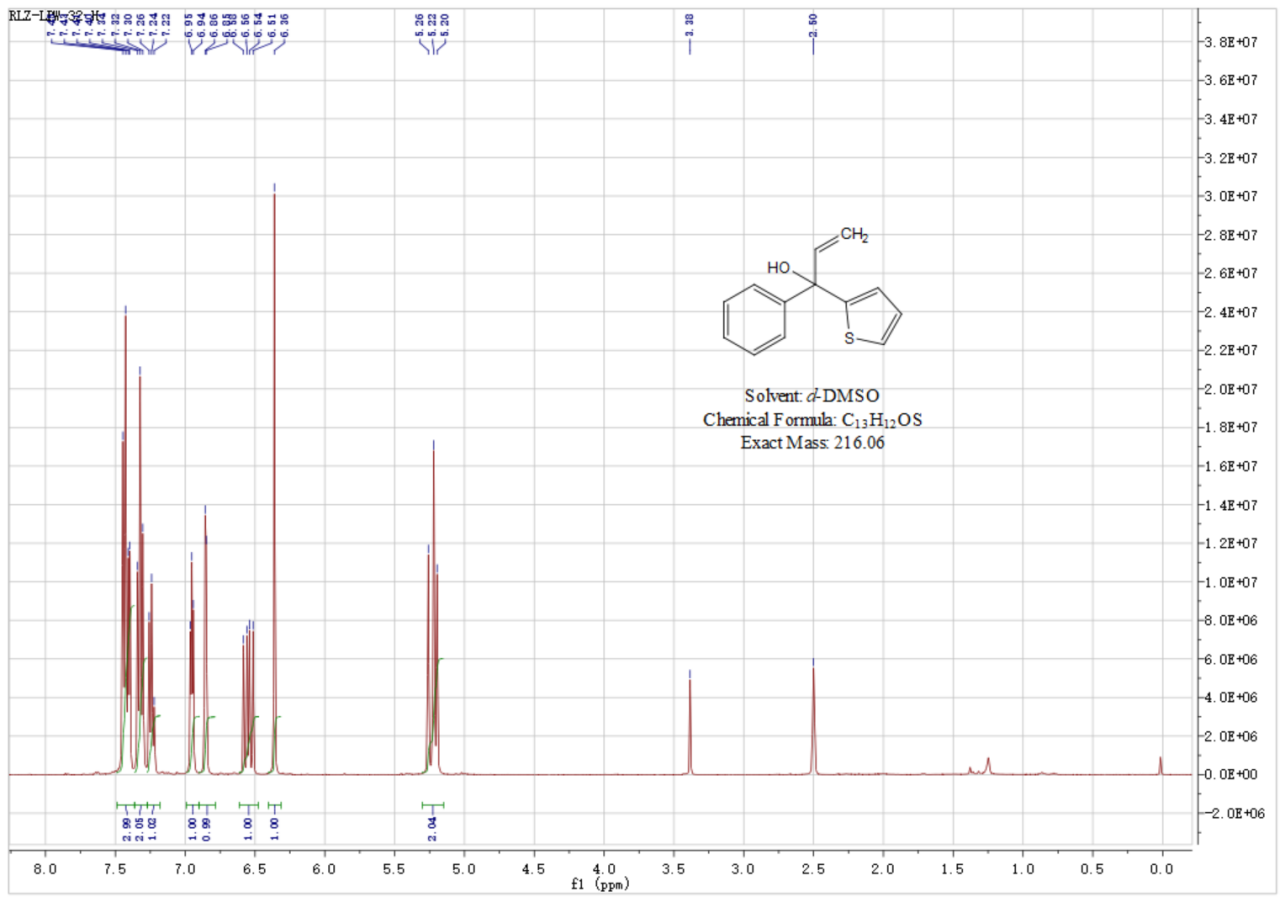


Figure S58. The ^1^H NMR of compound **2s.**


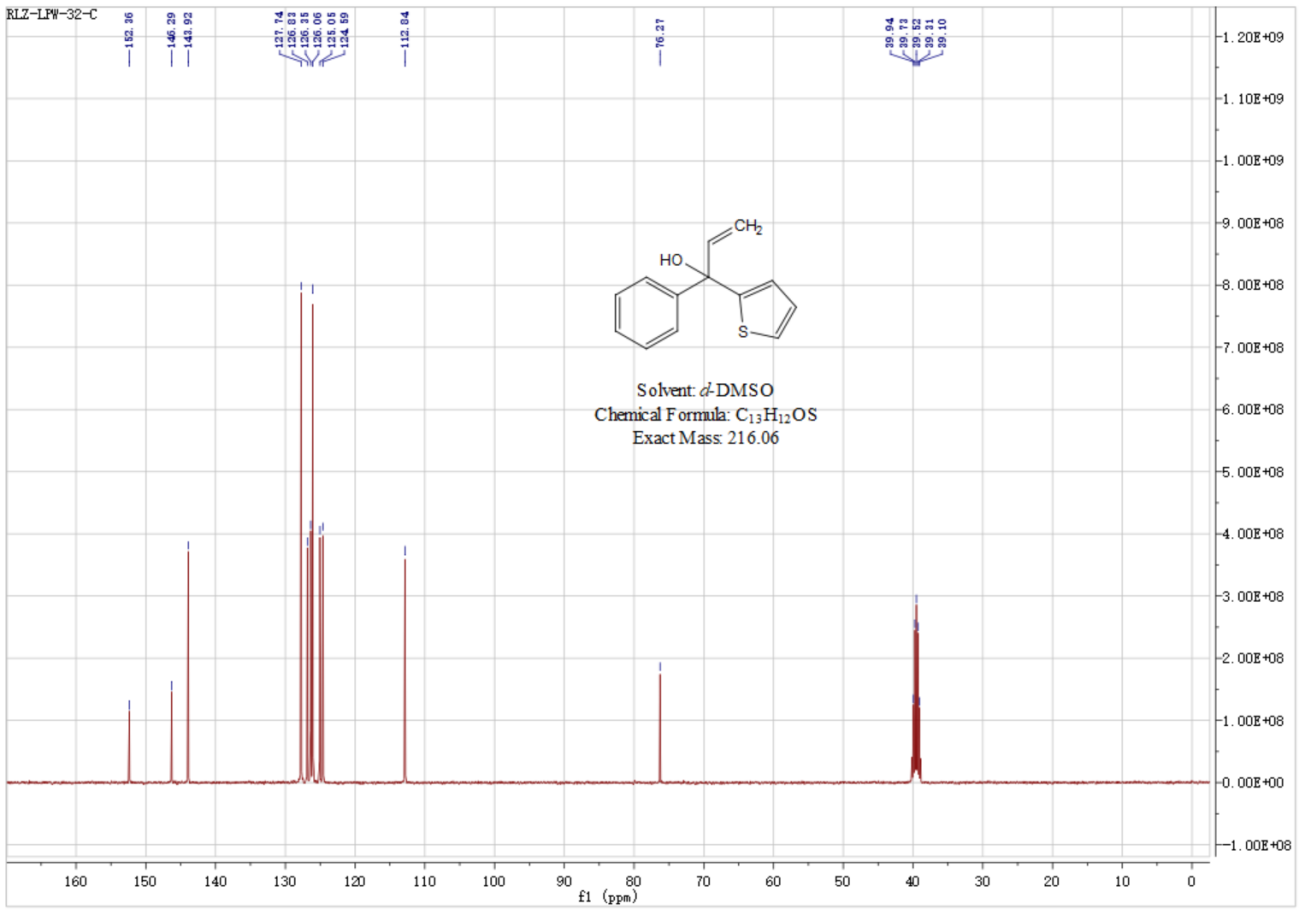


Figure S59. The ^13^C NMR of compound **2s.**


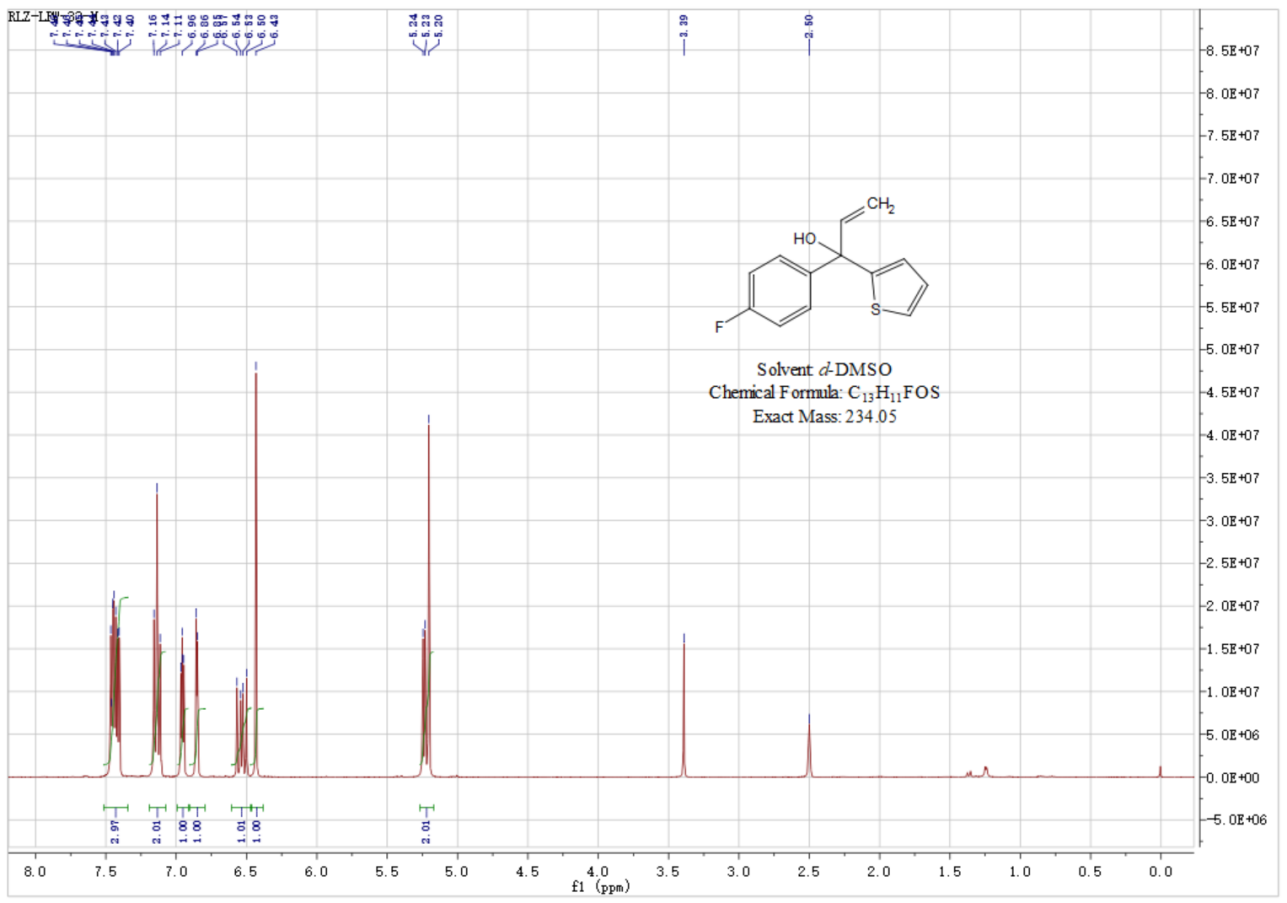


Figure S60. The ^1^H NMR of compound **2t.**


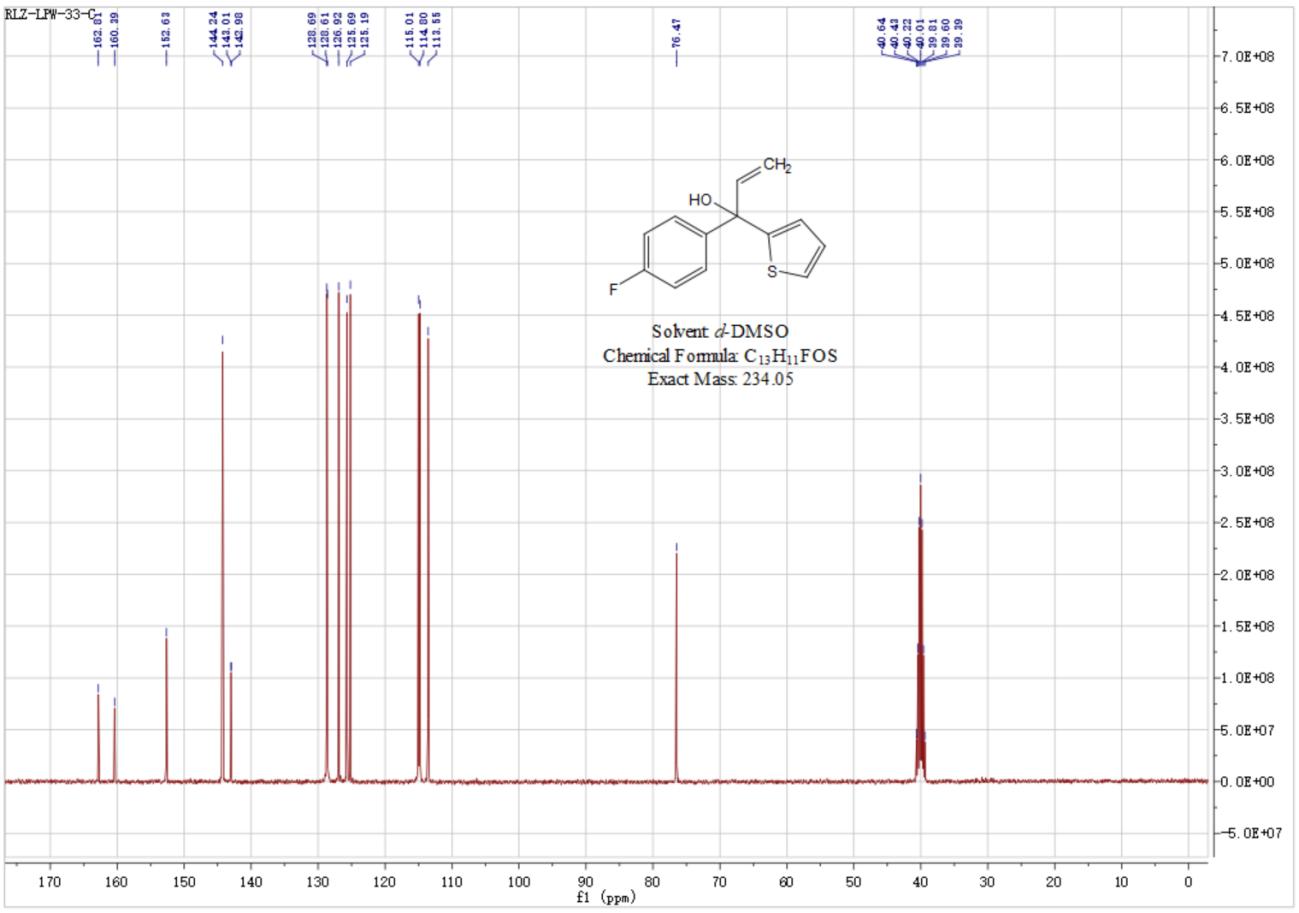


Figure S61. The ^13^C NMR of compound **2t.**


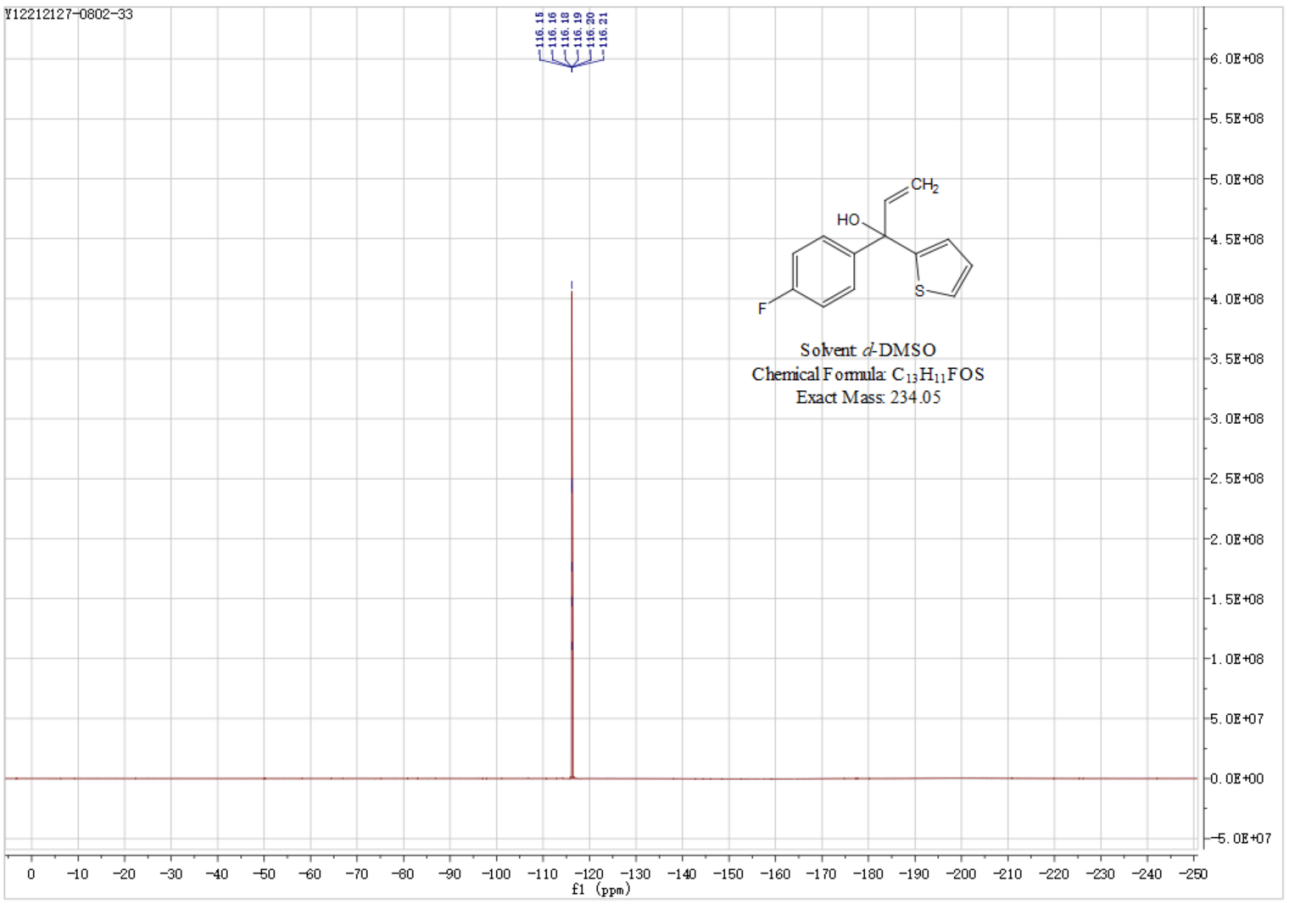


Figure S62. The ^19^F NMR of compound **2t.**


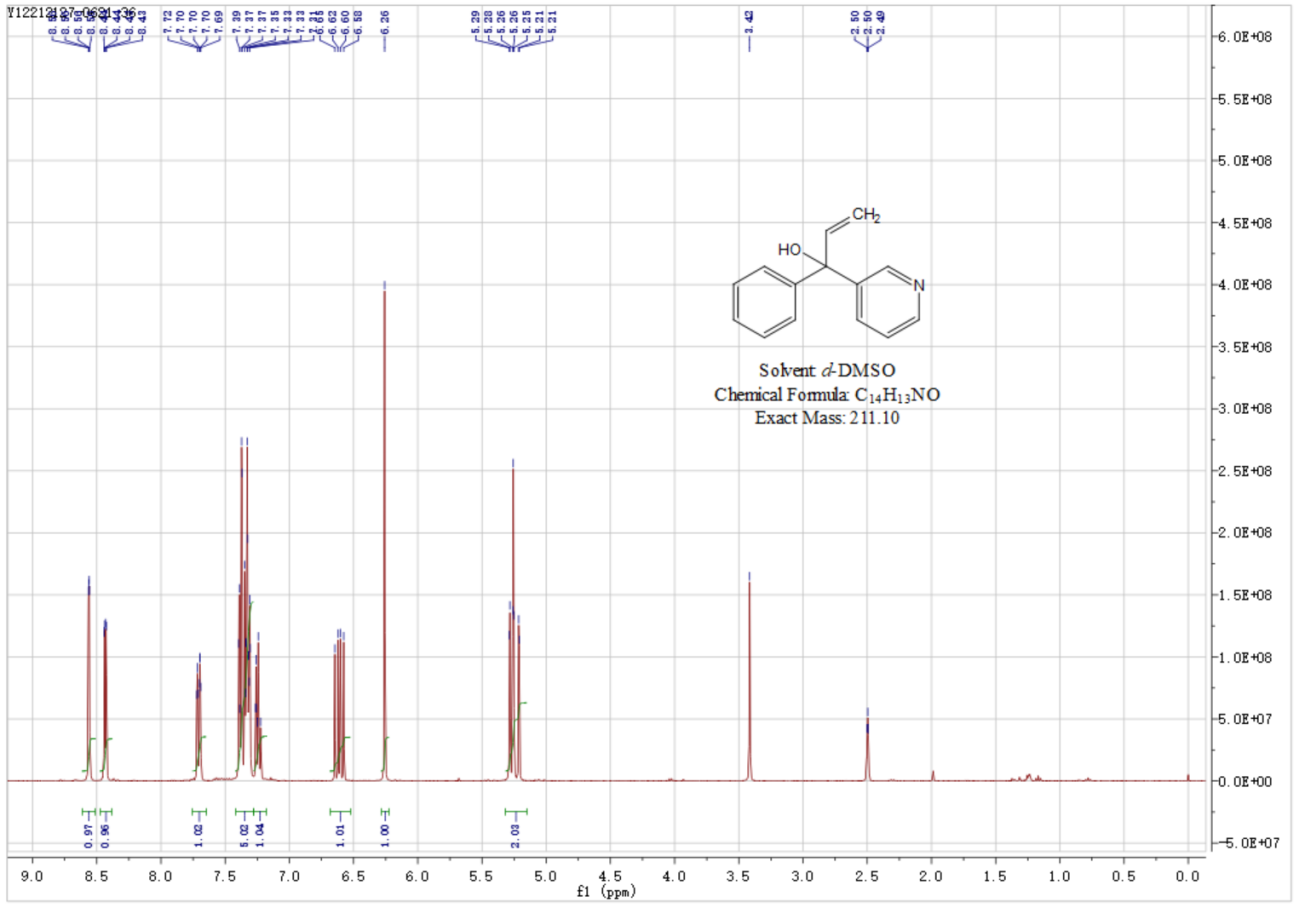


Figure S63. The ^1^H NMR of compound **2u.**


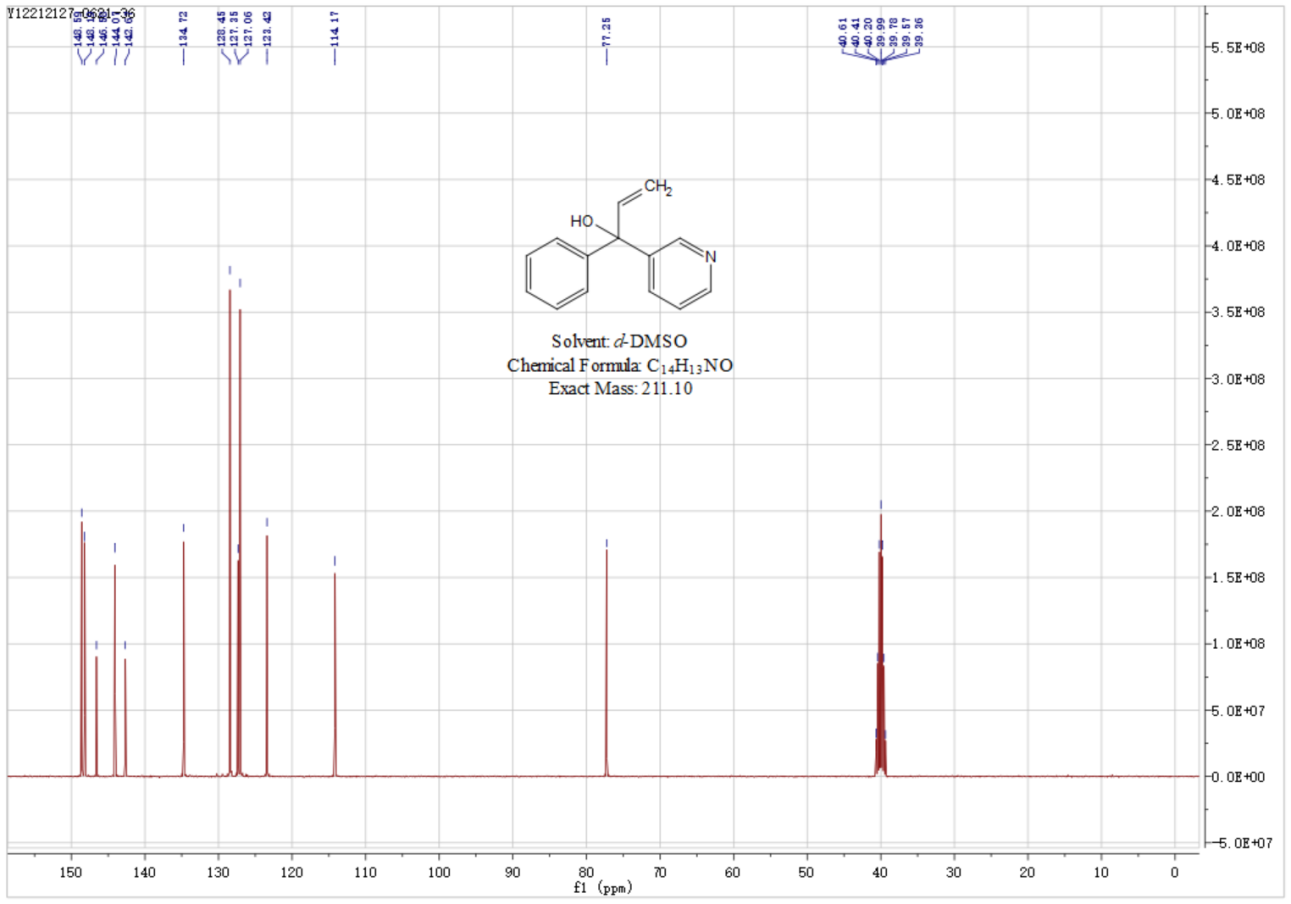


Figure S64. The ^13^C NMR of compound **2u.**


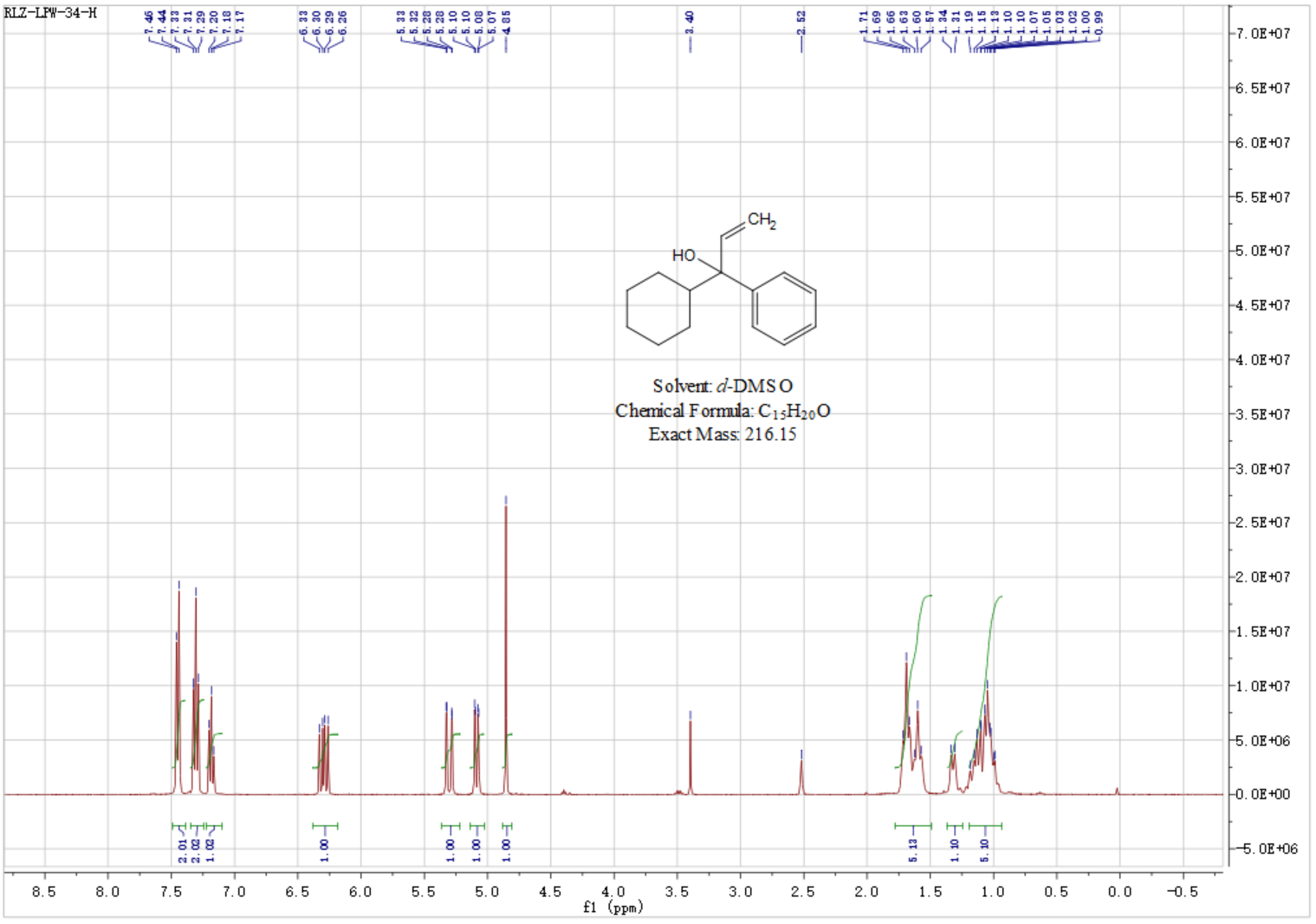


Figure S65. The ^1^H NMR of compound **2v.**


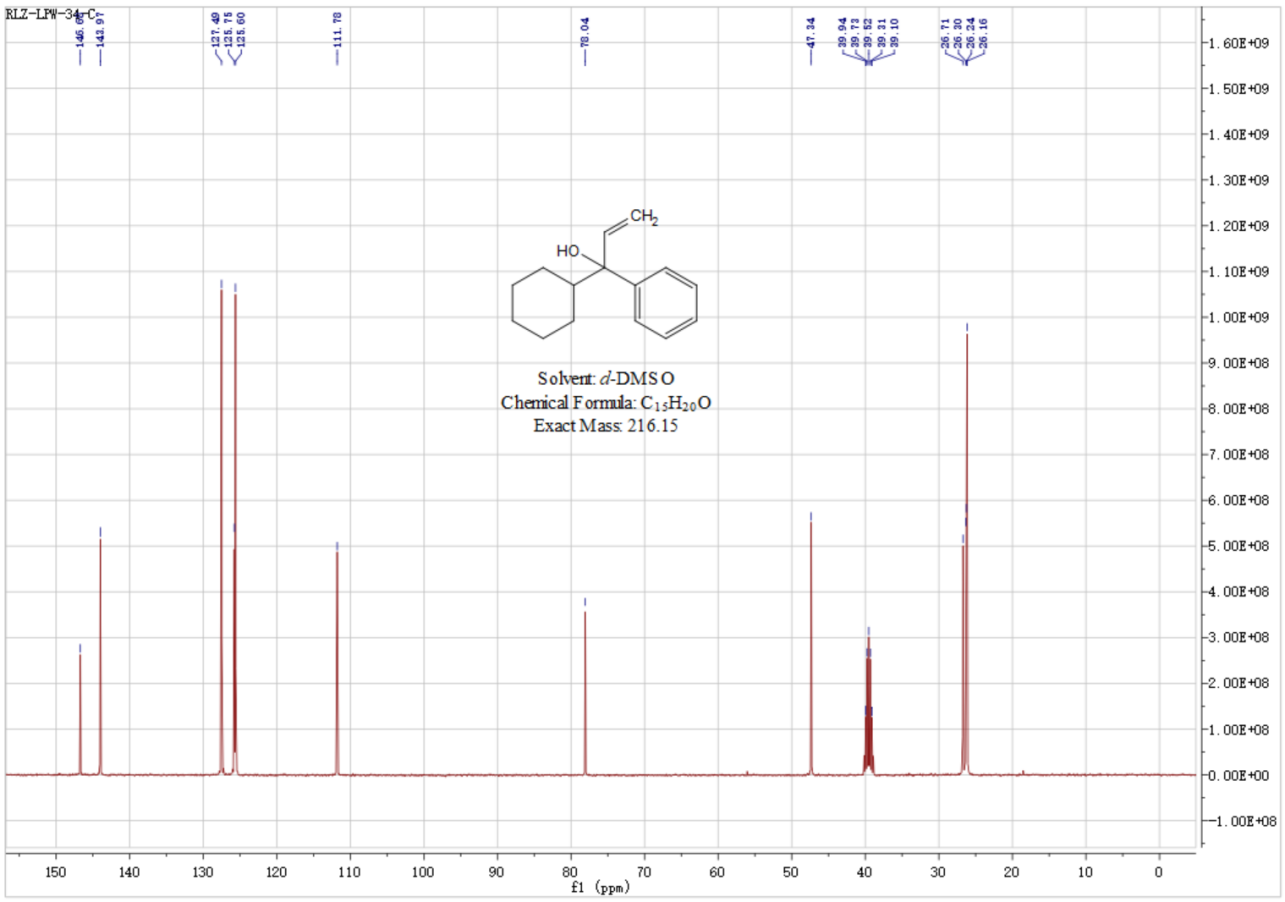


Figure S66. The ^13^C NMR of compound **2v.**


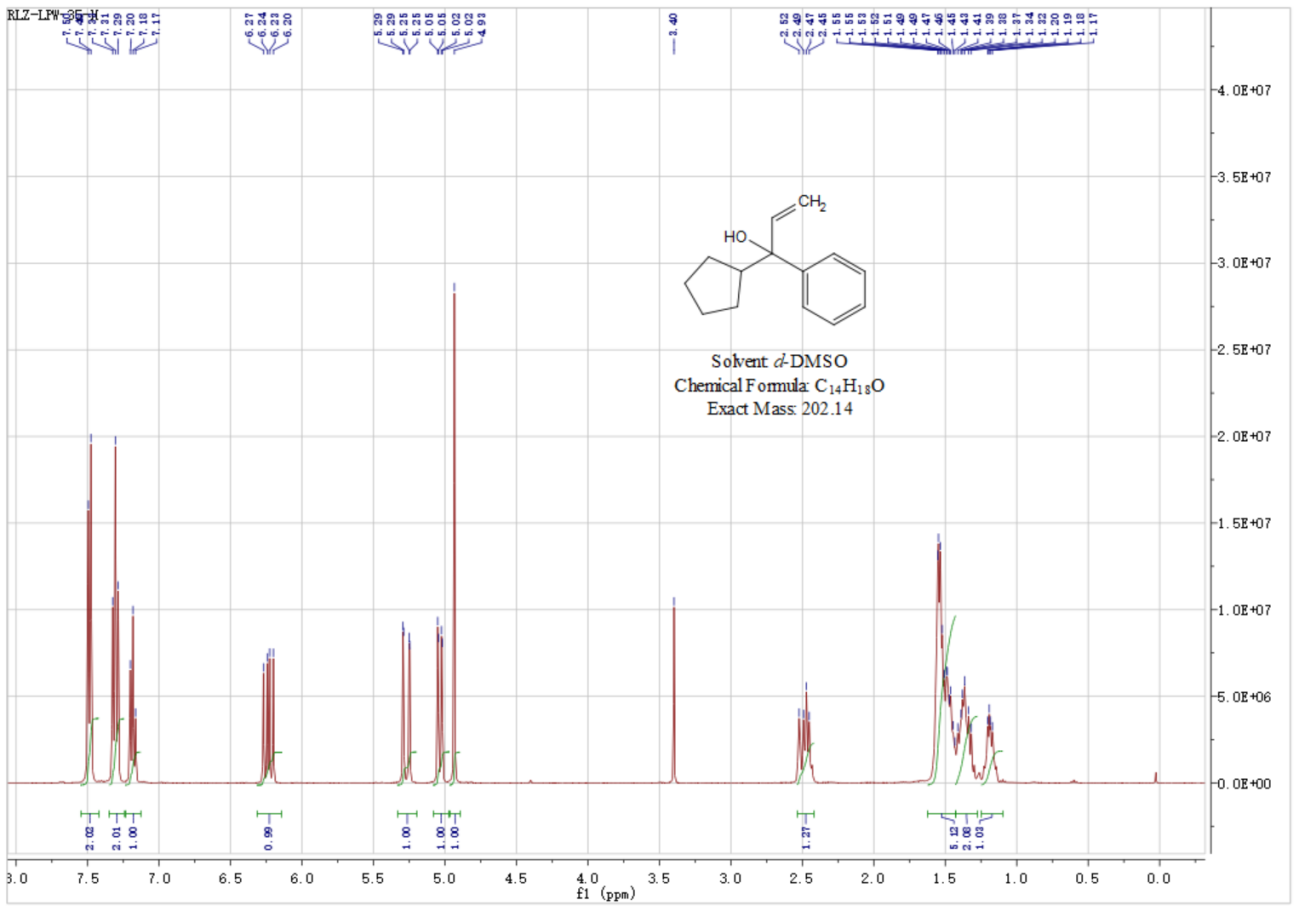


Figure S67. The ^1^H NMR of compound **2w.**


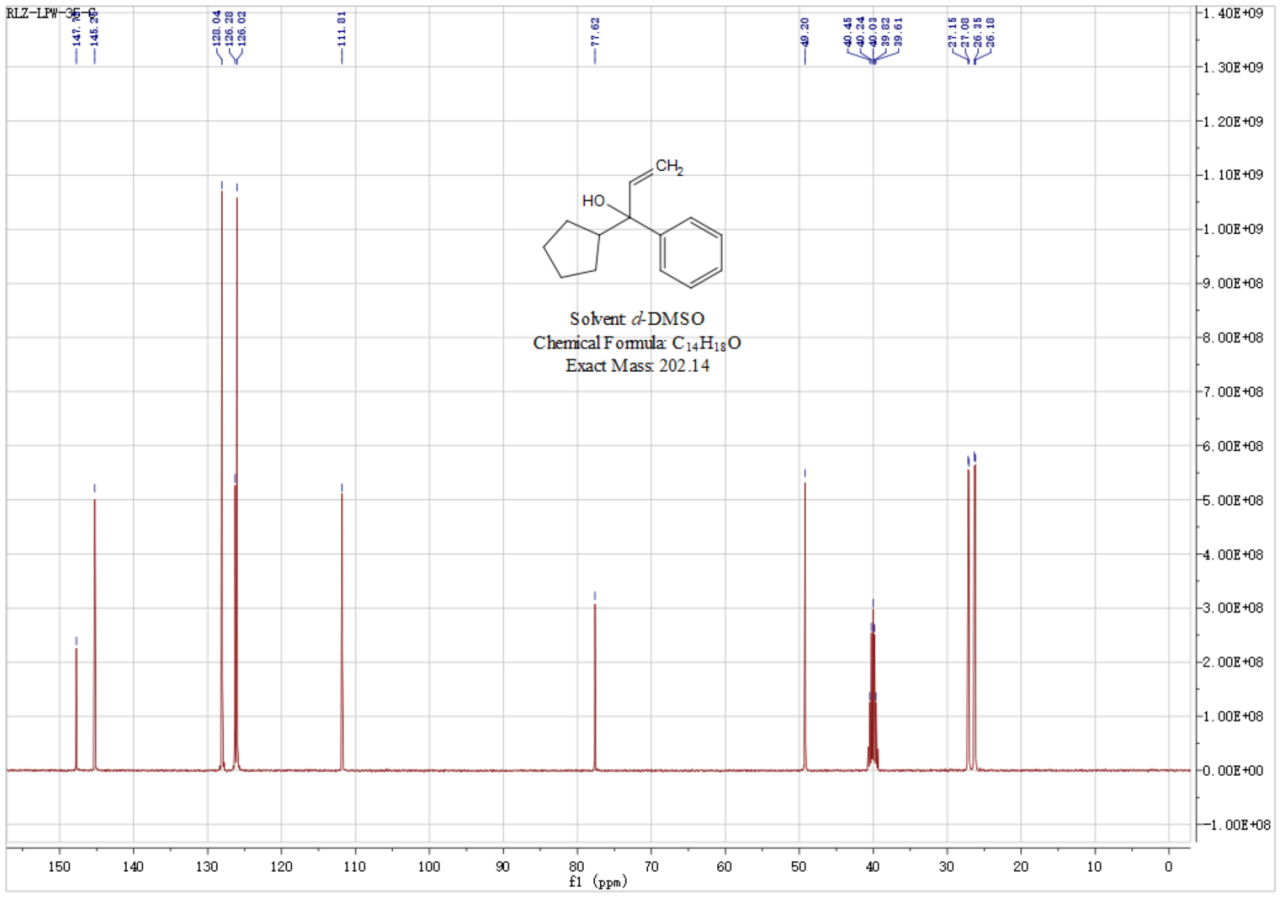


Figure S68. The ^13^C NMR of compound **2w.**


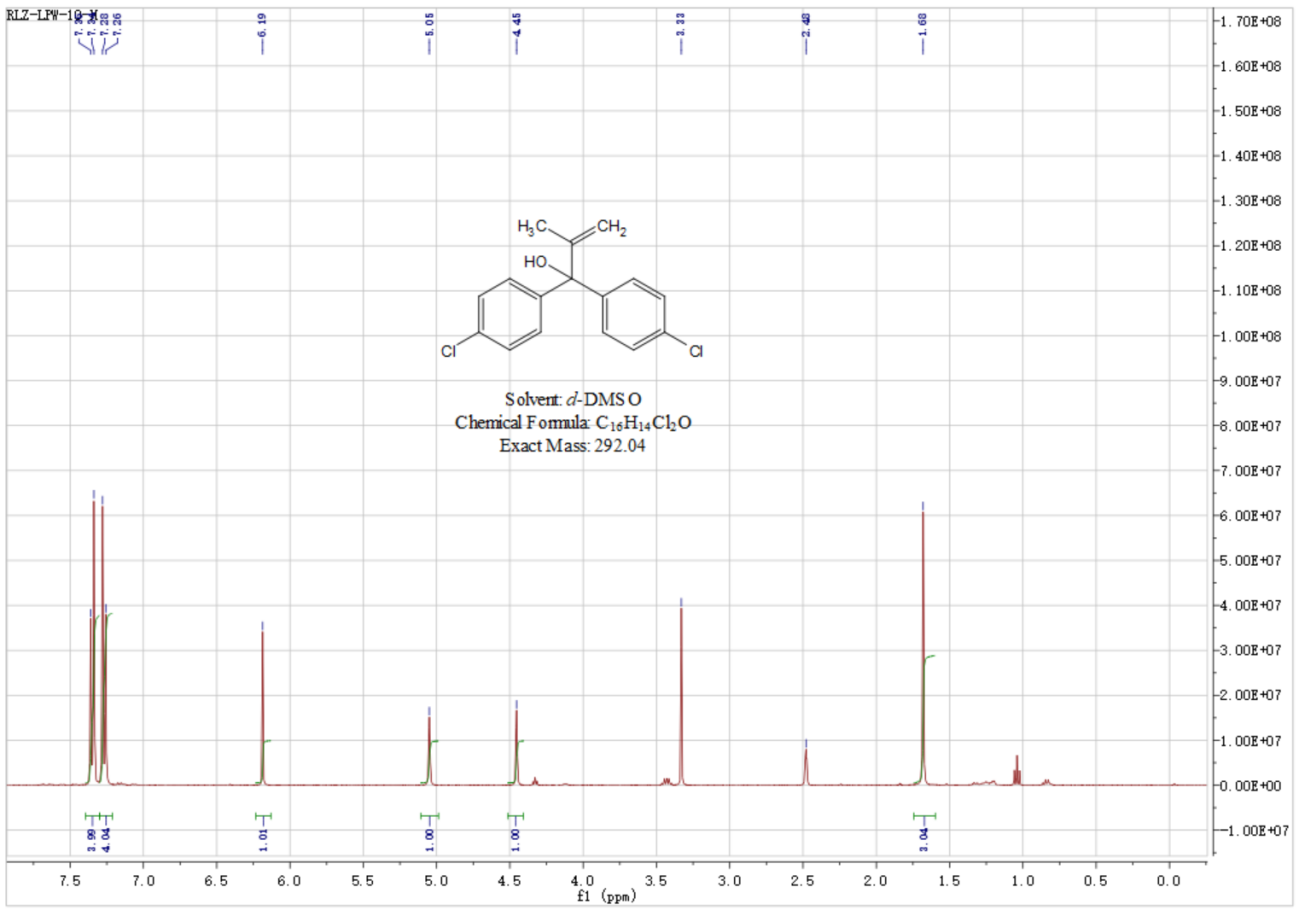


Figure S69. The ^1^H NMR of compound **2x.**


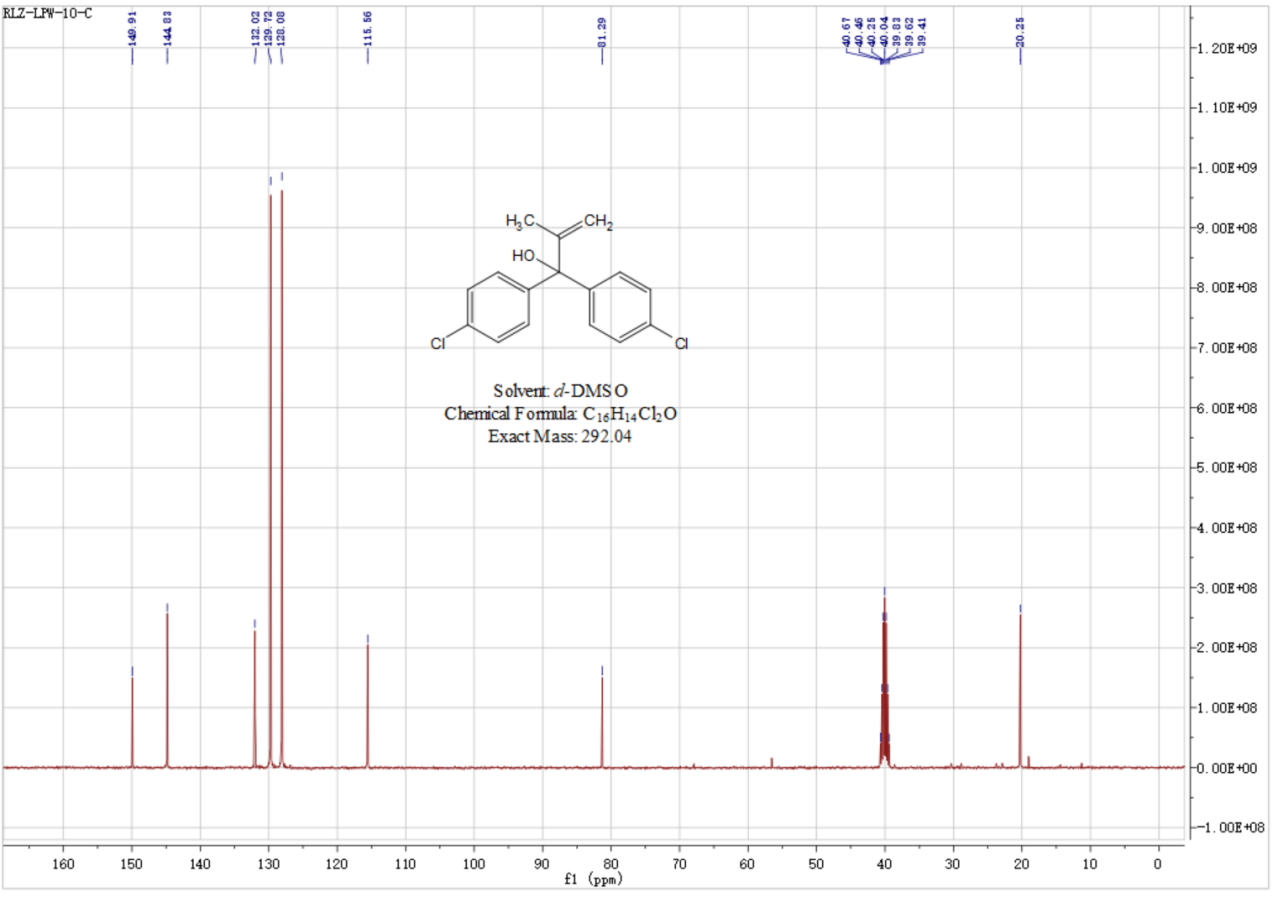


Figure S70. The ^13^C NMR of compound **2x.**


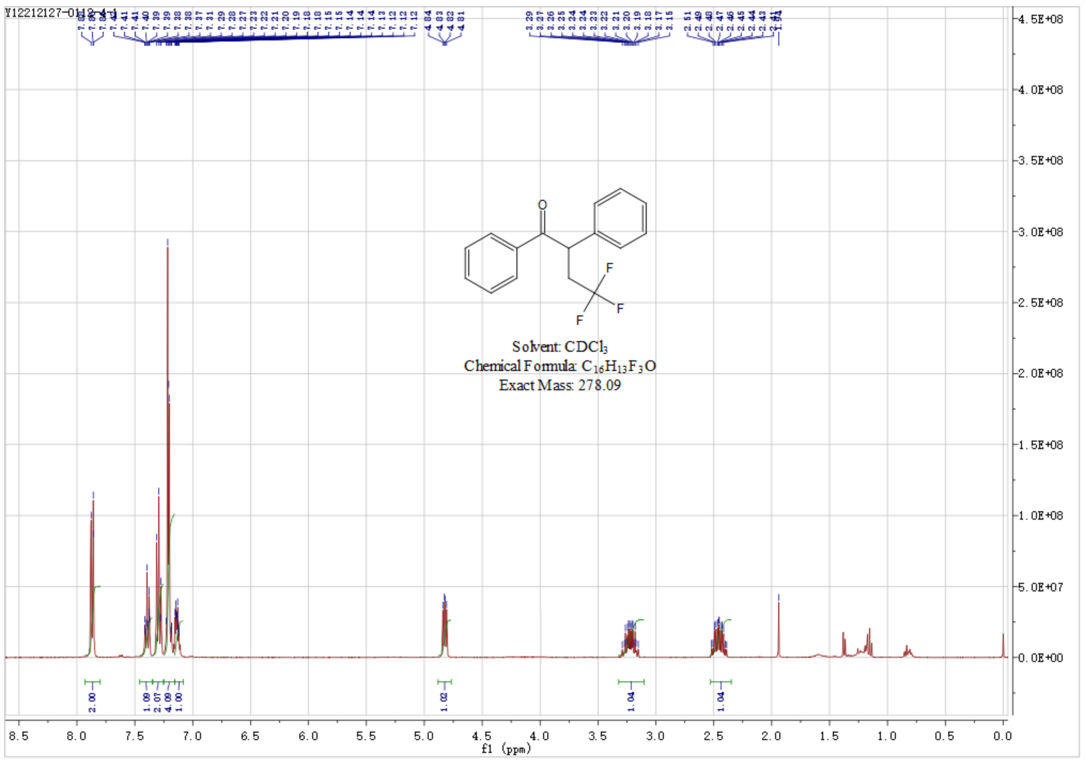


Figure S71. The ^1^H NMR of compound **3a.**


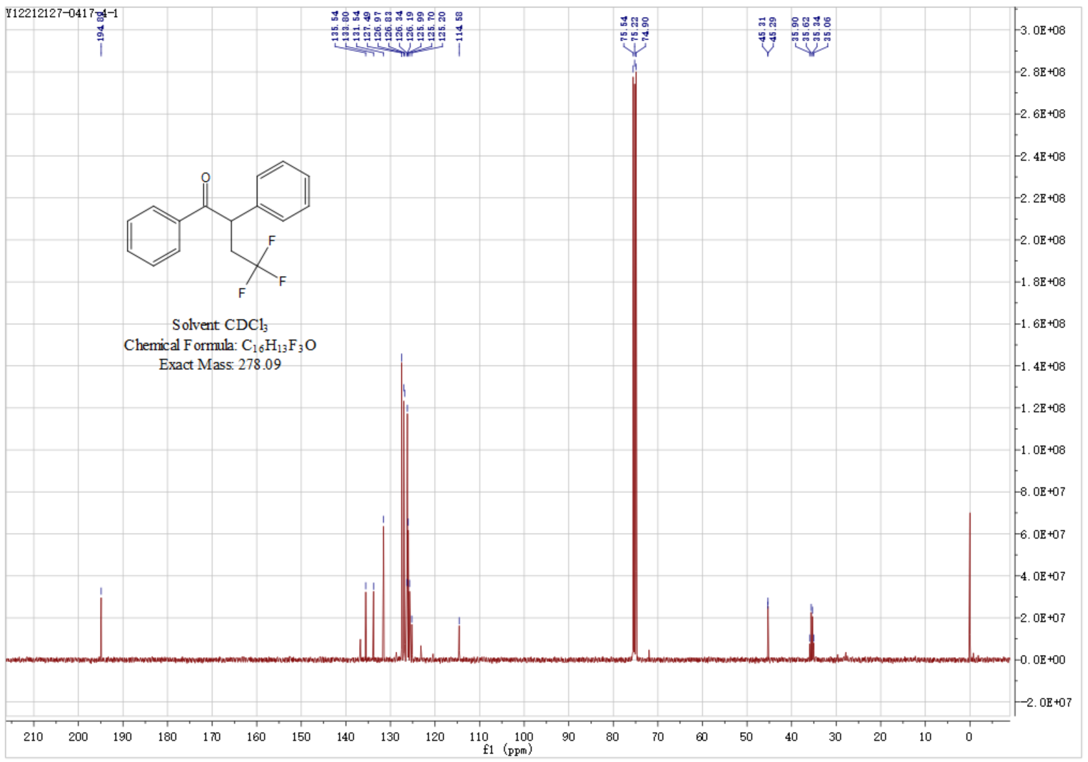


Figure S72. The ^13^C NMR of compound **3a.**


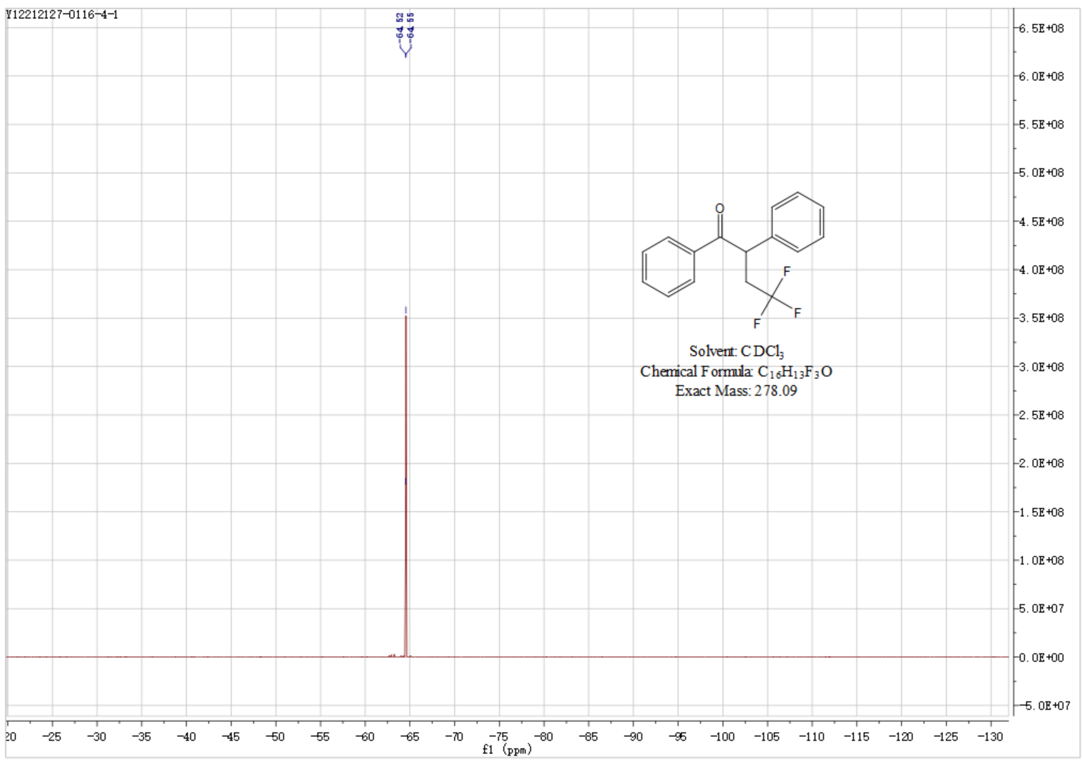


Figure S73. The ^19^F NMR of compound **3a.**


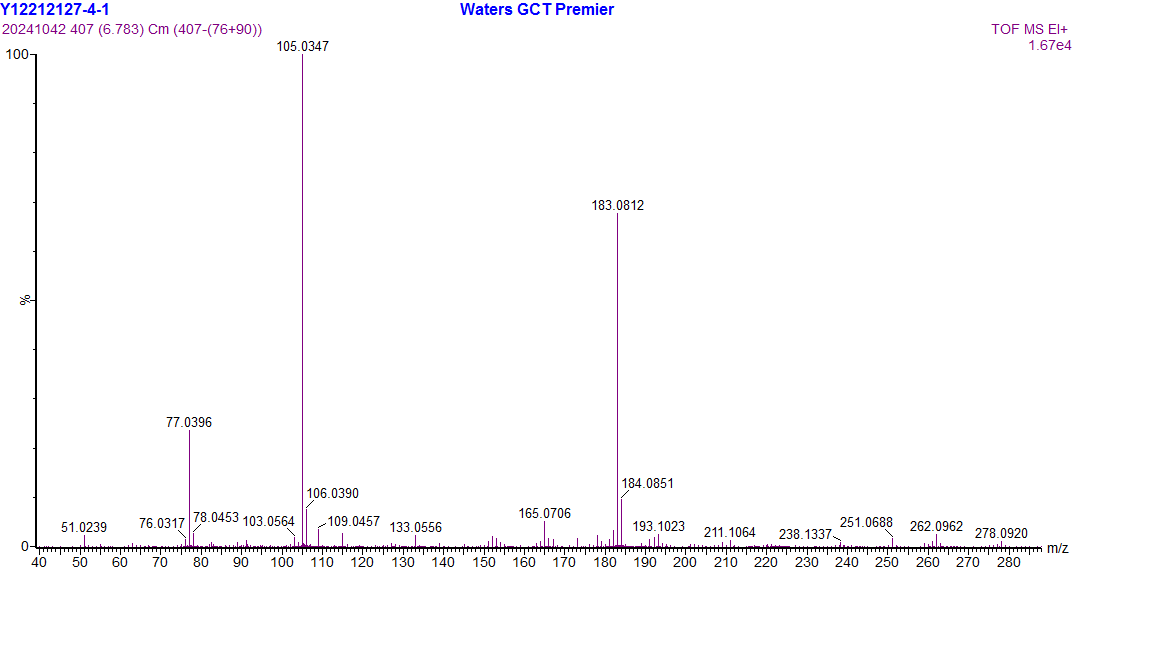


Figure S74. The HR-MS of compound **3a.**


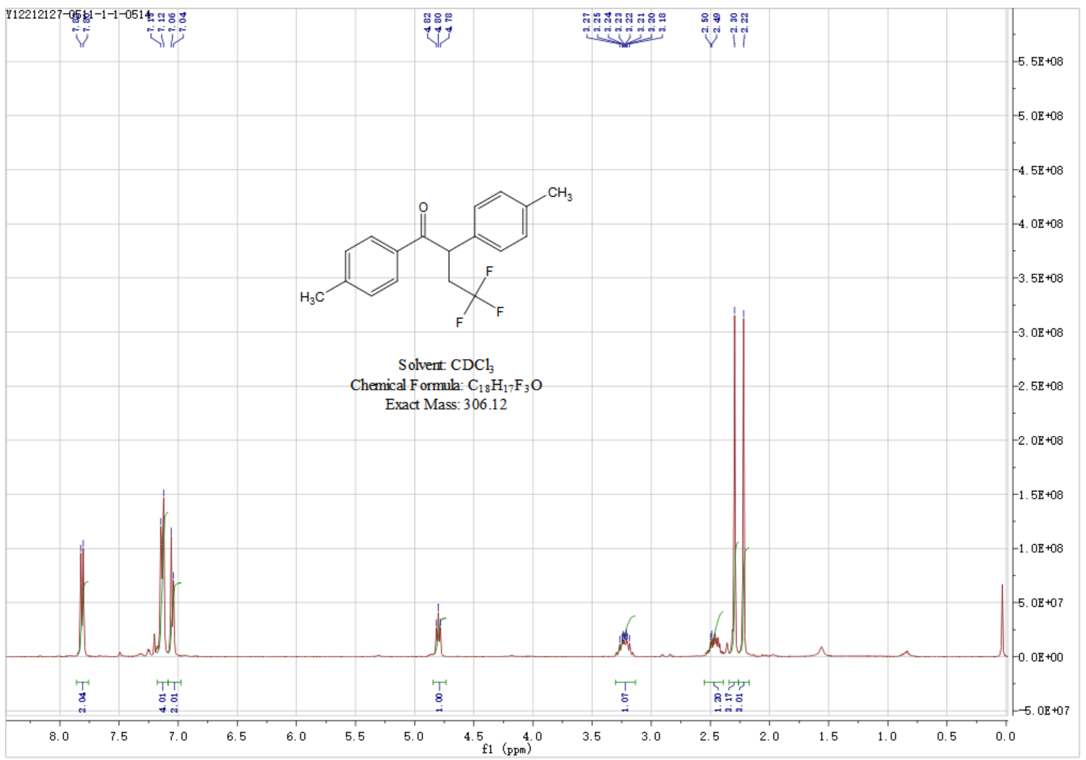


Figure S75. The ^1^H NMR of compound **3b.**


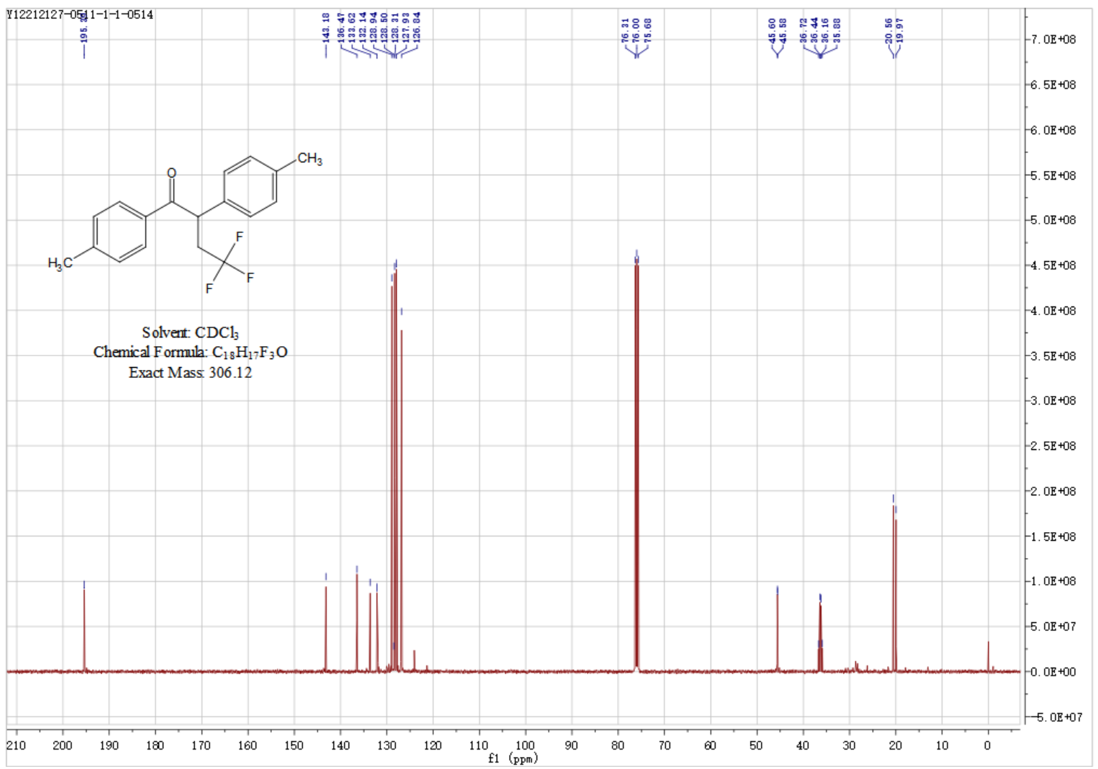


Figure S76. The ^13^C NMR of compound **3b.**


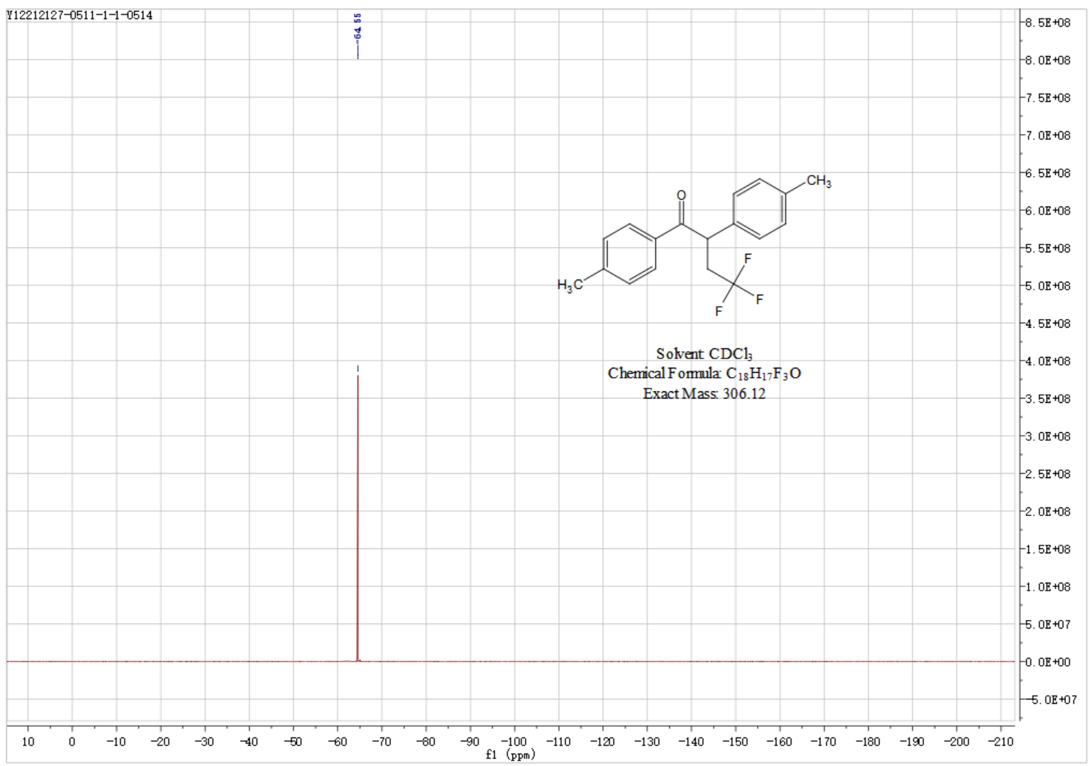


Figure S77. The ^19^F NMR of compound **3b.**


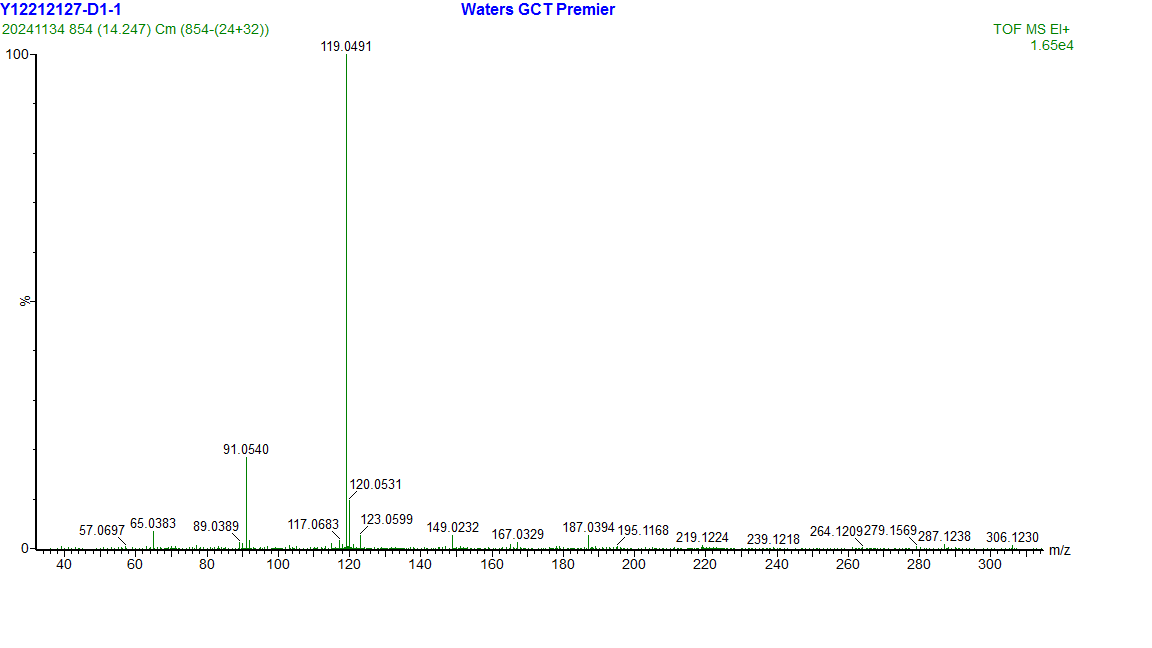


Figure S78. The HR-MS of compound **3b.**


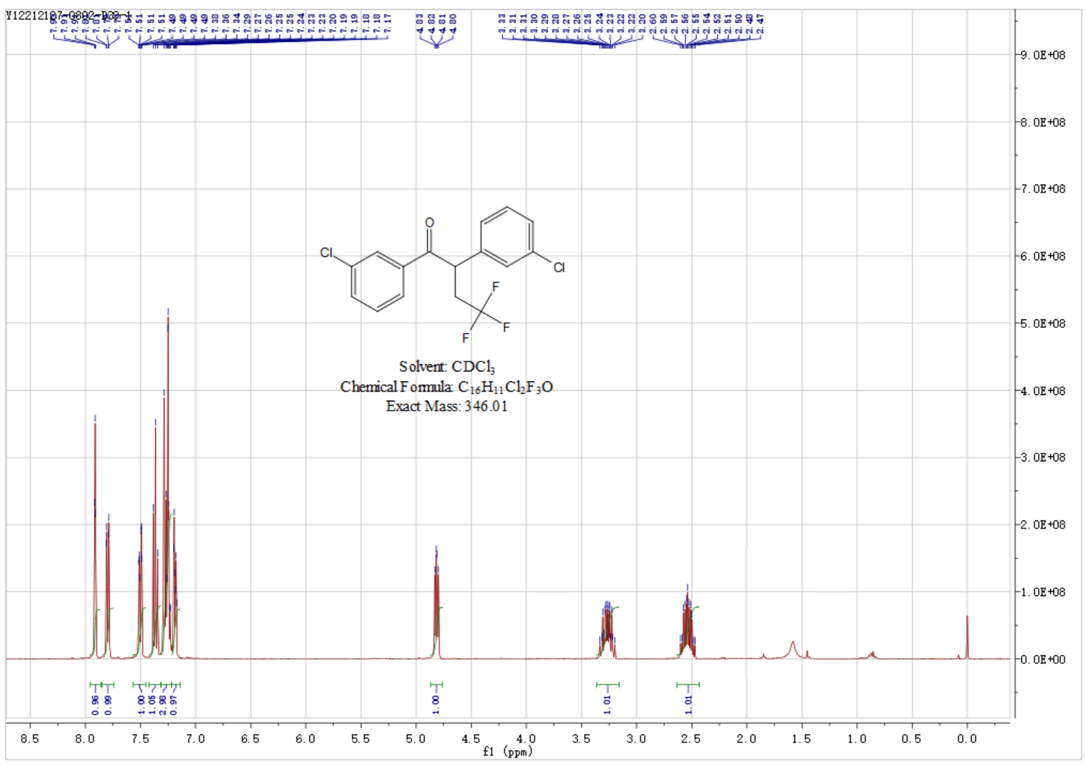


Figure S79. The ^1^H NMR of compound **3c.**


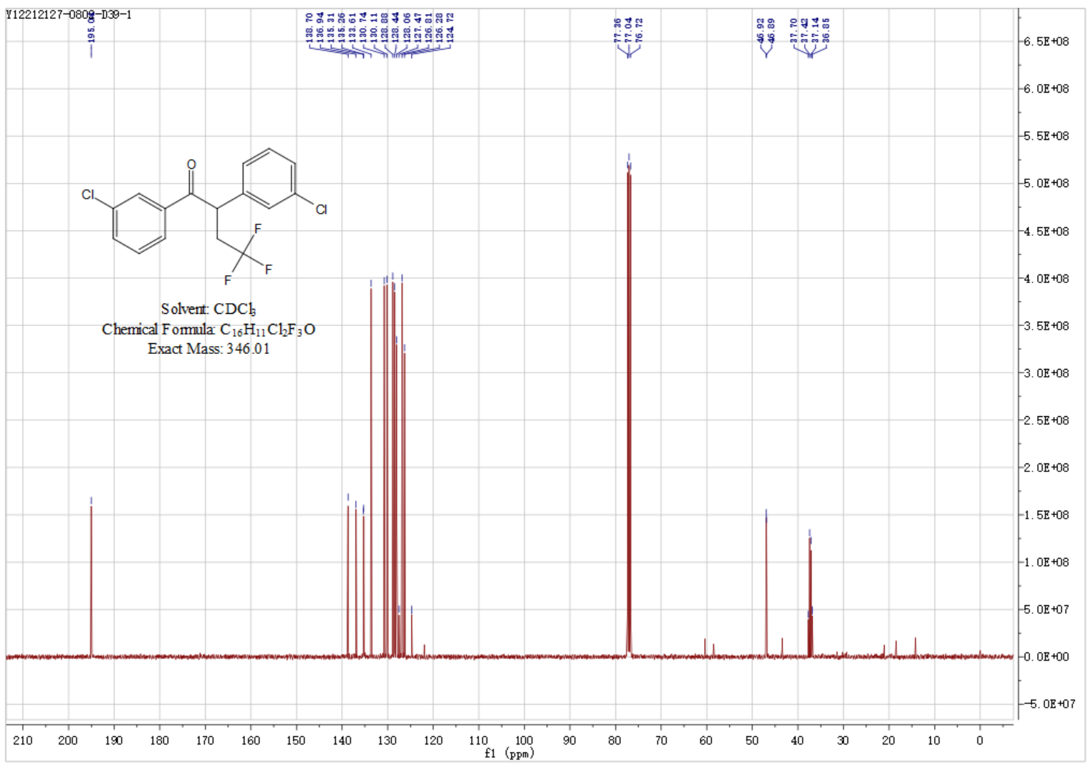


Figure S80. The ^13^C NMR of compound **3c.**


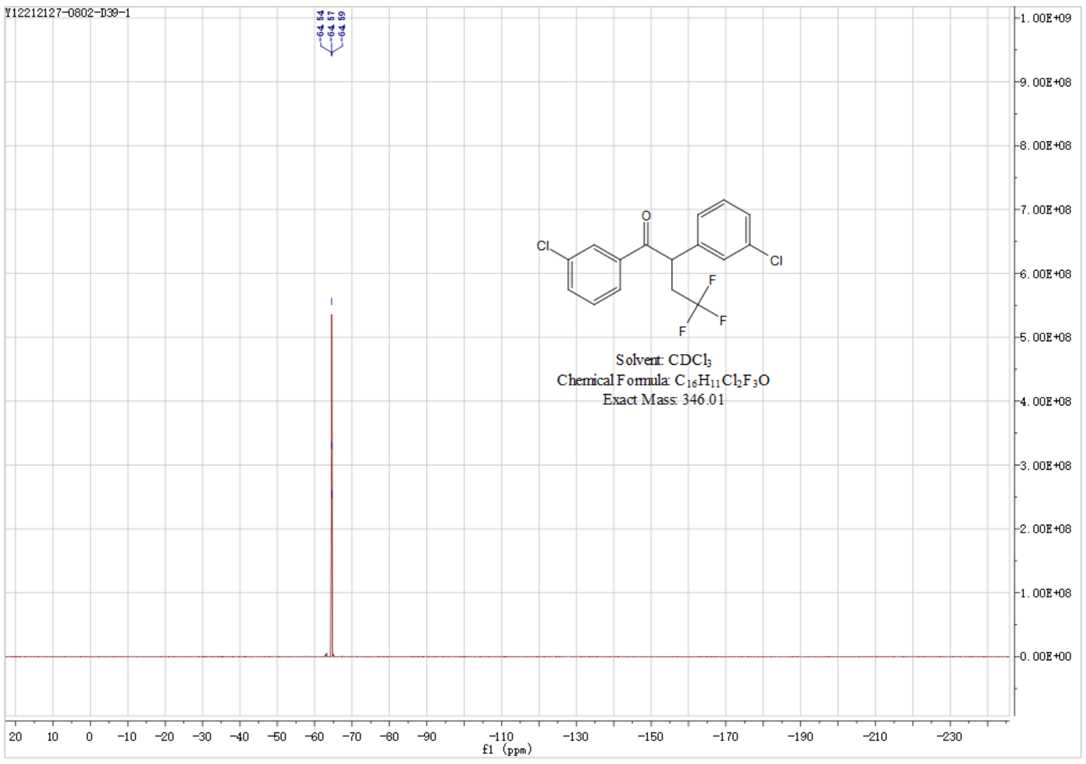


Figure S81. The ^19^F NMR of compound **3c.**


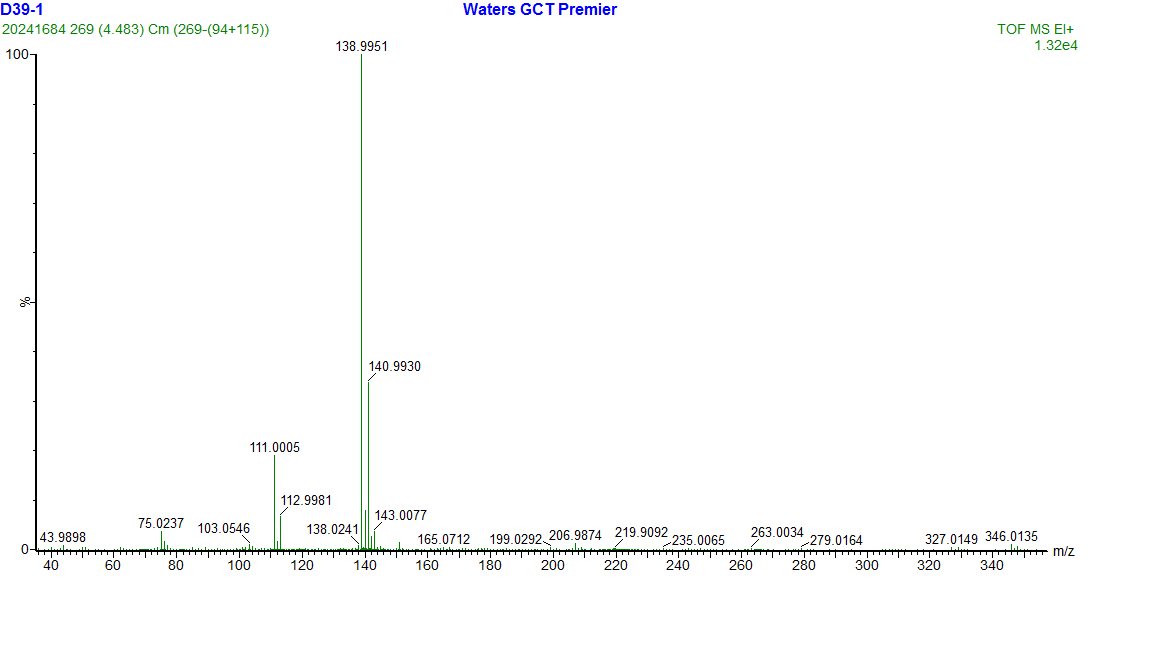


Figure S82. The HR-MS of compound **3c.**


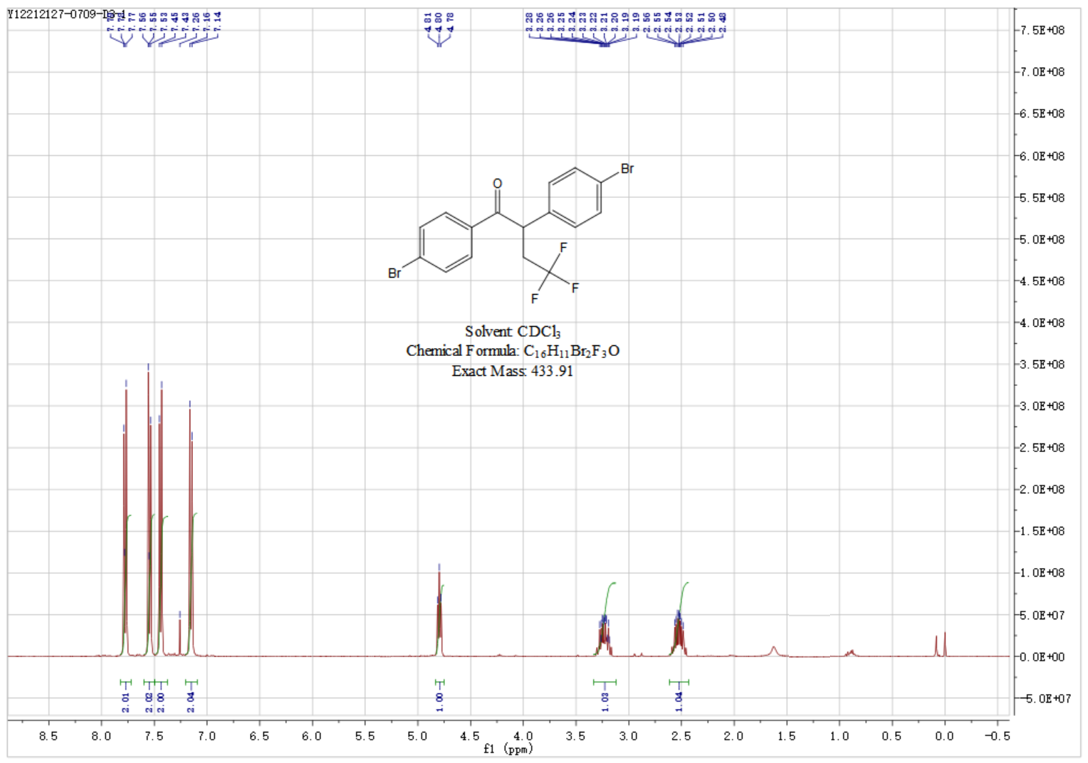


Figure S83. The ^1^H NMR of compound **3d.**


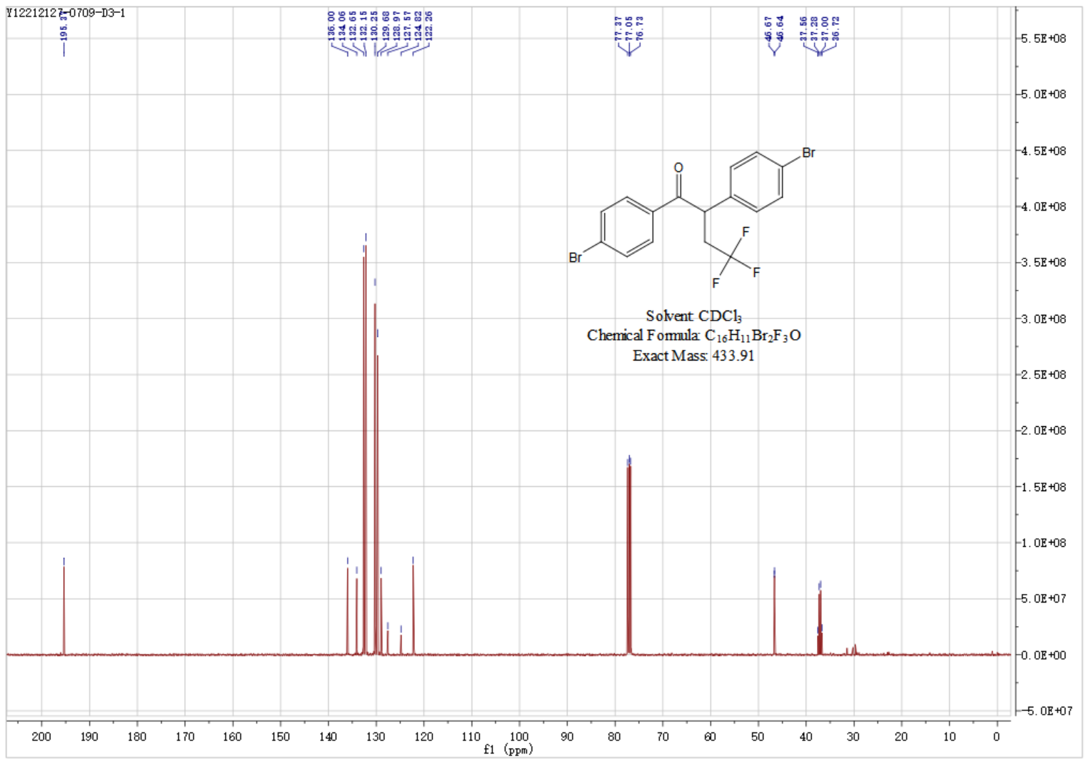


Figure S84. The ^13^C NMR of compound **3d.**


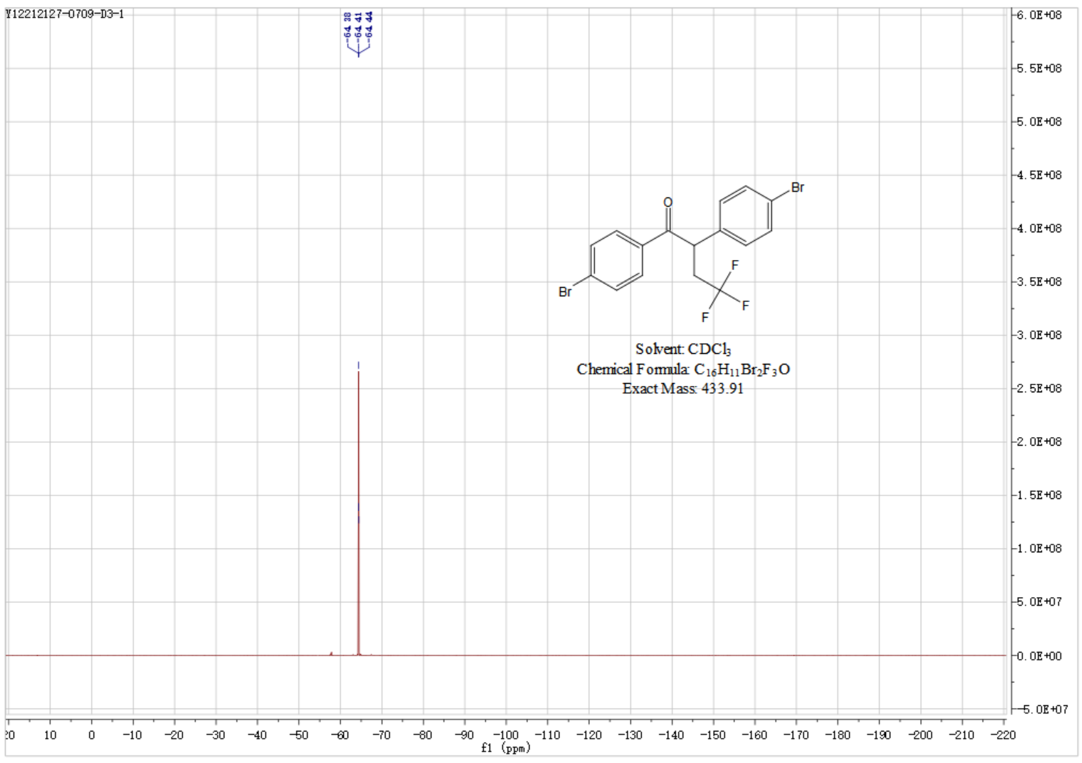


Figure S85. The ^19^F NMR of compound **3d.**


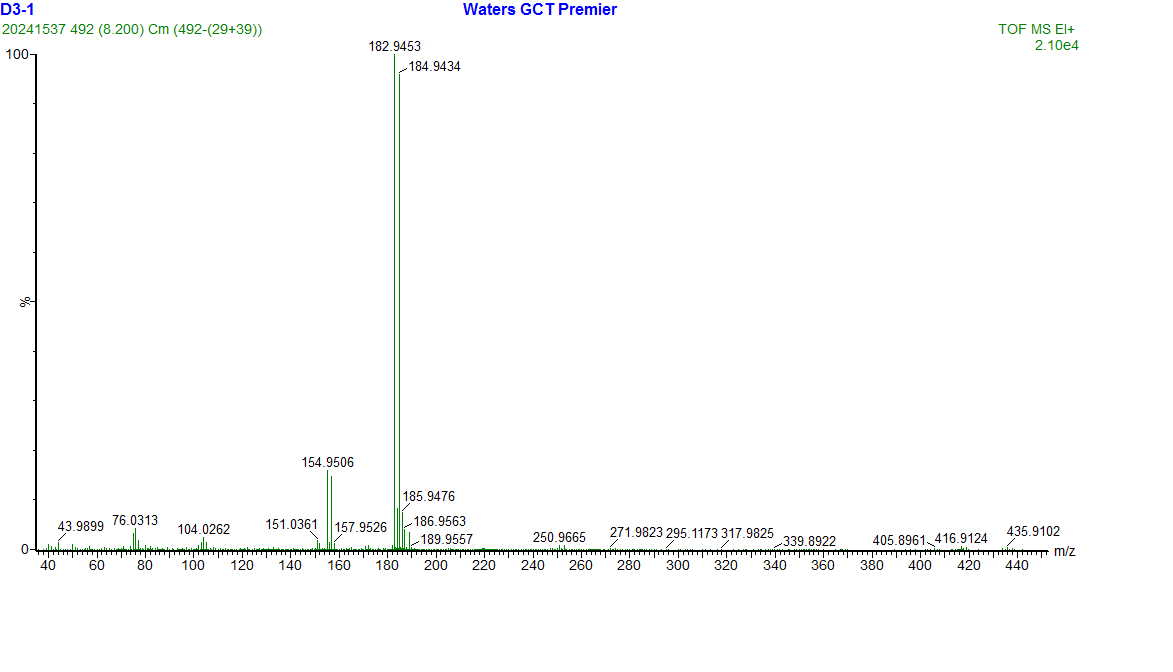


Figure S86. The HR-MS of compound **3d.**


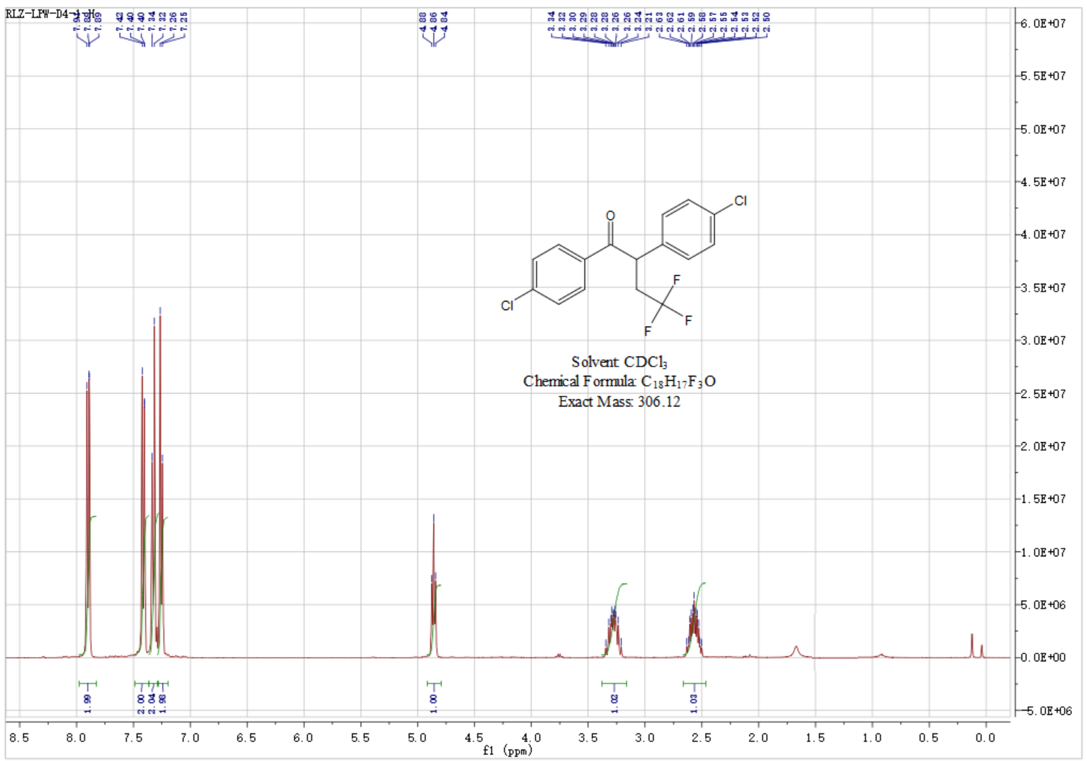


Figure S87. The ^1^H NMR of compound **3e.**


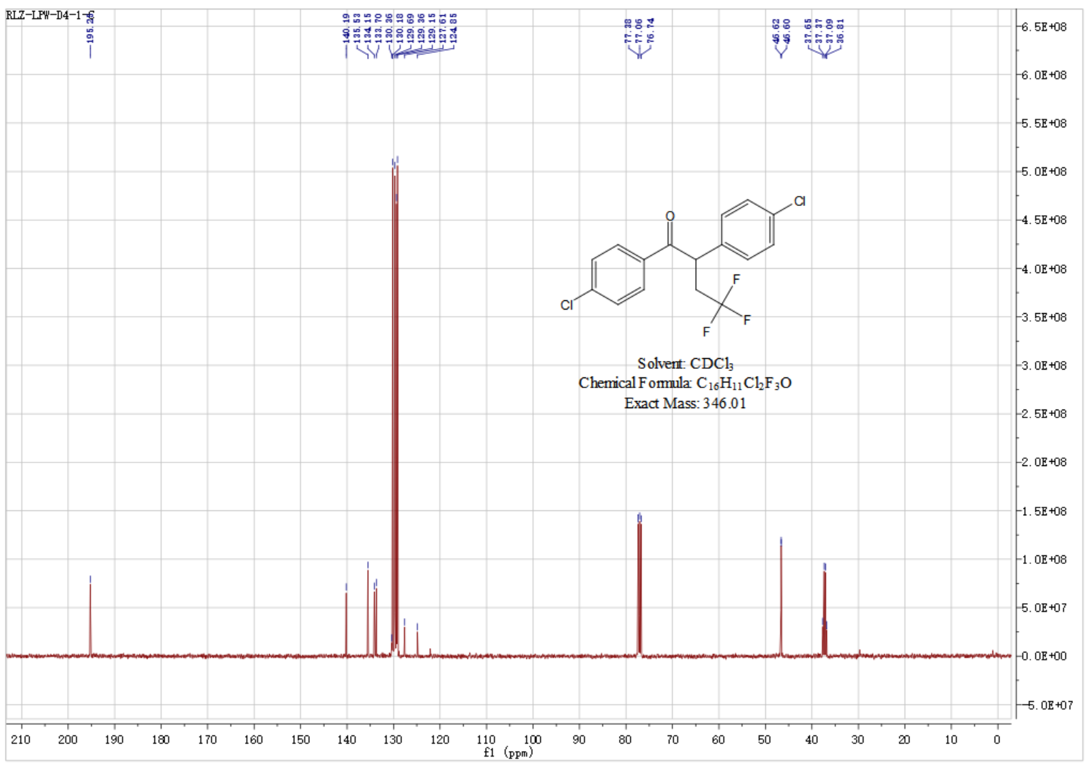


Figure S88. The ^13^C NMR of compound **3e.**


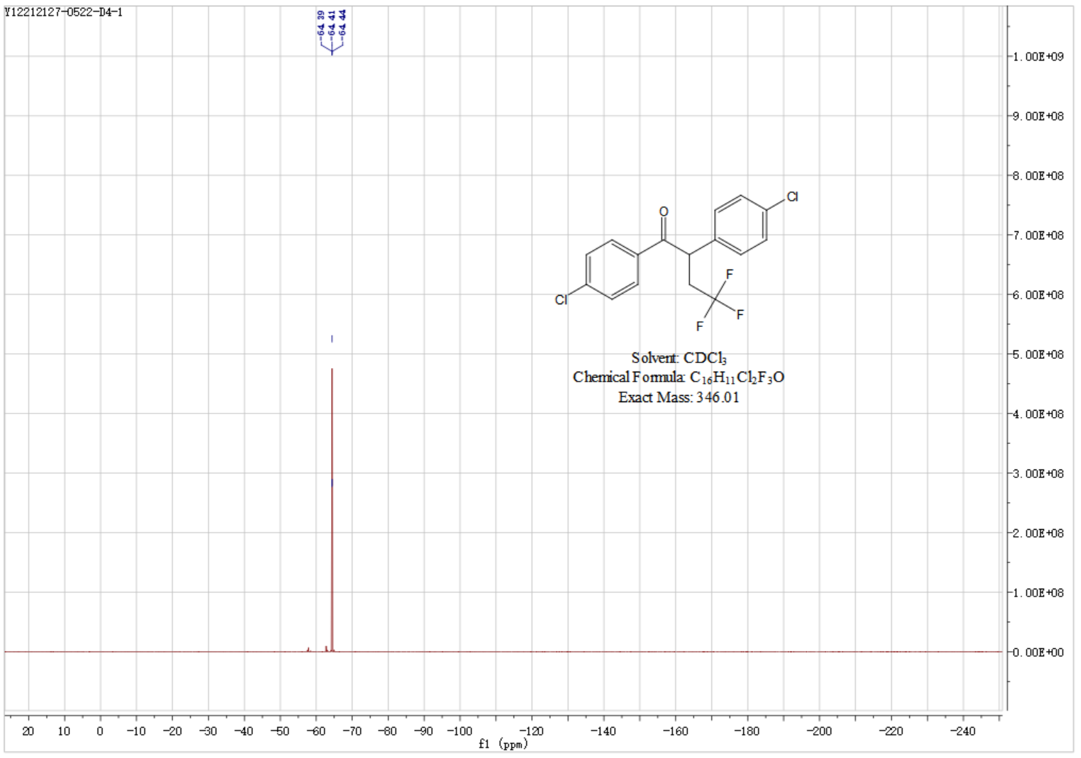


Figure S89. The ^19^F NMR of compound **3e.**


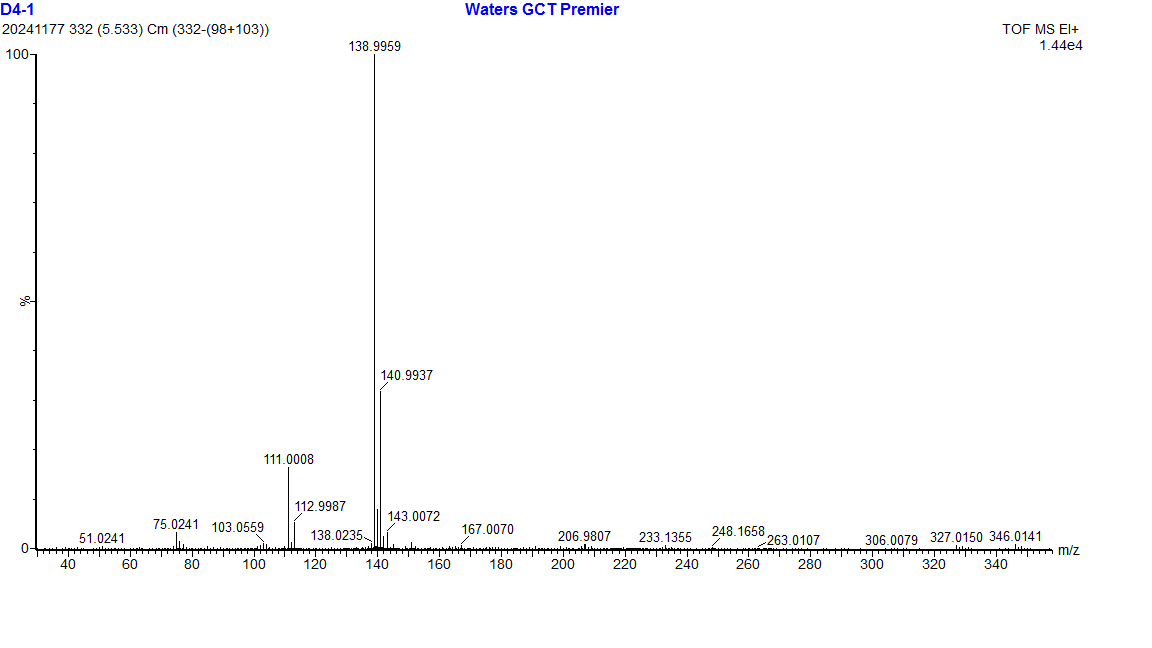


Figure S90. The HR-MS of compound **3e.**


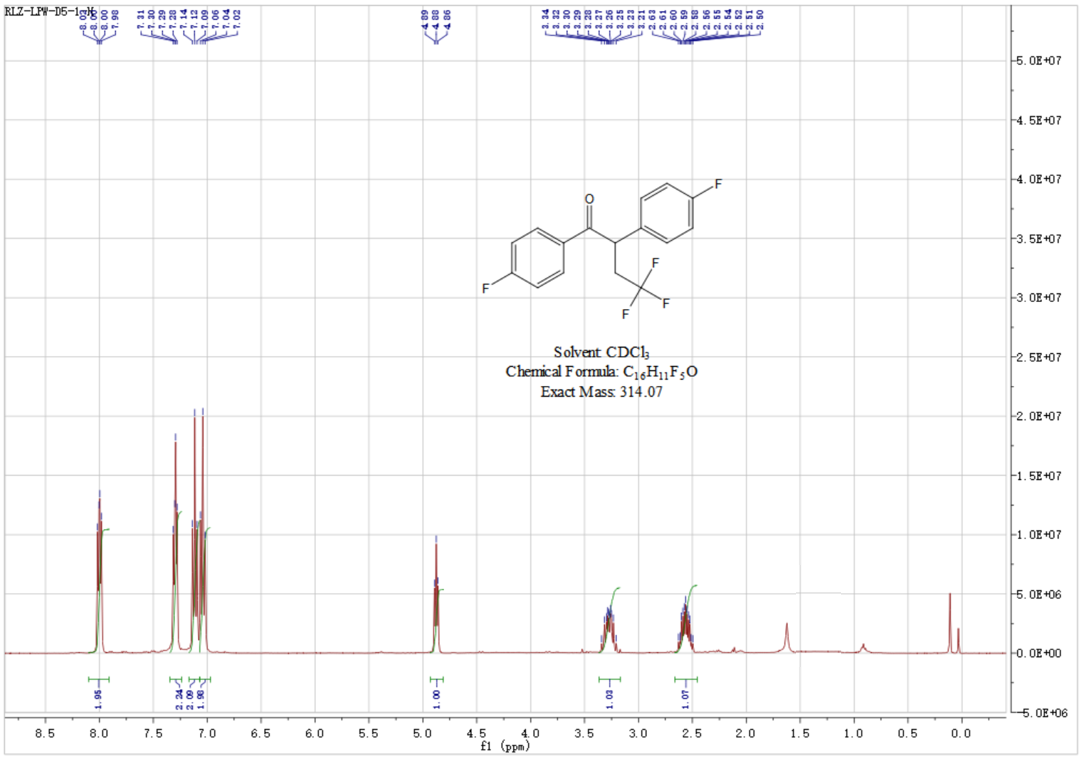


Figure S91. The ^1^H NMR of compound **3f.**


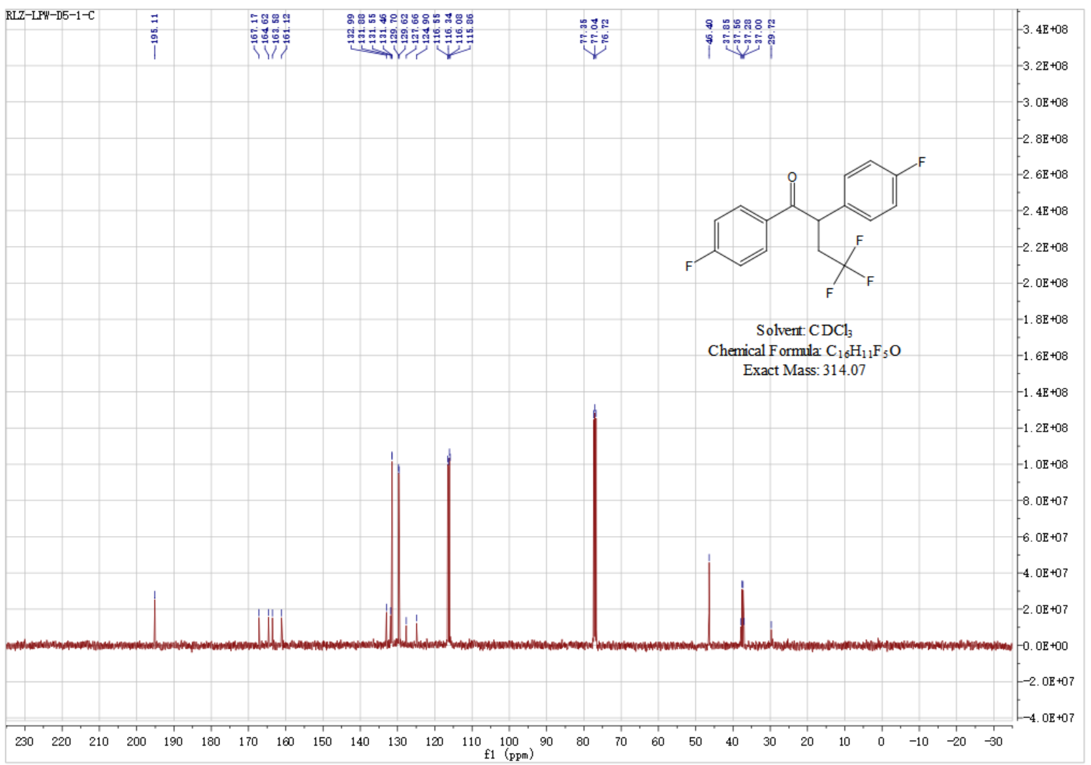


Figure S92. The ^13^C NMR of compound **3f.**


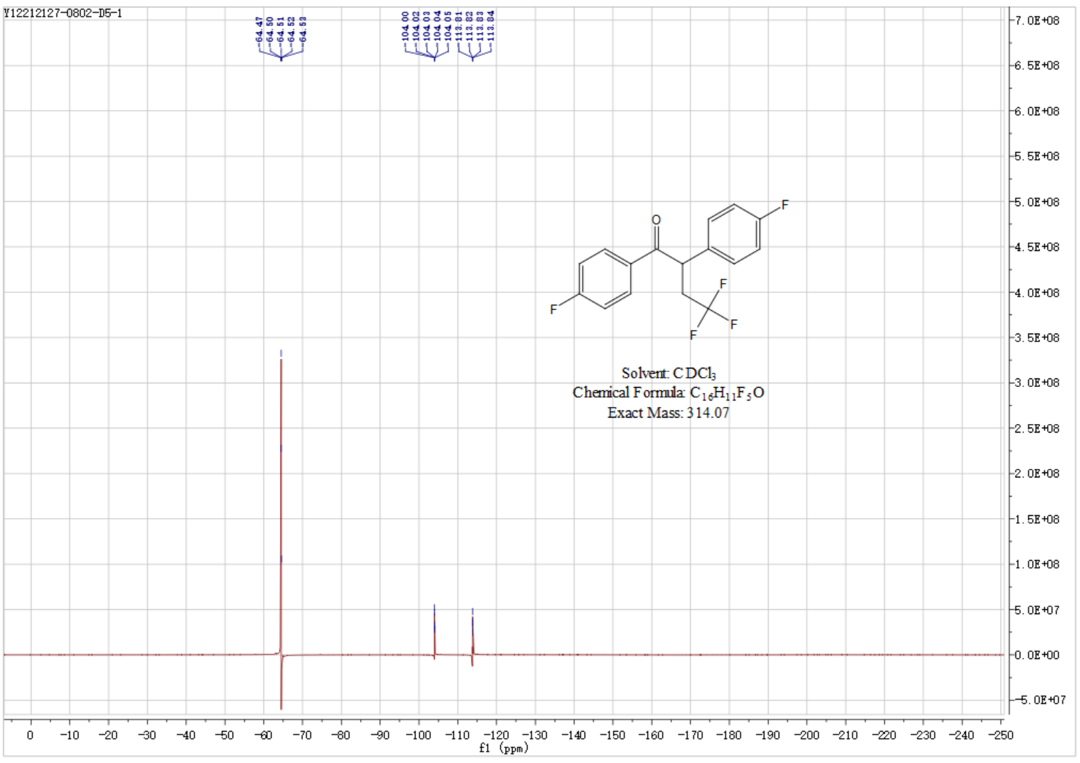


Figure S93. The ^19^F NMR of compound **3f.**


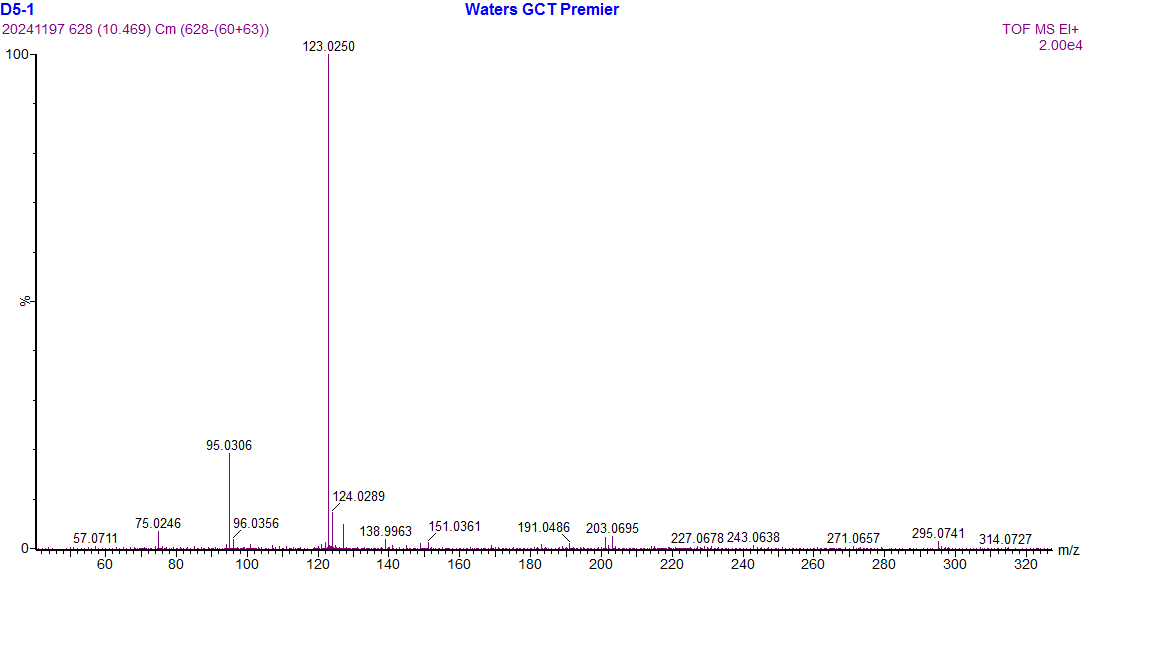


Figure S94. The HR-MS of compound **3f.**


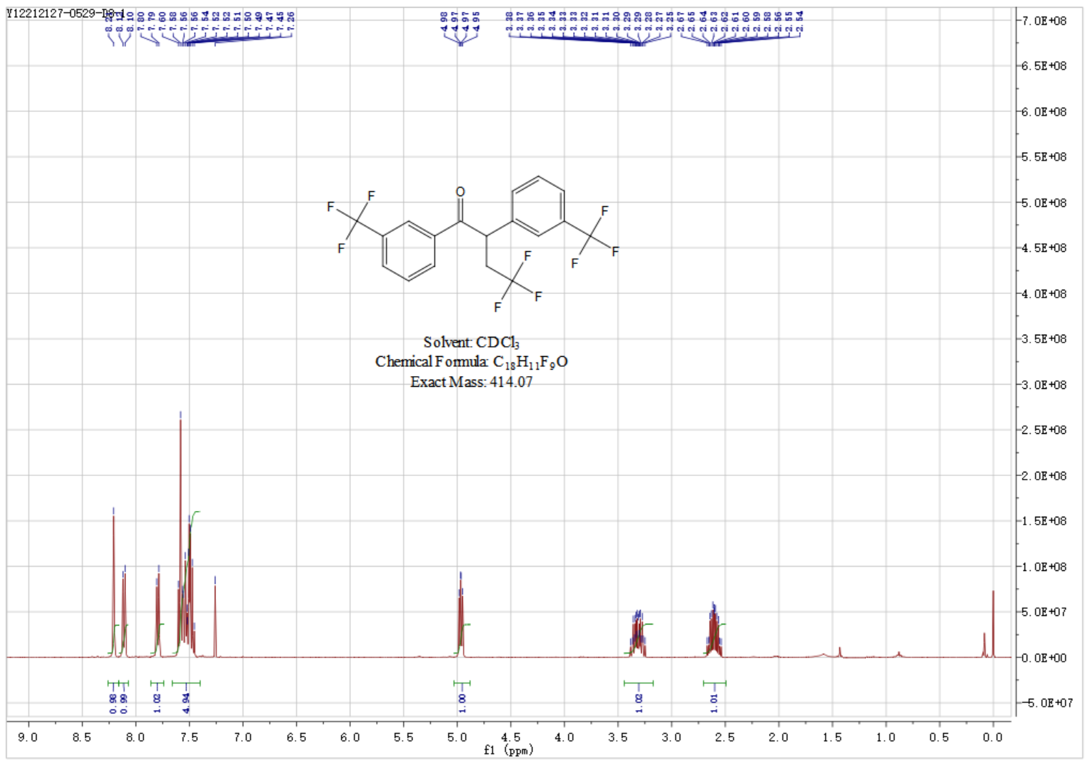


Figure S95. The ^1^H NMR of compound **3g.**


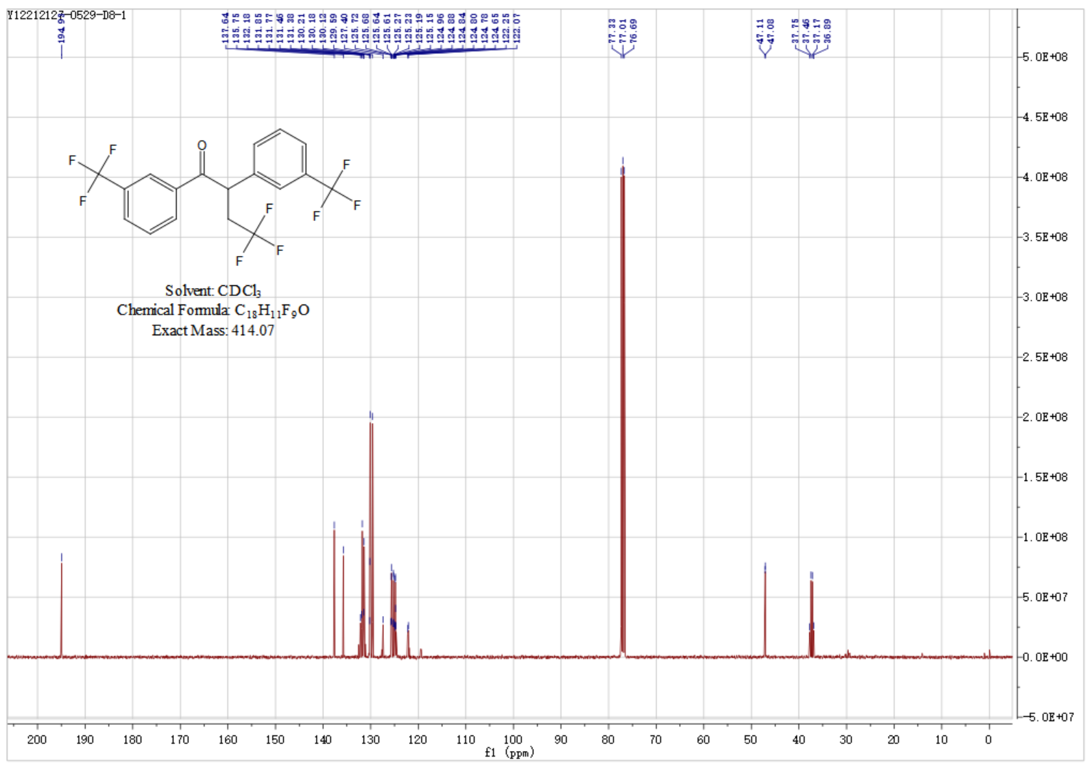


Figure S96. The ^13^C NMR of compound **3g.**


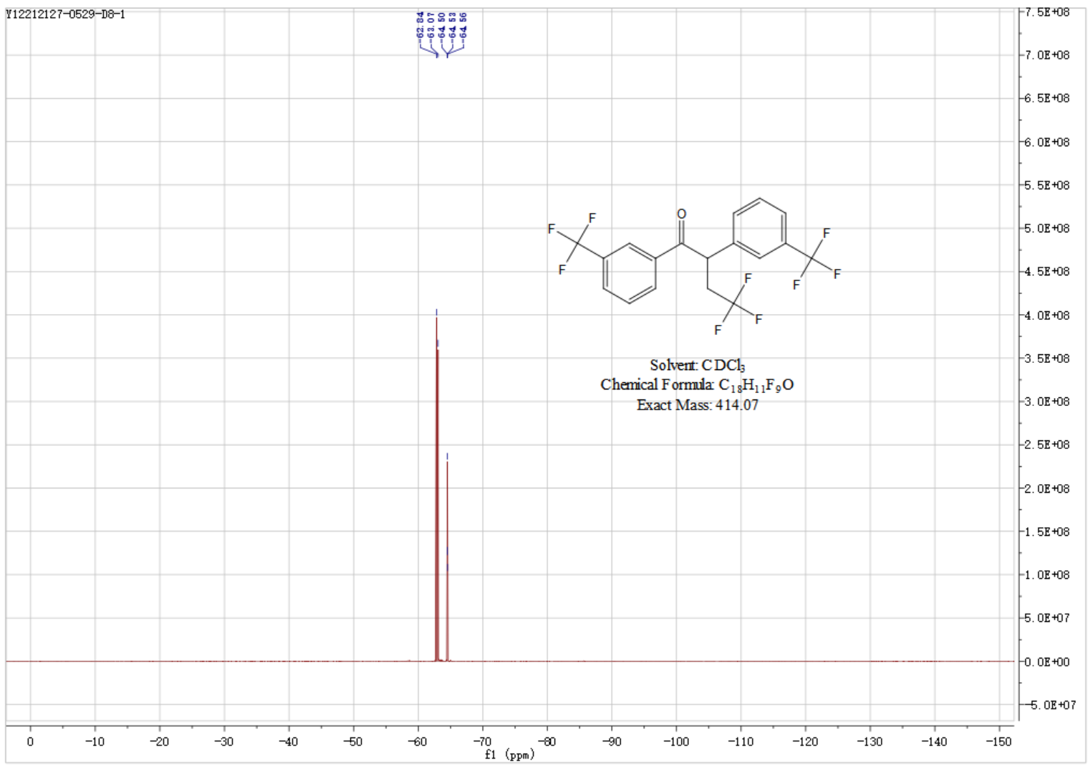


Figure S97. The ^19^F NMR of compound **3g.**


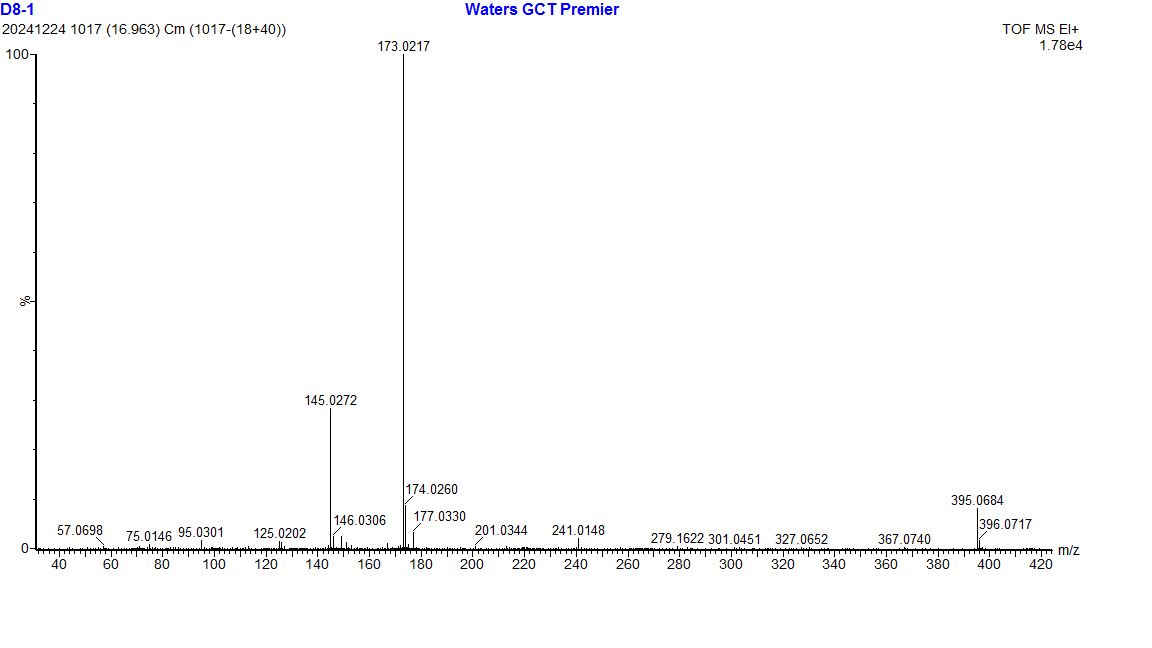


Figure S98. The HR-MS of compound **3g.**


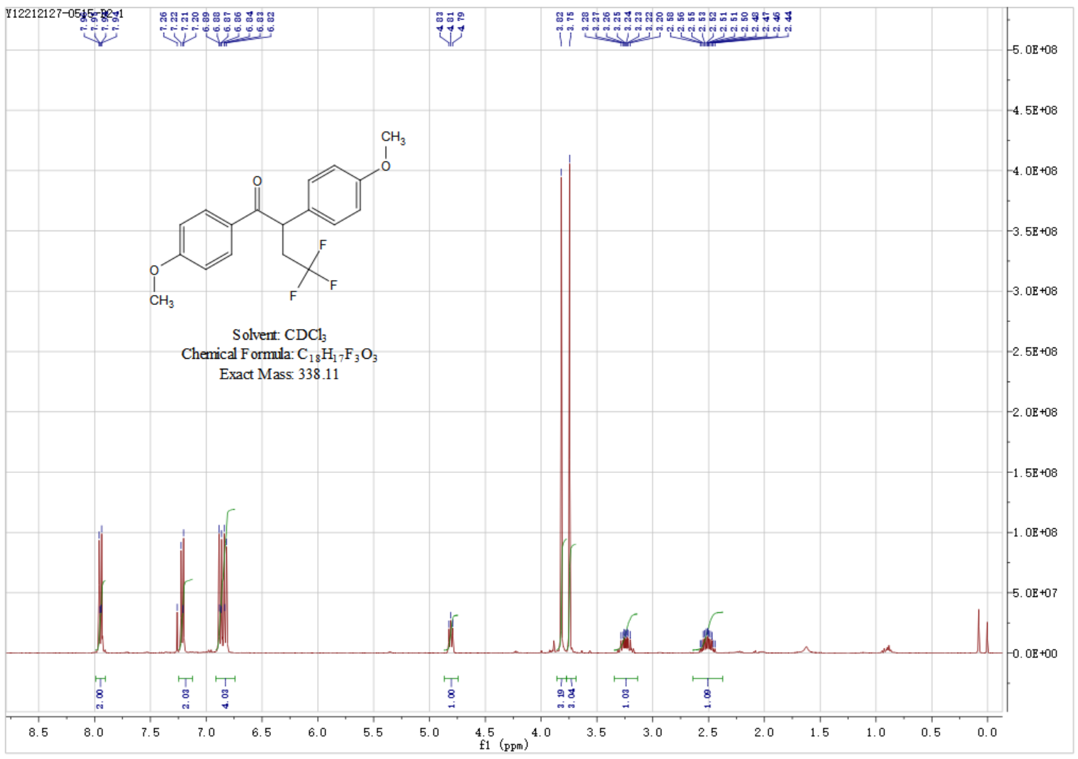


Figure S99. The ^1^H NMR of compound **3h.**


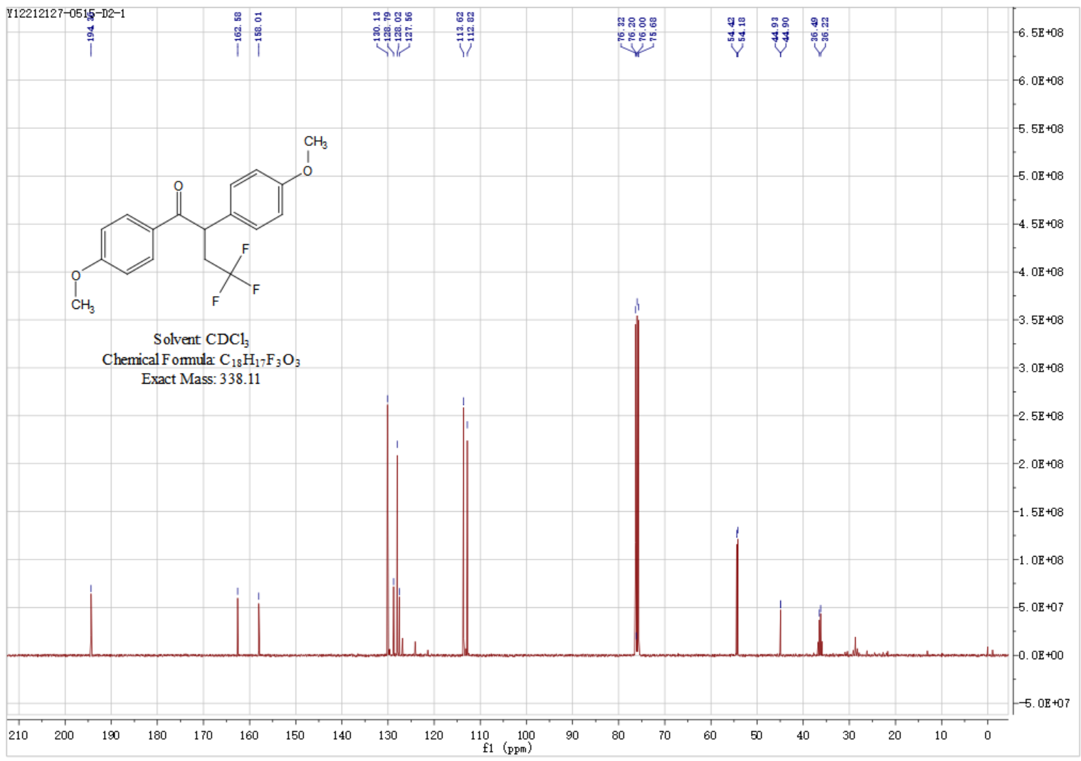


Figure S100. The ^13^C NMR of compound **3h.**

Figure S101. The ^19^F NMR of compound **3h.**

Figure S102. The HR-MS of compound **3h.**

Figure S103. The ^1^H NMR of compound **3i.**

Figure S104. The ^13^C NMR of compound **3i.**

Figure S105. The ^19^F NMR of compound **3i.**

Figure S106. The ^1^H NMR of compound **3i’.**

Figure S107. The ^13^C NMR of compound **3i’.**

Figure S108. The ^19^F NMR of compound **3i’.**

Figure S109. The HR-MS of compound **3i.**

Figure S110. The ^1^H NMR of compound **3j.**

Figure S111. The ^13^C NMR of compound **3j.**

Figure S112. The ^19^F NMR of compound **3j.**

Figure S113. The ^1^H NMR of compound **3j’.**

Figure S114. The ^13^C NMR of compound **3j’.**

Figure S115. The ^19^F NMR of compound **3j’.**

Figure S116. The HR-MS of compound **3j.**

Figure S117. The ^1^H NMR of compound **3k.**

Figure S118. The ^13^C NMR of compound **3k.**

Figure S119. The ^19^F NMR of compound **3k.**

Figure S120. The HR-MS of compound **3k.**

Figure S121. The ^1^H NMR of compound **3l.**

Figure S122. The ^13^C NMR of compound **3l.**

Figure S123. The ^19^F NMR of compound **3l.**

Figure S124. The ^1^H NMR of compound **3l’.**

Figure S125. The ^13^C NMR of compound **3l’.**

Figure S126. The ^19^F NMR of compound **3l’.**

Figure S127. The HR-MS of compound **3l.**

Figure S128. The ^1^H NMR of compound **3m.**

Figure S129. The ^13^C NMR of compound **3m.**

Figure S130. The ^19^F NMR of compound **3m.**

Figure S131. The HR-MS of compound **3m.**

Figure S132. The ^1^H NMR of compound **3n.**

Figure S133. The ^13^C NMR of compound **3n.**

Figure S134. The ^19^F NMR of compound **3n.**

Figure S135. The HR-MS of compound **3n.**

Figure S136. The ^1^H NMR of compound **3o.**

Figure S137. The ^13^C NMR of compound **3o.**

Figure S138. The ^19^F NMR of compound **3o.**

Figure S139. The ^1^H NMR of compound **3o’.**

Figure S140. The ^13^C NMR of compound **3o’.**

Figure S141. The ^19^F NMR of compound **3o’.**

Figure S142. The HR-MS of compound **3o.**

Figure S143. The ^1^H NMR of compound **3p.**

Figure S144. The ^13^C NMR of compound **3p.**

Figure S145. The ^19^F NMR of compound **3p.**

Figure S146. The HR-MS of compound **3p.**

Figure S147. The ^1^H NMR of compound **3q.**

Figure S148. The ^13^C NMR of compound **3q.**

Figure S149. The ^19^F NMR of compound **3q.**

Figure S150. The HR-MS of compound **3q.**

Figure S151. The ^1^H NMR of compound **3r.**

Figure S152. The ^13^C NMR of compound **3r.**

Figure S153. The ^19^F NMR of compound **3r.**

Figure S154. The HR-MS of compound **3r.**

Figure S155. The ^1^H NMR of compound **3s.**

Figure S156. The ^13^C NMR of compound **3s.**

Figure S157. The ^19^F NMR of compound **3s.**

Figure S158. The HR-MS of compound **3s.**

Figure S159. The ^1^H NMR of compound **3t.**

Figure S160. The ^13^C NMR of compound **3t.**

Figure S161. The ^19^F NMR of compound **3t.**

Figure S162. The HR-MS of compound **3t.**

Figure S163. The ^1^H NMR of compound **3u.**

Figure S164. The ^13^C NMR of compound **3u.**

Figure S165. The ^19^F NMR of compound **3u.**

Figure S166. The ^1^H NMR of compound **3u’.**

Figure S167. The ^13^C NMR of compound **3u’.**

Figure S168. The ^19^F NMR of compound **3u’.**

Figure S169. The HR-MS of compound **3u.**

Figure S170. The ^1^H NMR of compound **3v.**

Figure S171. The ^13^C NMR of compound **3v.**

Figure S172. The ^19^F NMR of compound **3v.**

Figure S173. The HR-MS of compound **3v.**

Figure S174. The ^1^H NMR of compound **3w.**

Figure S175. The ^13^C NMR of compound **3w.**

Figure S176. The ^19^F NMR of compound **3w.**

Figure S177. The HR-MS of compound **3w.**

Figure S178. The ^1^H NMR of compound **3x.**

Figure S179. The ^13^C NMR of compound **3x.**

Figure S180. The ^19^F NMR of compound **3x.**

Figure S181. The HR-MS of compound **3x.**

Figure S182. The ^1^H NMR of compound **5j.**

Figure S183. The ^13^C NMR of compound **5j.**

Figure S184. The ^19^F NMR of compound **5j.**

Figure S185. The HR-MS of compound **5j.**

Figure S186. The ^1^H NMR of compound **6j.**

Figure S187. The ^13^C NMR of compound **6j.**

Figure S188. The ^19^F NMR of compound **6j.**

Figure S189. The HR-MS of compound **6j.**
